# Large-area two-dimensional MoO_3_ as a high-*κ* dielectric for van der Waals integration

**DOI:** 10.1038/s41467-026-75196-1

**Published:** 2026-07-07

**Authors:** Zhenliang Hu, Bei Zhao, Qiang Fu, Huiyan Guan, Xiaozhi Liu, Tong Yang, Zijie Feng, Xiaoya Liu, Weiqiao Xia, Xudong Sun, Zucheng Zhang, Yang Wang, Yinzhu Chen, Yutian Yang, Shaopeng Feng, Xiao Tang, Yong Zhang, Jiahao Chen, Xinlei Zhang, Taotao Li, Zejun Li, Yanpeng Liu, Ming Yang, Yue Luo, Jing Wu, Hongwei Liu, Zhenhua Ni, Yuan Liu, Junpeng Lu

**Affiliations:** 1 School of Physics and Key Laboratory of Quantum Materials and Devices of Ministry of Education, Southeast University, Nanjing, China; 2 Beijing National Laboratory for Condensed Matter Physics, Institute of Physics, Chinese Academy of Sciences, Beijing, China; 3 Department of Applied Physics, The Hong Kong Polytechnic University, Hong Kong SAR, China; 4 Jiangsu Key Laboratory for Science and Applications of Molecular Ferroelectrics, Southeast University, Nanjing, China; 5 Hunan Key Laboratory of Two-Dimensional Materials, College of Chemistry and Chemical Engineering, Hunan University, Changsha, China; 6 State Key Laboratory of Mechanics and Control of Mechanical Structures, and Institute for Frontier Science, Nanjing University of Aeronautics and Astronautics, Nanjing, China; 7 National Laboratory of Solid State Microstructures, School of Electronic Science and Engineering and Collaborative Innovation Center of Advanced Microstructures, Nanjing University, Nanjing, China; 8 School of Electronic Science & Engineering, Southeast University, Nanjing, China; 9 Jiangsu Key Lab on Opto-Electronic Technology, School of Physics and Technology, Nanjing Normal University, Nanjing, China; 10 School of Physics and Electronics, Hunan University, Changsha, China

**Keywords:** Two-dimensional materials, Electronic devices

## Abstract

Large-area high-quality dielectrics are essential for the integration of two-dimensional (2D) transistors. However, this integration remains challenging, especially for conventional dielectrics that require deposition processes. Adopting van der Waals (vdW) dielectrics is attractive. Molybdenum trioxide (α-MoO_3_) has been demonstrated as a vdW dielectric in 2D field-effect transistors (FETs), but previous demonstrations showed limited performance and small size. Here, we grow large-area single-crystalline MoO_3_ with uniform thickness and explore its properties as a dielectric. Single-crystalline MoO_3_ reaches the millimeter scale and maintains a high dielectric constant of ~ 18.5, low gate leakage of approximately 10^−2^ A cm^−2^ at 3.0 MV cm^−1,^ and a large breakdown field of ~ 26.75 MV cm^−1^. Top-gated molybdenum disulfide (MoS_2_) transistors exhibit an average subthreshold swing of ~ 71 mV dec^−1^, and an on/off current ratio exceeding 10^8^. Furthermore, we demonstrate MoO_3_-based integrated circuits and low-power complementary metal-oxide-semiconductor (CMOS) inverters.

## Abstract (web summary)

The large-scale integration of high-κ dielectric materials with 2D semiconductors is important for the miniaturization of 2D electronics. Here, the authors report the growth of millimeter-scale high-κ MoO_3_ crystalline films and their application as gate dielectric for 2D transistors and complementary logic devices.

## Introduction

Two-dimensional (2D) semiconductors such as transition metal dichalcogenides (TMDs) are considered promising candidates for functional transistors and integrated circuits owing to their atomically thin-body thicknesses and exceptional electrical and optoelectronic properties^1–7^. Integrating 2D semiconductors with high-*κ* dielectrics is important but has proven challenging. The atomic layer deposition (ALD) process has been widely applied for depositing high-*κ* dielectrics (such as ZrO_2_ or HfO_2_) on semiconducting channels, which works well in silicon technology. However, applying the existing state-of-the-art ALD process to 2D semiconductors is challenging due to their pristine surface without any dangling bonds for chemical reactions^8^. Notable progress has been made by transferring Hf- and Zr-based TMDs as precursor layers and then oxidizing them into high-*κ* dielectrics^9–11^. While this approach has opened a viable route for dielectric integration on 2D semiconductors, it relies on multiple processing steps, and the resulting uniformity and interface quality depend sensitively on the precise control of the oxidation process.

As an alternative, van der Waals (vdW) dielectrics have been applied as the gate dielectric, which can be physically laminated on 2D semiconductors without the strict requirement of surface dangling bonds or lattice matching. Currently, hexagonal boron nitride (h-BN) is the dominant vdW insulator and has been widely used for various proof-of-concept devices. However, as a dielectric material, h-BN exhibits a low dielectric constant ( ~ 3.0-5.1) and large leakage current^12–15^. Additionally, the synthesis of h-BN with sufficient size, uniformity, and thickness is also challenging^16^. These shortcomings limit h-BN’s practical applications for low-power electronic devices and integrated circuits. Alternatively, quasi-vdW-surface materials like calcium fluoride (CaF_2_)^17^ and perovskite strontium titanium oxide (SrTiO_3_)^18–20^ have been recently reported to have high dielectric constants and atomically flat surfaces, and hence improved electrical performance as dielectrics for 2D transistors. However, these dielectrics have alternating cations and anions that are chemically bonded within adjacent layers, leading to an unsaturated dielectric surface with interfacial traps and a degraded interface compared to vdW dielectrics. Thus, there is currently a pressing need to develop a large-scale, high-quality, and high-*κ* dielectric with a vdW structure for advanced 2D electronics. As a high-*κ* vdW material, single-crystalline molybdenum trioxide (α-MoO_3_) has been demonstrated to be feasible as a dielectric layer for 2D field-effect transistors (FETs)^21^. However, the potential of growing large-area uniform MoO_3_ with atomic thickness^22,23^ and employing atomically thin MoO_3_ to fabricate 2D FETs^21,23–25^ with high performance has remained largely unexplored in previous demonstrations, where both scalability and device performance are severely underestimated.

In this work, we grew large-area single-crystalline MoO_3_ and demonstrated its potential as a gate dielectric. The single-crystalline MoO_3_ with atomic and uniform thickness was synthesized via the rapid cooling-induced vdW epitaxy method, with a lateral size over the millimeter scale. It has a high dielectric constant of ~18.5, an equivalent oxide thickness (EOT) of ~0.42 nm, a large electric breakdown field of ~26.75 MV cm^−1^, and a low gate leakage current of approximately 10^−2^ A cm^−2^ at 3.0 MV cm^−1^ at an ultrathin thickness of ~2.0 nm. Using MoO_3_ as the gate dielectric layer, molybdenum disulfide (MoS_2_) FETs exhibited a high on-off ratio over 10^8^ and an average low subthreshold swing (SS) down to ~71 mV dec^−1^. Importantly, MoO_3_ exhibits potential in integrated circuits and low-power complementary metal-oxide-semiconductor (CMOS) inverter, which exhibited low power consumption of 18 pW/μm at *V*_DD_ = 1.0 V.

## Results

### Epitaxial growth of large-area MoO_3_

**Fig. 1:**
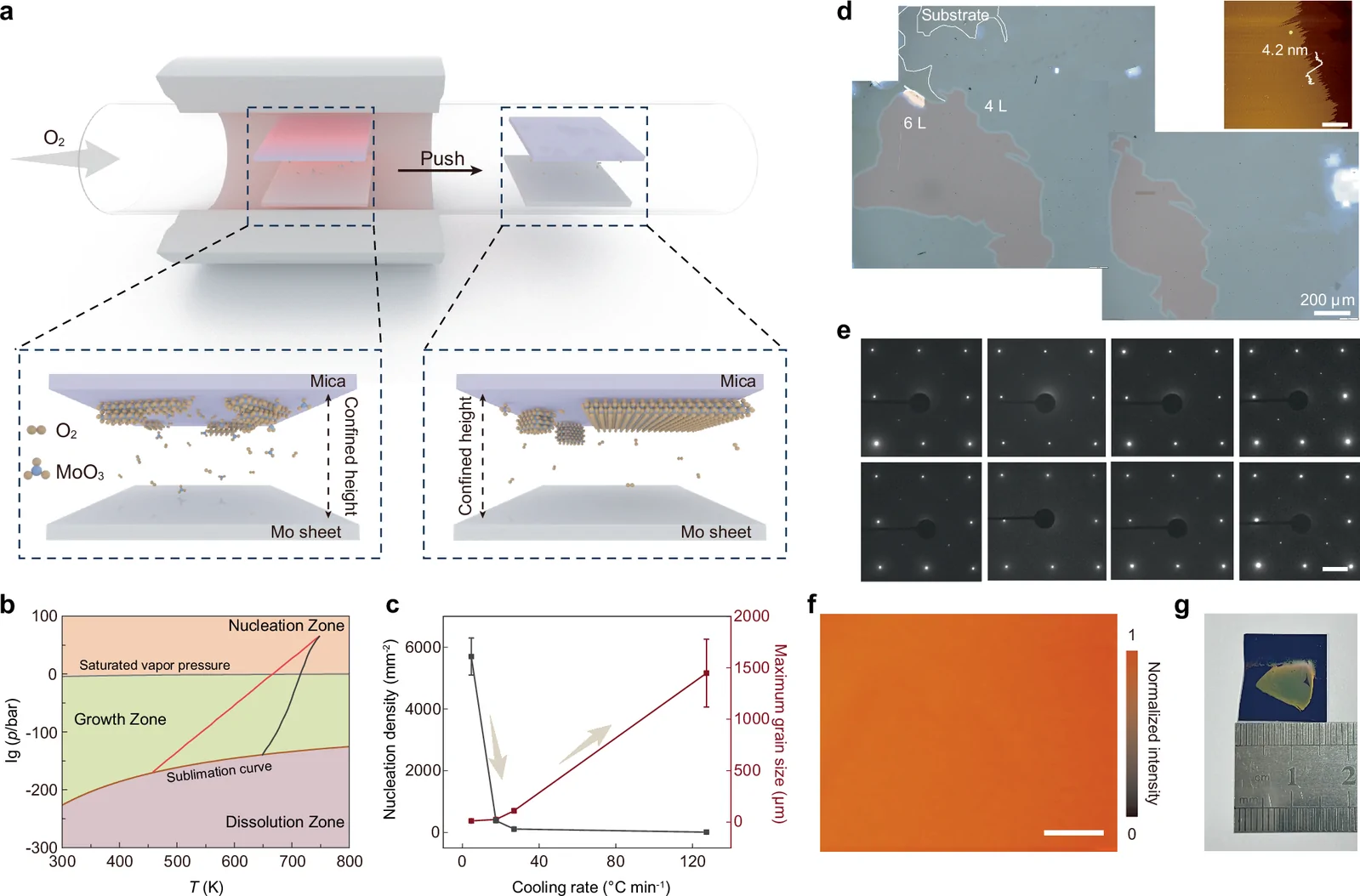
Epitaxial growth of 2D large-area MoO_3_ film. **a** Schematic illustration of the fast-cooling strategy for synthesizing large-area MoO_3_ single-crystalline films. **b** Temperature-dependent sublimation and saturated vapor pressure curves of MoO_3_ growth. Stages of increasing temperature and rapidly cooling are presented by the dark and red curves, respectively. *T*, temperature. *P*, vapor pressure. **c** The maximum grain size and nucleation density of MoO_3_ flakes with different cooling rates. Error bars represent the standard deviation of measurements. **d** Optical image of as-grown MoO_3_ single crystals on mica, the size of six-layer (6 L) MoO_3_ and four-layer (4 L) MoO_3_ are approximately 1.2 mm and 2.5 mm, respectively. Scale bar, 200 μm. Inset is the atomic force microscopy (AFM) image at the edge of the as-grown MoO_3_ film with a thickness of ~4.2 nm. Scale bar, 10 μm. **e** Representative selected area electron diffraction (SAED) patterns of a continuous MoO_3_ film, indicating the single domain orientation of the MoO_3_ film. Scale bar, 2 nm^-1^. **f** Raman mapping (integration of peak 820 cm^-1^) of the as-grown MoO_3_ film. Scale bars, 100 μm. **g** Photograph of centimeter-scale MoO_3_ polycrystalline films on SiO_2_/Si substrate.

Large-area 2D MoO_3_ films could be synthesized on the layered mica substrate via the rapid cooling-induced vdW epitaxy method (see Methods for details). The growth mainly involves two steps (Fig. 1a): (1) during the temperature increasing and holding stage, MoO_3_ evaporates and deposits onto the mica substrate; (2) during the rapid cooling stage (127 °C/min) (the substrate was pushed out of the tube furnace), MoO_3_ rapidly diffuses and grows horizontally.

Figure 1b shows temperature-dependent sublimation and saturation vapor pressure curves for MoO_3_ growth, which separate the processing zone into nucleation, growth, and sublimation parts. During the temperature-holding stage, MoO_3_ is formed by the oxidation of Mo atoms and rapidly evaporates upwards. As the temperature increases, the oxidation and volatilization processes are accelerated, leading to higher vapor flux and partial pressure of gaseous MoO_3_. In the fast-cooling stage, the equilibrium vapor pressure of MoO_3_ decreases dramatically. Therefore, a supersaturated vapor environment is created near the mica surface. This supersaturation provides the thermodynamic driving force for nucleation and subsequent crystal growth, with the chemical potential difference given by Δ*μ* = R*T* ln(*p*/*p*_eq_), where *p* and *p*_eq_ are the actual and equilibrium vapor pressures of MoO_3_, respectively. Under a slow cooling rate (4.6 °C/min) (keep the substrate in the tube furnace), prolonged exposure to MoO_3_ vapor leads to continuous deposition and repeated nucleation, leading to small ( ~ 13 μm), thicker ( ~ 300 nm), and amorphous MoO_3_ (Supplementary Fig. 1a). In contrast, increasing the cooling rate from 4.6 °C/min to 127.0 °C/min substantially shortens the effective deposition window, suppressing sustained nucleation from 5700 mm^-2^ to 11 mm^-2^. The existing nuclei can capture more incoming species and expand laterally due to the larger Δ*μ*, increasing the grain size from ~13 μm to ~1.8 mm (Fig. 1c and Supplementary Fig. 1). In addition, the thickness of MoO_3_ could be controlled by the growth temperature. The thickness decreased from 260 nm to 10 nm as we lowered the growth temperature from 570 °C to 510 °C (Supplementary Fig. 2). This method can also be applied to increase the domain size of transition metal dichalcogenides, such as WSe_2_ from 20 to 444.12 μm (Supplementary Note 1).

The optical images of large-scale single-crystalline MoO_3_ on mica are presented in Fig. 1d. The shapes of MoO_3_ are outlined for clarity, with the mica substrate, six-layer (6 L) MoO_3_, and four-layer (4 L) MoO_3_ labeled. The sizes of 6 L MoO_3_ and 4 L MoO_3_ are approximately 1.2 and 2.5 mm, respectively. The layer numbers of MoO_3_ were determined by the atomic force microscope (AFM) characterization (Supplementary Fig. 3), revealing the uniformity and flatness of ~4.2 nm MoO_3_ single crystal. Over a relatively large scan area of 40 × 40 μm, most regions exhibit an RMS roughness of 1-2 nm, indicating good large-area surface uniformity (Supplementary Fig. 4). In selected local regions, the roughness is even lower, reflecting the intrinsic local flatness and nanoscale thickness homogeneity of the film. These features are important for high-quality vdW integration and high-performance FETs. Figure 1e shows selected area electron diffraction (SAED) patterns measured at eight random locations within the large area MoO_3_ film region (Supplementary Fig. 5). The essentially identical SAED patterns provide strong evidence for the single-crystalline nature of the grown large-area MoO_3_ films. In the Raman spectrum (Supplementary Fig. 6a), the representative Raman peaks at 158 and 820 cm^−1^ are associated with *A*_g_ and *B*_1g_ modes, and those at 286 and 666 cm^-1^ are associated with *B*_2g_ and *B*_3g_ modes, which match well with the reported MoO_3_ Raman signals^22,23^. In addition, no apparent changes in the positions and line widths of the characteristic peaks (Supplementary Fig. 6b) are observed in the room-temperature Raman spectra, obtained from randomly selected 30 regions on the large-area MoO_3_ films, together with Raman mapping (500 × 400 μm^2^), further demonstrating the uniformity of the MoO_3_ single crystal (Fig. 1f). Before electrical characterization, the as-grown large-area single-crystalline MoO_3_ was transferred from mica to SiO_2_/Si substrate using the water-assisted transfer method (Supplementary Fig. 7). The transferred MoO_3_ film on SiO_2_/Si substrate has a lateral size of approximately 1.6 mm (Supplementary Fig. 8) and an average thickness of approximately 5.5 nm (Supplementary Fig. 9), indicating an 8-layer MoO_3_ (Supplementary Fig. 10). However, there are some cracks and wrinkles of MoO_3_ on the SiO_2_/Si substrate, which are introduced in the transfer process. In addition, the water-assisted transfer method could be used to transfer centimeter-scale MoO_3_ polycrystalline film onto the SiO_2_/Si substrate (Fig. 1g), highlighting the capability of the transfer process for large-area film.

The XPS results in Supplementary Fig. 11 confirm that the as-grown film is stoichiometric MoO_3_ with a low density of oxygen vacancies. The XRD patterns in Supplementary Fig. 11 only exhibit (0, k, 0) diffraction peaks, indicating that MoO_3_ is highly oriented along the **b** direction. The cross-sectional high-angle annular dark-field scanning transmission electron microscopy (HAADF-STEM) image reveals the layered structure of MoO_3_, consistent with the simulated single-layer structure of MoO_3_, which is composed of two distorted MoO_6_ octahedral layers, as shown in Supplementary Fig. 12. The adjacent layer distance is approximately 0.74 nm (Supplementary Fig. 12a), close to the simulated result of 0.70 nm (Supplementary Fig. 13) and AFM result of 0.72 nm (Supplementary Fig. 14). The extracted in-plane unit-cell lengths (Supplementary Fig. 12b) are in great agreement with the reported unit-cell lengths^26–28^. In addition, the absorption spectrum (Supplementary Fig. 15) indicates that the bandgap of MoO_3_ flake is approximately 3.60 eV, in reasonable agreement with the value of 3.13 eV obtained from density functional theory (DFT) calculations with the HSE06 hybrid functional (see Methods and Supplementary Fig. 16). While the bandgap of MoO_3_ is smaller than that of h-BN and some other wide-bandgap dielectrics, its dielectric constant is significantly higher, as will be discussed later. This highlights an important trade-off for vdW dielectrics^28^ and conventional oxide dielectrics^29^, that is, larger-bandgap insulators generally provide weaker dielectric screening, whereas higher-*κ* dielectrics typically come with more moderate bandgaps. Furthermore, to confirm the pure vdW nature of the synthesized MoO_3_, the interlayer binding energy was also calculated through DFT simulations. As shown in Supplementary Fig. 17, the binding energies of graphene and 2H-MoS_2_ are 21.0 meV/Å^2^ and 17.7 meV/Å^2^, respectively, which are in agreement with literature results^30^. The binding energy of MoO_3_ is approximately 17.1 meV/Å^2^, close to that of other vdW materials (graphene and 2H-MoS_2_) and much smaller than that of quasi-vdW material (CaF_2_), indicating that the MoO_3_ surface is fully saturated.

### Dielectric properties of MoO_3_

**Fig. 2:**
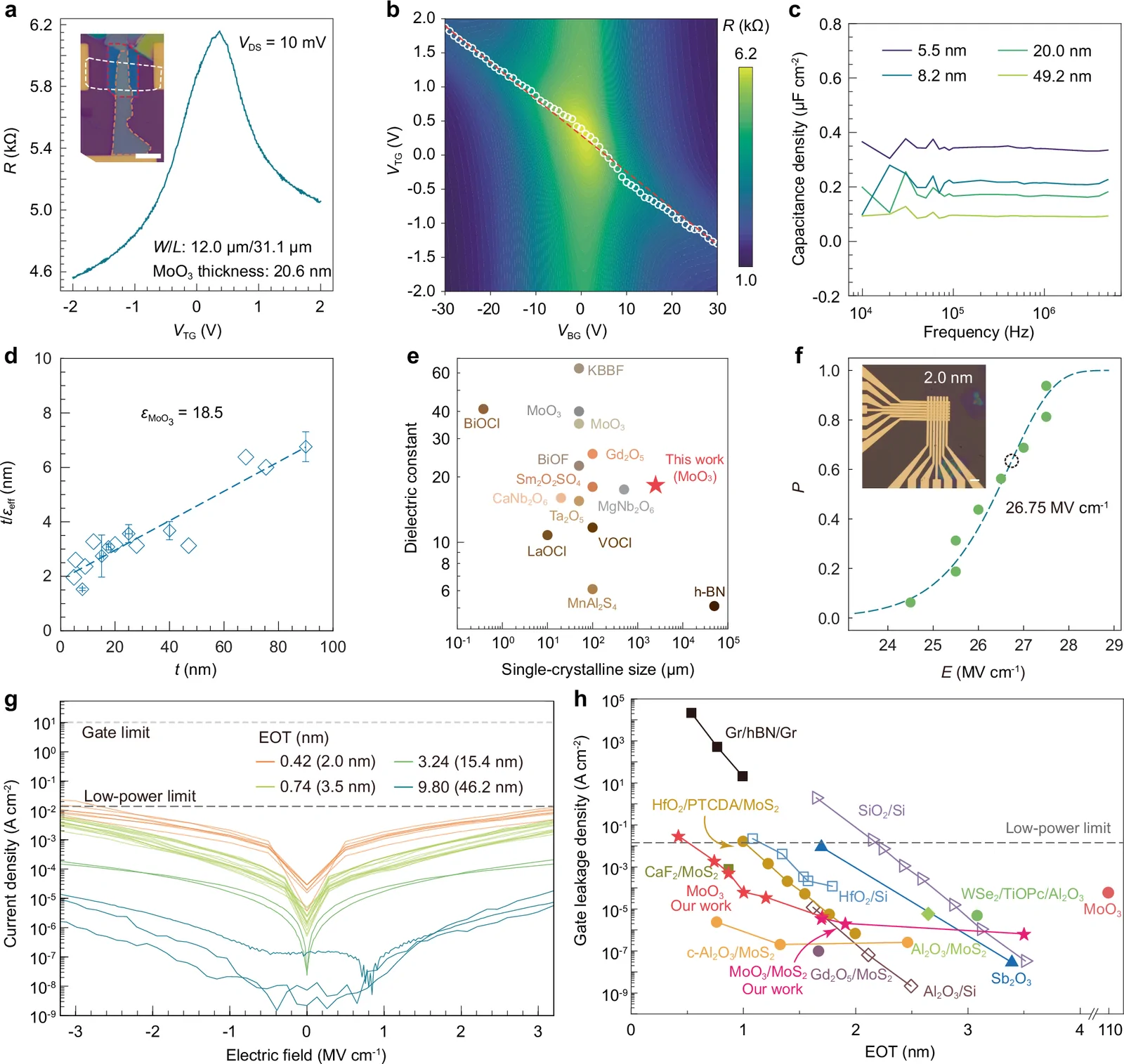
Dielectric properties of MoO_3_. **a** Transfer curve of single-layer graphene using MoO_3_ as the top-gate dielectric *V*_DS_, drain-source voltage. *V*_TG_, top-gate voltage. *W*, Channel width. *L*, channel length. Inset: optical image of the device, the top-FLG, MoO_3_, bottom-FLG are outlined in orange, red, and white colored dashed lines, respectively. The scale bar is 10 μm. **b** Sheet resistance of graphene as a function of the top- and bottom-gate voltages. The Dirac points are marked as white circles, and the red dashed line is the linear fitting of the top-gate Dirac points as a function of *V*_BG_. *V*_BG_, back-gate voltage. **c** Capacitance-voltage characterization (*C*-*V*) of MoO_3_ flakes with four different thicknesses. Note that the background signal had been subtracted. The background of the *C*-*V* test is also plotted (Supplementary Fig. 19b). **d** *t*/*ε*_eff_ as a function of MoO_3_ thickness. The dashed line is the fitting result, and the bulk dielectric constant is approximately 18.5, as extracted from the slope. t, thickness of MoO_3_. *ε*_eff_, effective dielectric constant of MoO_3_. Devices with MoO_3_ thicknesses differing less than 10% were grouped into the same thickness category, and the corresponding mean values and standard deviations for each group were calculated and plotted using the error bar. A linear fit based on the combined dataset was then performed. **e** Lateral size of single-crystalline dielectric versus dielectric constant of state-of-the-art vdW dielectrics, including BiOCl^61^, LaOCl^62^, Ta_2_O_5_^63^, VOCl^64^, Gd_2_O_5_^65^, MnAl_2_S_4_^66^, CaNb_2_O_6_^67^, MgNb_2_O_6_^68^, Sm_2_O_2_SO_4_^69^, BiOF^28^, KBBF^70^, MoO_3_^21,71^, and h-BN^16^. **f** The plot of cumulative failure probability *P* versus electric field, together with the Weibull fitting curve calculated using *β* and *E*_0_. *E*_0_ is the electric field when *P* is approximately 63.2%, and *β* is the shape parameter representing the distribution spread. The dashed circle marks *E*_0_, which is approximately 26.75 MV cm^-1^. Inset is the optical image of the device with a scale bar of 40 μm. **g** Leakage current density of single-crystalline MoO_3_ at different EOT as a function of applied electric fields. The physical thicknesses are supplied for convenience. EOT, equivalent oxide thickness. **h** Leakage current density versus EOT measured at the standard operating voltage of 1 V for Si-based^72–74^ (open symbols) and 2D material-based^17,31,48,65,71,75–78^ structures (filled symbols).

To evaluate the performance of MoO_3_ as a gate dielectric material in transistors, the dielectric constant was extracted via the dual-gate transfer curve method of graphene and capacitance-voltage (*C*-*V*) measurements. The graphene transistor is a three-layer vdW heterostructure in which MoO_3_ is sandwiched between the bottom and top few graphene layers (FLG). The outlines of the graphene and MoO_3_ layers are depicted in white and red (inset in Fig. 2a). During the measurements, the source-drain voltage (*V*_DS_) was maintained at 1 mV, the top-gate voltage (*V*_TG_) was swept from −2 V to 2 V, and the bottom gate voltage (*V*_BG_) was adjusted from -30 V to 30 V with a step of 1 V. Figure 2a shows the sheet resistance of graphene with respect to the top-gate voltages, which is a standard transfer curve for graphene^30^. The Dirac point was approximately 0.4 V, indicating the slight p-doping of graphene. In the dual-gate measurement, *V*_TG_ and *V*_BG_ can simultaneously adjust the charge carrier concentration of the graphene channel. As a result, the top-gate transfer characteristics shift with *V*_BG_ (Supplementary Fig. 18). Figure 2b shows the contour map of graphene sheet resistance as a function of *V*_TG_ and *V*_BG_, with white circles denoting the top-gate Dirac points (*V*_Dirac, TG_) of each sweep. If the ideal capacitance model is adopted in the top and bottom gates, the ratio of *V*_Dirac, TG_ to V_BG_ should be inversely proportional to the ratio of the top and bottom capacitances according to the equation *Q* = *CV* (*Q* is the charge quantity, *C* is the capacitance, and *V* is the voltage)^19,31^. By fitting *V*_Dirac, TG_ with respect to *V*_BG_ (Fig. 2b), we extracted a *C*_BG_/*C*_TG_ ratio of approximately 0.0534. Using the capacitance equation $C={A\varepsilon }_{0}\varepsilon /t$, (*A* is the area of the channel, ${\varepsilon }_{0}$ is the vacuum permittivity, ε is the dielectric constant, and *t* is the thickness of dielectric layer), the dielectric constant of MoO_3_ was calculated to be 25.1, considering the thickness of the bottom gate (285 nm) and top-gate dielectric (20.6 nm, as shown in Supplementary Fig. 18), the areas of the bottom and top gate (with a ratio of approximately 5.0), and the dielectric constant of bottom SiO_2_ (~3.9).

In addition, direct measurements of the capacitance were conducted on the Au/MoO_3_/(FLG or Au) structure on the sapphire substrate to eliminate the influence of parasitic capacitance. The measured total capacitance density has no obvious change with the voltage and a moderate decrease with the frequency (Fig. 2c, Supplementary Fig. 19a)^32,33^. The background noise of the *C*-*V* characteristic was less than 20 fF (Supplementary Fig. 19b), indicating the validity of the measurements. By characterizing multiple devices, as shown in Fig. 2d, the results were fitted using the series capacitance model^34^, resulting in an effective capacitance of $t/{\varepsilon }_{{{{\rm{eff}}}}}=t/{\varepsilon }_{{{{\rm{bulk}}}}}+{{{\rm{D}}}}$, where t is the thickness, *ε*_bulk_ is the dielectric constant of MoO_3_ and D is mainly from the interfacial residue and the quantum capacitance of FLG^35,36^ (see Supplementary Note 2 for the discussion of the series capacitance model and the quantum capacitance). The constant D is approximately 1.9 nm, and *ε*_bulk_ is approximately 18.5 from the linear fitting, which is close to the reported value of ~17 of ALD-grown MoO_3_^37^ (Supplementary Table 1). It should be emphasized that this dielectric constant can be extended to ultrathin MoO_3_ due to the thickness-independent dielectric constant that has been demonstrated before^38^. The value ~ 18.5 extracted from the direct capacitor measurement is considered as the intrinsic dielectric constant of MoO_3_, whereas the larger value ~ 25.1 extracted from the dual-gate graphene device is an effective dielectric constant in a specific device geometry, which may include contributions from interfacial residues, parasitic electrostatic coupling, and other non-idealities. Figure 2e summarizes the lateral single-crystalline size versus dielectric constant of state-of-the-art vdW dielectrics. Compared to other vdW dielectrics, MoO_3_ possesses a millimeter size and a high dielectric constant simultaneously, making it an exceptional choice for future monolithic 3D integration.

Additionally, we investigated the electric field at breakdown and leakage current of MoO_3_ at ultralow equivalent oxide thicknesses (EOTs), which are critical parameters and directly related to the power consumption and reliability of a transistor. EOT (${{{\rm{EOT}}}}=3.9{t}_{{{{\rm{ox}}}}}/{\varepsilon }_{{{{\rm{ox}}}}}$) is defined as the thickness of silicon oxide (*κ* = 3.9) that gives the same electrical performance as that of the high-*κ* dielectric material. Figure 2f presents the Weibull statistical analysis of the electric field at breakdown of ~2.0 nm MoO_3_, from which we obtained a characteristic electric field at breakdown *E*_0_ ≈ 26.75 MV cm^-1^ for ~2.0 nm MoO_3_ film at room temperature (see details in Supplementary Note 3). The electric field at breakdown versus dielectric constant of state-of-the-art dielectrics is listed in Supplementary Fig. 20. MoO_3_ has a competitive electric field at breakdown compared to other dielectrics. Compared to the widely used vdW dielectric h-BN, MoO_3_ exhibits a higher dielectric constant and a competitive electric field at breakdown. Moreover, the lifetime characterization suggests a projected lifetime exceeding ten years under an electric field of 2.5 MV cm^-1^ (see details in Supplementary Note 4). Figure 2g presents the leakage currents of MoO_3_ in the metal-insulator-metal structure with different MoO_3_ thicknesses. The leakage current for 2.0 nm MoO_3_ (EOT = 0.42 nm) is approximately 10^−2 ^A cm^-2^ at an electric field ~3.0 MV cm^-1^, meeting the low-power logic limits (1.5 × 10^-2 ^A cm^-2^) set by ITRS (International Technology Roadmap for Semiconductors)^39^. The leakage current densities versus EOT for the dielectrics at 1 V gate voltage are summarized in Fig. 2h. Attributed to the high dielectric constant and vdW interface, MoO_3_ shows the competitive leakage currents compared to other dielectrics at the same EOT.

### MoS_2_ transistor using MoO_3_ as the gate dielectric

**Fig. 3:**
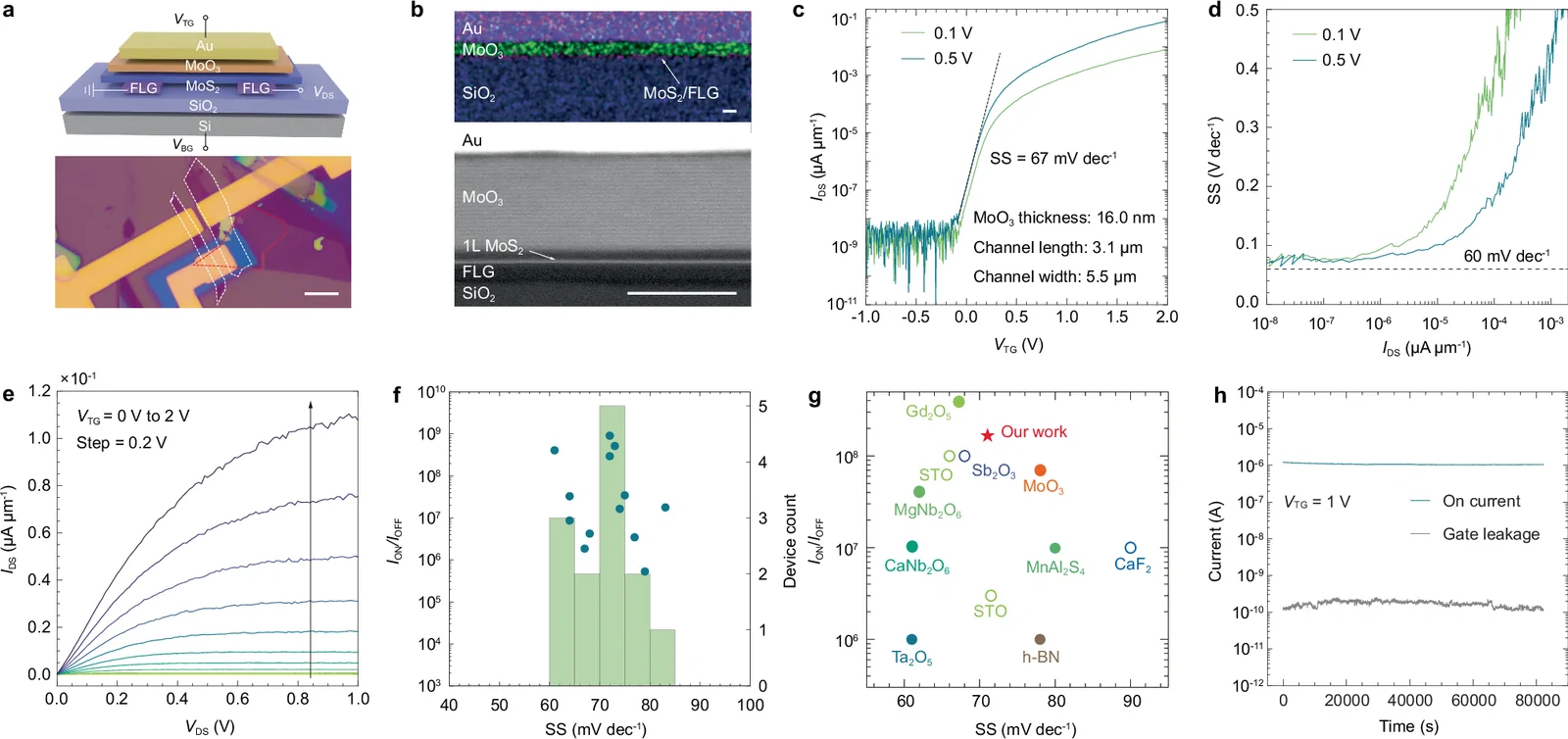
Field-effect transistors with MoO_3_ dielectric. **a** Schematic and optical image of MoS_2_ top-gated field-effect transistors (FETs). FLG marked in white is the source and drain electrodes. MoS_2_ is marked in red. FLG, few-layer graphene. **b** Cross-sectional HAADF-STEM image and EDS mapping of the whole device. Scale bar, 20 nm. HAADF-STEM, high-angle annular dark-field scanning transmission electron microscopy. EDS, energy-dispersive X-ray spectroscopy. **c** Transfer curve of monolayer MoS_2_ in 0.1 V and 0.5 V *V*_DS_, SS is approximately 67 mV dec^-1^. *I*_DS_, drain-source current. SS, subthreshold swing. **d** SS as a function of *I*_DS_. **e** Output curves of MoS_2_ FETs with V_TG_ ranging from 0 V to 2 V. **f** Scatter plot of the on/off current ratios and SS extracted from all micro-scale MoS_2_ FETs, and statistical histogram of SS. **g** *I*_on_/*I*_off_ and SS of our FETs compared to state-of-the-art MoS_2_ transistors using vdW (filled symbols) and quasi-vdW (open symbols) dielectrics, such as h-BN^79^, Ta_2_O_5_^63^, MnAl_2_S_4_^66^, CaF_2_^17^, Sb_2_O_3_^76^, STO^19,20^, Gd_2_O_5_^65^, CaNb_2_O_6_^67^, MgNb_2_O_6_^68^, and MoO_3_^21^. **h** The operational stability of the FETs was evaluated at room temperature in vacuum ( ~ 10^-6 ^Torr) under a continuous gate voltage of 1 V for over 80,000 s.

Molybdenum disulfide (MoS_2_) has been demonstrated to be an ideal semiconductor for high-performance thin-body FETs^40–43^. To evaluate the performance of MoO_3_ as a gate dielectric, monolayer MoS_2_ transistors were fabricated using MoO_3_ as the top-gate dielectric and FLG as the source and drain contacts. Natural FLG contacts were initially prepared on the SiO_2_/Si substrate via mechanical exfoliation, and then monolayer MoS_2_ and MoO_3_ flakes were successively transferred to form a MoO_3_/MoS_2_/FLG heterostructure. Finally, prepatterned gold electrodes were transferred onto the heterostructure to complete the device (Supplementary Fig. 22) (see Methods for more details). The layer number and quality of MoS_2_ were confirmed by photoluminescence (PL) and Raman characterizations (Supplementary Fig. 23). Figure 3a shows the schematic and optical image of the MoS_2_ FET, where FLG and MoS_2_ are marked in white and red, respectively. Figure 3b illustrates the cross-sectional structure of the MoS_2_ top-gate FET via HAADF-STEM, with the materials of each layer being confirmed by energy-dispersive X-ray spectroscopy (EDS) mapping (Supplementary Fig. 24, Supplementary Fig. 25) (Note that EDS mapping and cross-sectional STEM image have different magnifications). The sharp and clean interface between MoO_3_ and Au and between MoO_3_ and MoS_2_ was confirmed from the cross-sectional image. The band alignment of MoS_2_/MoO_3_ is illustrated in Supplementary Fig. 26. The charge redistribution after contact leads to giant potential barriers at the MoS_2_/MoO_3_ interface. The channel length, width, and thickness of MoO_3_ are all labeled in Fig. 3c. By monitoring the drain current when sweeping the *V*_TG_, we collected the top-gate transfer characteristics (*I*_DS_ versus *V*_TG_) on a logarithmic scale (Fig. 3c). The drain current *I*_DS_ was kept on the order of 10^-14 ^A and abruptly increased when *V*_TG_ exceeded the subthreshold voltage (*V*_TH_). The drain current was efficiently modulated from 10^-14 ^A to approximately 0.6 μA at 0.5 V *V*_DS_, showing a high on/off ratio (*I*_on_/*I*_off_) close to 10^8^. The subthreshold swing (SS), which describes the amount of gate voltage needed to increase the drain current by one decade, was approximately 67 mV dec^-1^, close to the thermionic limit of 60 mV dec^-1^ of the transistor at room temperature. Before the drain current started to saturate, SS remained at low values over several orders of magnitude of *I*_DS_ (Fig. 3d). The output characteristics (*I*_DS_ versus *V*_DS_) for various *V*_TG_ are plotted in Fig. 3e. *I*_DS_ eventually saturates at large *V*_DS_. In the small *V*_DS_ region, *I*_DS_ increased linearly with *V*_DS_ (Supplementary Fig. 28), suggesting the presence of an Ohmic contact. To evaluate the reproducibility of the device performance, more devices with the same structure were fabricated. Note that three among those devices were using the usual on-site nano-fabrication process for the metal electrode deposition. All devices exhibited high performance on SS, on-off current ratio, and gate leakage current (Supplementary Fig. 29). SS is related to the interface between MoO_3_ and MoS_2_, given as ${{{\rm{SS}}}}={{\mathrm{ln}}}\left(10\right)\frac{{kT}}{q}\left(1+\frac{{C}_{{{{\rm{it}}}}}}{{C}_{{{{\rm{G}}}}}}\right)$, where *k* and *T* are the Boltzmann constant and absolute temperature, respectively, and ${C}_{{{{\rm{it}}}}}$ is the capacitance of the interface trap states^44,45^. The near-ideal SS suggests a small ${C}_{{it}}/{C}_{G}$ value, indicating the low density of the trap state, as further supported by the hysteresis results (Supplementary Fig. 30), which remain negligible across different sweep rates and voltage steps. The interfacial trap density was further obtained as ~5 × 10^11 ^cm^-2^ eV^-1^ by the frequency-dependent *C*-*V* conductance analysis^46^, ~2 × 10^11 ^cm^-2^ eV^-1^ by the charge pumping method^47^, and ~1.5 × 10^11 ^cm^-2^ eV^−1^ by the low-frequency 1/*f* noise measurements^48,49^, which is competitive to most *D*_it_ values of exfoliated MoS_2_ channels (Supplementary Note 5)^48^. Figure 3f summarizes the distribution of the on/off current ratio and SS of all devices. Most devices have on/off current ratios in the range of 10^6^ and 10^9^, have SS values that are lower than 70 mV dec^−1^, with the lowest one approaching 61 mV dec^−1^. SS and current on/off ratio show no clear dependence on MoO_3_ thickness (Supplementary Fig. 31). In contrast, the threshold voltage exhibits a linear dependence on MoO_3_ thickness, consistent with that of conventional dielectrics. The top-gate (Supplementary Fig. 32) and bottom-gate (Supplementary Fig. 33) MoS_2_ transistors with Au source-drain electrodes also exhibited high performance in terms of SS and gate leakage current. The relatively low current on/off ratio observed in the top-gate devices is mainly attributed to the large ungated regions. Figure 3g summarizes the average SS and on/off ratio of our MoS_2_ transistors with the state-of-the-art few-layer MoS_2_ transistors using vdW or quasi-vdW dielectric materials. Our devices exhibited an average SS ~ 71 mV dec^−1^ and an average on/off ratio over 10^8^. Note that conventional non-vdW dielectrics are not listed here for convenience. Besides, the transistor exhibited stable performance in a vacuum at room temperature. The gate leakage current, on current, off current, and current on/off ratio were maintained nearly constant during the entire test, including the continuous and pulsed bias (Fig. 3h, Supplementary Note 4).

### Large-area device array and the CMOS inverter

**Fig. 4:**
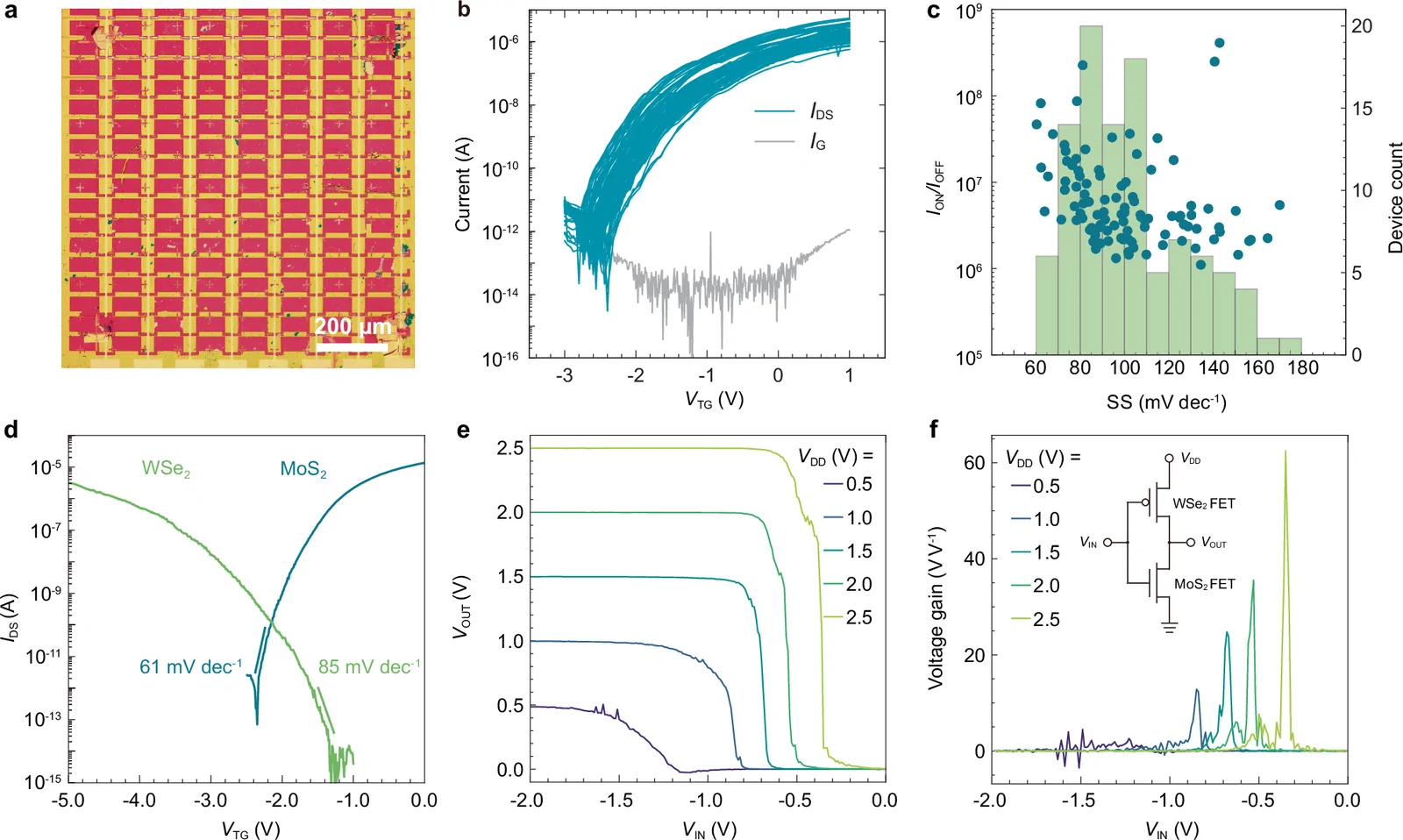
FET device array and low-power complementary metal-oxide-semiconductor inverter. **a** Optical image of the fabricated FET array using the chemical vapor deposition-grown MoS_2_ and MoO_3_ film. **b** Transfer curves of successful FET devices and the gate leakage current. *I*_G_, gate leakage current. **c** Scatter distribution of the on/off current ratios and SS extracted from (**b**), and statistical histogram of SS of those devices. **d** Transfer characteristics of monolayer MoS_2_ N-type FET and WSe_2_ P-type FET, the corresponding SS values are labeled. **e** Measured output voltage as a function of input voltage of the CMOS inverter at *V*_DD_ = 0.5 V, 1.0 V, 1.5 V, 2.0 V, and 2.5 V. CMOS, complementary metal oxide semiconductor. *V*_DD_, supply voltage. *V*_IN_, input voltage. *V*_OUT_, output voltage. **f** Voltage gain as a function of input voltage extracted from (**e**). The structure of our CMOS inverter is schematically illustrated.

To evaluate the integration capability of large-area MoO_3_ film, we fabricated top-gate MoS_2_ FET arrays by integrating MoO_3_ with CVD-grown monolayer MoS_2_, as shown in Fig. 4a (see Methods for details). In our work, a device is defined as successful when it exhibits clear gate-modulated transfer behavior with both *I*_on_/*I*_off_ beyond 10^4^ and *I*_on_ beyond 10^-7 ^A. Devices not meeting these criteria are classified as failed devices. Figure 4b presents the *I*_D_-*V*_G_ curves of successful devices, and the data of the failed devices are provided in Supplementary Fig. 35a. The spatial distribution of *I*_on_/*I*_off_, *I*_on_, and the device yield map are summarized in Supplementary Fig. 35. The overall device yield is approximately 81%, corresponding to 102 functional devices out of 126 fabricated devices. Statistical analysis of the successful device shows relatively uniform performance (Fig. 4c, Supplementary Fig. 36). Specifically, the SS has an average value of ~103 mV dec^-1^, with a standard deviation of ~27 mV dec^-1^. For the on-off ratio, the average and standard deviation were calculated in logarithmic scale, yielding an average log_10_(*I*_on_/*I*_off_) value of ~6.8 and a standard deviation of ~0.59. The field-effect mobility extracted from the transfer curve is summarized in Supplementary Fig. 37, with most devices exhibiting mobilities in the range from 5 to 20 cm^2 ^V^-1^s^-1^. The non-ideal yield and relatively reduced mobility are mainly attributed to local cracking, wrinkles, and interfacial trap states introduced during the large-area integration of MoO_3_ and MoS_2_ films. Those performances can be further improved through continued optimization of the transfer and integration processes.

We further examine the performance of the MoO_3_ single crystal on p-type FETs. We used the monolayer WSe_2_ as the p-type channel and utilized the same top-gate structure in Fig. 3a. The transfer and output characteristics of the WSe_2_ FET are presented in Fig. 4d and Supplementary Fig. 38. Similarly, several WSe_2_ FETs were fabricated to verify the reproducibility (Supplementary Fig. 39). The device exhibited an on/off current ratio of over 10^8^ and an SS value of approximately 85 mV dec^-1^. The potential of MoO_3_-gated FETs in the integrated circuit is evaluated in the CMOS inverter unit. A CMOS inverter comprises a single series-connected n-type and p-type transistors, as shown in Fig. 4f inset. *W*/*L* is 9 μm /2 μm for the MoS_2_ transistor and 4 μm /1.5 μm for the WSe_2_ transistor. The input voltage (*V*_IN_) is applied to the gates of both FETs, resulting in a high or low output voltage (*V*_OUT_) that depends on the on/off states of two transistors. The measured output voltage as a function of input voltage at different *V*_DD_ is presented in Fig. 4e. When the input signal is low (logic ‘0’), the output voltage is close to the supply voltage (*V*_DD_), corresponding to logic ‘1’. Conversely, when the input signal is high (logic ‘1’), the output voltage is close to the ground voltage (logic ‘0’). The voltage gain (-d*V*_OUT_/d*V*_IN_) as a function of input voltage is plotted in Fig. 4f. The peak values of voltage gain are much greater than unity (12.8 VV^-1^ at *V*_DD_ = 1 V and 35.5 VV^-1^ at *V*_DD_ = 2 V). It is worth noting that the threshold voltage of the MoS_2_ transistor could be modulated by the back-gate voltage, as presented in Supplementary Fig. 28b and Supplementary Fig. 40a. The transition range of the input voltage of the CMOS inverter could also be modulated (Supplementary Fig. 40b). Therefore, the CMOS inverter could work properly in a small positive transition region for the input voltage (Supplementary Fig. 40d), and meanwhile, maintain the high voltage gains (Supplementary Fig. 40e). For the power consumption, we calculated the static power consumption, which is equal to *V*_DD_ × *I*_DD,_ as shown in Supplementary Fig. 41. The power exhibited a peak value for supply voltage at 2.0 V and 2.5 V. However, at lower supply voltages, no apparent peak of power consumption was observed, which is a result of 10^-10^-10^-9 ^A gate leakage current in the transition range in the WSe_2_ transistor. In the transition region, the static power consumptions at different supply voltages are ~500 pW/μm for *V*_DD_ = 2.0 V, 0.130 nW/μm for *V*_DD_ = 1.5 V, ~38 pW/μm for *V*_DD_ = 1.0 V, and ~15 pW/μm for *V*_DD_ = 0.5 V. For another CMOS inverter, the power consumption is ~18 pW/μm at *V*_DD_ = 1.0 V (Supplementary Fig. 42), which is competitive as compared to reported 2D CMOS inverters.

## Discussion

In brief, we have synthesized a large-area single-crystalline vdW MoO_3_ film with atomically uniform thickness, and explored its performance in terms of crystalline quality, scalability, and dielectric strength. Owing to its large size and free integration with 2D semiconductors, we demonstrate the capability of MoO_3_ in FET device arrays and promise in the integrated circuit. As a vdW dielectric, MoO_3_ holds tremendous potential for investigating the fundamental 2D physics, such as the application in high-quality device encapsulation, 2D light-emitting diodes (see details in Supplementary Note 6), as well as for constructing high-performance monolithic 3D integration not previously possible. Future work will focus on the direct growth of large-area MoO_3_ on MoS_2_, which could provide an in-situ integration strategy to substantially reduce the interfacial disorder and contamination associated with transfer processes.

## Methods

### Preparation of MoO_3_ films

A sliding-rail chemical vapor deposition (CVD) tube furnace serves as a rapid cooling device, where the heating temperature zone was set at a temperature of 520 °C to ensure that the molybdenum (Mo) metal flake used as an evaporation source could sufficiently evaporate and assist in the subsequent chemical reaction. The layered mica covered the upper surface of the Mo metal flake, with the distance confined within 0.5 mm. Before deposition, the mica substrate was mechanically exfoliated to create a fresh and atomically flat surface. The Mo foil was polished to eliminate the influence of molybdenum oxide formed on the foil surface. Next, the Mo foil and mica substrate were placed inside the tube furnace with the mica’s fresh surface facing downward directly above the Mo foil. The furnace was heated to 520 °C and maintained at this temperature for 6 hours in the atmosphere environment before rapidly cooling to room temperature. Afterwards, a PDMS support was attached to the MoO_3_/mica with the MoO_3_ film sandwiched in the middle. The whole sample was then submerged in deionized water, followed by slightly peeling off the mica from one of the corners. Due to the hydrophilic behavior, the MoO_3_ film was separated from the mica substrate and attached to the PDMS support. The PDMS-supported MoO_3_ sample was ready to be used in the subsequent transfer process after being dried with nitrogen gas.

### Micro-sized device fabrication

For the micro-scale MoS_2_ and WSe_2_ transistor devices with the few-layer graphene (FLG) as the electrodes, monolayer MoS_2_ was mechanically exfoliated onto PDMS, and the single-layer property was confirmed by fluorescence microscopy. The FLG was mechanically exfoliated onto the SiO_2_/Si (285 nm) substrate. Fortunately, it is easy to find two parallelly placed FLG flakes with a separation distance of only several micrometers. These FLG flakes were chosen as the FLG electrodes. The MoS_2_ monolayer was then transferred onto the FLG electrodes, and a MoO_3_ flake was transferred onto the MoS_2_ layer. To avoid damage to the MoO_3_ surface during the thermal deposition of the metal electrodes, the metal electrodes were prefabricated and then dry-transferred to the sample^50,51^. The metal electrodes were made using photolithography and thermal evaporation. A polymethyl methacrylate (PMMA) layer was coated on the device after the electrode transfer process. Finally, the device was annealed at 180 °C for 2 hours before the removal of the PMMA layer in an acetone bath. To test the performance of MoO_3_ on a practical application, a usual on-site nano-fabrication process, including electron beam lithography and thermal deposition, was used on some MoS_2_ top-gate devices. We used FLG as the electrical contact rather than the metal electrodes in the top-gate transistor for the following reasons: (I) clean and sharp vdW contacts between electrodes and channel semiconductors could improve the transistor performance^50–52^, (II) we want the whole channel region to be gate-controlled to further improve the on current with small *V*_DS_, which can only be realized by pre-patterned electrodes in top-gate FETs, and (III) the graphene electrodes are thin enough to eliminate the influence of strain compared to the thick metal electrodes.

### Device array fabrication

The CVD-grown monolayer MoS_2_ film was grown on the sapphire substrate^53^. We first transferred the MoS_2_ film from the sapphire substrate to the SiO_2_/Si substrate using the standard wet transfer method^53^. Then, standard EBL and argon plasma etching processes were used to make the MoS_2_ strips with a width of 5 μm, followed by the fabrication of source and drain electrodes, the transfer of large-area MoO_3_, and the fabrication of gate electrodes. Finally, the device was thermally annealed.

### Electrical measurements

After the removal of the PMMA layer, the substrate was adhered to a chip carrier using silver paste, and the device electrode pads were wire-bonded to the pins. The transistor characterization was conducted using a Keithley 4200 semiconductor parameter analyzer in a vacuum chamber at room temperature. The pressure was approximately 10^-6 ^Torr. For the device array measurement, we used the Lake Shore probe station and Keithley 4200 semiconductor parameter analyzer in a vacuum chamber (10^-4 ^Torr) at room temperature. Capacitance measurements were conducted using a Keithley 4215-CVU module with 4225-RPM remote amplifier on a Lake Shore PS-100 probe station with a shielded enclosure under ambient conditions.

### Optical and structural characterizations

Raman and PL spectra were collected in a homemade optical system with a Horiba iHR550 spectrometer. The thickness of the MoO_3_ flakes and the morphology of the devices were measured by a Bruker Dimension ICON. XRD spectra were collected by Rigaku SmartLab. STEM imaging and EDS analyses were performed using a Titan Cubed Themis G2 300 STEM instrument (200 keV) and a JEOL JEM-ARM300CF S/STEM system (300 keV).

### DFT simulations

All density functional theory (DFT) calculations were carried out using the Vienna Ab Initio Simulation Package (VASP)^54,55^. The projector augmented-wave method and the Perdew-Burke-Ernzerhof (PBE) parametrized generalized gradient approximation were used to describe the core-electron interaction and the exchange-correlation interaction, respectively^56–58^. The electronic wavefunction was expanded using a plane wave basis with a kinetic energy cutoff of 520 eV. A Γ-centered *k*-mesh of 11×10×3 and 11×10×1 were used for Brillouin-zone integrations for bulk and *n* layers of α-MoO_3_, respectively. For the latter, a 20 Å vacuum layer was inserted along the out-of-plane direction to alleviate the spurious interlayer interaction. The convergence criterion was $1.0\times {10}^{-5}{{{\rm{eV}}}}$ for the electronic optimization and $-0.01{{{\rm{eV}}}}/\mathring{\mathrm{A}}$ for the ionic optimization. The DFT-D2 method was also applied to account for the dispersive van der Waals interaction^59^. The optimized lattice constants of bulk α-MoO_3_ are 3.693×3.910×13.929 Å. To obtain an accurate electronic structure and bandgap of α-MoO_3_, the Heyd-Scuseria-Ernzerhof (HSE06) hybrid functional was adopted^60^. The interlayer binding energy (IBE) was defined as ${E}_{{{{\rm{IBE}}}}}=\frac{N\times {E}_{{{{\rm{monolayer}}}}}-{E}_{{{{\rm{bulk}}}}}}{N\times A}$, where ${E}_{{{{\rm{monolayer}}}}}$ and ${E}_{{{{\rm{bulk}}}}}$ are the energy of a monolayer material and its bulk counterpart, respectively. *N* denotes the number of monolayer constituents in the bulk phase, and *A* denotes the interface area.

## Supplementary information

Supplementary Information

Transparent Peer Review file

## Source data

Source Data

## Data Availability

All data supporting the results of this study are available within the paper and Supplementary information. Additional datasets are available from the corresponding author upon request. Source data are provided with this paper.
