# Supplementary material for: Large-area two-dimensional MoO3 as a high-κ dielectric for van der Waals integration
Source: Nat Commun. 2026 Jul 7;17:8371. doi: 10.1038/s41467-026-75196-1 (PMC13473665; doi:10.1038/s41467-026-75196-1)
Supplement: Supplementary file 1 [file 41467_2026_75196_MOESM1_ESM.pdf]

Supplementary Information for

Large-area two-dimensional MoO_3 as a high-κ dielectric for van der Waals integration

Zhenliang Hu[1#], Bei Zhao[1#], Qiang Fu[1#], Huiyan Guan[1], Xiaozhi Liu[2], Tong Yang[3], Zijie Feng[4], Xiaoya Liu[1], Weiqiao Xia[1], Xudong Sun[1], Zucheng Zhang[5], Yang Wang[6], Yinzhu Chen[1], Yutian Yang[1], Shaopeng Feng[7], Xiao Tang[1], Yong Zhang[1], Jiahao Chen[1], Xinlei Zhang[1], Taotao Li[8], Zejun Li[1], Yanpeng Liu[6], Ming Yang[3], Yue Luo[9], Jing Wu[9], Hongwei Liu[10]*, Zhenhua Ni[1,9]*, Yuan Liu[11]*, Junpeng Lu[1,9]*

[1]School of Physics and Key Laboratory of Quantum Materials and Devices of Ministry of Education, Southeast University, Nanjing 211189, China.

[2]Beijing National Laboratory for Condensed Matter Physics, Institute of Physics, Chinese Academy of Sciences, Beijing 100190, China.

[3]Department of Applied Physics, The Hong Kong Polytechnic University, Hung Hom, Hong Kong SAR, China.

[4]Jiangsu Key Laboratory for Science and Applications of Molecular Ferroelectrics, Southeast University, Nanjing 211189, China.

[5]Hunan Key Laboratory of Two-Dimensional Materials, College of Chemistry and Chemical Engineering, Hunan University, Changsha 410082, China.

[6]State Key Laboratory of Mechanics and Control of Mechanical Structures, and Institute for Frontier Science, Nanjing University of Aeronautics and Astronautics; Nanjing 210016, China.

[7]Department of Physics, University of Texas at Austin, Austin, TX, USA.

[8]National Laboratory of Solid State Microstructures, School of Electronic Science and Engineering and Collaborative Innovation Center of Advanced Microstructures, Nanjing University, Nanjing, China.

[9]School of Electronic Science & Engineering, Southeast University, Nanjing 210096, China.

[10]Jiangsu Key Lab on Opto-Electronic Technology, School of Physics and Technology, Nanjing Normal University, 1 Wenyuan Road, Nanjing 210023, China.

[11]School of Physics and Electronics, Hunan University, Changsha 410082, China.

[#]These authors contributed equally: Zhenliang Hu, Bei Zhao, Qiang Fu

*Corresponding authors: phylhw@njnu.edu.cn; zhni@seu.edu.cn; yuanliuhnu@hnu.edu.cn; phyljp@seu.edu.cn

Table of contents

Supplementary Figure 1 The growth of 2D MoO_3 flakes with different cooling rates.

Supplementary Figure 2 The influence of growth temperature on the thickness of the MoO_3 film.

Supplementary Figure 3 AFM characterization of another 5 different regions at the edge of the MoO_3 film on the mica substrate.

Supplementary Figure 4 AFM characterization of 15 inner regions of MoO_3 film on mica substrate.

Supplementary Figure 5 Eight random locations within the large area of the MoO_3 film region.

Supplementary Figure 6 Raman spectra of MoO_3.

Supplementary Figure 7 Schematic illustration of the water-assisted MoO_3 transfer process.

Supplementary Figure 8 The optical images of transferred CVD-grown MoO_3 on SiO_2/Si substrate.

Supplementary Figure 9 AFM characterization of 7 different regions at the edge of the transferred MoO_3 film.

Supplementary Figure 10 AFM characterization of 12 inner regions of transferred MoO_3 film.

Supplementary Figure 11 XPS and XRD characterization of the grown MoO_3..

Supplementary Figure 12 HAADF-STEM image of MoO_3.

Supplementary Figure 13 The crystal structure of orthorhombic MoO_3.

Supplementary Figure 14 AFM measurement of MoO_3 layer distance.

Supplementary Figure 15 Ultraviolet–visible spectrum of as-grown MoO_3 on mica substrate.

Supplementary Figure 16 Density functional theory (DFT) calculation of the electronic band structure of MoO_3 and the charge density of the Bloch states.

Supplementary Figure 17 DFT simulation of the interlayer binding energies.

Supplementary Figure 18 Characterization of the graphene-MoO_3-FLG device used in the dual gate method for dielectric constant measurement.

Supplementary Figure 19 Capacitances of MoO_3 as a function of voltage and frequency.

Supplementary Figure 20 The electric field at breakdown versus dielectric constant of state-of-the-art dielectrics.

Supplementary Figure 21 The leakage current at different temperatures.

Supplementary Figure 22 The fabrication process of MoS_2 top-gate FET using FLG electrodes.

Supplementary Figure 23 Raman, PL, and AFM characterization of micro-scale MoS_2 FET.

Supplementary Figure 24 EDS mapping of the MoS_2 FET device.

Supplementary Figure 25 Cross-sectional HAADF, bright-field STEM image, and corresponding EDS mapping.

Supplementary Figure 26 Band structure of MoS_2 and MoO_3, and band alignment of MoS_2/MoO_3 heterostructure.

Supplementary Figure 27 The transfer curve of MoS_2 bottom-gate FET using 285 nm SiO_2 as the gate dielectric.

Supplementary Figure 28 Hysteretic behavior and Ohmic contact of the FET device.

Supplementary Figure 29 Transfer characteristics of the other 12 MoS_2 FETs.

Supplementary Figure 30 Hysteresis analysis.

Supplementary Figure 31 Effects of MoO_3 thickness on the device performance.

Supplementary Figure 32 Transfer curves and leakage currents of MoS_2 top-gate devices using MoO_3 dielectric and Au contacts.

Supplementary Figure 33 Transfer curves and leakage currents of MoS_2 bottom-gate devices using MoO_3 dielectric and Au contacts.

Supplementary Figure 34 The gate leakage current at different temperatures of device #9.

Supplementary Figure 35 Heatmaps of the key parameters of the MoS_2 FET array over a 1×1 mm^2 area.

Supplementary Figure 36 Statistical analysis of SS and on/off ratio of the device array.

Supplementary Figure 37 Statistical analysis of the field-effect mobility of the device array.

Supplementary Figure 38 Output characteristics of p-type micro-scale WSe_2 FETs that used FLG as the source and drain contacts.

Supplementary Figure 39 Transfer characteristics of fabricated micro-scale WSe_2 FETs.

Supplementary Figure 40 Performance of the CMOS inverter.

Supplementary Figure 41 The static power consumption of the CMOS inverter as a function of input voltage at different supply voltages.

Supplementary Figure 42 The transfer curve result, voltage gain, and static power consumption as a function of input voltage of another CMOS inverter.

Supplementary Figure 43 The effect of the fast-cooling methods on the increase in WSe_2-domain size.

Supplementary Figure 44 Breakdown characterization of MoO_3.

Supplementary Figure 45 Lifetime prediction.

Supplementary Figure 46 Operational stability test.

Supplementary Figure 47 Stability test of the MoO_3 device in ambient conditions.

Supplementary Figure 48 Estimation of the interfacial trap density of a bottom gate device using the *C*-*V* conductance method.

Supplementary Figure 49 Estimation of interfacial trap density of a top gate device using the charge pumping method.

Supplementary Figure 50 Estimation of interfacial trap density of a top gate device using the $1/f$ method.

Supplementary Figure 51 AFM, Raman, and PL characterization of the LED device.

Supplementary Figure 52 Performance of the WS_2 transient LED using the MoO_3 dielectric.

Supplementary Table 1 The reported MoO_3 dielectric constant.

Supplementary Note 1 Fast-cooling method

Supplementary Note 2 Estimation of the dielectric constant in *C*-*V* measurement.

Supplementary Note 3 Estimation of the electric field at breakdown.

Supplementary Note 4 Reliability test.

Supplementary Note 5 Estimation of the interfacial trap density.

Supplementary Note 6 Potential of MoO_3 in vdW light-emitting diodes.

Supplementary References

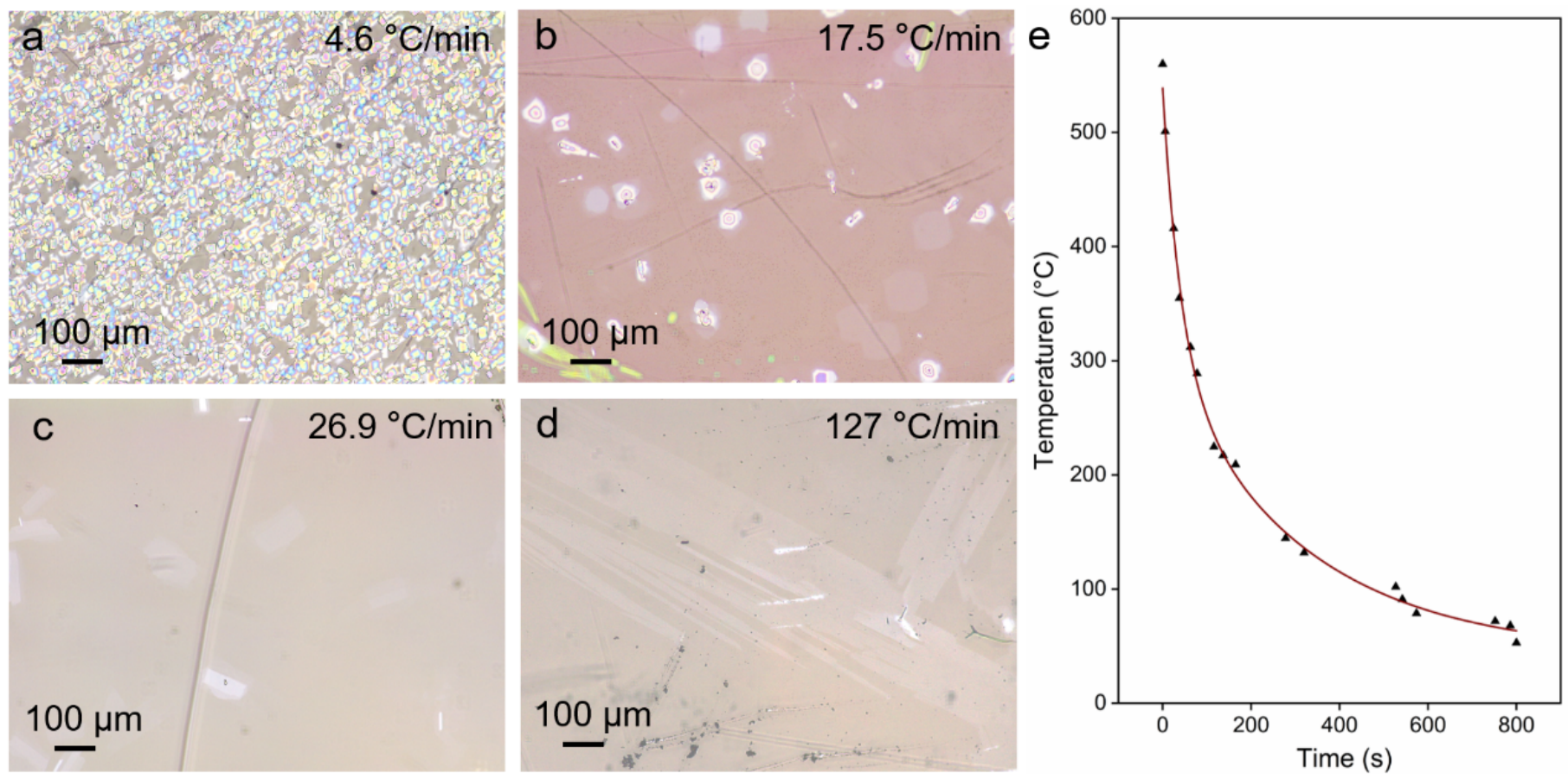

Supplementary Figure 1 The growth of 2D MoO_3 flakes with different cooling rates. **a-d**, The optical images of 2D MoO_3 flakes on a mica substrate with different cooling rates. **e,** Temperature-time diagram in the rapid cooling step.

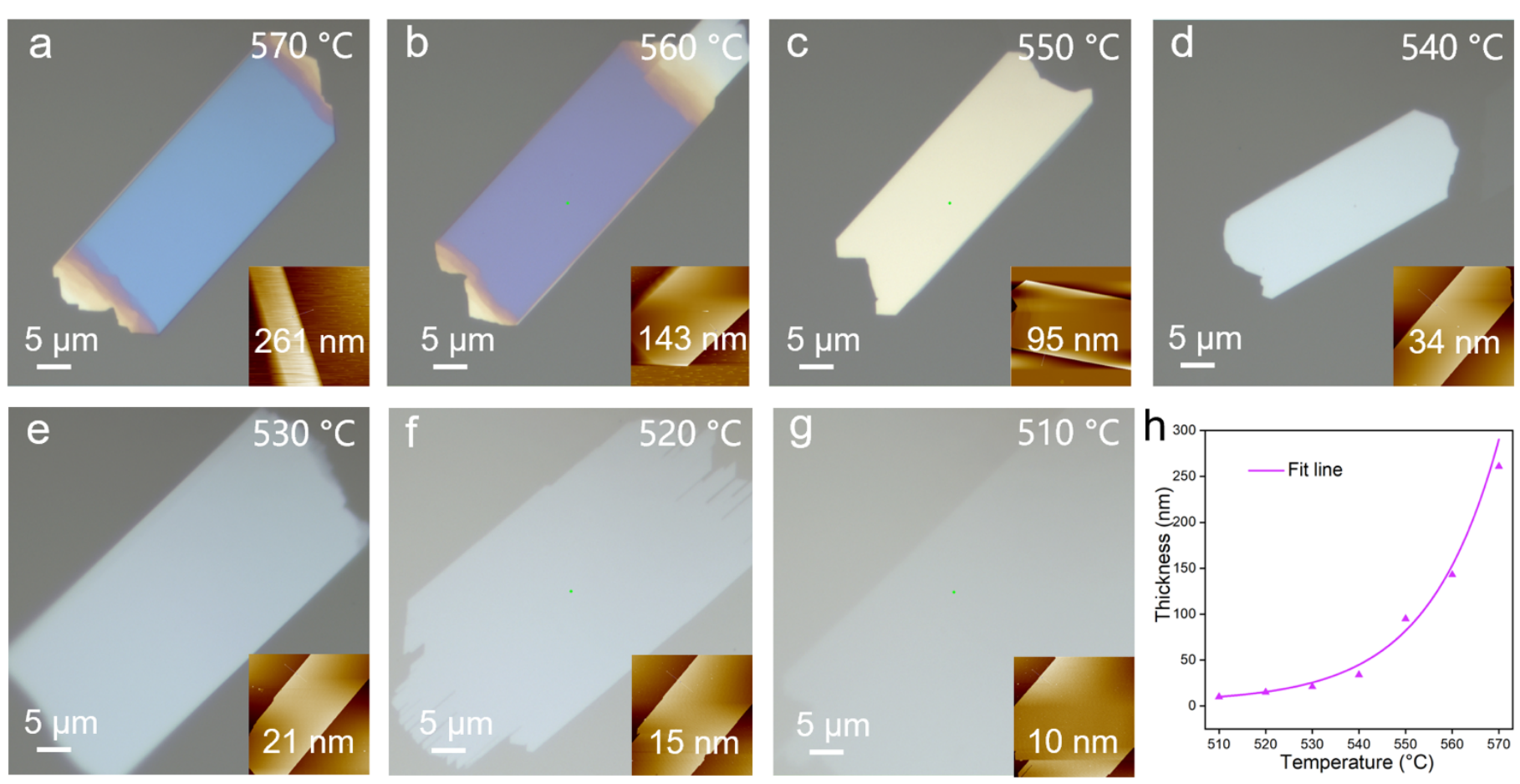

Supplementary Figure 2 The influence of growth temperature on the thickness of the MoO_3 film. **a-g**, The optical images of MoO_3 flakes with varying growth temperatures from 510℃-570℃, the inset is the corresponding AFM images. **h**, The thickness variations of MoO_3 at different holding temperatures corresponding to **a-g**.

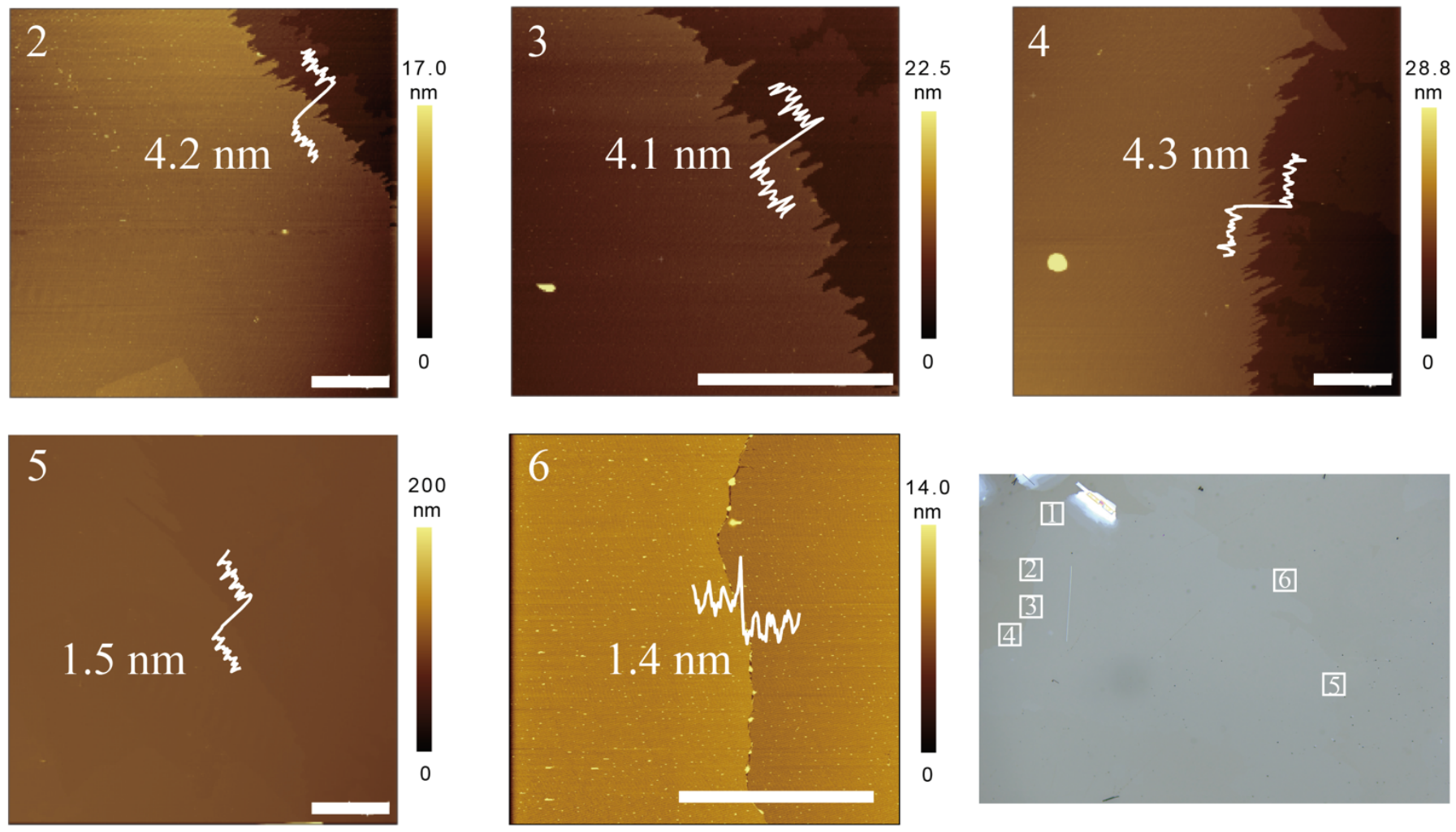

Supplementary Figure 3 AFM characterization of another 5 different regions at the edge of the MoO_3 film on the mica substrate, those regions are labeled in the optical images. Scale bars are 10 μm. Noted that AFM characterization of position 1 is presented in Fig. 1d.

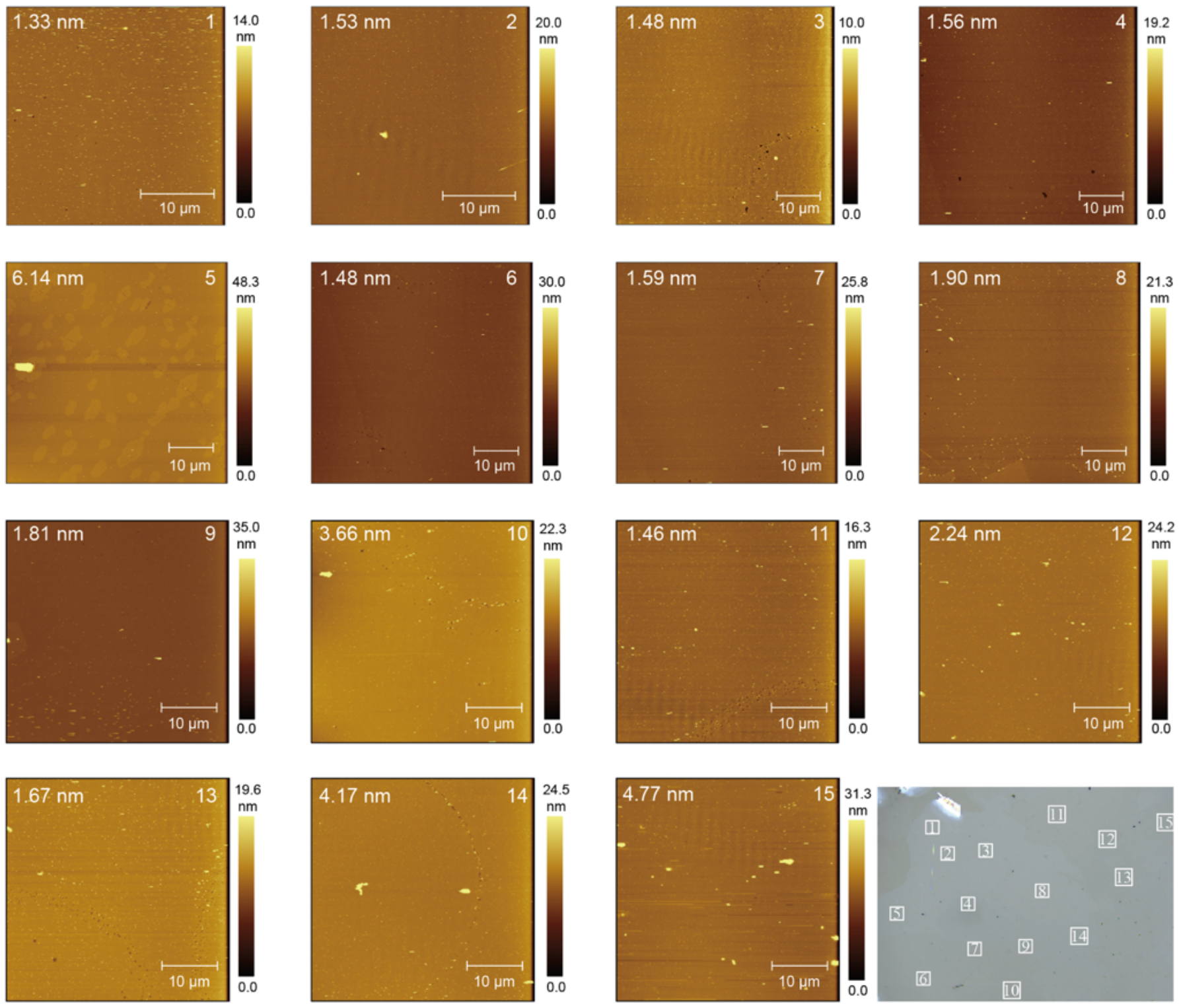

Supplementary Figure 4 AFM characterization of 15 inner regions of MoO_3 film on mica substrate, the place of each region is labeled in the optical image. The RMS roughness of each region is shown in the top left corner. Scale bars are 10 µm. The extracted RMS roughness of most regions ranges from 1-2 nm in a scan area of 40 µm × 40 µm. In regions containing dust or surface contaminants, the RMS roughness becomes significantly larger, as these extrinsic features introduce substantial local height fluctuations and therefore increase the measured overall roughness.

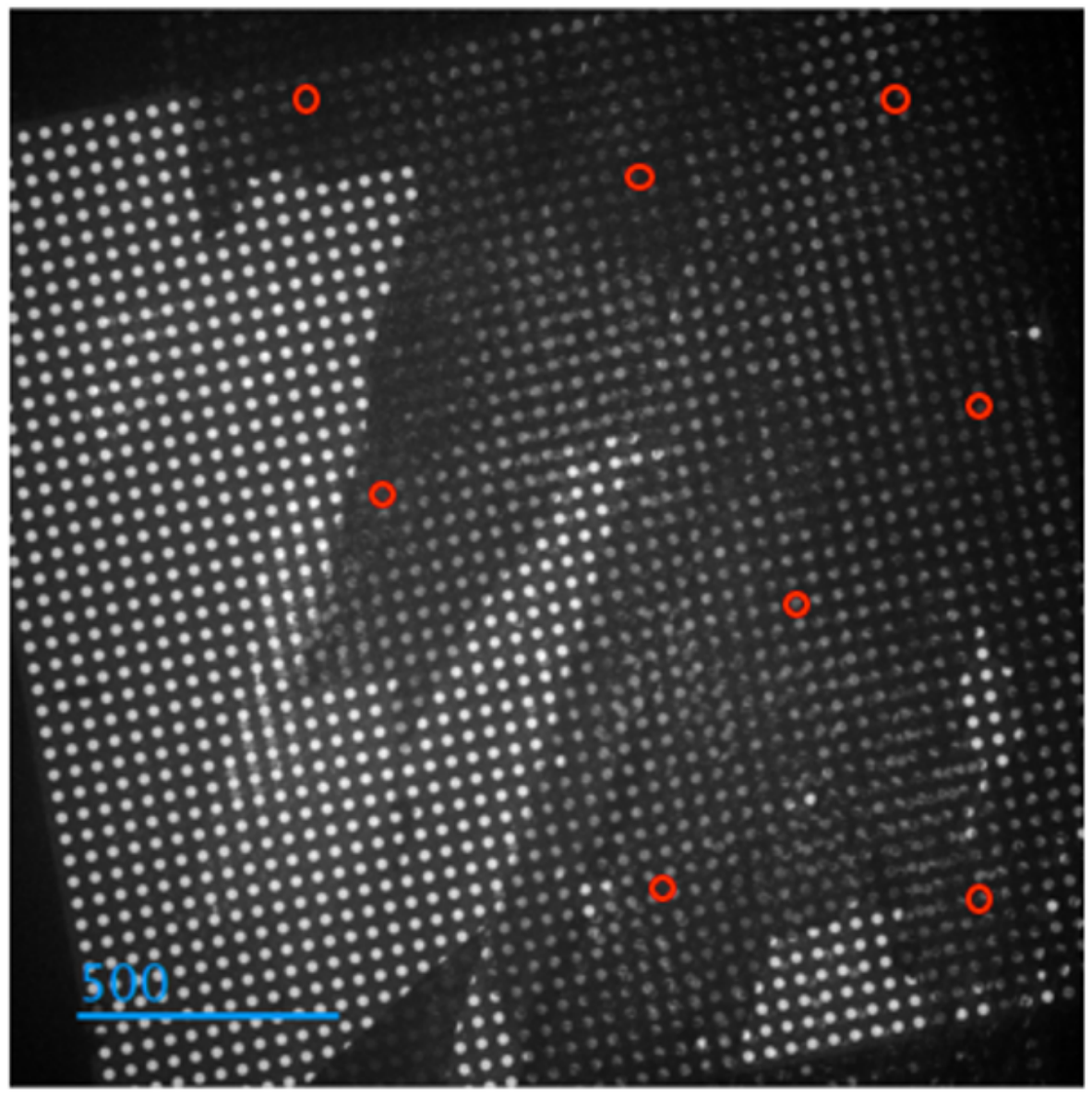

Supplementary Figure 5 Eight random locations within the large area MoO_3 film region, scale bar is 500 nm.

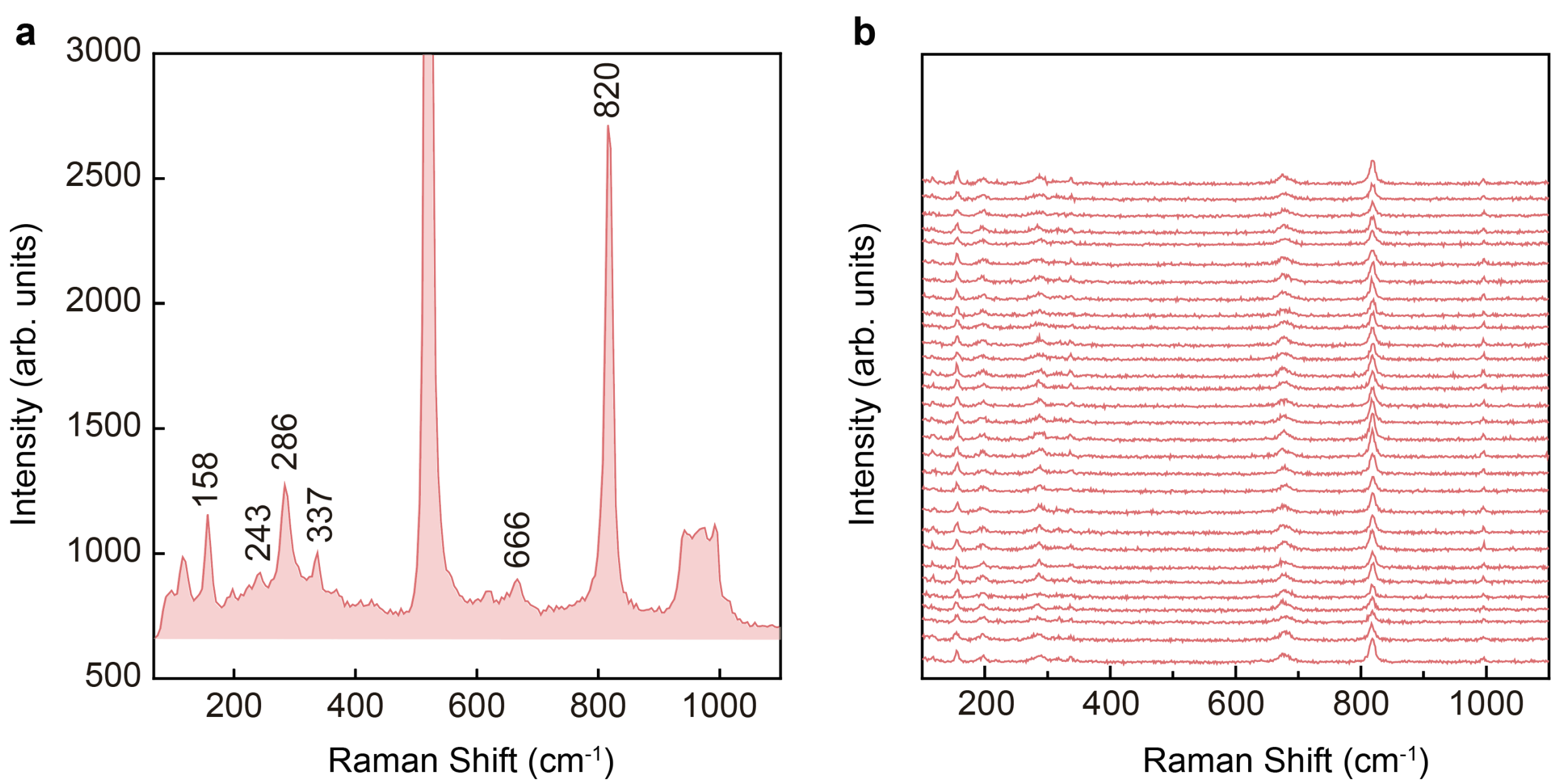

Supplementary Figure 6 Raman spectra of MoO_3. **a,** Raman spectrum of MoO_3 after transfer to the SiO_2 substrate. Raman shifts of the dominant peaks are labeled. Peaks at approximately 521 and 950 cm^{-1} are from the substrate. **b,** Representative Raman spectra at 30 different locations on the large-area MoO_3 film.

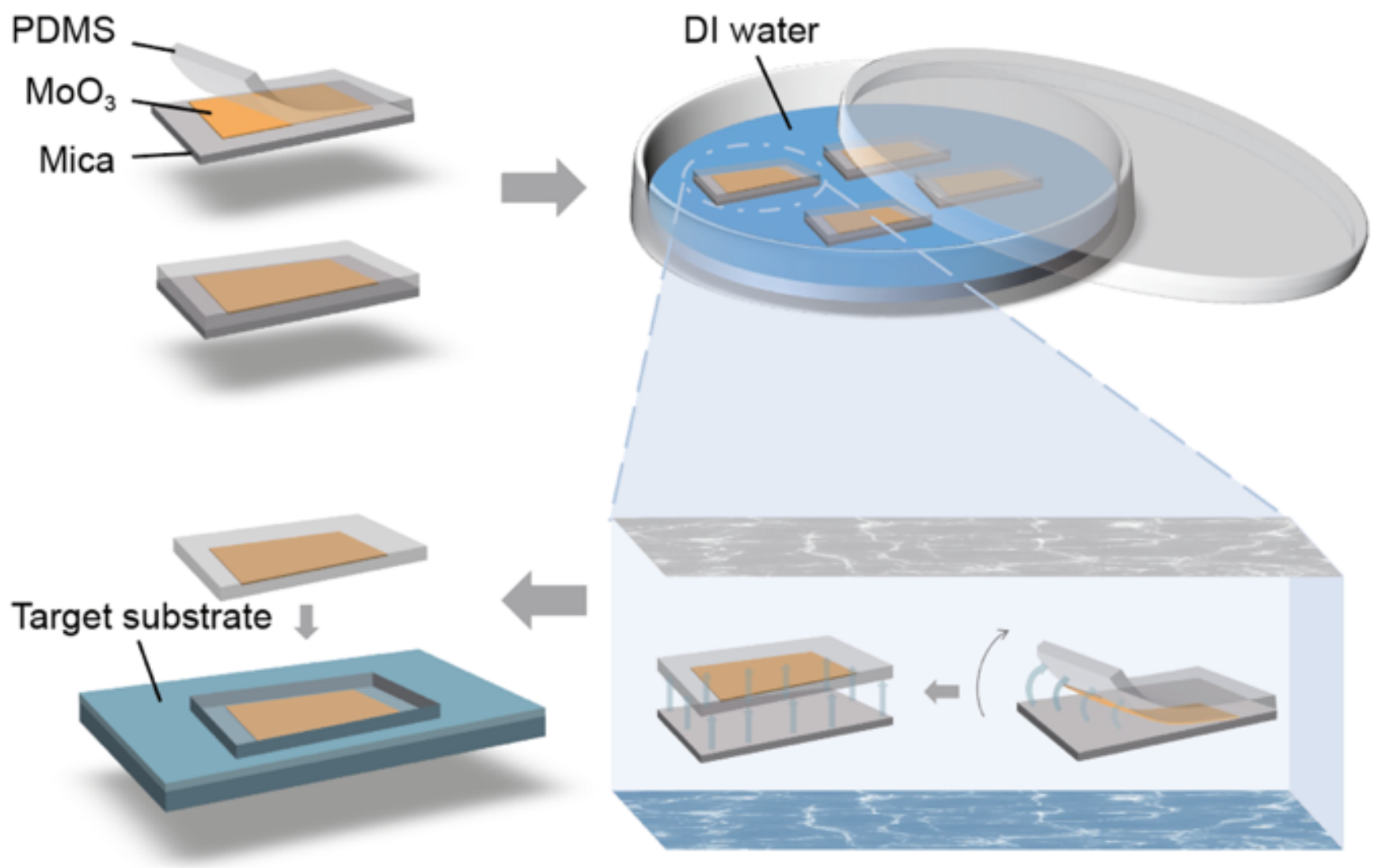

Supplementary Figure 7 Schematic illustration of the water-assisted MoO_3 transfer process from the mica substrate to the target substrate.

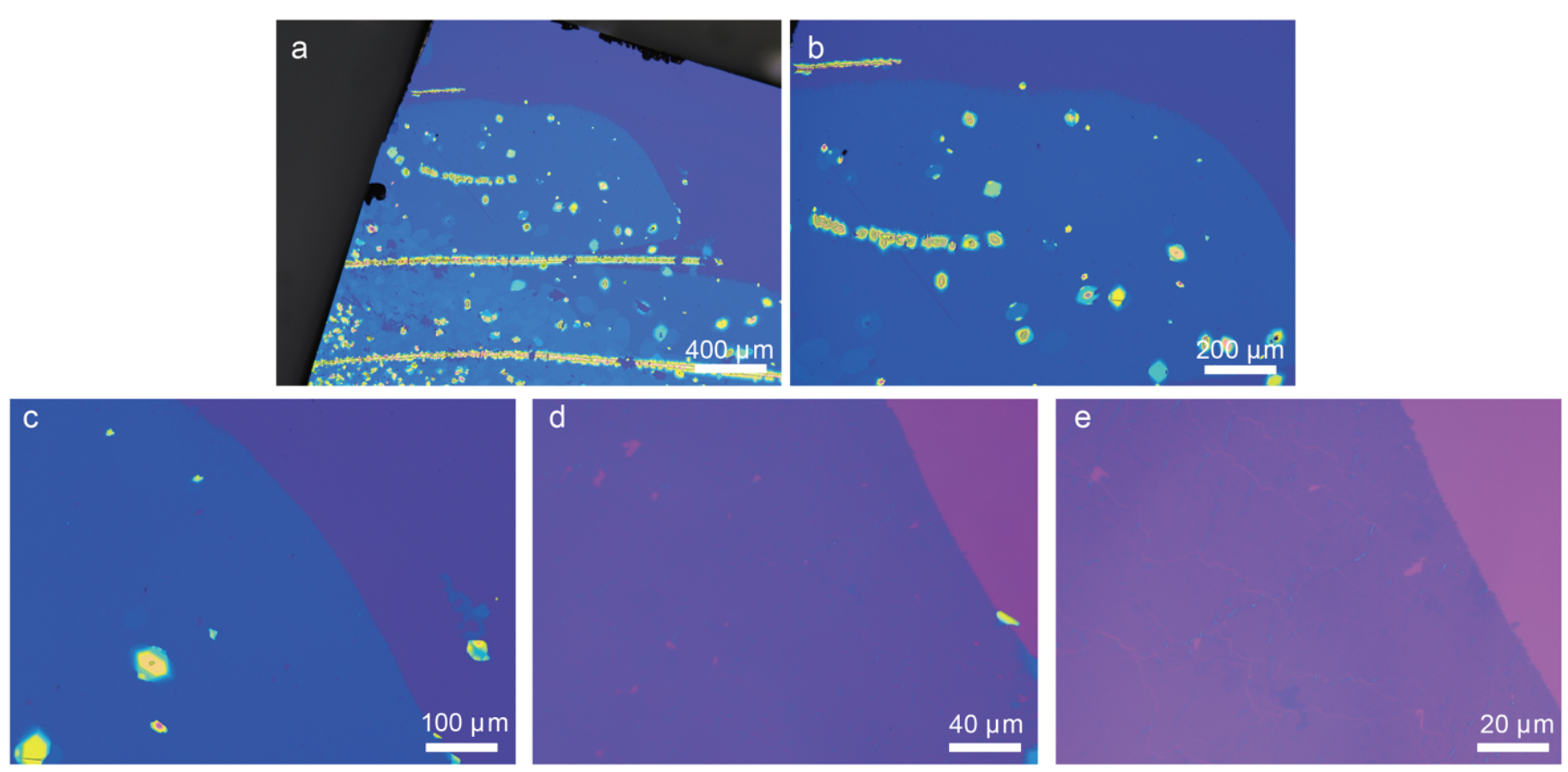

Supplementary Figure 8 The optical images of transferred CVD-grown MoO_3 on SiO_2/Si substrate. The size of MoO_3 is approximately 1.6 mm.

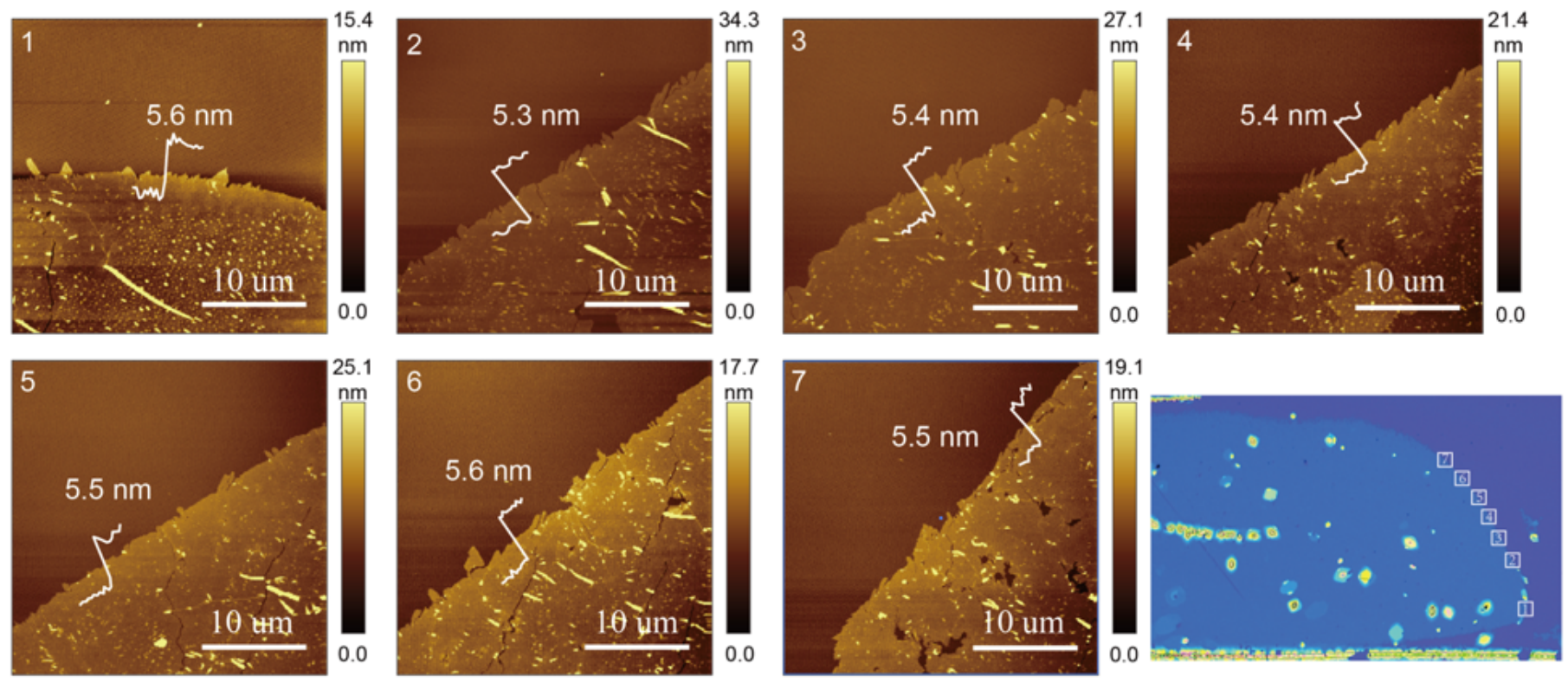

Supplementary Figure 9 AFM characterization of 7 different regions at the edge of the transferred MoO_3 film, which are labeled in the optical images. The size of each region is approximately 30 μm.

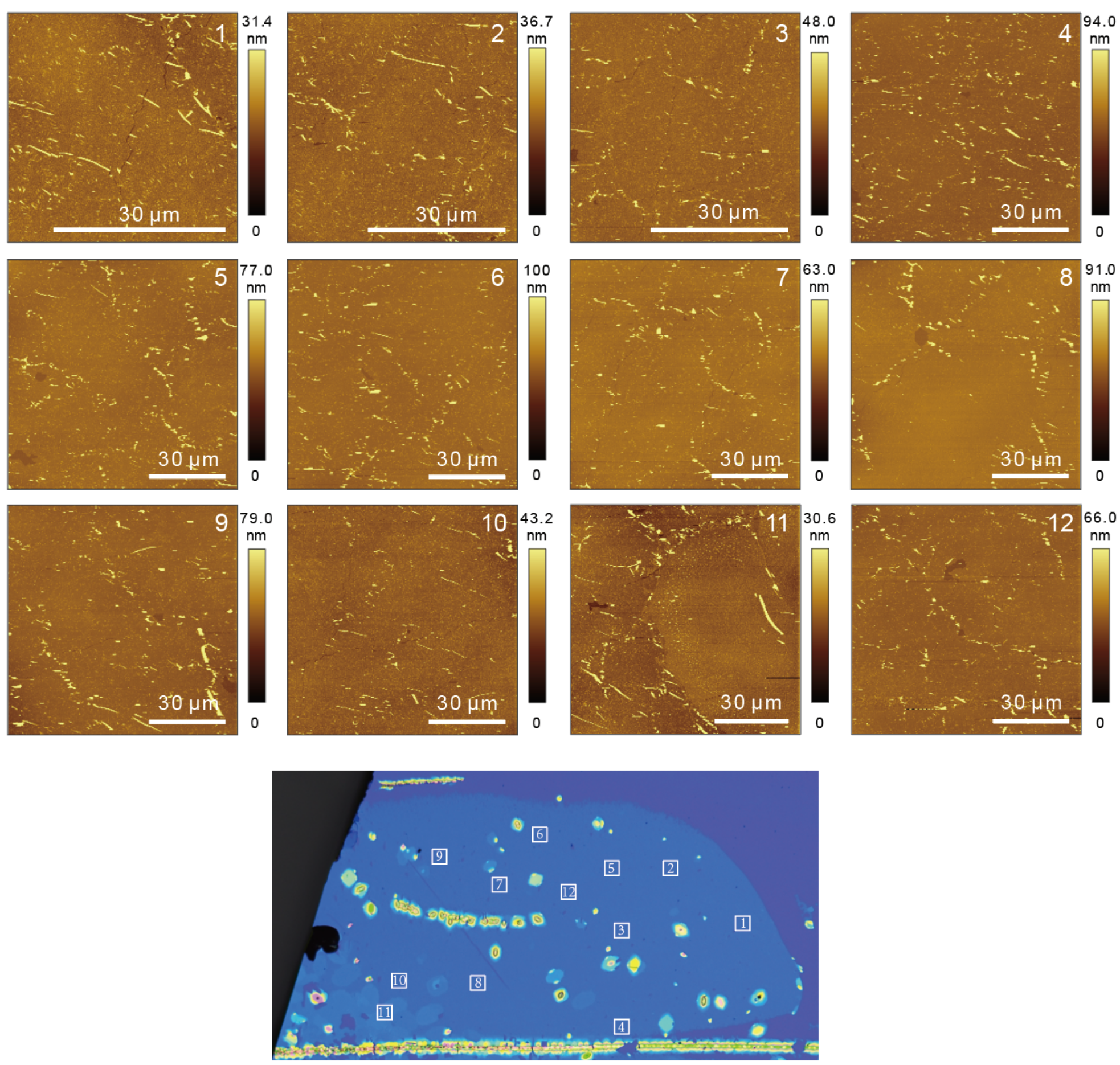

Supplementary Figure 10 AFM characterization of 12 inner regions of the transferred MoO_3 film, the place of each region is labeled in the optical image.

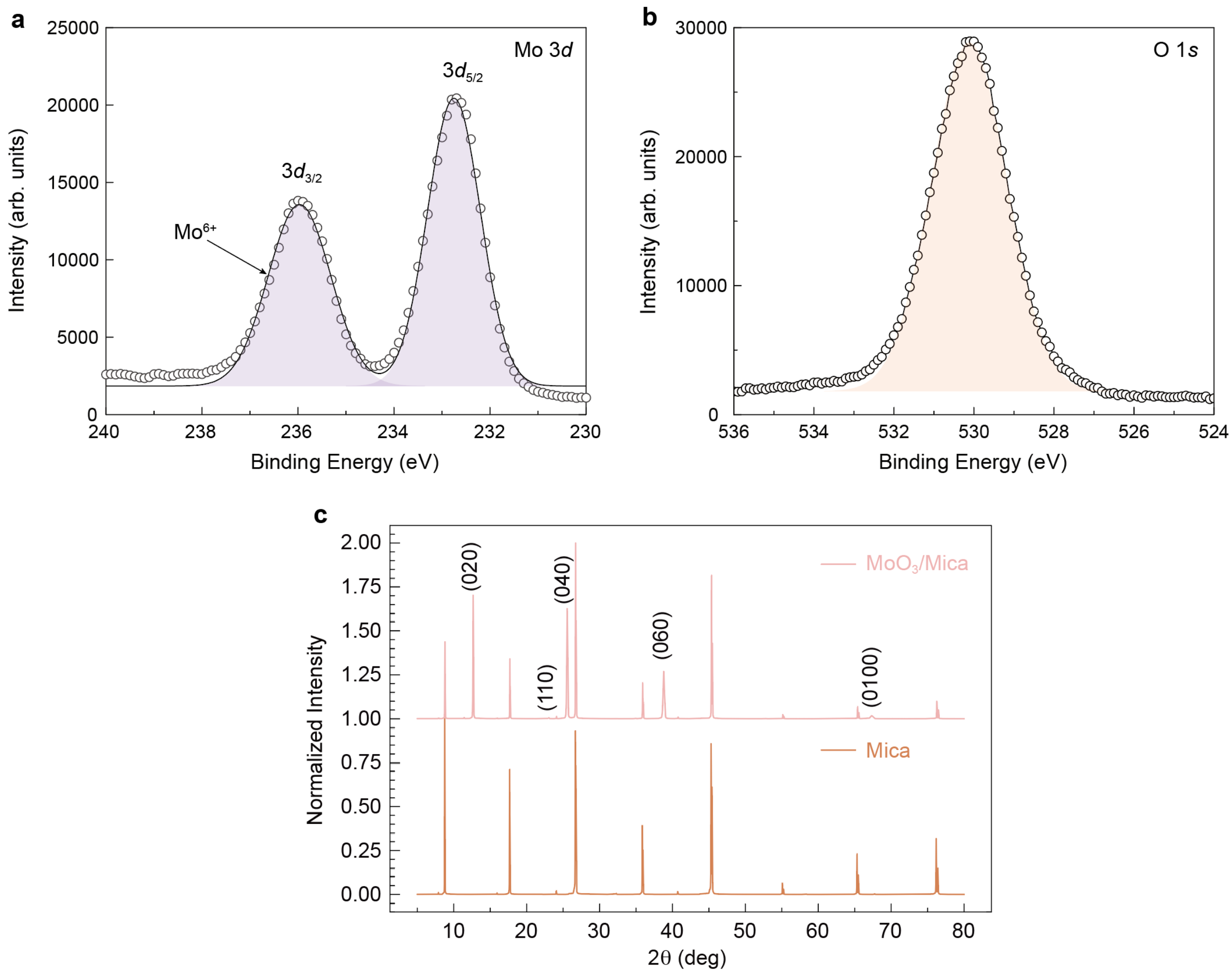

Supplementary Figure 11 XPS and XRD characterization of the grown MoO_3. **a,** the XPS spectra of Mo 3*d*. **b,** the XPS spectra of O 1*s*. The Mo 3*d* spectrum exhibits two characteristic peaks located at 232.75 eV and 236.0 eV, which are assigned to Mo $3d_{5/2}$ and $3d_{3/2}$ of Mo 3*d*, respectively[1]. Meanwhile, the spectrum of O 1*s* is dominated by a single peak at ～530.1 eV, corresponding to lattice oxygen, with almost no obvious peak at ~531.6 eV that is typically associated with defective site oxygen[2]. The XPS results confirm that the as-grown film is stoichiometric MoO_3 with a low density of oxygen vacancies. **c,** The XRD peaks of MoO_3 match well with the orthorhombic MoO_3 XRD peaks, indicating the single crystallinity and orthorhombic phase of the CVD-grown MoO_3. In addition, only the (0, k, 0) peaks showed strong intensities, suggesting an anisotropic growth orientation during CVD growth.

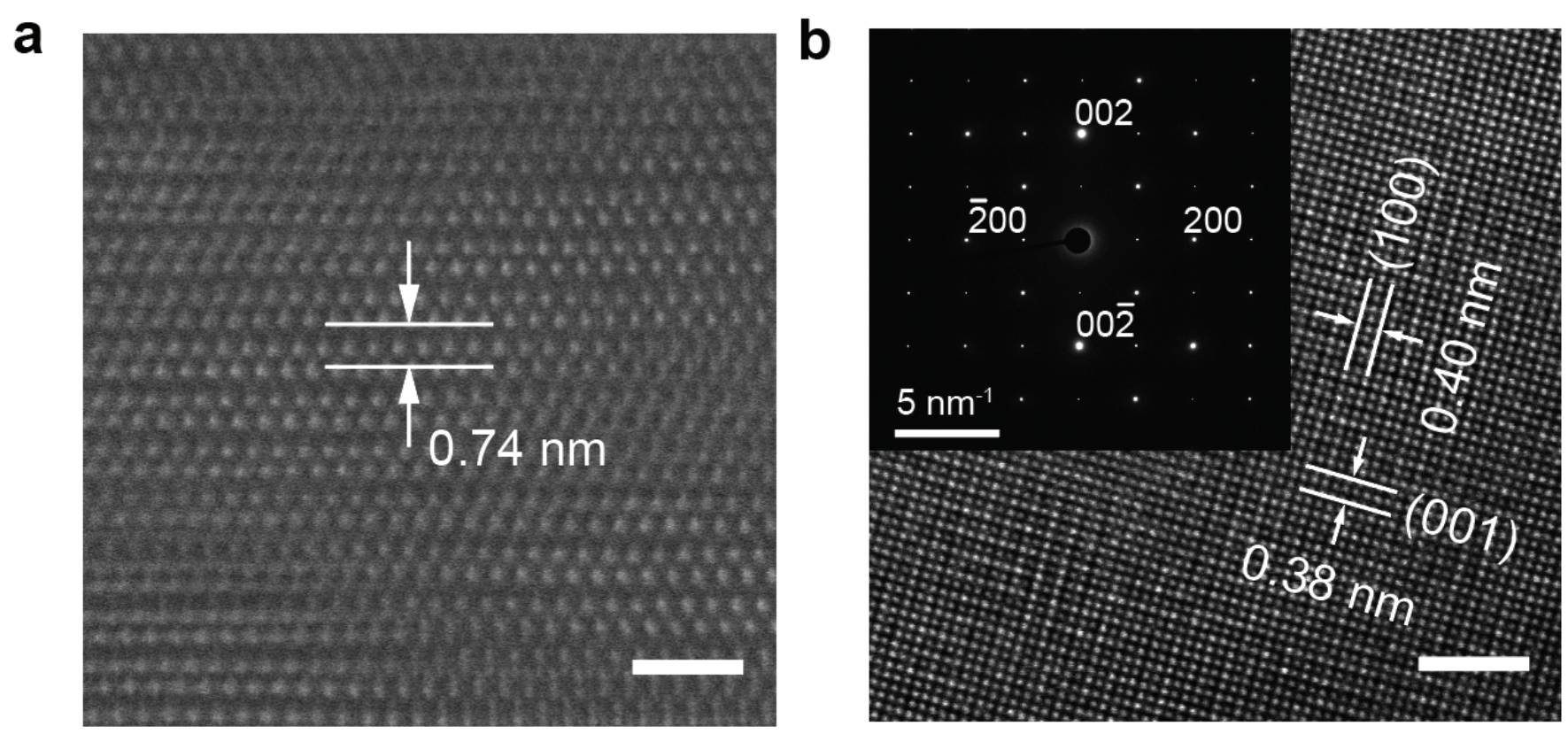

Supplementary Figure 12 HAADF STEM image of MoO_3. **a,** Cross-sectional HAADF STEM image of MoO_3. Scale bar, 2 nm. **b,** Plane-view HAADF STEM image of MoO_3. Scale bar, 2 nm. Inset is the SAED pattern of MoO_3.

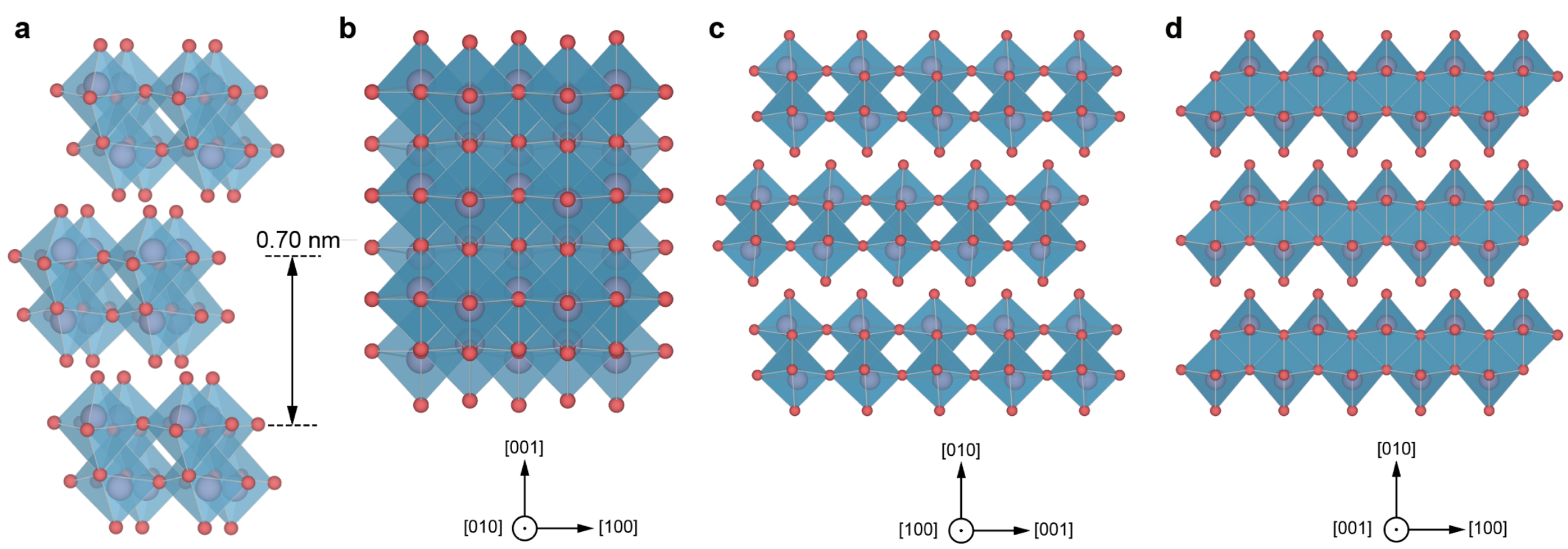

Supplementary Figure 13 The crystal structure of orthorhombic MoO_3. **a**, Lattice polyhedral structure and layer distance of the orthorhombic MoO_3. **b-d,** Polyhedral structure viewed from the [010], [100], and [001] directions. Oxygen and molybdenum atoms are represented in red and cyan, respectively.

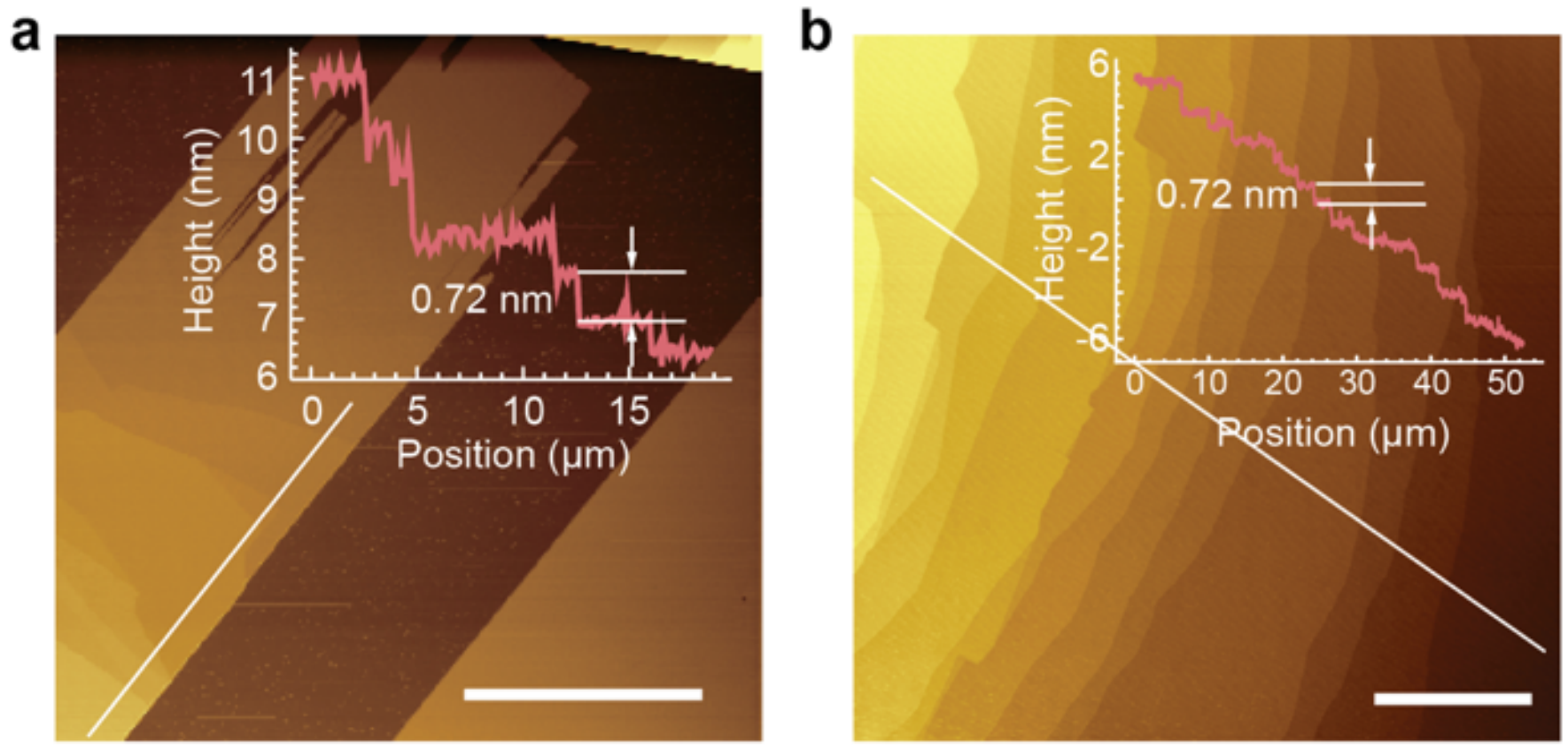

Supplementary Figure 14 AFM images of MoO_3 flakes on mica substrates, exhibiting the layered structure. The height profile of the white line is plotted. Scale bars are 10 µm.

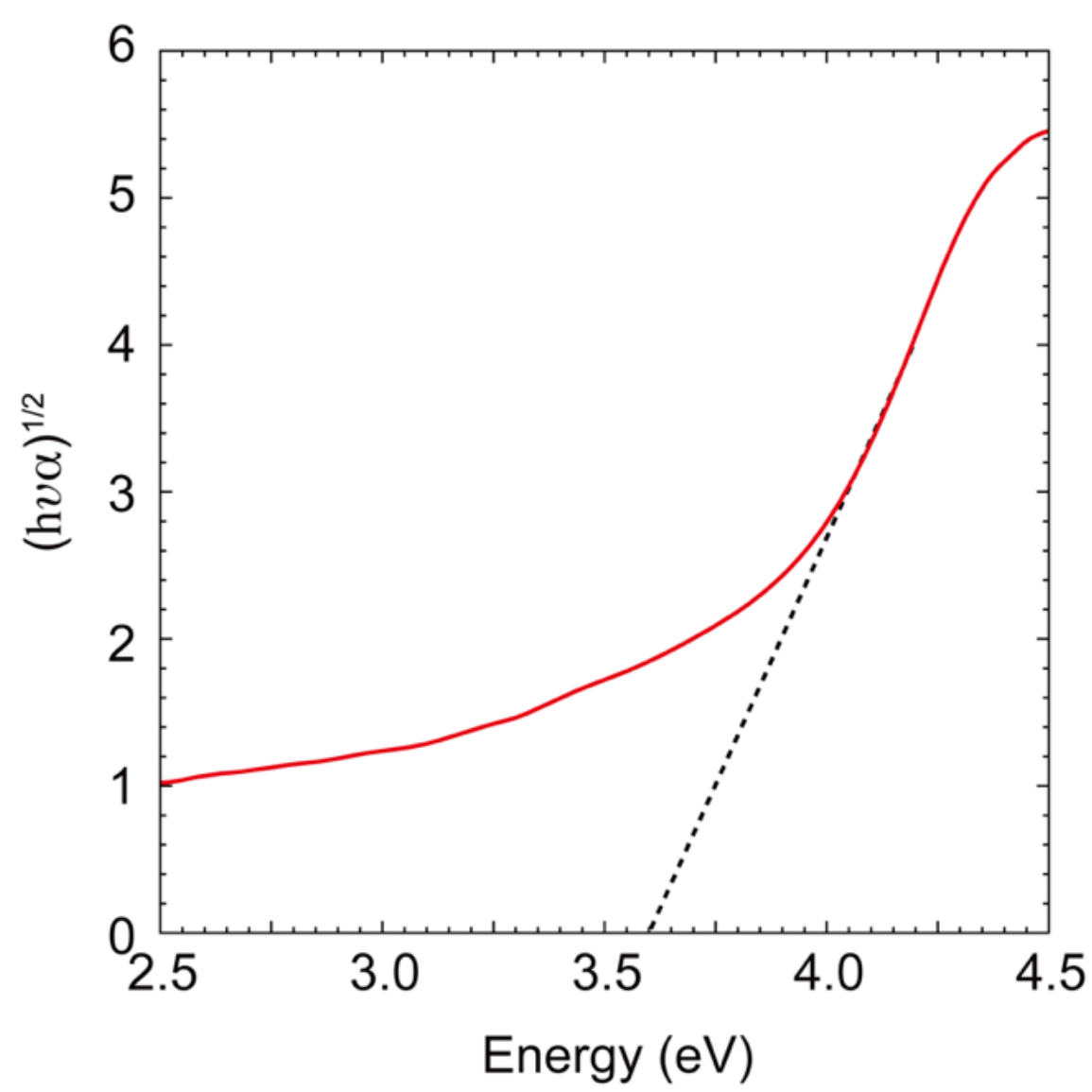

Supplementary Figure 15 Ultraviolet–visible spectrum of MoO_3 on mica substrate. The dashed line is tangent to the point of inflection, and the value at the point of intersection of the tangent line and the x-axis is the bandgap size.

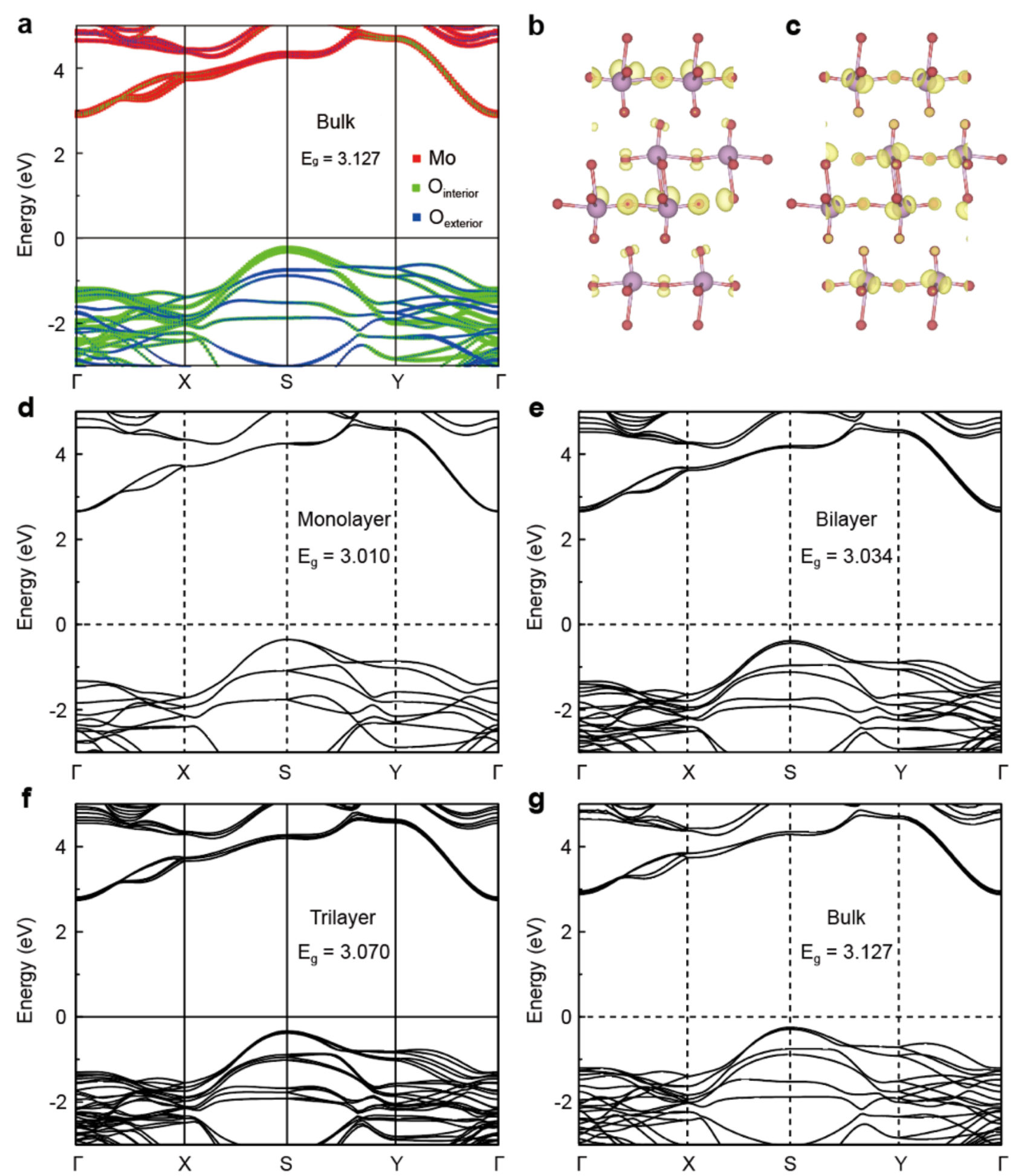

Supplementary Figure 16 Density functional theory (DFT) calculation of the electronic band structure of MoO_3 and the charge density of the Bloch states. **a,** Electronic band structure of bulk MoO_3. The valence band maximum (VBM) and conduction band minimum (CBM) mainly originate from the interior O atoms and Mo atoms, respectively. **b-c,** The charge density of the Bloch states of bulk MoO_3 at the (**b**) VBM and (**c**) CBM. The iso-value is set to 0.008 $e/\text{Å}^3$. The visualized charge densities show that the band edge states are mainly confined in the interior region of each layer. As a result, the electronic structure of MoO_3 has little dependence on the layer number. **d-g,** DFT calculated electronic band structures of (**d**) monolayer, (**e**) bilayer, (**f**) trilayer, and (**g**) bulk MoO_3. The small difference in bandgaps of monolayer, bilayer, trilayer, and bulk MoO_3 suggests the small dependence of the electronic structures on the MoO_3 layer number.

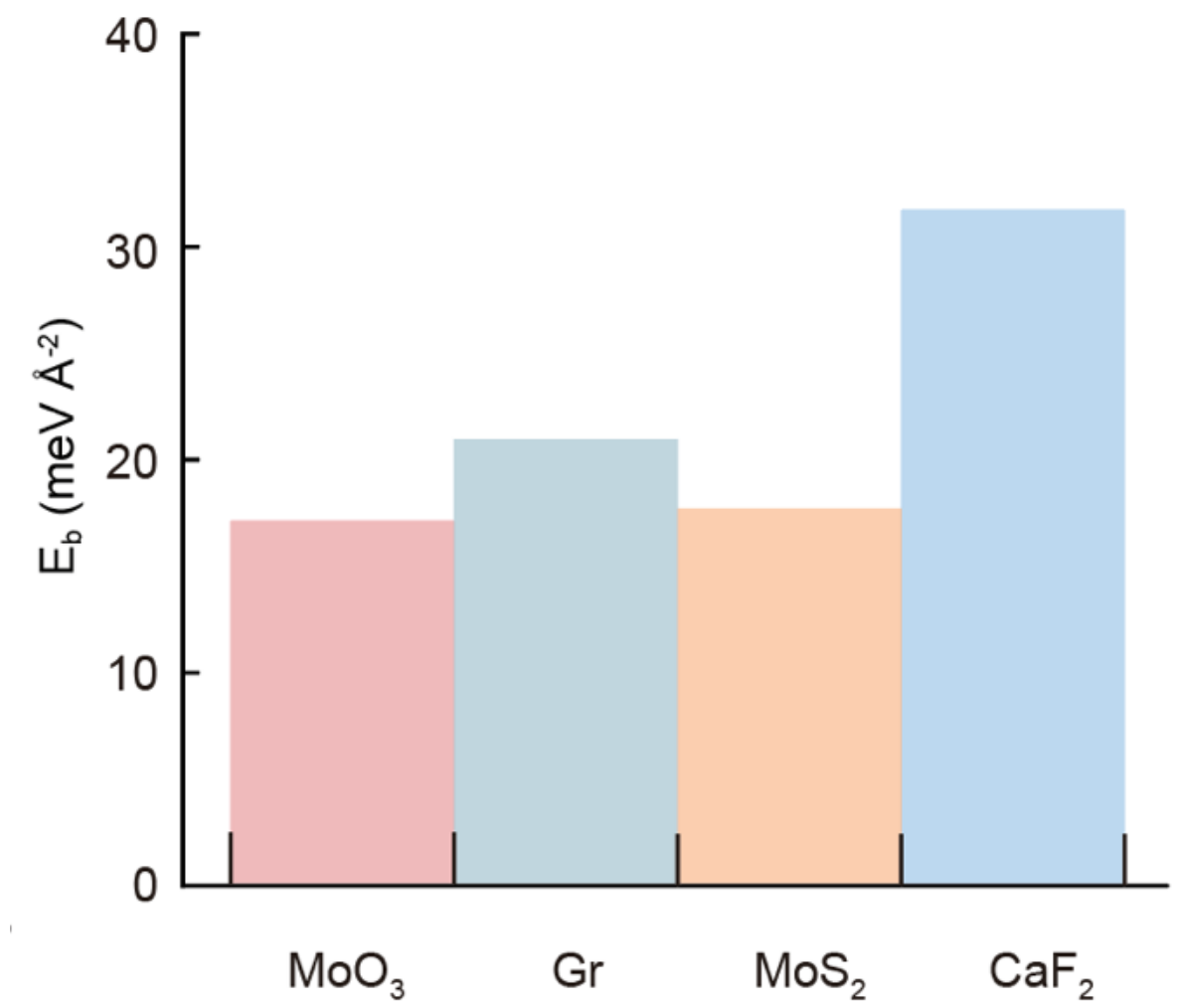

Supplementary Figure 17 DFT simulation of the interlayer binding energies of MoO_3, Graphene, MoS_2, and CaF_2.

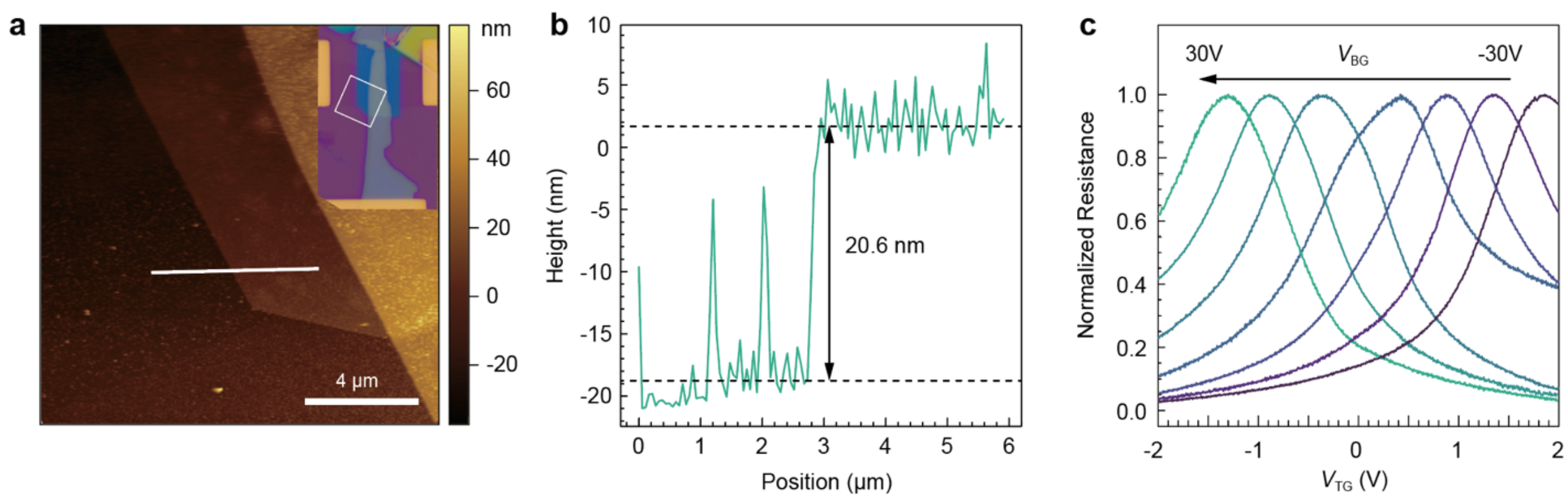

Supplementary Figure 18 AFM characterization of the graphene-MoO_3-FLG device used in the dual gate method for dielectric constant measurement. **a,** AFM images of the squared area in the inset. **b,** the corresponding height profile of the white line in **(a)**; the thickness of the MoO_3 flake is approximately 20.6 nm. **c**, Top-gate transfer curve of graphene FETs using MoO_3 dielectric shift with bottom-gate voltage V_{BG}.

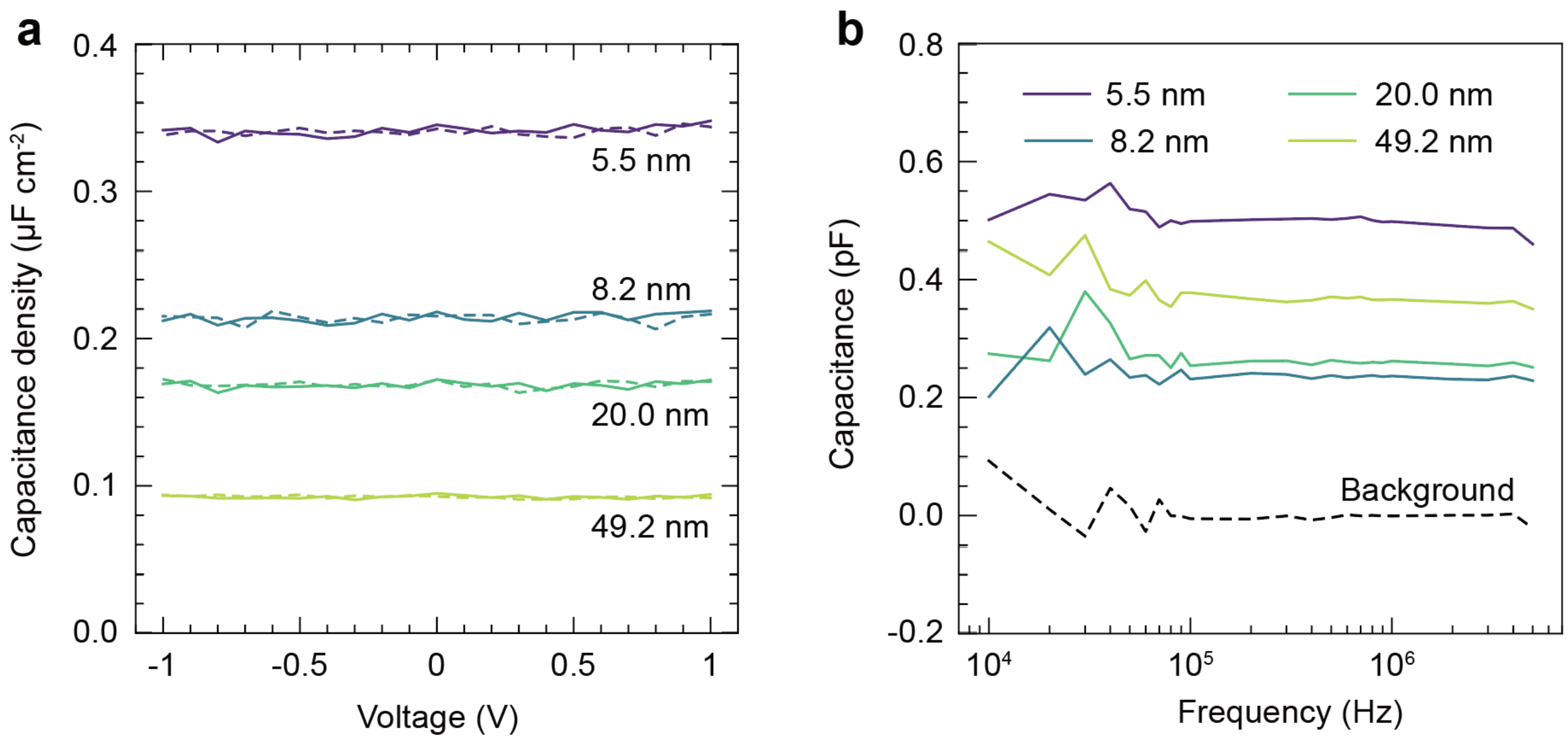

Supplementary Figure 19 Capacitances of MoO_3 as a function of voltage and frequency. **a,** Forward and backward sweep of voltage-dependent capacitance density for MoO_3 flakes of four different thicknesses at 500 kHz. The capacitances have almost no change with the applied voltages. In addition, there are no obvious differences between the forward and backward sweeps, indicating the low density of interface states. **b,** Frequency-dependent capacitance for MoO_3 flakes of four different thicknesses at 1 V. To demonstrate the validity of the C-V characterization, the background of the C-V test is also plotted. The background noise of the C-V characteristic was less than 20 fF, indicating the validity of the measurements. Noted that the capacitance has relatively larger fluctuation, which could be the result of interfacial states caused by the defects.

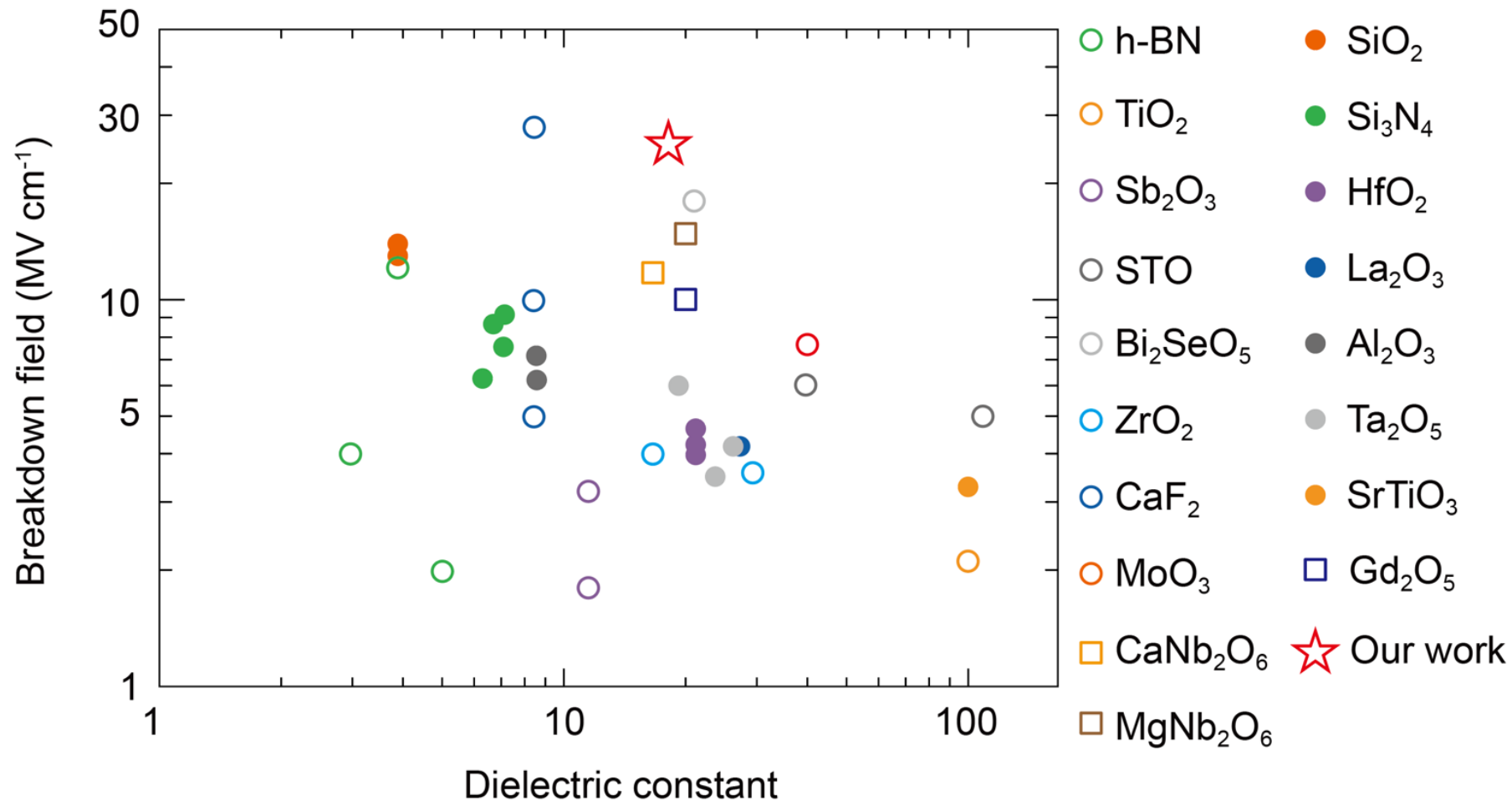

Supplementary Figure 20 The electric field at breakdown versus dielectric constant of state-of-the-art dielectrics**.** For convenience, vdW (quasi-vdW) and non-vdW dielectrics are labeled in hollow circles and solid circles, respectively. As compared to the vdW dielectric h-BN, MoO_3 has better performance in terms of either dielectric constant or electric field at breakdown[3–7].

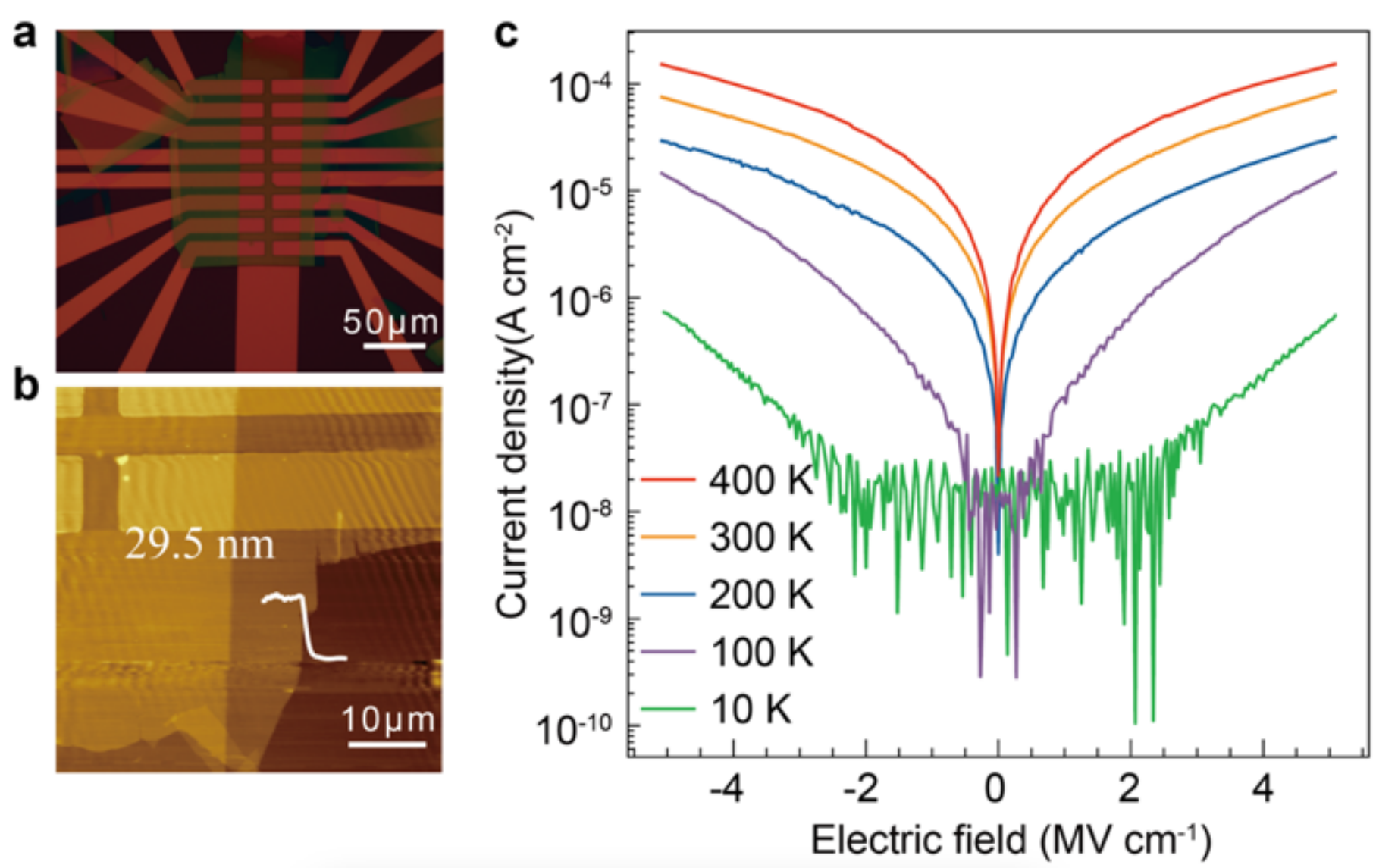

Supplementary Figure 21 The leakage current at different temperatures. **a, b,** Optical and AFM images of Au-MoO3-Au device. **c**, *I-V* characteristic of ~29.5 nm MoO_3 at different temperatures.

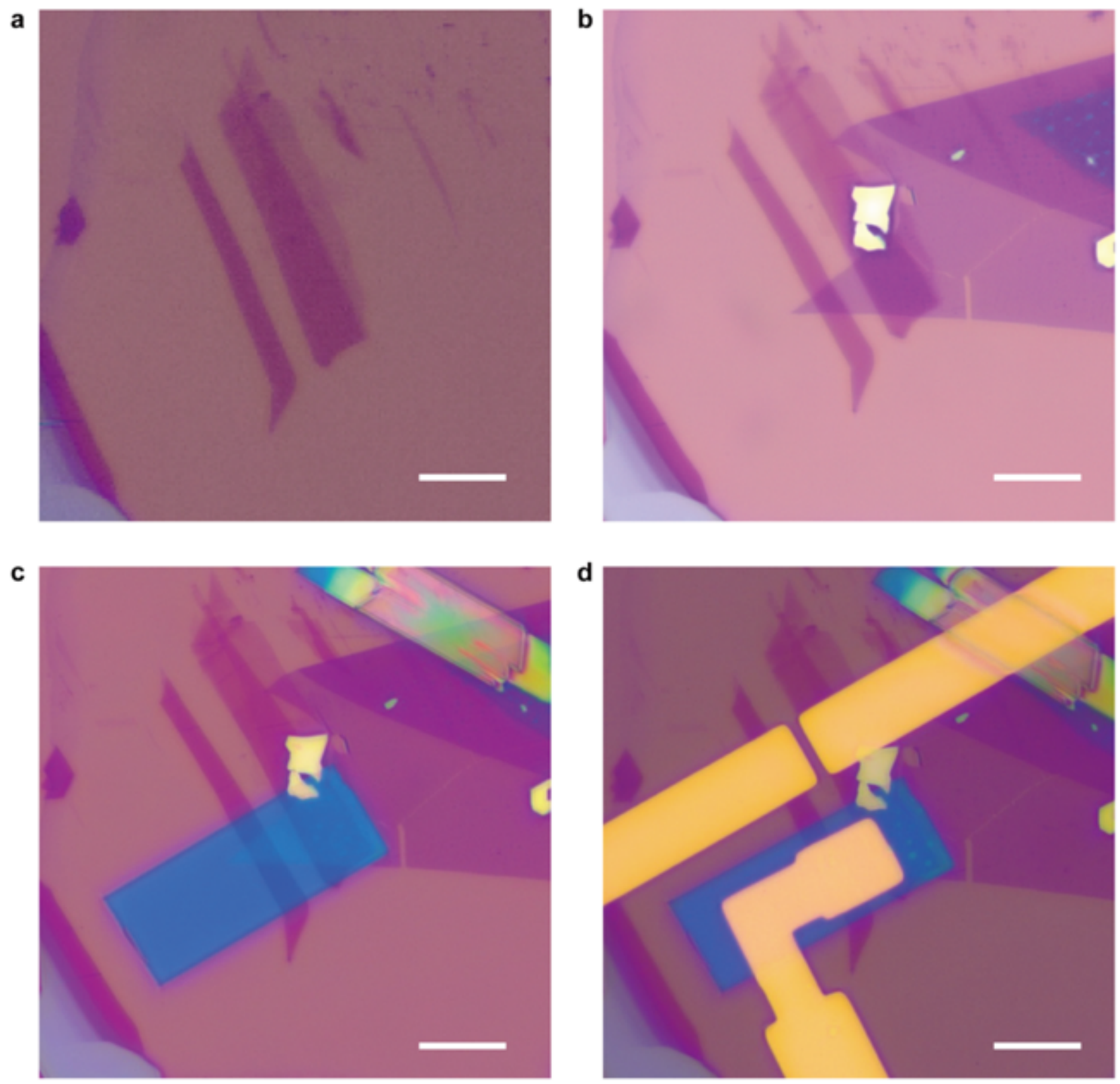

Supplementary Figure 22 The fabrication process of MoS_2 top-gate FET using graphene electrodes. **a,** The exfoliated graphene bottom electrodes on SiO_2/Si substrate. We could get such parallel electrodes via mechanical exfoliation directly. **b,** Graphene/MoS_2 stack after dry transfer of MoS_2 monolayer. **C,** Graphene/MoS_2/MoO_3 stack after transferring MoO_3 flake. **d,** Completion of the device after transferring the gold electrodes. Scale bars, 10 μm.

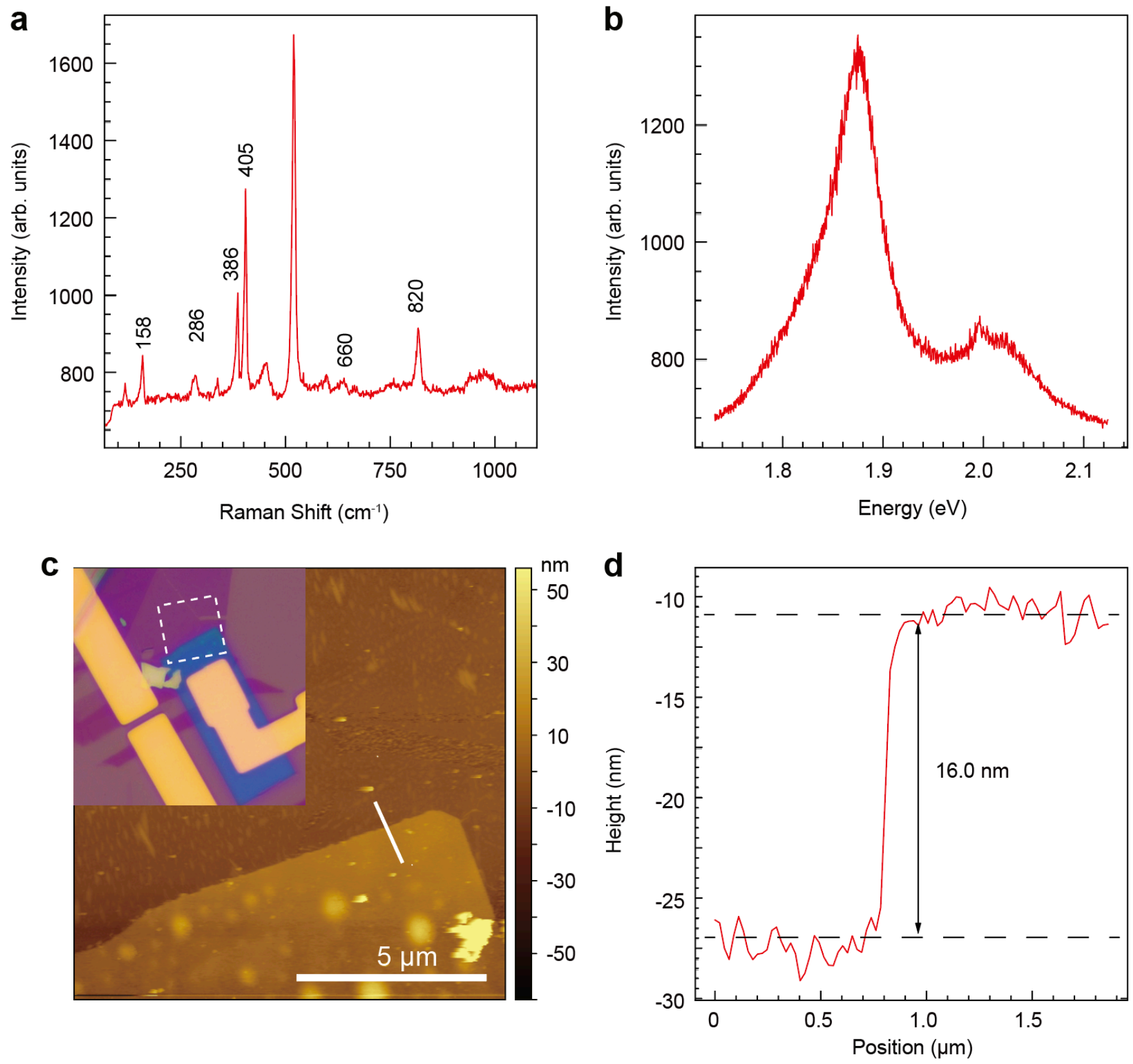

Supplementary Figure 23 Raman, PL, and AFM characterization of MoS_2 FET. **a,** Raman spectrum of the FET device, the signal was taken at the region of Graphene/MoS_2/MoO_3 heterostructure. Raman peaks at 158 cm^{-1}, 286 cm^{-1}, 660 cm^{-1} and 820 cm^{-1} are from MoO_3 flake, peaks at 386 cm^{-1} and 405 cm^{-1} are from MoS_2. **b,** PL spectrum of MoS_2. Two peaks contribute to the A exciton and B exciton. **c,** AFM image of the square area labeled in the inset. **d,** The corresponding height profile of the white line in **c**, the thickness of MoO_3 flake is approximately 16.0 nm.

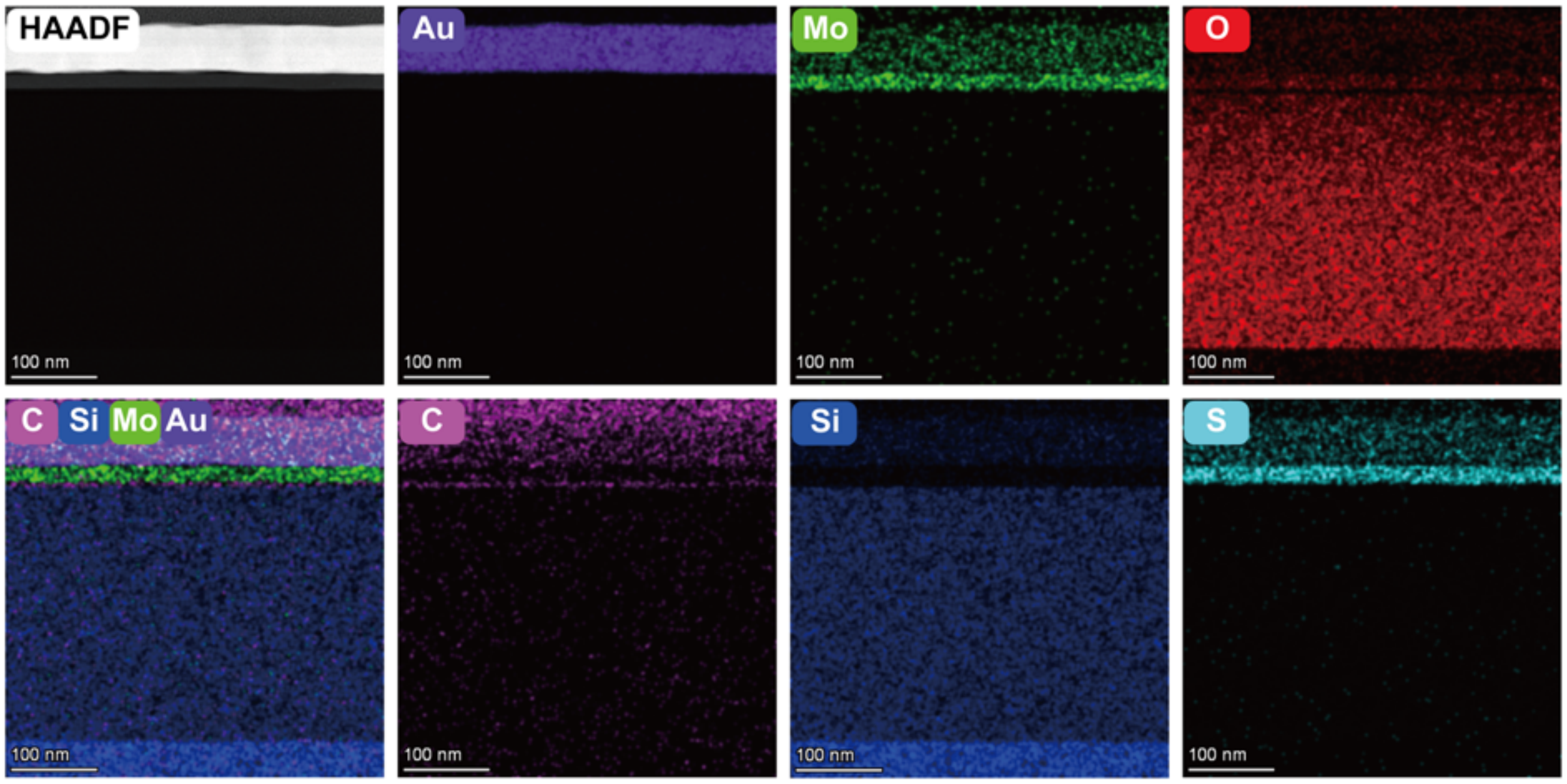

Supplementary Figure 24 Cross-sectional HAADF STEM image and corresponding EDS mapping of Au, Mo, C, Si, S, and O elements.

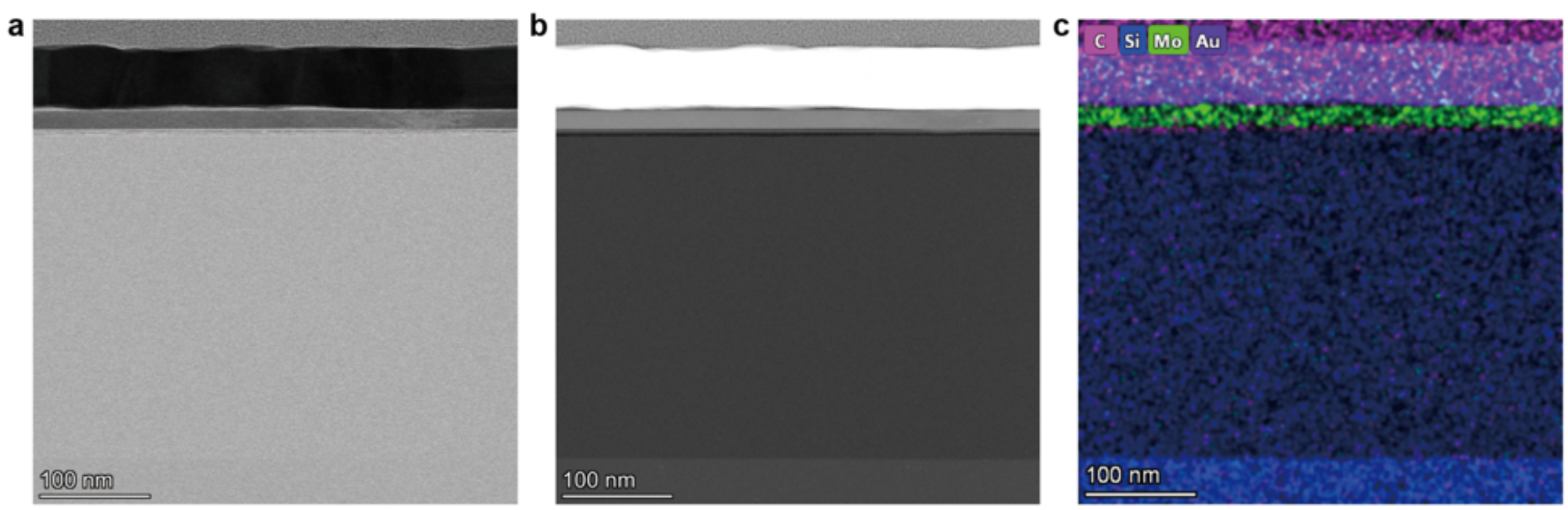

Supplementary Figure 25 STEM results. Cross-sectional (**a**) bright-field image, (**b**) HAADF image, and (**c**) corresponding EDS mapping of the device. The device architecture from bottom to top is Si-SiO_2-FLG-MoS_2-MoO_3-Au-PMMA.

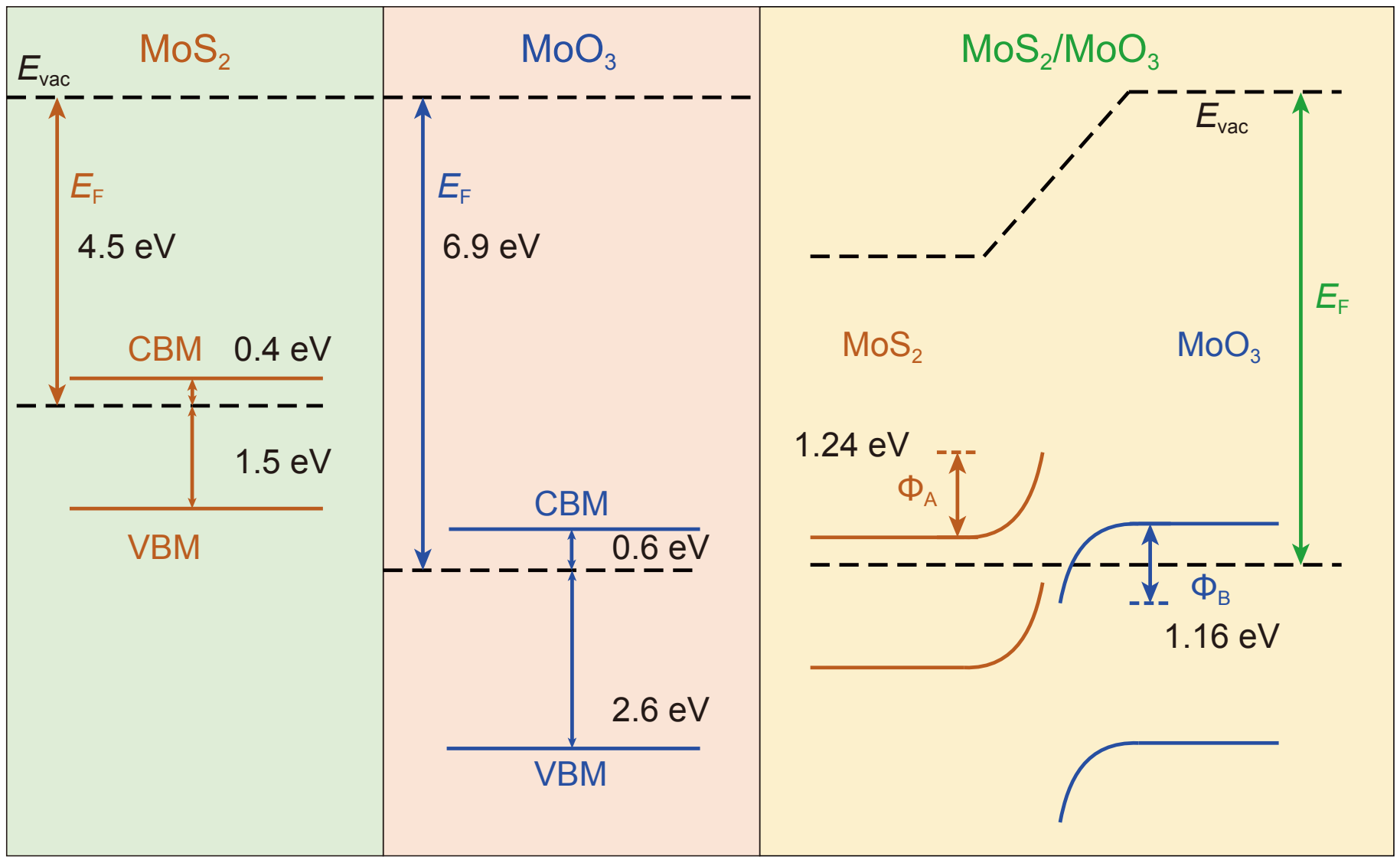

Supplementary Figure 26 Band structure of MoS_2 and MoO_3, and band alignment of MoS_2/MoO_3 heterostructure.

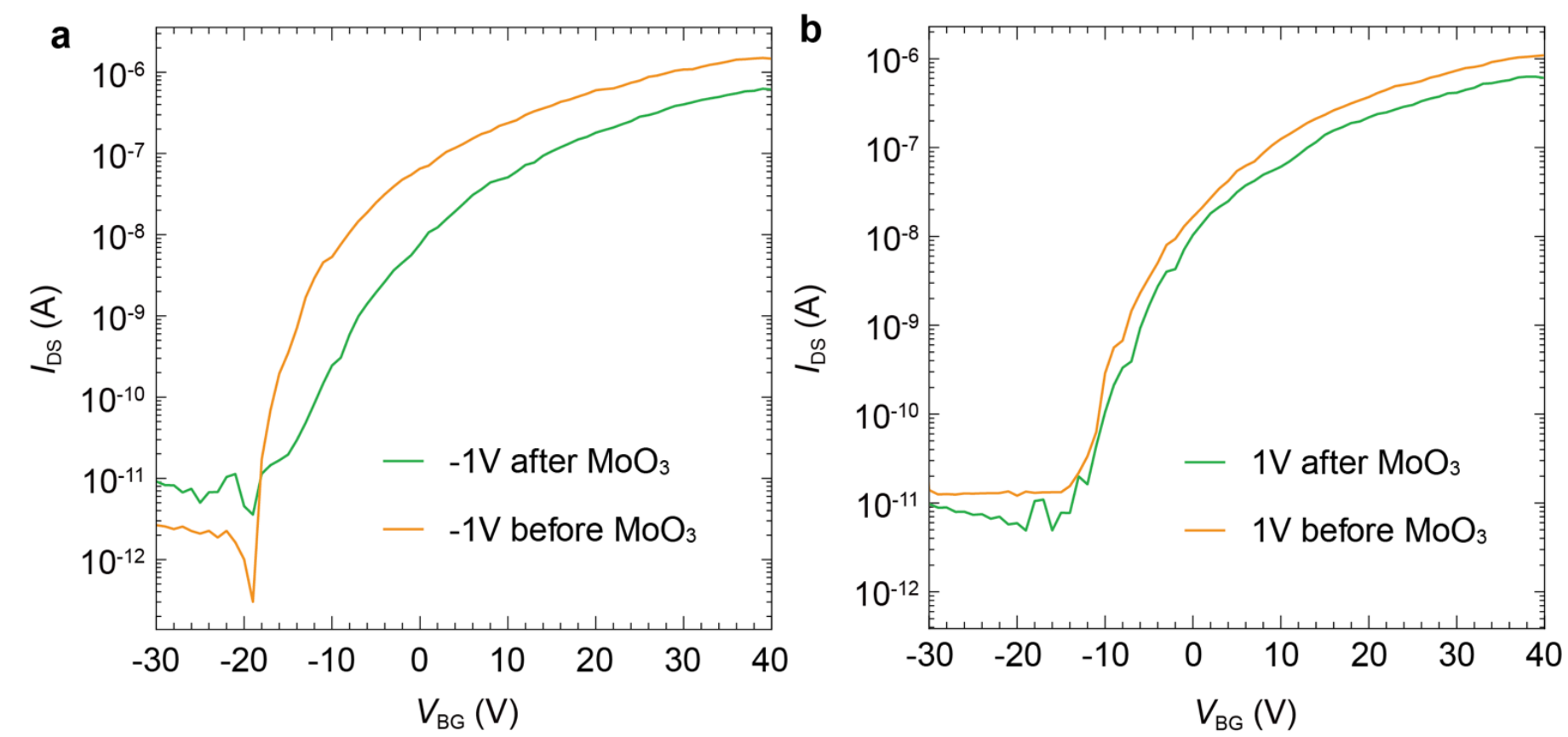

Supplementary Figure 27 The transfer curve of MoS_2 bottom-gate FET using 285 nm SiO_2 as the gate dielectric. **a,** The voltage bias was -1 V. **b,** The voltage bias was 1 V. No obvious shift of threshold voltage was observed before and after the transfer of MoO_3 flake onto the MoS_2 channel, indicating the small amount of charge transfer from MoS_2 to MoO_3.

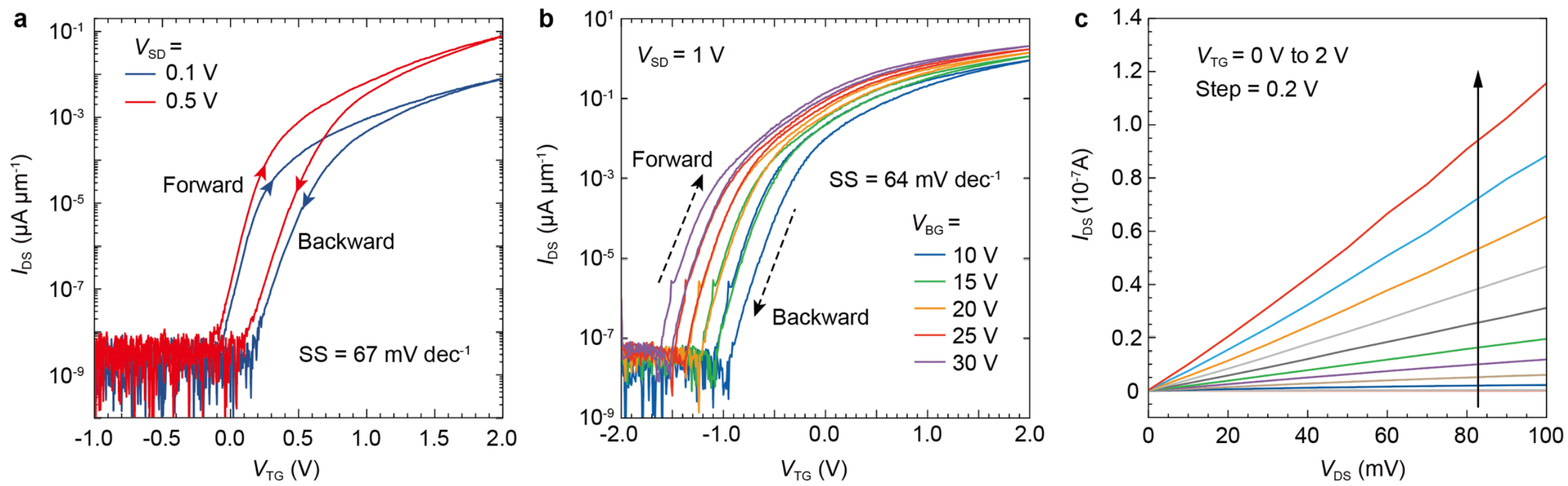

Supplementary Figure 28 Hysteretic behavior and Ohmic contact of the FET device. **a,** Hysteretic behavior of transfer characteristics with the dual sweep of V_{TG}. **b,** transfer characteristics with the dual sweep of V_{TG} at different back-gate voltages. **c**, Output characteristics of MoS_2 FET at a small V_{DS}, indicating the Ohmic contacts.

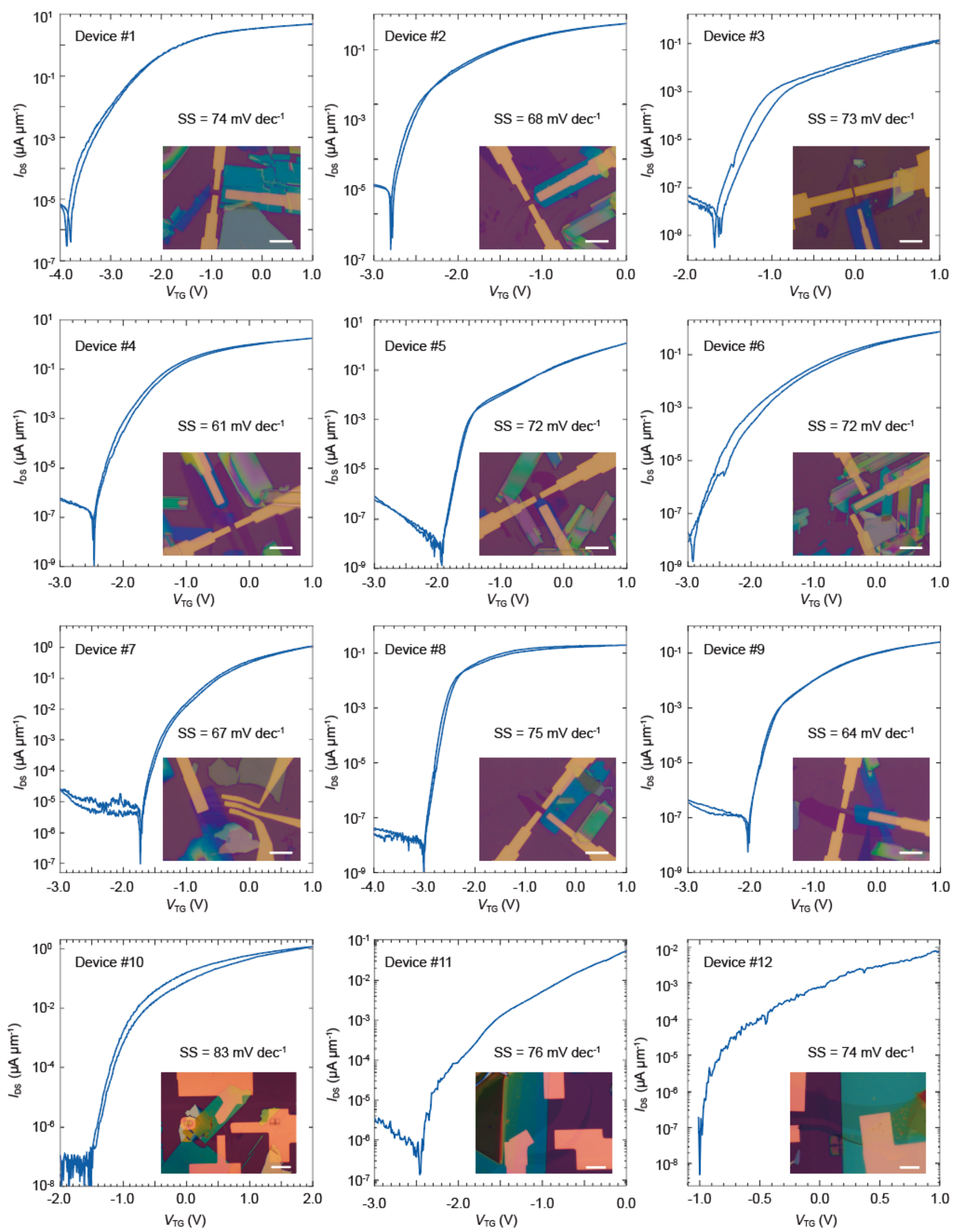

Supplementary Figure 29 Transfer characteristics of extra 12 devices, electrodes in device #1-#9 were fabricated using the dry-transfer method, electrodes in device #10-#12 were fabricated using the standard lithography and thermal deposition method. Scale bars are 10 μm.

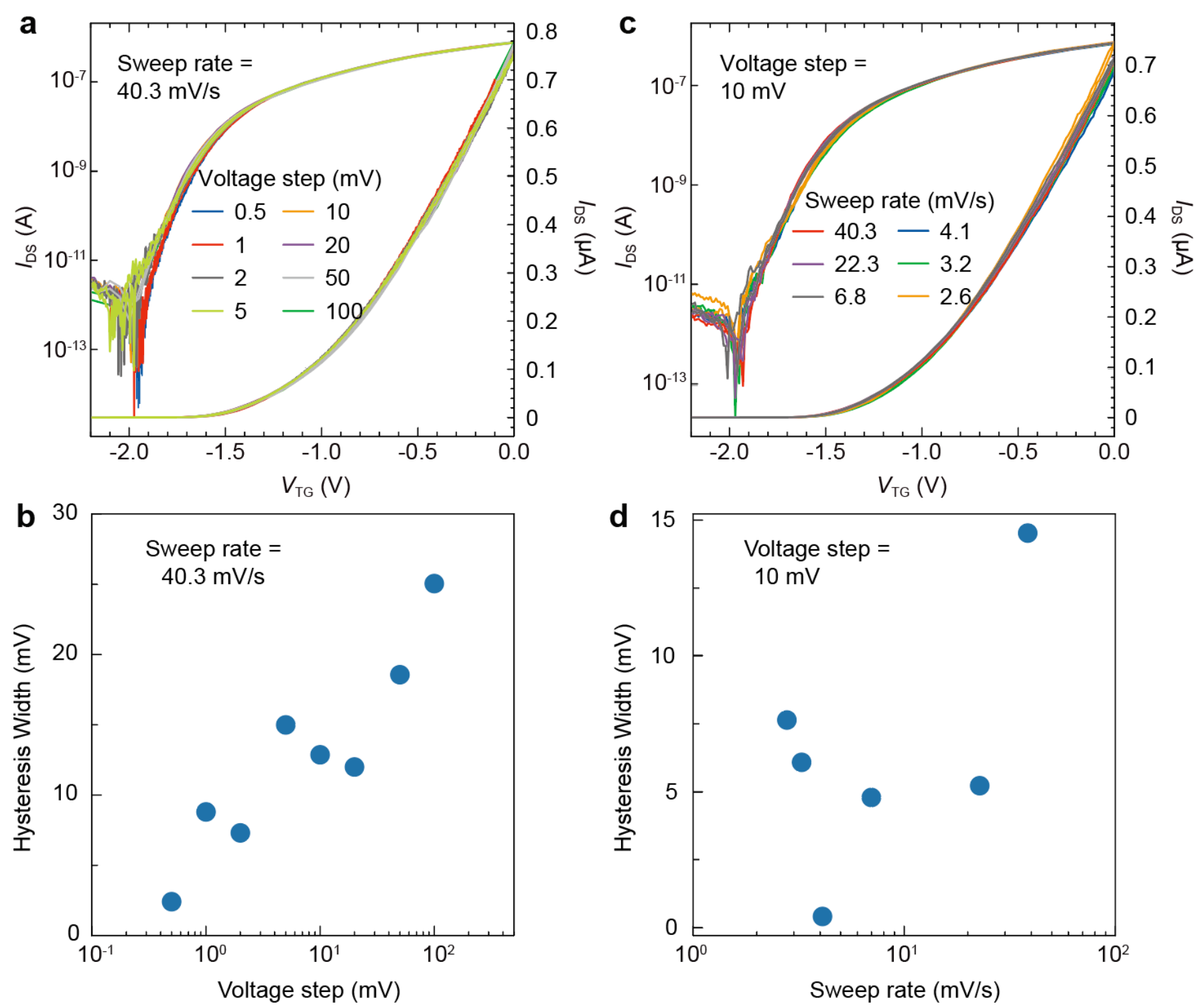

Supplementary Figure 30 Hysteresis analysis. **a, b,** Transfer curves and hysteresis width as a function of voltage step, **c, d,** Transfer curves and hysteresis width as a function of sweep rate. Overall, the hysteresis remains relatively small. Additionally, it shows a weak increasing trend with increasing sweep in the tested range and an obvious increase with the increasing voltage step. It is reasonable because a faster sweep usually gives less time for the trap-related charge process to fully equilibrate with the applied gate voltage, which leads to an offset between the forward and backward sweeps. In addition, a large voltage step not only corresponds to a faster sweep rate but also reduces the voltage resolution in the extraction, which can further enlarge the hysteresis width.

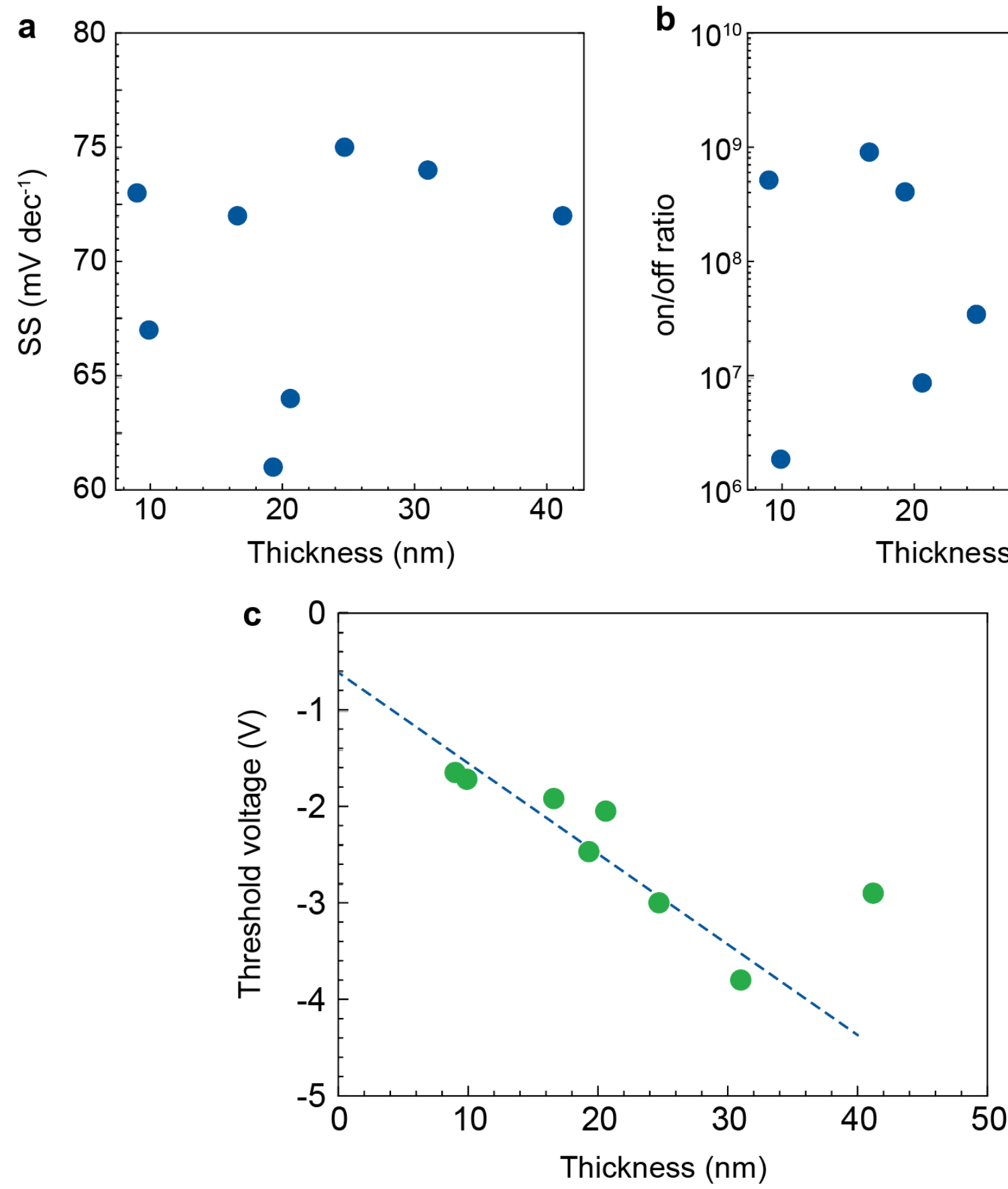

Supplementary Figure 31 Effects of MoO_3 thickness on the device performance. (**a**) SS, (**b**) current on/off ratio, and (**c**) threshold voltage. SS and current on/off ratio show no clear dependence on MoO_3 thickness. In contrast, the threshold voltage exhibits a nearly linear dependence on MoO_3 thickness, consistent with that of conventional dielectrics, indicating that MoO_3 follows the same electrostatic modulation principles as traditional dielectric layers.

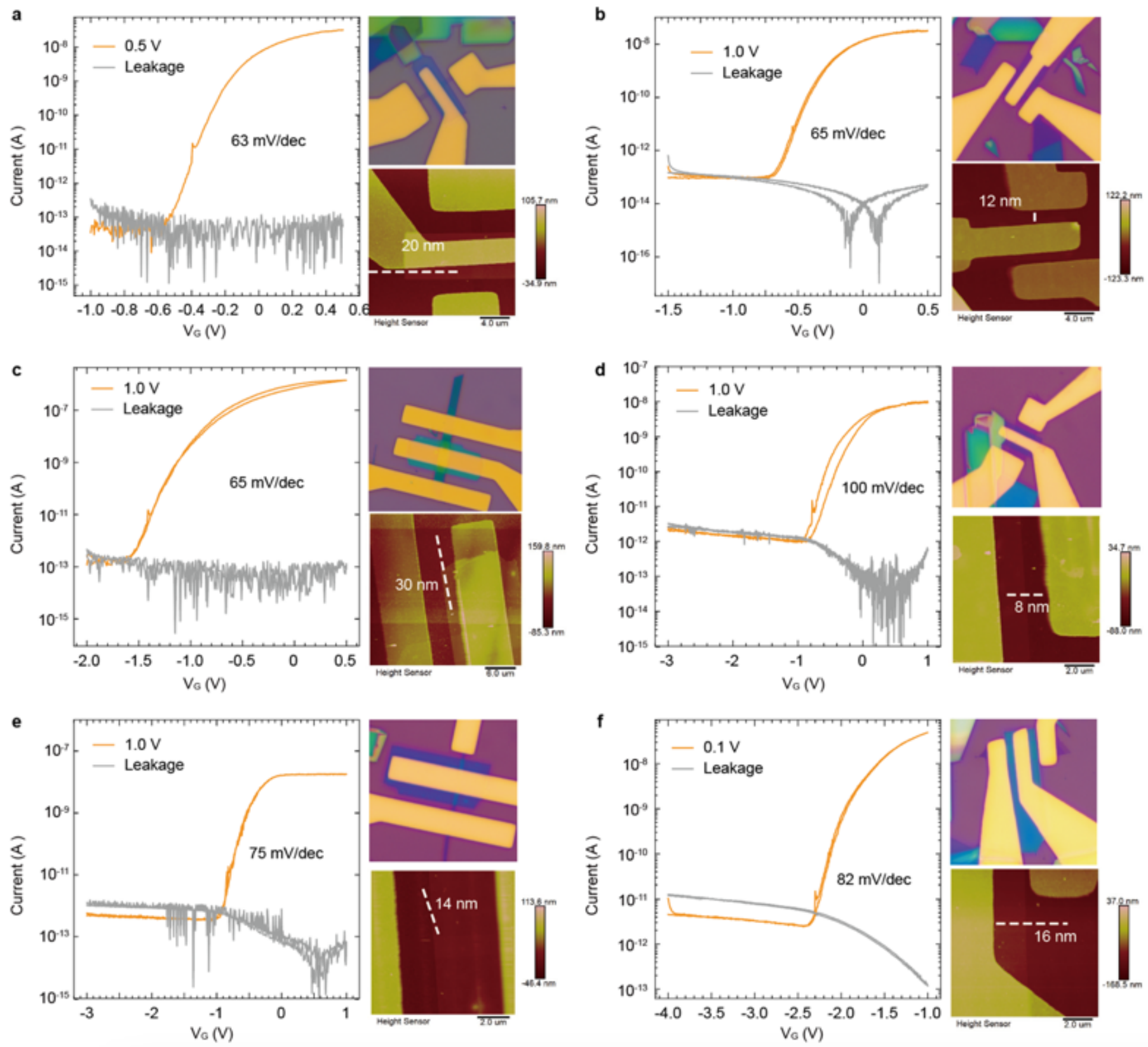

Supplementary Figure 32 Transfer curves and leakage currents of MoS_2 top-gate devices using MoO_3 as the dielectric and Au as the contacts. The corresponding optical and AFM images are presented, with the height marked.

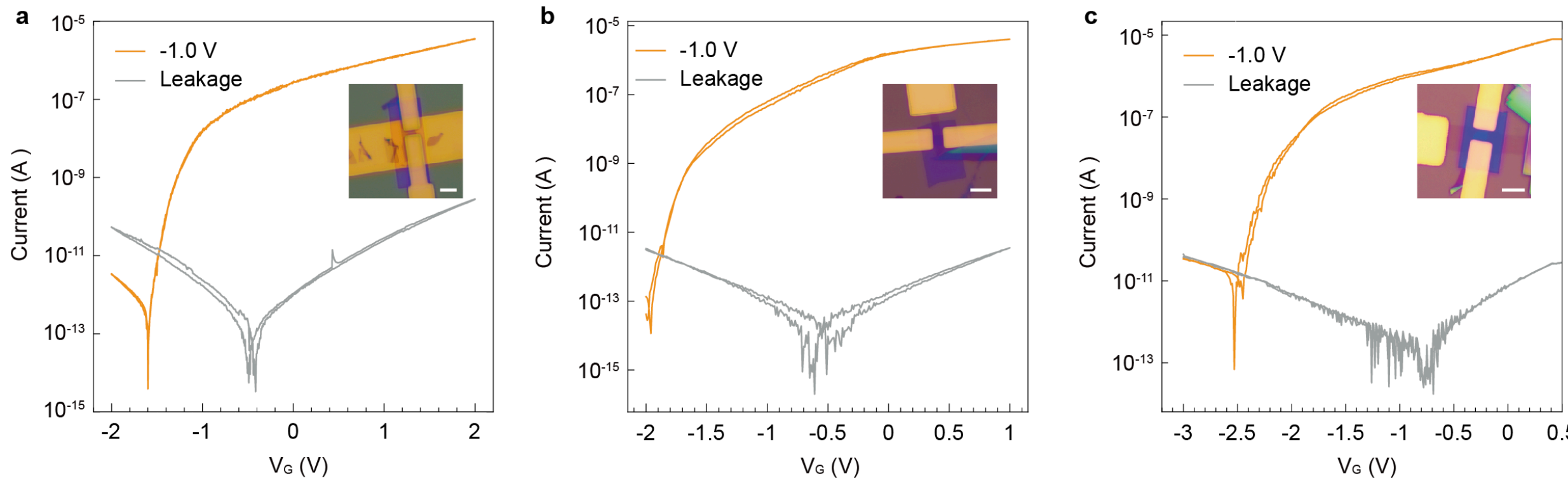

Supplementary Figure 33 Transfer curves and leakage currents of MoS_2 bottom-gate devices using MoO_3 as the dielectric and Au as the contacts, the corresponding optical image is attached. **a,** Au was used as the gate electrode. **b, c,** FLG was used as the gate electrode. Scale bars are 10 μm.

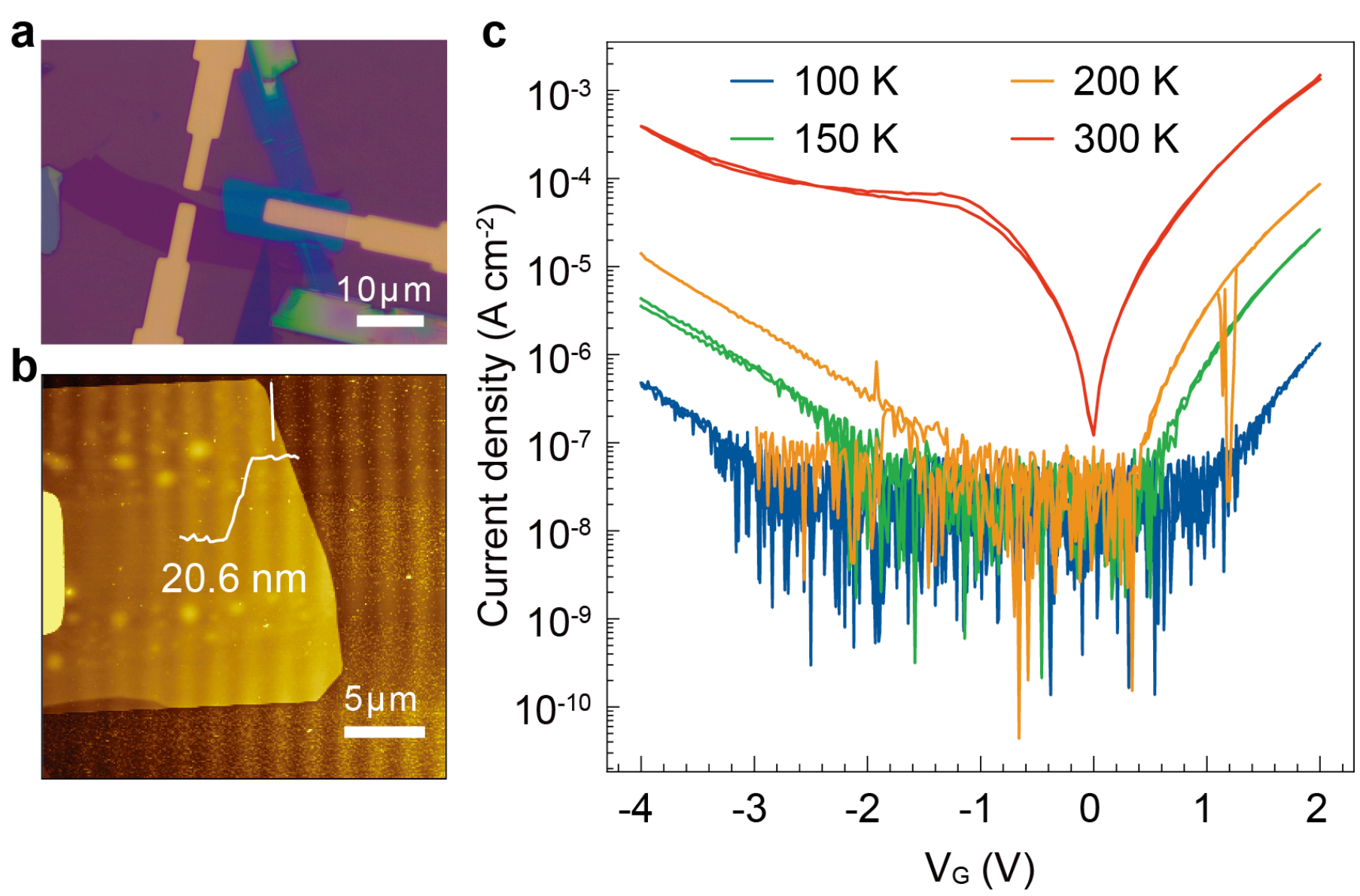

Supplementary Figure 34 The gate leakage current at different temperatures of device #9.

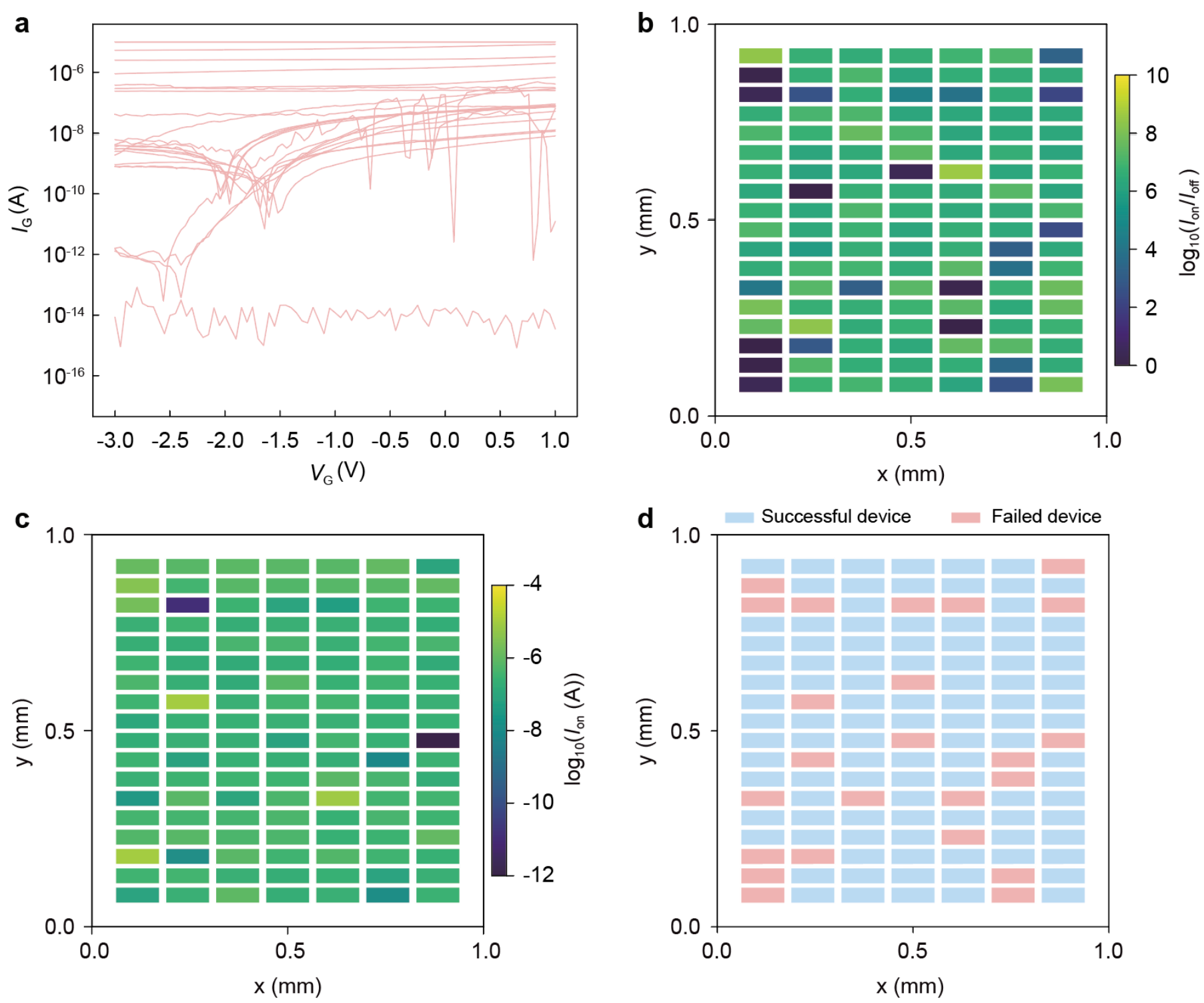

Supplementary Figure 35 Heatmaps of the key parameters of the MoS_2 FET array over a 1 × 1 mm^2 area. **a,** Transfer curves of those failed devices. **b,** I_{on}/I_{off}. **c,** I_{on}. **d,** yield map of the fabricated array.

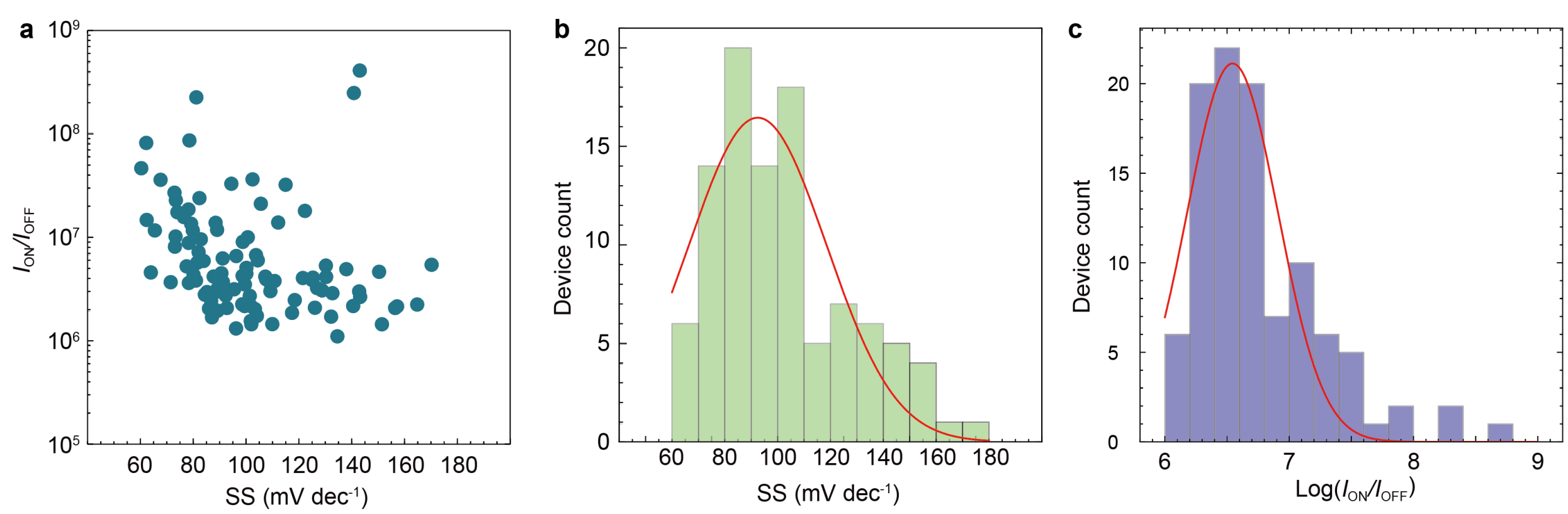

Supplementary Figure 36 Statistical analysis of SS and on/off ratio of the device array. **a**, Scattering plot of current on/off ratios and SS. **b**, **c**, Statistical distribution of SS and on/off ratio.

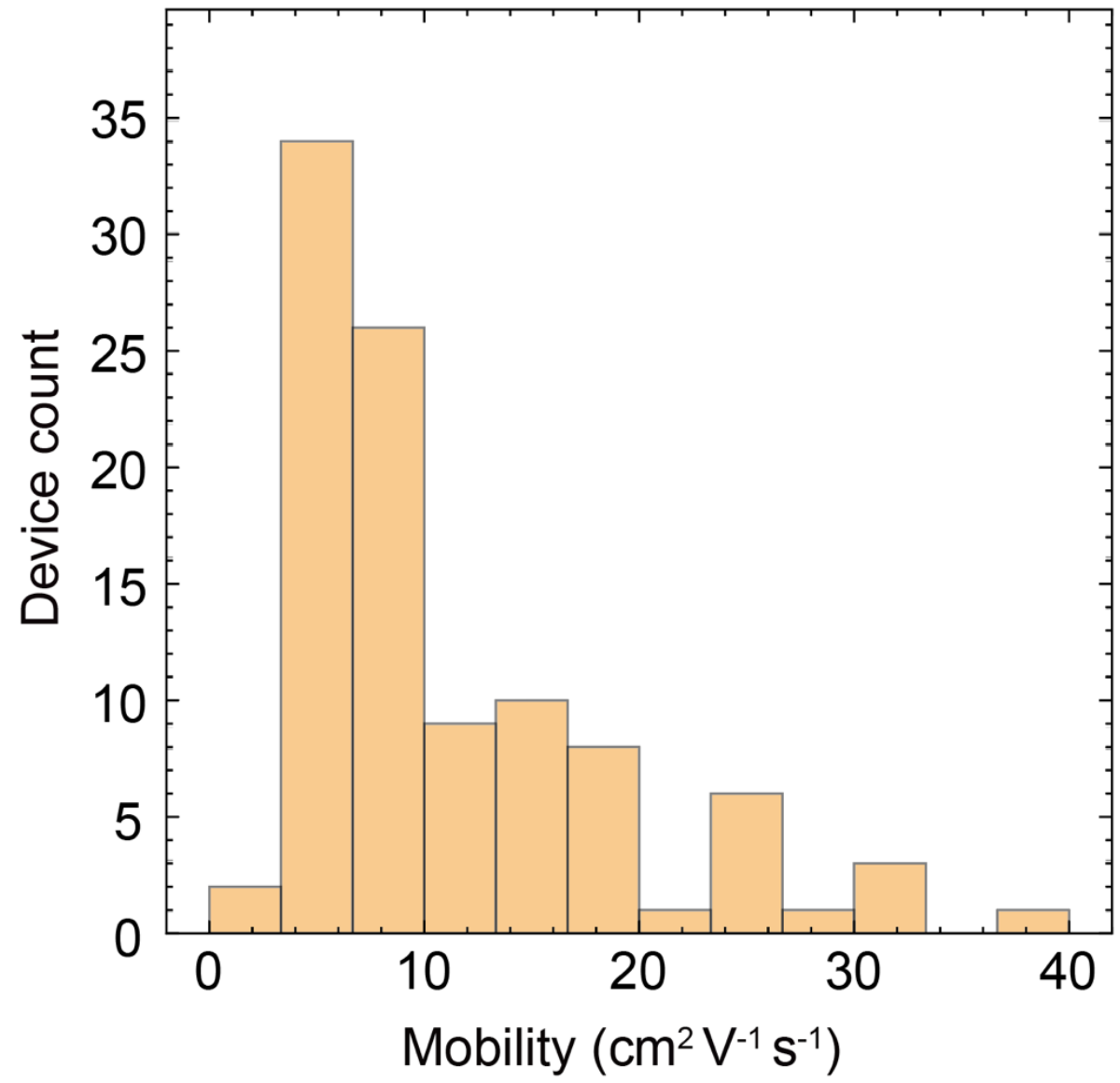

Supplementary Figure 37 Statistical analysis of the field-effect mobility of the device array. The field-effect mobility was calculated using the standard formula, as shown below:

$$\mu = \frac{L}{W}\frac{1}{C_{\mathrm{ox}}}\frac{\mathrm{d}\sigma}{\mathrm{d}V_{\mathrm{g}}}$$

where L, W, and σ are the channel length, width, and conductivity of the channel, respectively. C_{ox} is the capacitance per unit area of the MoO_3 dielectric layer, which is used as the gate dielectric with a dielectric constant of approximately 18.4. The obtained mobilities are relatively lower compared to the conventional dielectrics using the same CVD-grown MoS_2 channel[8]. This can be attributed to the presence of interface trap states at the MoO_3/MoS_2 interface, which scatter carriers and degrade mobility. The defects include the surface roughness and residual contamination at the dielectric/channel interface, introduced from the fabrication process, such as the wet-transfer of MoS_2 from the sapphire substrate, PDMS-assisted transfer of MoO_3 from the mica substrate with the help of water, and the etching process.

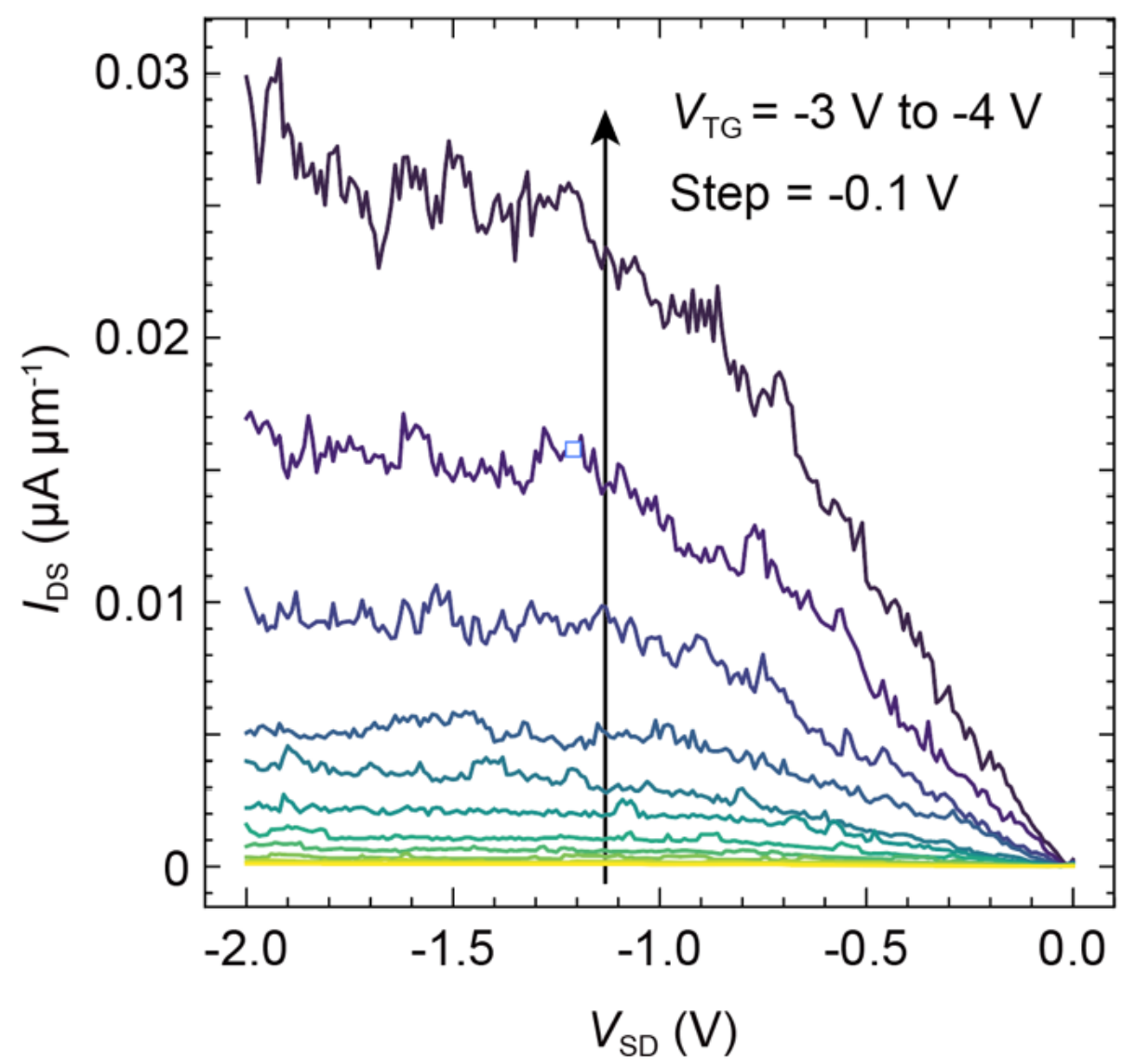

Supplementary Figure 38 Output characteristics of p-type micro-scale WSe_2 FETs, which use FLG as the source and drain contacts.

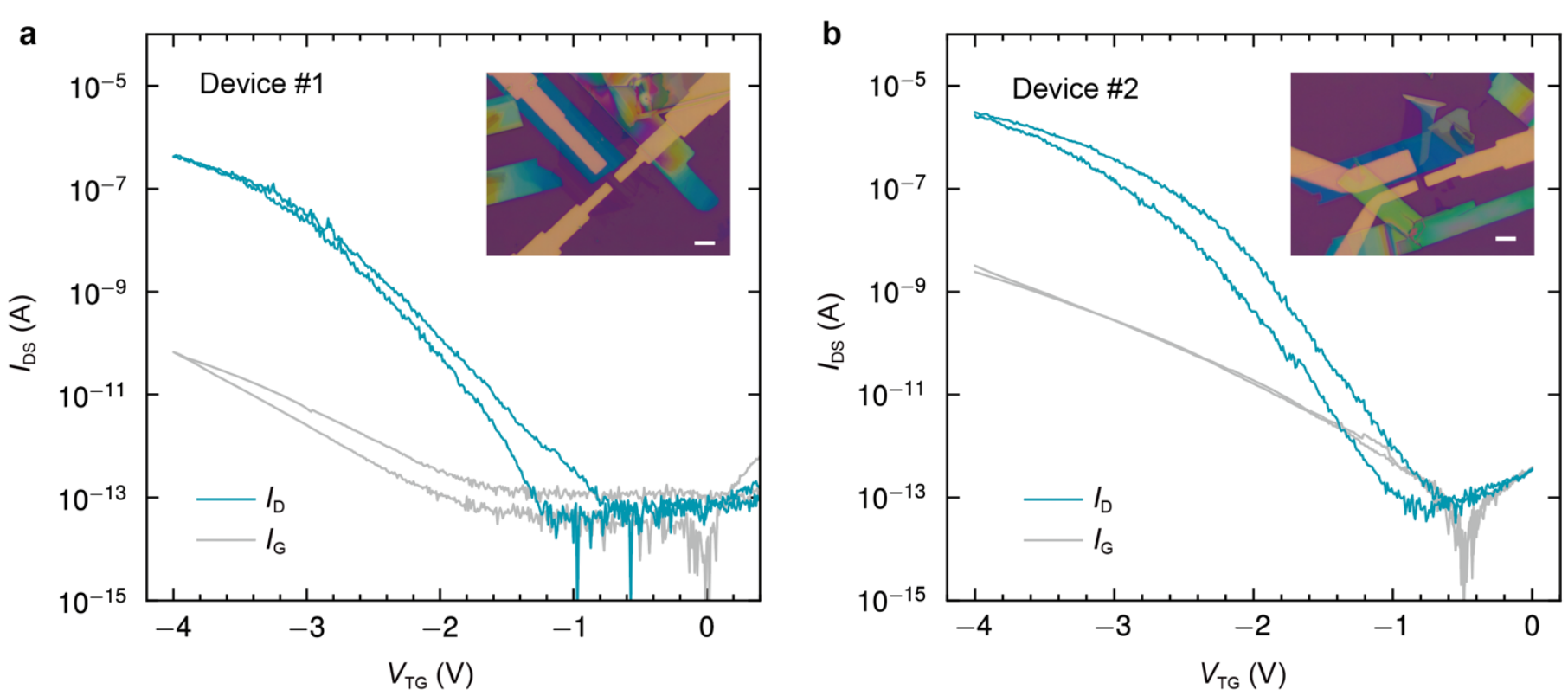

Supplementary Figure 39 Transfer characteristics of fabricated micro-scale WSe_2 FETs, the electrodes were all fabricated using the dry-transfer method. Scale bars are 10 μm.

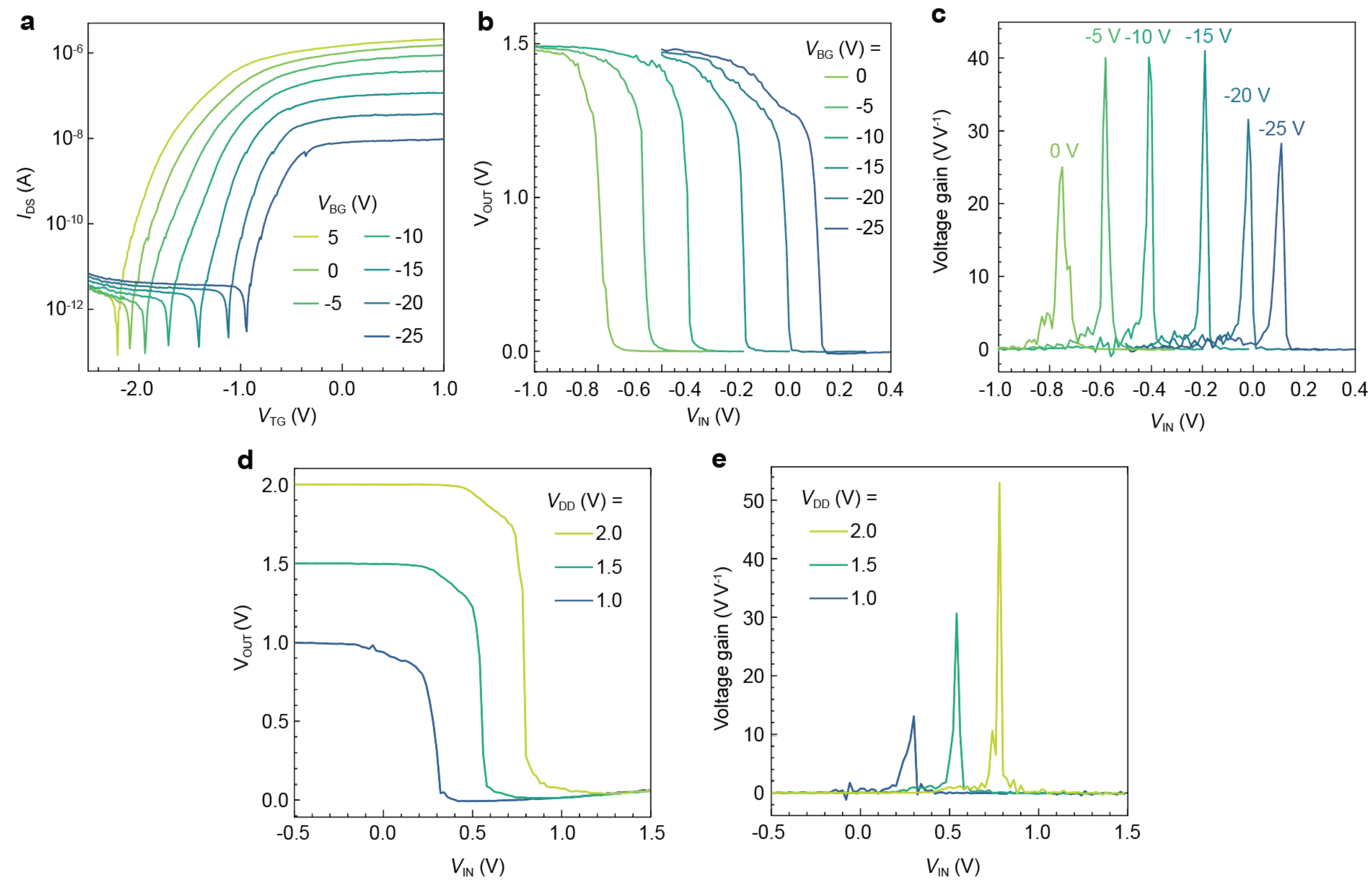

Supplementary Figure 40 V_{OUT}-V_{IN} characteristic of the CMOS inverter as a function of back-gate voltage of MoS_2 FET. **a**, transfer curves of the MoS_2 FET as a function of back-gate voltage from – 25 V to 5 V. **b**, V_{OUT}-V_{IN} curves of the CMOS inverter at different back-gate voltages of the MoS_2 transistor. **c**, the voltage gains as a function of back-gate voltage extracted from the curves in **b**. **d-e**, V_{OUT}-V_{IN} curves and corresponding voltage gains at V_{BG} = -30 V of MoS_2 FET and V_{BG} = -80 V of WSe_2 FET.

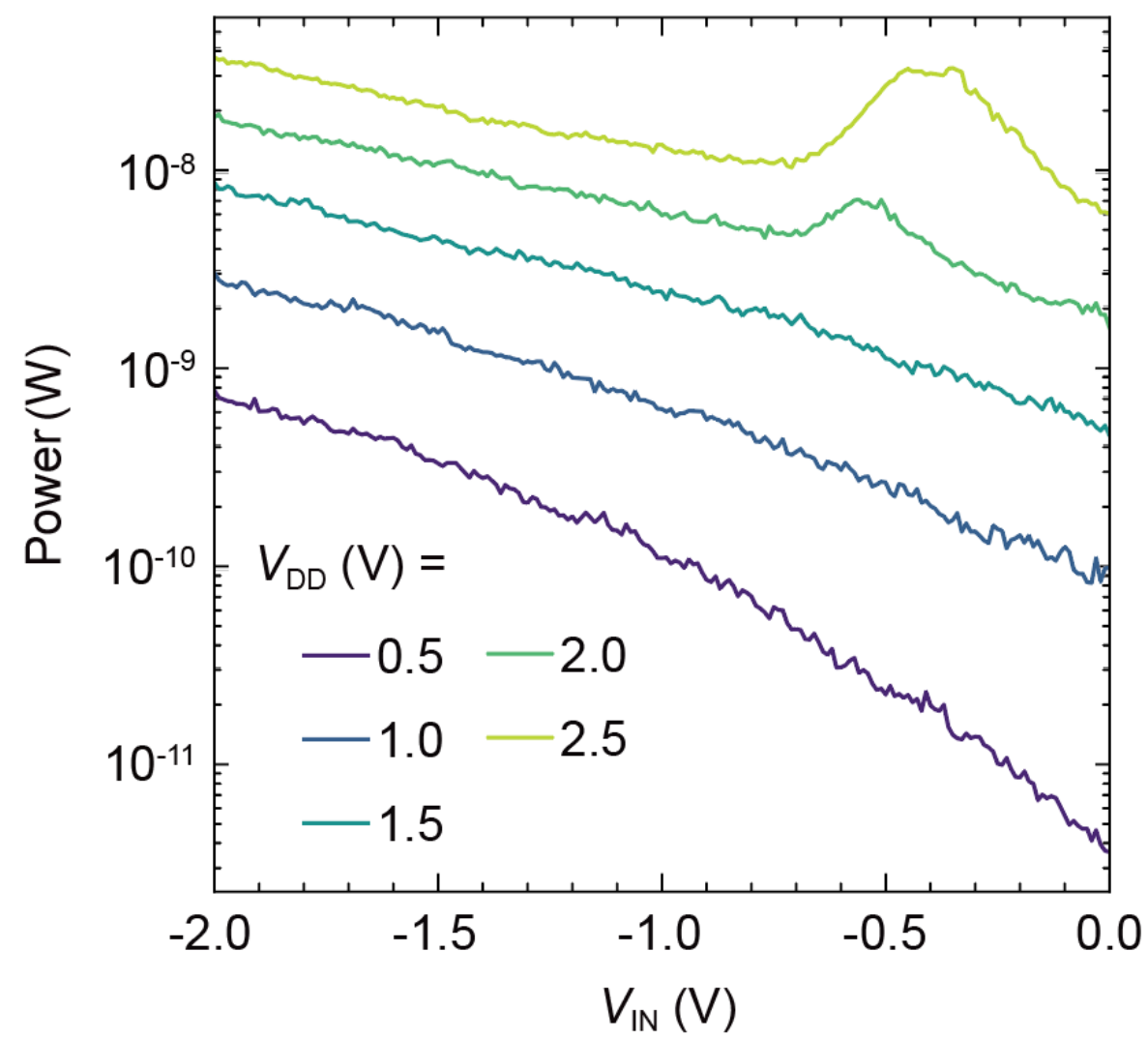

Supplementary Figure 41 The static power consumption of the CMOS inverter as a function of input voltage at different supply voltages. Noted that the electrodes for the back gate are left unconnected.

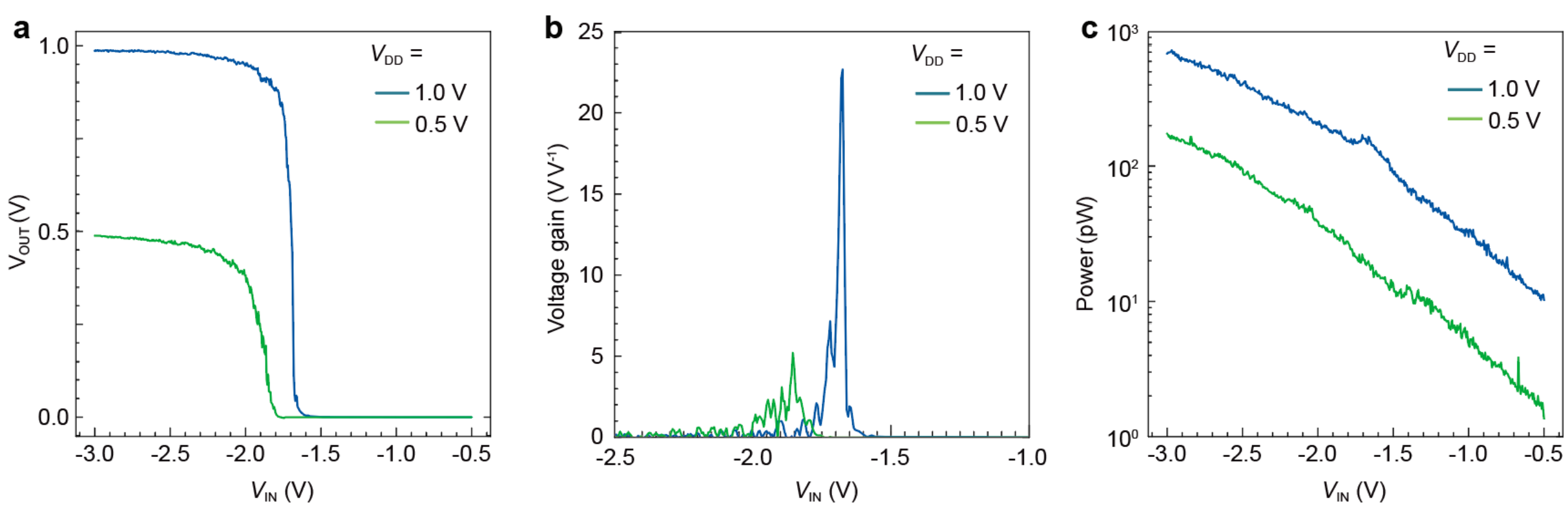

Supplementary Figure 42 The transfer curve result, voltage gain, and static power consumption as a function of input voltage of another CMOS inverter. *W*/*L* is 7 μm /3 μm for the MoS_2 transistor and 4 μm /4 μm for the WSe_2 transistor.

Supplementary Table 1 The reported MoO_3 dielectric constant.

Material	Thickness	size	Dielectric constant	Method	SS (mV/dec)	On/off ratio	Supplementary References
PVD	9.31 - 208 nm	~50 μm	40	C-V	78	7E7	9
Exfoliation	15-20 μm	~50 μm	35.1	C-V	2200	1E3	10
van der waals epitaxy	>1.4 nm	~20 μm	6 for 2L; 10.5-11 for >3L	F-D	-	-	11
exfoliation	48.2 nm	~100 μm	27.2	C-V; Dual-gate	-	-	12
MoO_3 nanostructure	~33 nm	~1 μm	25	EFM	-	-	13
CVD	3 nm	~10 μm	40	C-f	-	-	14
PVD	90 nm	100 μm	35	From reference	400	1E5	15
van der waals epitaxy	35 nm-500 nm	400 nm	18	C-V			16
PVD	108 nm-243 nm	-	5.7	refractive index	-	-	17
ALD	-	~46 nm	17	C-V	-	-	18
-	-	-	5.0	DFT+U	-	-	19
-	-	-	5.3	DFT with local-field effects	-	-	20
**CVD**	**~ 4.2 nm**	**~2 mm**	**~18.4**	**C-V; Dual-gate**	**62**	**1E8**	**Our work**

Supplementary Note 1 Fast-cooling method

The fast-cooling method can be used to increase the domain size of transition metal dichalcogenides such as WSe_2. WSe_2 was synthesized in a single-zone chemical vapor deposition (CVD) system using SiO_2/Si substrates. In the growth process, 5 g of precursor (WSe_2 powder) was used, the argon gas flow rate was maintained at 150 sccm, and the furnace temperature was increased to 1170 °C under atmospheric pressure. After the 2 min growth stage, the sample was subjected to a fast-cooling process. In this work, the fast-cooling method refers to immediately pulling the substrate out of the heating zone once the growth is completed, so that the sample rapidly leaves the high-temperature region and cools down quickly. Switchable gas flow was applied to accelerate heat dissipation and remove residual reactants around the substrate[21]. The fast-cooling process and switched gas flow together help suppress continued deposition and secondary nucleation during the cooling stage, thereby preserving the as-grown WSe_2 domains. By contrast, in the conventional cooling process, the gas-flow direction was not switched after growth, and the substrate was kept in its original position until the furnace cooled naturally to room temperature, after which the substrate was taken out. Except for the cooling protocol, the precursor amount, substrate type, gas flow rate, growth temperature, and growth time were all kept the same.

As shown in Supplementary Figure 43, without this method, the WSe_2 domain size was approximately 20 μm. However, when the fast-cooling method was employed, the WSe_2 domain size increased to approximately 444 μm, which is approximately 20 times larger.

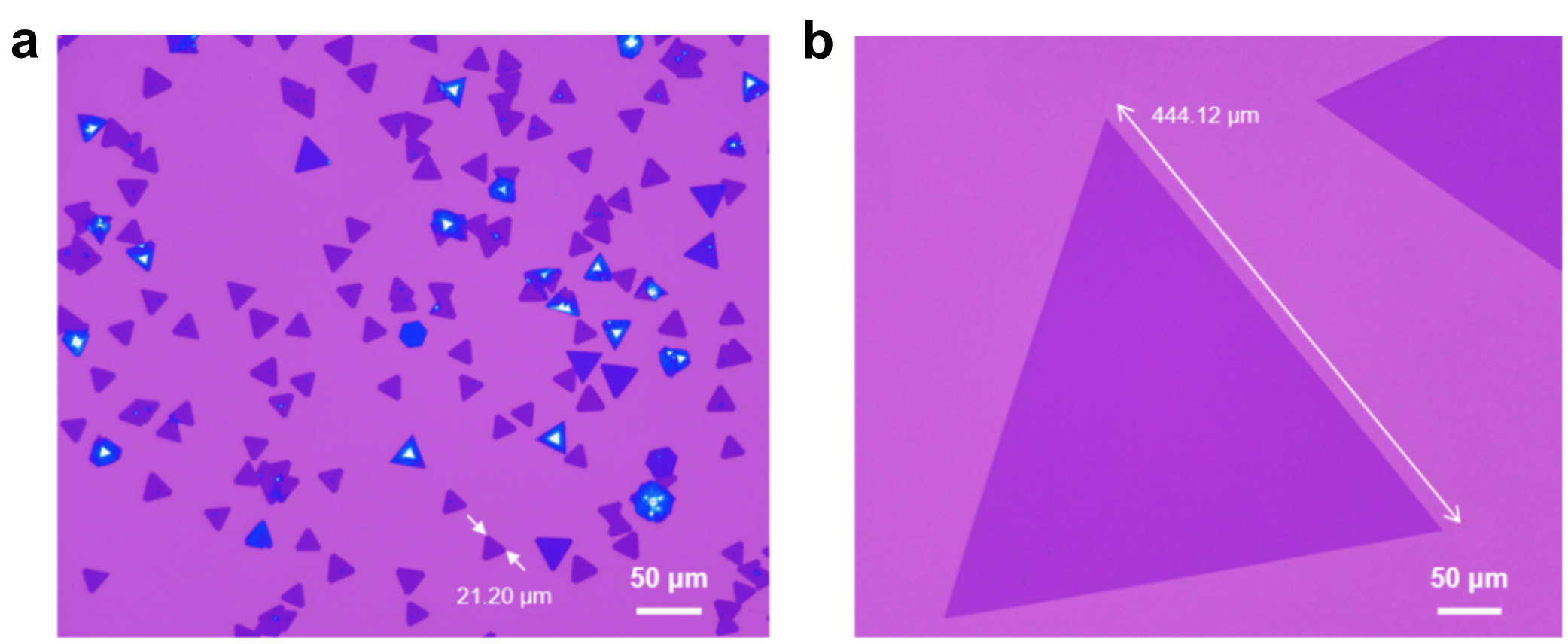

Supplementary Figure 43 The effect of the fast-cooling methods on the increase in WSe_2-domain size. a, The WSe_2 domains prepared without fast-cooling, **b,** The WSe_2 domains prepared using the fast-cooling method. The white arrow indicates the characteristic domain size. Scale bar: 50 μm.

Supplementary Note 2 Estimation of the dielectric constant in *C*-*V* measurement

In the estimation of the dielectric constant in the *C*-*V* measurements, we need to carefully consider the composition of capacitance. The capacitance is the series combination of three main terms[22]: the MoO_3 capacitance, the contribution of interfacial residue, and the capacitance of the few-layer graphene (FLG) electrode.

The contribution of interfacial residue is mainly from the PDMS residue (κ = 2.3 - 2.8)[23] and air bubbles after the dry transferring method. The annealing process was taken after the device fabrication, however, the removal of polymer residue still remains challenging. We assume that the residue in all the devices is almost the same due to the same fabrication process and environment.

The capacitance of the FLG electrode is from the quantum capacitance, which is important for low-density-of-state materials, such as graphene[24,25]. For graphene, the quantum capacitance C_{Q} is given by the relation

$$C_{\mathrm{Q}} = \rho e^2 = \frac{2e^2\mu}{\pi\hbar^2{\upsilon_F}^2}$$

where e is the elementary charge, $\hbar$ is the reduced Planck constant, υ_F is the Fermi velocity in graphene, and μ is the chemical potential. For multilayer graphene, the quantum capacitance is approximately linear with the layer number for low energies. In our experiments, the graphene electrodes are approximately 10 layers (3 - 4 nm). According to the quantum capacitance relation, the value is approximately 23.6 $\mu F\ cm^{-2}$ for the 10-layer graphene in the chemical potential of 0.1 eV.

The total capacitance is

$$\frac{1}{C} = \frac{1}{C_{\mathrm{OX}}} + \frac{1}{C_{\mathrm{D}}} + \frac{1}{C_{\mathrm{Q}}}$$

C is in the range of a few 0.1 $\mu F\ cm^{-2}$ from our *C*-*V* measurements. However, C_{Q} is in the range of 10 $\mu F\ cm^{-2}$. Therefore, the influence of C_{Q} is almost negligible.

In addition, according to the equation $C = \frac{\varepsilon\varepsilon_0}{t}$, we have

$$\frac{t}{\varepsilon_{\mathrm{eff}}} = \frac{t}{\varepsilon_{\mathrm{ox}}} + \mathrm{D}$$

where D contains the contribution from the interfacial polymer residue and the quantum capacitance of FLG. As we have equivalent oxide thickness EOT = 3.9 $t_{\mathrm{ox}}/\varepsilon_{\mathrm{ox}}$, then we have ECT

= EOT + t_D, where ECT stands for effective capacitance thickness and is define as ECT = 3.9 t_{ox}/ε_{eff}, and t_D is the difference of EOT and ECT, which is from interfacial polymer residue and the quantum capacitance of FLG (Noted that ECT is also called CET, an abbreviation of capacitance equivalent thickness). By fitting $\frac{t}{\varepsilon_{eff}}$ with respect to thickness t, we got the constant D is approximately 1.9 nm, and ε_{bulk} is approximately 18.5.

Supplementary Note 3 Estimation of the electric field at breakdown.

The dielectric breakdown is a statistical process rather than a single deterministic "breakdown field". To address this, we have analyzed our MoO_3 devices using a Weibull statistical approach, which is widely applied to evaluate breakdown behavior in dielectric films. In this method, the cumulative failure probability P is calculated from the measured devices according to $P_i = (i\text{-}0.5)/N$, where i is from 1 to N, and N is the number of measured devices. Note that the measured device should be arranged in ascending order of E (E denotes the electric field at breakdown). The statistical distribution of E was then fitted using the two-parameter Weibull distribution:

$$P = 1 - \exp[-(\frac{E}{E_0})^{\beta}]$$

where E_0 is the electric field when P is approximately 63.2%, and β is the shape parameter representing the distribution spread.

We performed breakdown measurement on multiple MoO_3 devices with the same thickness of ~2.0 nm (Supplementary Figure 44b) at room temperature, as shown in Supplementary Figure 44a. As we optimized the growth procedure, the materials' size allows us to fabricate multiple devices with consistent channel area and the same layer thickness, which is essential for statistical analysis. All devices were fabricated using a single flake at the same time, ensuring the comparison and Weibull fitting were meaningful. Supplementary Figure 44c presents the current density as a function of electric field collected from 11 devices, the electric field at device breakdown can be extracted. According to P and E, we plotted the $\ln[-\ln(1-P)]$ against $\ln(E)$ as shown in Supplementary Figure 44d. β and E_0 were obtained from the slope and intercept of the linear fitting. Supplementary Figure 44e presented P versus E, together with the Weibull fitting curve calculated using β and E_0. E_0 is marked with a dashed circle, approximately 26.75 MV cm^{-1}.

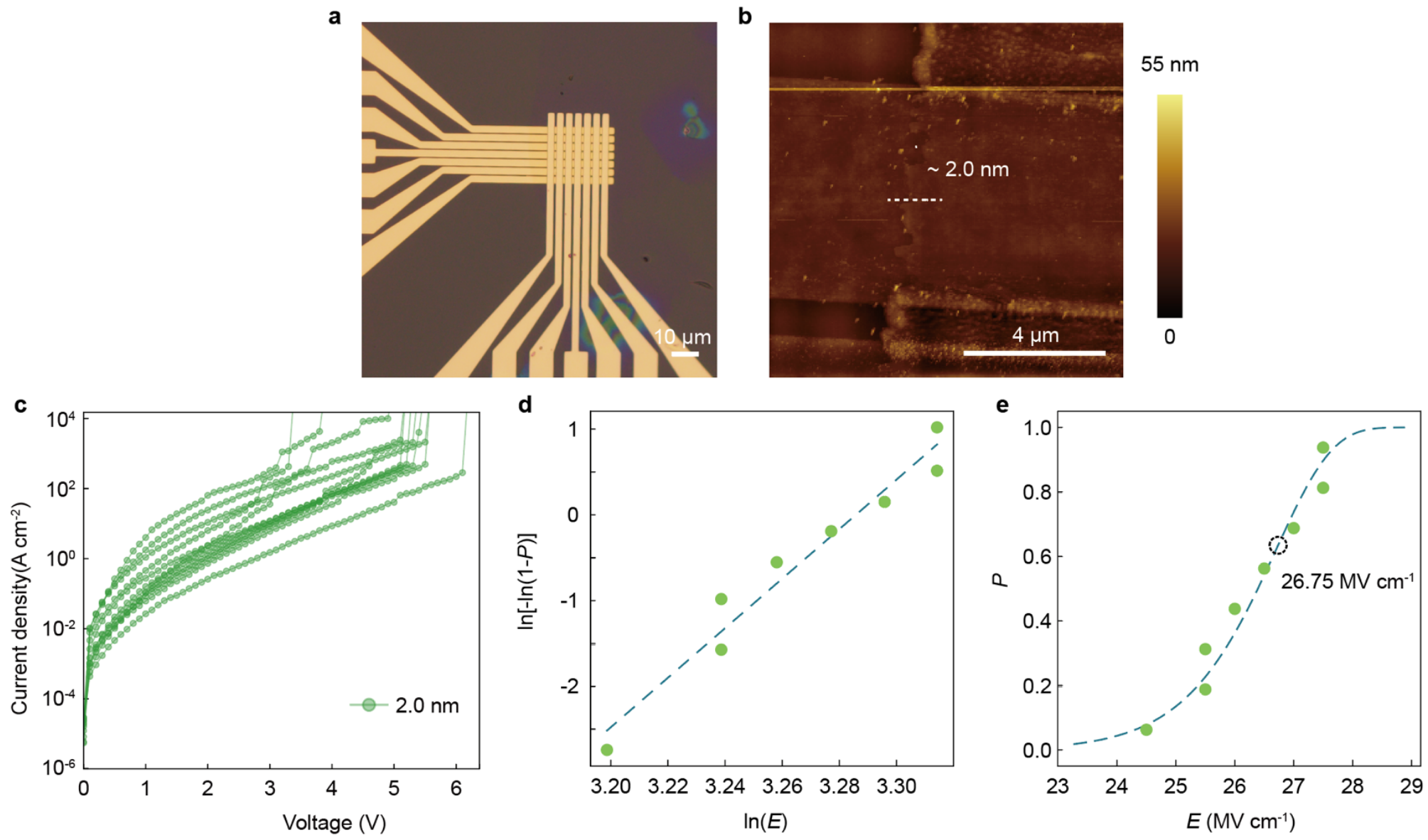

Supplementary Figure 44 Breakdown characterization of MoO_3. **a,** Optical image of the fabricated Au-MoO_3-Au devices. **b,** The AFM height profile of the device, showing a thickness of approximately 2.0 nm. **c,** The current density as a function of the voltage of the MoO_3 devices. **d,** The plot of $\ln[-\ln(1-P)]$ versus $\ln(E)$ and their linear fitting, from where we extracted β and E_0. **e** The plot of cumulative failure probability P versus electric field, together with the Weibull fitting curve calculated using β and E_0. The dashed circle marks the electric field when P is approximately 63.2%, corresponding to E_0, which is approximately 26.75 MV cm^{-1}.

Supplementary Note 4 Reliability test

We systematically evaluated the time-dependent dielectric breakdown (TDDB) of Au-MoO_3-Au metal-insulator-metal (MIM) structures with a fixed MoO_3 thickness (29.5 nm). These devices were subjected to constant voltage stresses ranging from 18 V to 22 V at room temperature, with the time-to-breakdown (t_{BD}) recorded for each voltage.

The results revealed a strong exponential dependence of t_{BD} on the applied voltage. At lower voltages near the operational range, no breakdown was observed within the maximum testing window. As the voltage increased to stress levels (18 V to 22 V), t_{BD} decreased sharply from ~3 hours to ~15 seconds as shown in Supplementary Figure 45a, consistent with field-accelerated dielectric degradation mechanisms.

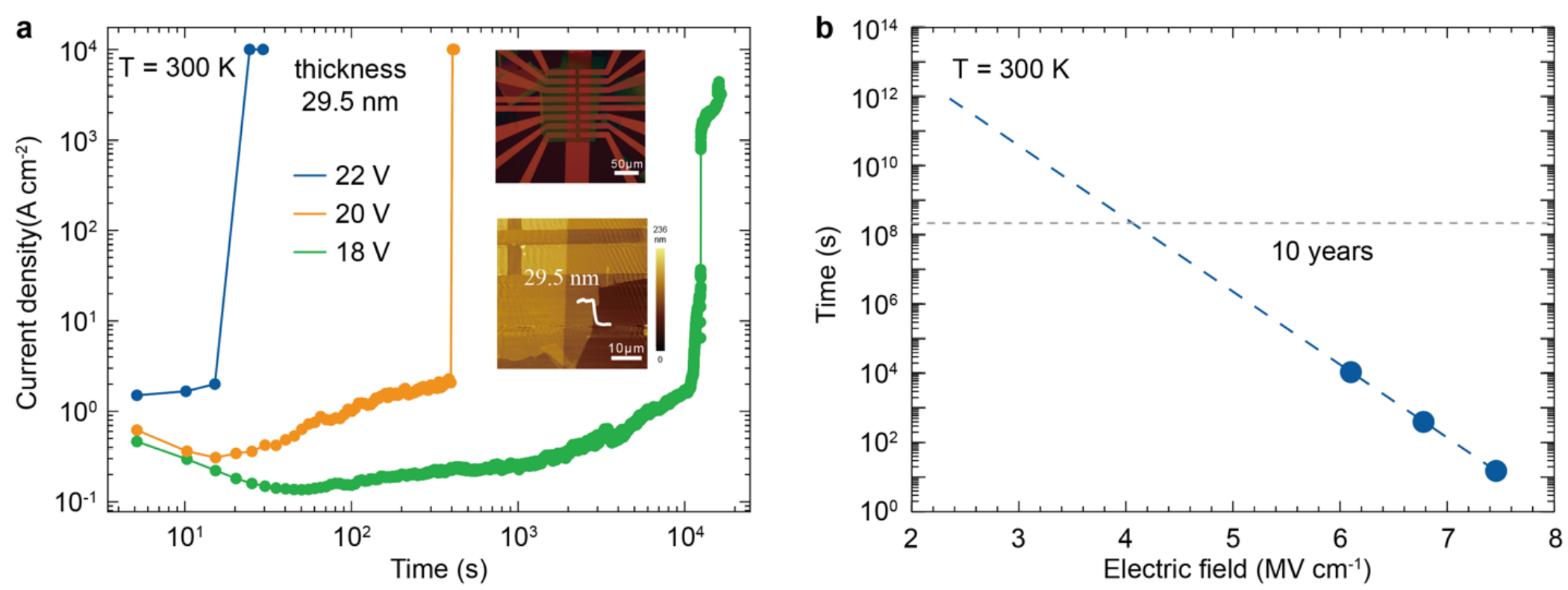

Supplementary Figure 45 Lifetime prediction. a, Time to breakdown of metal-insulator-metal devices with a 29.5 nm thick MoO_3 dielectric at various voltage stresses. **b**, Time to breakdown versus stress voltage characteristics, where the blue dashed line represents the fitting for lifetime prediction.

To quantify the lifetime under realistic operating conditions, we applied the E-model for electric field-driven TDDB to extrapolate the t_{BD} data[4,26]. The fitted curve (Supplementary Figure 45b) enabled us to project a t_{BD} exceeding 10 years under an electric field of 2.5 MV cm^{-1}. This prediction is consistent with the stable transfer characteristics observed in MoO_3-based FETs.

We investigated the stability of MoO_3-based FETs under continuous gate voltage (1 V) and repeated on and off states in vacuum at room temperature. The continuous $V_g = 1$ V and input pulses with $V_g = -2$ or 1 V as well as a constant $V_d = -1$ V were applied to the device for more than 80000 s (Fig. 3h) and 7000 s (Supplementary Figure 46), respectively, as shown in Supplementary Figure 46. Notably, a negligible variation in the cyclic tests was observed during this evaluation of the long-term operational stability. Specifically, the gate leakage current, on current, off current,

and current on/off ratio were maintained nearly constant during the entire test, including the continuous and pulsed bias.

In addition, we characterized the device stability under ambient conditions. There is no obvious change as shown in Supplementary Figure 47, indicating the good stability of the MoO_3 device during storage under ambient conditions. When we measured the device under a continuous gate voltage in ambient conditions, the current experienced a slow degradation, which could be from the surface adsorption of ambient moisture and oxygen molecules[27].

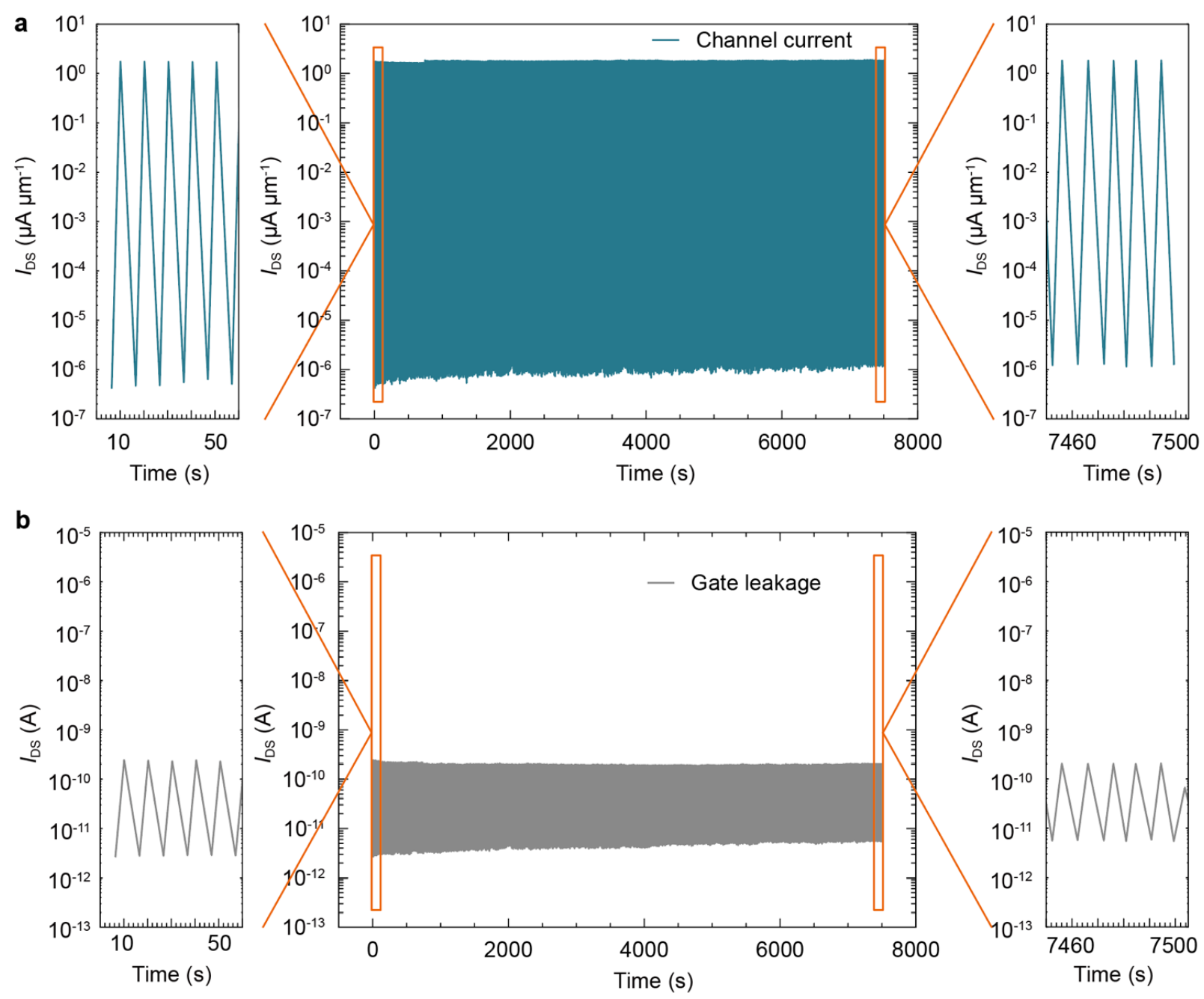

Supplementary Figure 46 Operational stability test. The operational stability of the FETs under over 7000 s of periodic inputs.

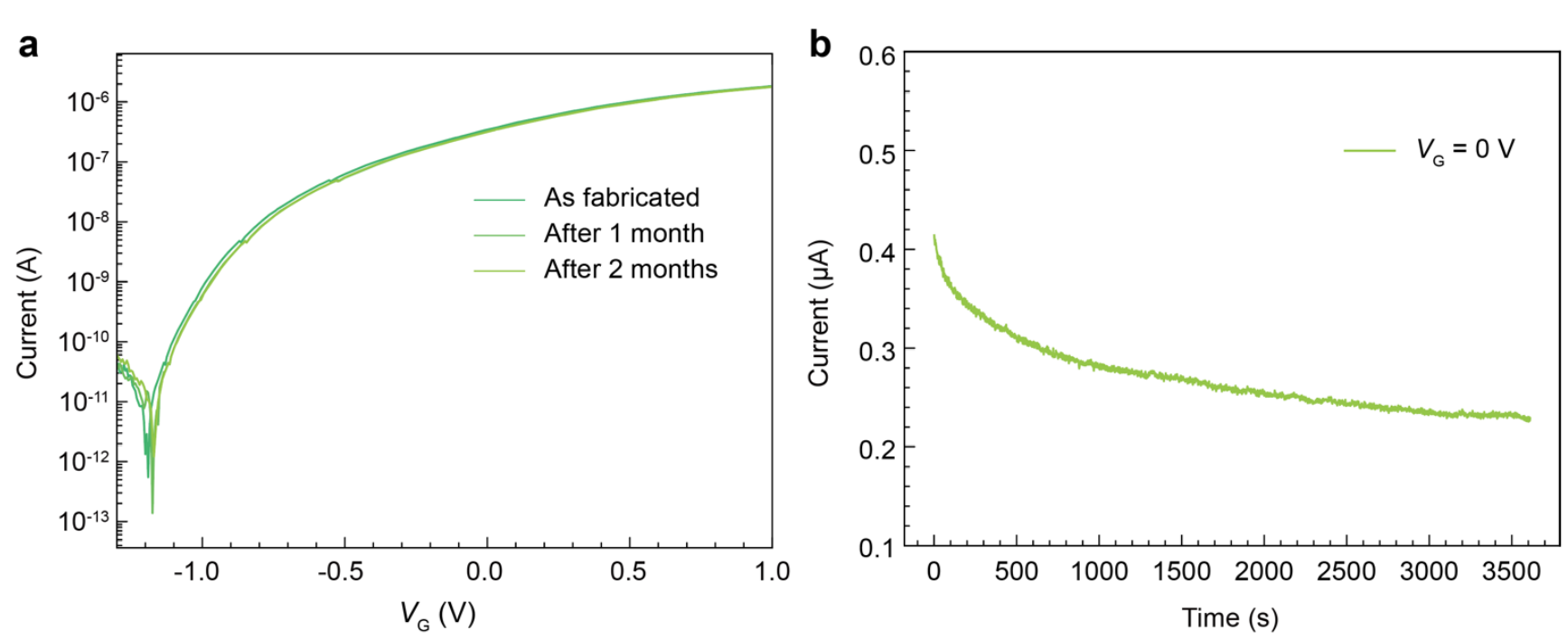

Supplementary Figure 47 Stability test of the MoO_3 device in ambient conditions. a, The transfer curves of the device as fabricated, after 1 month, and after 2 months. **b,** The operational stability of the device under a continuous gate voltage of 0 V for 1 hour in ambient conditions.

Supplementary Note 5 Estimation of the interfacial trap density

We carried out direct quantitative evaluations of the trap density D_{it} using three complementary approaches, including frequency-dependent *C-V* conductance analysis[28], charge pumping[29], and low-frequency $1/f$ noise measurements[30,31].

***C-V* conductance analysis.** We carried out frequency-dependent capacitance and conductance measurements on the Au(bottom)/MoO_3/MoS_2/Au(top) bottom-gate structure using the Keithley 4200-CMU system. During the measurement, the top Au electrode was grounded, while a small AC signal superimposed on a DC bias was applied to the bottom Au electrode. To extract the interfacial trap response, we plotted G_p/ω as a function of frequency under different DC bias as shown in Supplementary Figure 48a. G_p is the AC conductance of the traps and is obtained by considering the dielectric contribution of the MoO_3 layer using the formula below[32]:

$$\frac{G_p}{\omega} = \frac{\omega G_m C_{\mathrm{ox}}^2}{G_m^2 + \omega^2 (C_{\mathrm{ox}} - C_m)^2}$$

where $\omega = 2\pi f$, C_m and G_m are the measured capacitance and conductance of the device, and C_{ox} is the effective capacitance of the MoO_3 dielectric. The interface trap density was then estimated from the peak value of the corrected conductance using the equation

$$D_{it} \approx \frac{2.5}{qA} \left(\frac{G_p}{\omega}\right)_{peak}$$

where q is the elementary charge, and A is the device area. The extracted D_{it} values as a function of voltage are plotted in Supplementary Figure 48b. The interface trap density is on the order of ~5×10^{11} cm^{-2} eV^{-1} and shows little dependence on V_G within the measured bias range.

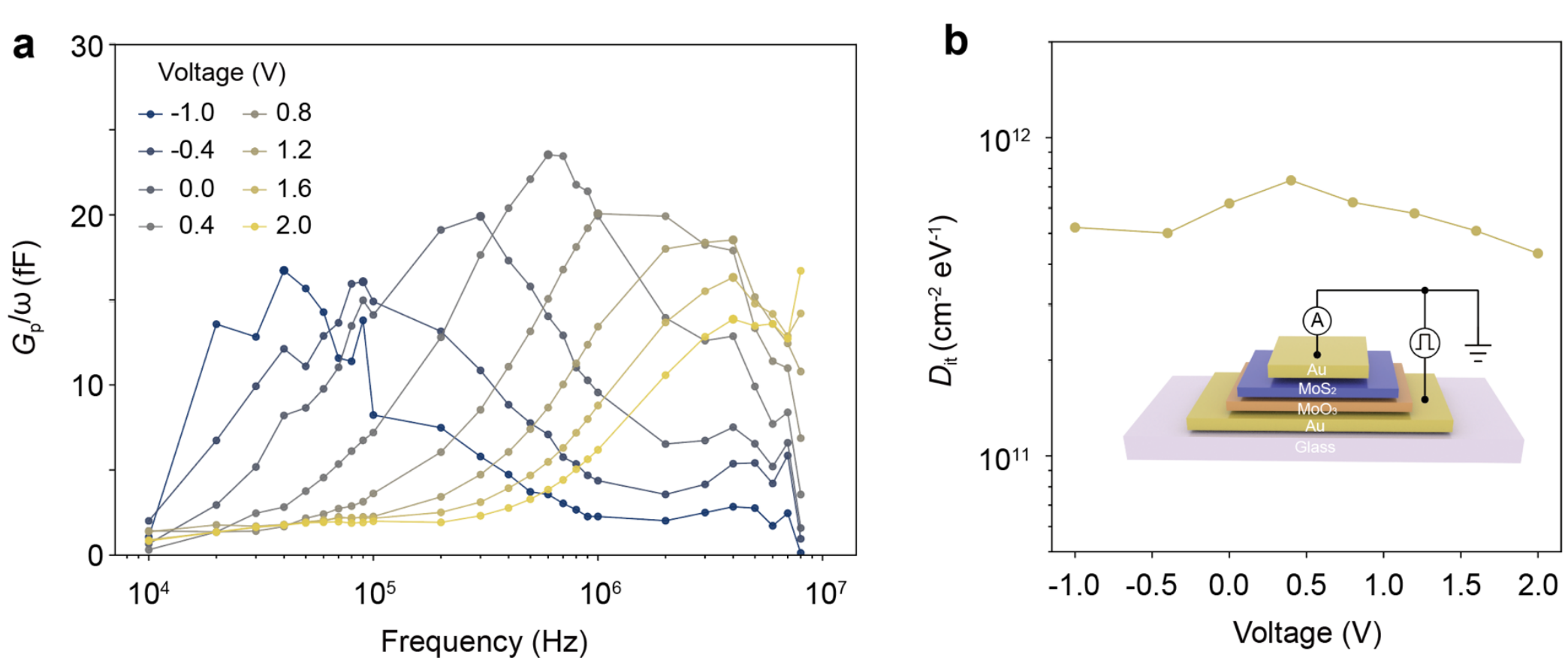

Supplementary Figure 48 Estimation of the interfacial trap density of a bottom gate device

using the *C-V* conductance method. a, G_p/ω versus the frequency of the device under different voltages. **b,** Extracted trap density from the peak values in (**a**) as a function of voltage.

To obtain the interficial trap density in the top-gated device, we further fabricated the MoS_2-MoO_3-Au device with a MoS_2 layer directly exfoliated on a sapphire substrate and measured D_{it} using the charge pumping method and $1/f$ method.

Charge pumping method. The charge pumping method has been adapted to 2D TMD FETs and probes interfacial traps through the periodic capture and emission of carriers during gate-pulse cycling[33]. In our measurements, the source and drain were grounded, while a DC offset together with a square-wave gate pulse was applied to the gate electrode, and the resulting current at the source terminal was monitored, as illustrated in Supplementary Figure 49. In this method, the gate pulse periodically sweeps the channel Fermi level across the interfacial trap states, leading to repeated carrier capture and emission during each pulse cycle. The resulting average charge-pumping current I_{cp} is therefore directly related to the density of electrically active traps. The average charge-pumping current can be written as $I_{cp} = qAfN_{it}$, where q is the elementary charge, A is the device area, f is the pulse frequency, and N_{it} is the interface trap charge density involved in the pumping process. If the trap density is approximately uniform within the pulse-swept energy window ΔE, the effective interface trap density can be estimated as

$$D_{it} \approx \frac{1}{qA\Delta E}\frac{dI_{CP}}{df}$$

As shown in Supplementary Figure 49, I_{cp} increases linearly with frequency under fixed pulse conditions (V_{base} = -1V and V_{pp} = 1V), which is a characteristic signature of charge pumping. The fitted slope is about 8.1×10^{-15} A/Hz, confirming that the measured current mainly originates from periodic trap-assisted carrier exchange rather than simple leakage current. The dependence of I_{cp} on the V_{base} is shown in Supplementary Figure 49b for f = 0.5 MHz and V_{pp} = 1V. A clear charge-pumping window is observed, that is, the current is small at both low and high V_{base}, but reaches a maximum in an intermediate bias range. This indicates that only within this bias window does the pulse effectively sweep across the energy range where interfacial traps can actively participate in carrier capture and emission. The dependence of I_{cp} on the pulse amplitude is presented in Supplementary Figure 49c for f = 0.5 MHz and V_{base} = -1V. As V_{pp} increases, I_{cp} first rises and then tends to saturate. This behavior indicates that a larger pulse amplitude probes a wider trap energy

window, while the saturation at high V_{pp} suggests that most accessible active traps have already been involved in the pumping process. Based on the processed charge-pumping data, we estimate an effective interface trap density of approximately ~2 × 10^{11} cm^{-2} eV^{-1}. Overall, the linear frequency dependence, the well-defined V_{base} window, and the V_{pp}-dependent activation behavior consistently confirm the presence of electrically active trap states at the MoO_3/MoS_2 interface.

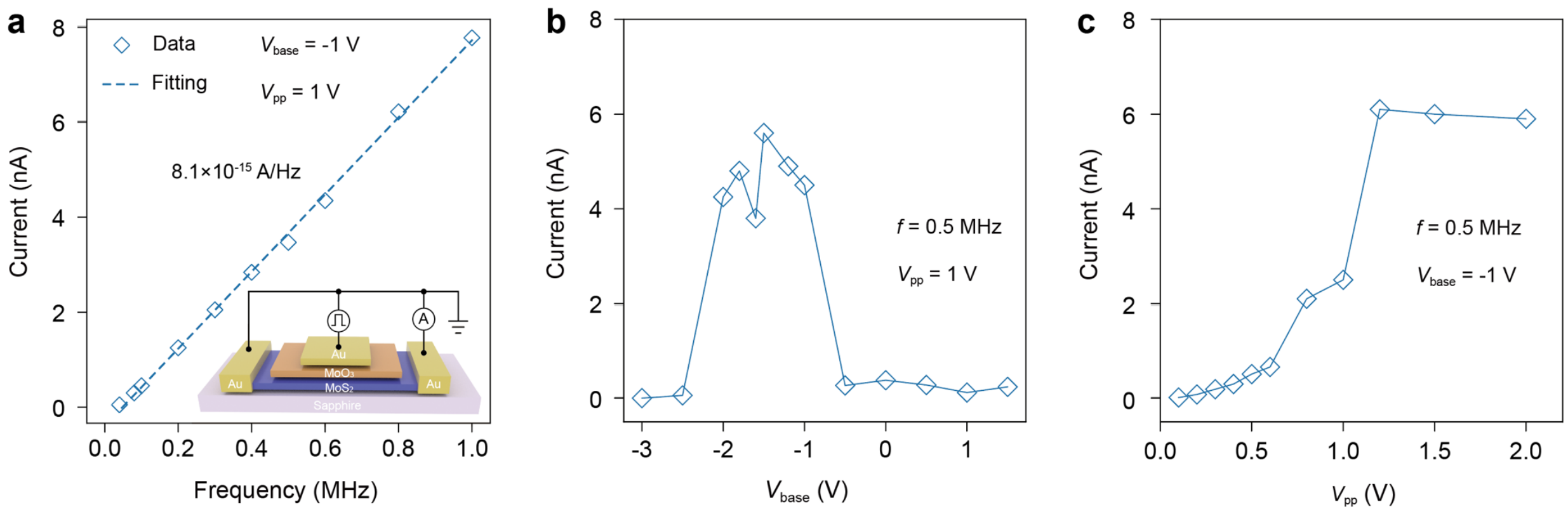

Supplementary Figure 49 Estimation of interfacial trap density of a top gate device using the charge pumping method. a, The charge pumping current as a function of frequency, inset is the basic measurement circuit. **b,** The charge pumping current as a function of base voltage. **c,** The charge pumping current as a function of pulse amplitude.

Low-frequency 1/*f* noise measurements. In addition, using the same bottom-gate MoS_2 FET platform, we independently evaluated the trap density by low-frequency 1/*f* noise measurements. In this method, the source and drain were grounded, and the current-noise spectra were measured under different gate voltages. The D_{it} could be extracted from the following equation:

$$D_{\mathrm{it}} = \frac{S_I\, W L C_{\mathrm{ox}}^2 f}{g_{\mathrm{m}}^2 q^2 k_{\mathrm{B}} T}$$

Where S_I is the spectral density, W and L are the width and the length of the channel, C_{ox} is the gate capacitance per unit area, g_m is the gate transconductance, q is the electron elementary charge, k_B is the Boltzmann constant, and T is the temperature. The obtained noise spectra remain similar at different gate biases and exhibit good linearity in the low-frequency regime (Supplementary Figure 50a), consistent with 1/*f* behavior. According to the equation, the interfacial trap density extracted from the noise data and the transfer curve is approximately ~1.5 × 10^{11} cm^{-2} eV^{-1}, which is in good agreement with the value obtained from charge pumping. And the D_{it} value almost shows no obvious difference in the low frequency range as shown in Supplementary Figure 50c.

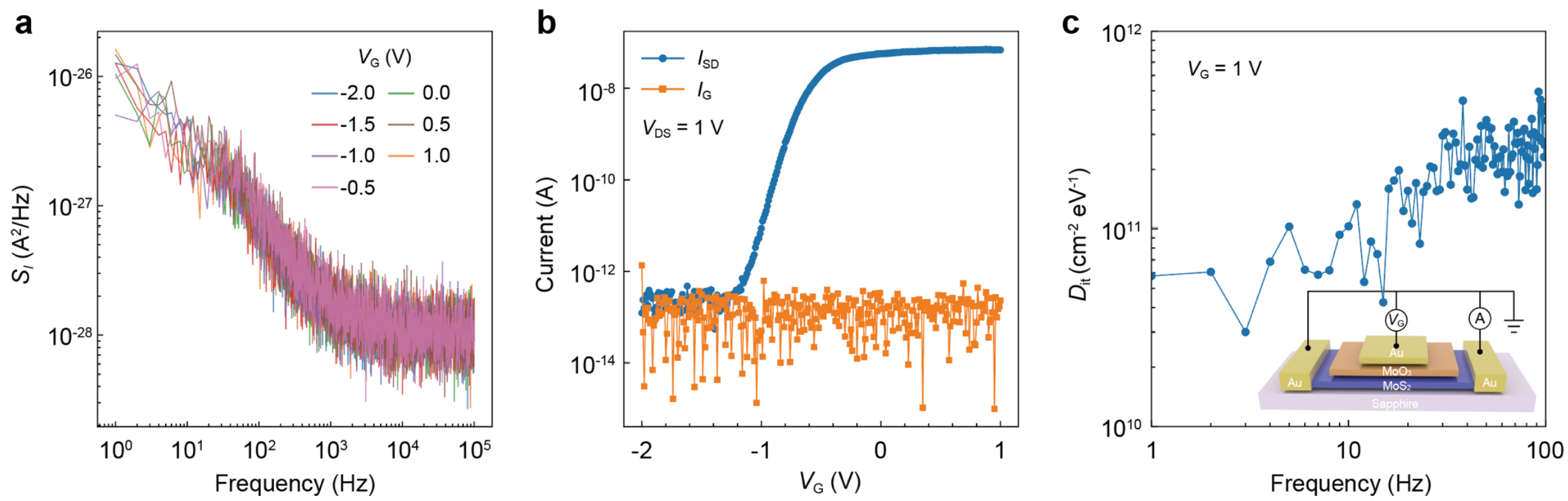

Supplementary Figure 50 Estimation of interfacial trap density of a top gate device using the 1/*f* method. a, Noise spectra as a function of frequency with different gate voltage at V_{DS} = 1 V. **b,** The source-drain current and gate leakage current as a function of gate voltage. **c,** Calculated interfacial trap density at V_G = 1 V, the interfacial trap density exhibits no obvious difference at low frequency.

Overall, the value of D_{it} is ~2 × 10^{11} cm^{-2} eV^{-1} from the charge pumping method, ~1.5 × 10^{11} cm^{-2} eV^{-1} from the 1/*f* method. By contrast, we obtained a larger value of D_{it} (~5 × 10^{11} cm^{-2} eV^{-1}) from the vertical *C*-*V* conductance structure in the Au-MoO_3-MoS_2-Au bottom-gate device structure, which is likely associated with the additional transfer step in the fabrication process. In this process, MoS_2 was transferred from PDMS to the MoO_3/Au bottom stack, which can introduce additional interfacial contamination and local defects, leading to a higher effective trap density. This is consistent with previous reports showing that transfer-induced residues, wrinkles, and contamination can substantially affect the interfacial quality and electronic properties of 2D-material devices[34].

Supplementary Note 6 Potential of MoO_3 in 2D light-emitting diodes

Here, we used monolayer WS_2 as the emission material to demonstrate the application of MoO_3 due to the relatively higher quantum yield of WS_2 compared to other TMDs. MoO_3 and WS_2 were dry-transferred step by step onto the graphene layer on the SiO_2/Si substrate to form the graphene-MoO_3-WS_2 heterostructure, followed by transferring gold electrodes to the heterostructure. The schematic of the device is shown in Supplementary Figure 51a. The graphene layer here acted as the gate electrode, and MoO_3 with a thickness of 10.4 nm (Supplementary Figure 51b, c) served as an insulating layer for applying an alternating-current (AC) gate voltage (square wave) to the monolayer WS_2. The AC voltage through the MoO_3 layer leads to an alternate injection of electrons and holes into the WS_2 layer, resulting in electroluminescence (EL) emission around the electrodes[35,36]. The light emission is called transient EL, as it is only created during the transition of the AC voltage. The characterization of photoluminescence (PL) in Supplementary Figure 51d, e indicates the uniform and high-quality WS_2 film in the LED device, together with the Raman spectrum (Supplementary Figure 51f). Initially, we measured the PL spectra as a function of the direct-current (DC) gate voltage (Supplementary Figure 52a). MoO_3 shows great ability for the gate voltage (V_G) to tune the PL signals in terms of intensity and wavelength. At low V_G, the PL was dominated by the neutral exciton (2.02 eV). Upon applying negative voltages, the PL intensity peaked at approximately -1.1 V, showing the intrinsic n-doping behavior of monolayer WS_2. Subsequently, the PL intensity began to decrease due to the screening effect and the enhanced formation of trions. Positive V_G resulted in a monotonic decrease in PL intensity. The trion peak (X^-) at 1.97 eV appeared at approximately 1.0 V and eventually became dominant.

For EL emission, we swept the gate voltage from 0 V to 4.3 V at a fixed frequency of 1 MHz. In contrast to PL, which could be observed with low optical injection levels, EL emission was only observable when V_G exceeded a certain threshold voltage (V_{TH}) (Supplementary Figure 52b). The threshold voltage is approximately 2.6 V, and the corresponding energy is close to the electronic band gap of monolayer WS_2. The EL intensity increased with V_G, while the peak position remained constant and was at approximately 2.005 eV, indicating that the emission was attributed to both excitons and trions. To compare the LED performance using other dielectrics, especially 2D insulator h-BN, we fabricated the same transient structure with h-BN acting as the dielectric layer. The EL emission as a function of V_G is plotted in Supplementary Figure 52c. Similarly, EL emission was only observable when V_G exceeded a threshold voltage. The threshold voltage for h-BN was

approximately 4 V, significantly higher than V_{TH} for MoO_3. Here, we emphasize that the much lower threshold voltage was due to the larger dielectric constant of MoO_3 than h-BN. Additionally, the AC frequency was also adjusted to alter the charge carrier injection rate, and EL emission was observed at frequencies above 50 kHz. The device remained functional as the frequency increased up to 20 MHz (Supplementary Figure 52d). The external quantum efficiency (EQE) of the transient LED was also calculated using the reported method[35,36]. The EQE as a function of the generation rate is shown in Supplementary Figure 52e. The EQE decreased as the generation rate increased, similar to what we have seen in SiO_2 and Al_2O_3 devices. The EQE of the MoO_3 LED is comparable to that of the h-BN LED device. Supplementary Figure 52f summarizes the threshold voltage (V_{TH}) versus EQE of WS_2 LEDs using different structures and dielectrics. Our WS_2 transient LEDs exhibited competitive EQE and much lower V_{TH} than state-of-the-art WS_2 LEDs using other gate dielectrics, such as h-BN, SiO_2, and Al_2O_3.

While our current work emphasizes light-emitting applications, the observed advantages of MoO_3—particularly its high dielectric constant and compatibility with low-voltage operation—suggest its potential for dual-gate FETs once the fabrication challenges are resolved. We are still exploring dry-transfer methods and contact engineering to mitigate impurity incorporation. These efforts aim to enable future studies on MoO_3-based dual-gate architectures for high-performance electronics.

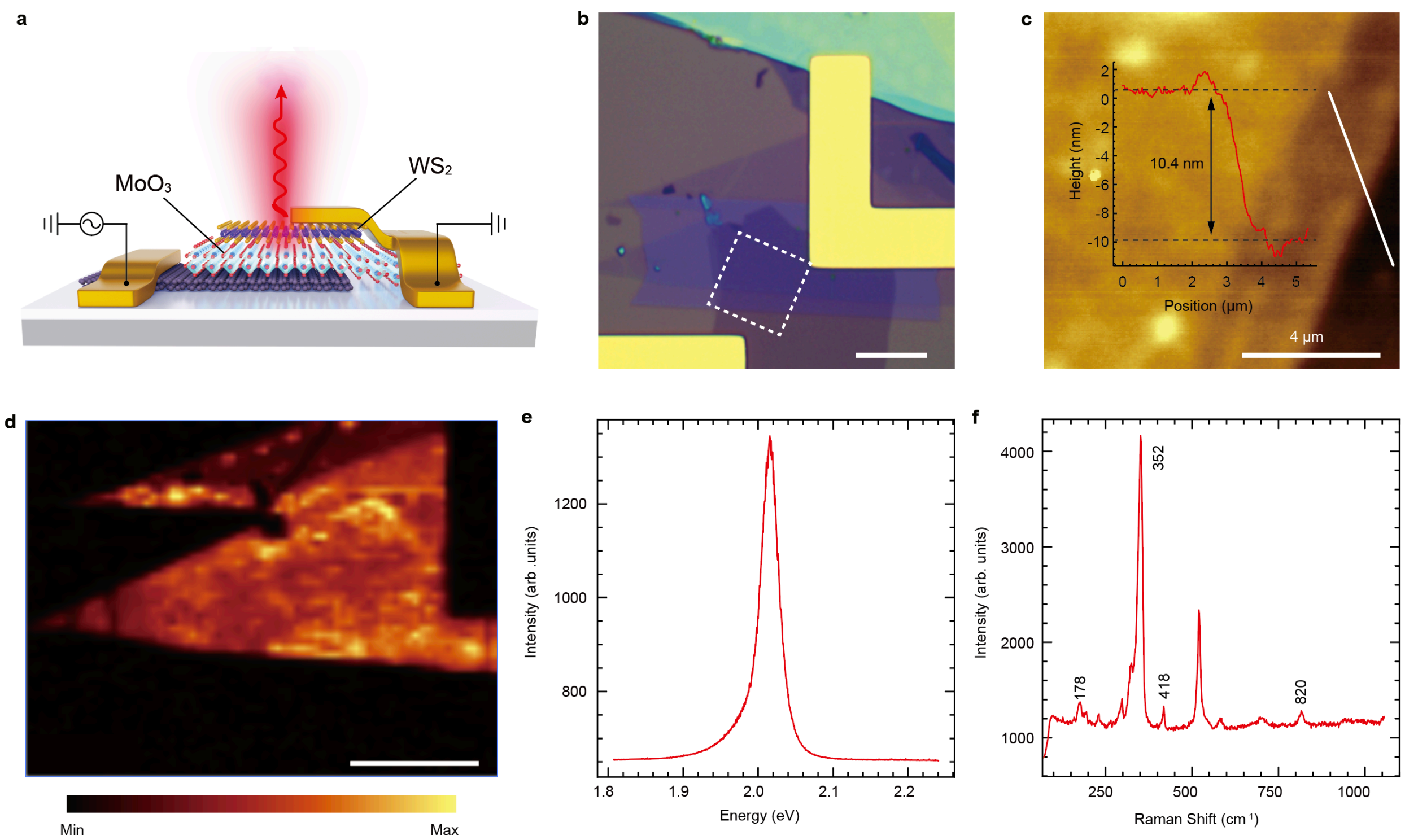

Supplementary Figure 51 AFM, Raman, and PL characterization of the LED device. **a**, Schematic illustration of the structure of a transient LED. **b**, The optical image of the LED device employing MoO_3 as the gate dielectric, scale bar is 10 µm. **c**, AFM image of the square area in b and corresponding height profile of the white line. **d**, PL mapping of the LED device, the integration is from 595 nm to 635 nm, scale bar is 10 µm. **e**, PL spectrum of the LED device, the signal was taken at the region of FLG/WS_2/MoO_3 heterostructure. The PL signal is from the WS_2 monolayer. **f**, Raman spectrum of the LED device, the signal was taken at the same point in the PL measurement. Raman peaks at 178 cm^{-1}, 352 cm^{-1}, 418 cm^{-1} and 820 cm^{-1} are from WS_2 monolayer, the peak at 820 cm^{-1} are from MoO_3.

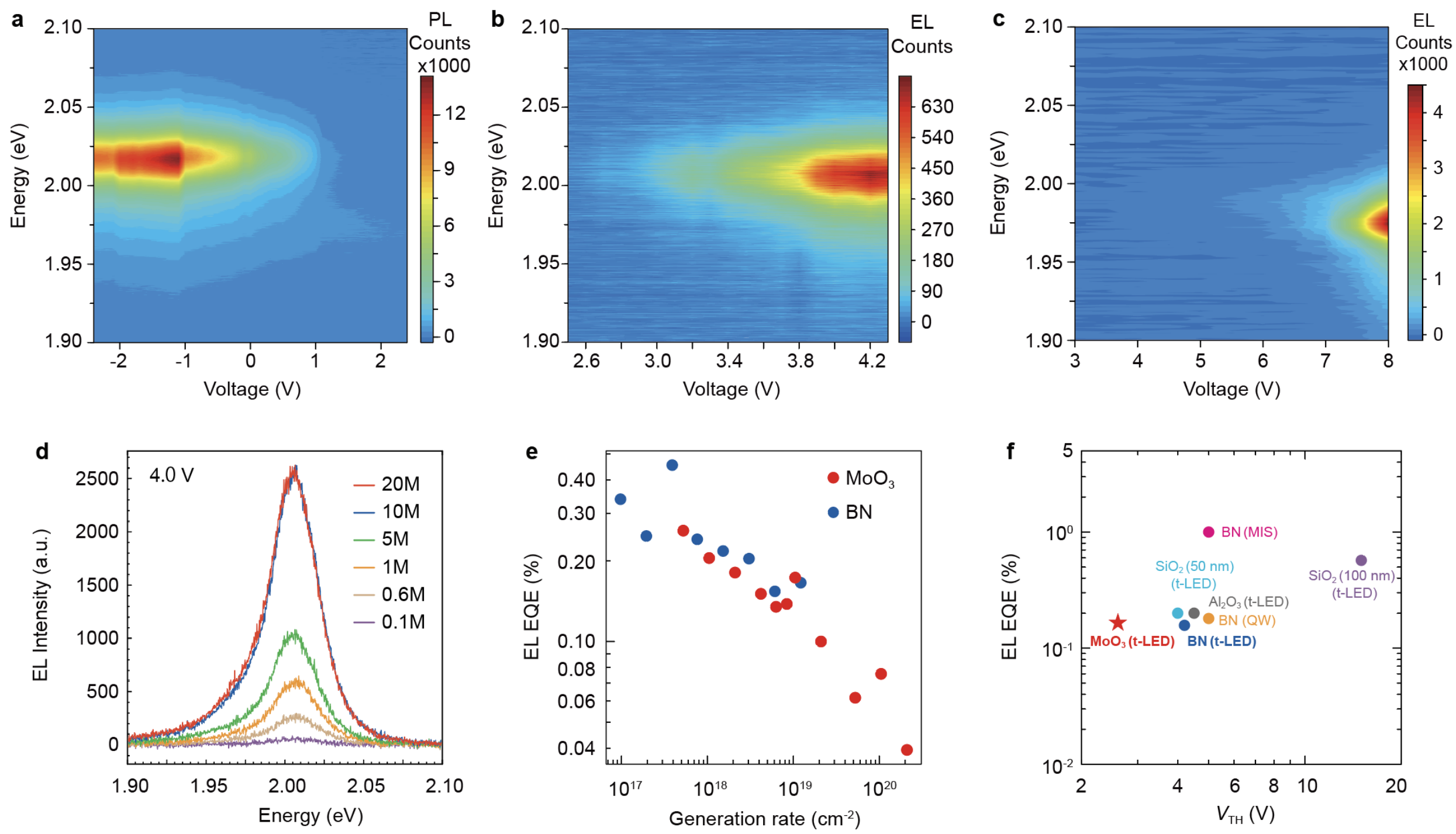

Supplementary Figure 52 Performance of the WS_2 transient LED using the MoO_3 dielectric. **a, b,** Contours of PL and EL intensity as a function of applied voltage and photon energy. **c,** Contour of the EL intensity of the transient LED using the h-BN dielectric. **d**, Frequency-dependent EL spectra. The EL peak positions are approximately at 2.005 eV, different from the PL peak at 2.105 eV, indicating the trion component. There is no shift in the peak position when varying the voltage and frequency. **e**, EQE of transient LEDs using MoO_3 and h-BN dielectrics under different generation rates. The voltage for MoO_3 and h-BN is 4 V and 8 V, respectively. The almost three-order range in generation rate is caused by the varied alternate frequency. **f,** State-of-the-art EQE and V_{TH} of WS_2 LEDs with different dielectrics and structures[35,37–39], 'MIS', 'QW', and 't' stand for metal-insulator-semiconductor, quantum well, and transient, respectively. Our results are labeled with a red star.

Supplementary References

1. Borgschulte, A. *et al.* Hydrogen reduction of molybdenum oxide at room temperature. *Sci Rep* **7**, 40761 (2017).
2. Guha, P. *et al.* Defect-Engineered MoO2 Nanostructures as an Efficient Electrocatalyst for Oxygen Evolution Reaction. *ACS Appl. Energy Mater.* **3**, 5208–5218 (2020).
3. McPherson, J., Kim, J., Shanware, A., Mogul, H. & Rodriguez, J. Proposed universal relationship between dielectric breakdown and dielectric constant. in *Digest. International Electron Devices Meeting,* 633–636 (2002). doi:10.1109/IEDM.2002.1175919.
4. Huang, J.-K. *et al.* High-κ perovskite membranes as insulators for two-dimensional transistors. *Nature* **605**, 262–267 (2022).
5. Liu, K. *et al.* A wafer-scale van der Waals dielectric made from an inorganic molecular crystal film. *Nat. Electron.* **4**, 906–913 (2021).
6. Li, T. *et al.* A native oxide high-κ gate dielectric for two-dimensional electronics. *Nat. Electron.* **3**, 473–478 (2020).
7. Kim, H.-S., Campbell, S. A. & Gilmer, C. Charge trapping and degradation in high-permittivity TiO_2 dielectric films. *IEEE Electron Device Lett.* **18**, 465–467 (1997).
8. Li, T. *et al.* Epitaxial growth of wafer-scale molybdenum disulfide semiconductor single crystals on sapphire. *Nat. Nanotechnol.* **16**, 1201–1207 (2021).
9. Li, X. *et al.* Controllable growth of MoO_3 dielectrics with sub-1 nm equivalent oxide thickness for 2D electronics. *Nat. Commun.* **16**, 6758 (2025).
10. Holler, B. A., Crowley, K., Berger, M.-H. & Gao, X. P. A. 2D Semiconductor Transistors with Van der Waals Oxide MoO_3 as Integrated High-κ Gate Dielectric. *Adv. Electron. Mater.* **6**, 2000635 (2020).

11. Kim, J. H. *et al.* Thickness-Insensitive Properties of α-MoO_3 Nanosheets by Weak Interlayer Coupling. *Nano Lett.* **19**, 8868–8876 (2019).

12. Zhu, Y. *et al.* Synthesis of Large-Sized van der Waals Layered MoO3 Single Crystals with Improved Dielectric Performance. *Precision Chemistry* **2**, 406–413 (2024).

13. Gu, M., Kim, T., Lee, D., Park, J. & Kim, T. Dielectric Constant of a Single MoO3 Nanostructure. *Nano Lett.* **25**, 1436–1442 (2025).

14. Hong, Y. *et al.* Thinner 2D α-MoO3 makes setting up memristors easier. *Journal of Materiomics* **10**, 1279–1289 (2024).

15. Wang, J. *et al.* High-κ van der Waals Oxide MoO3 as Efficient Gate Dielectric for MoS2 Field-Effect Transistors. *Materials* **15**, 5859 (2022).

16. Parmendu Kant, K. & Srivastava, R. Dielectric Permittivity and Breakdown Strength of Molybdenum Trioxide Films. *J. Phys. Soc. Jpn.* **39**, 1316–1318 (1975).

17. Deb, S. K. & Chopoorian, J. A. Optical Properties and Color-Center Formation in Thin Films of Molybdenum Trioxide. *J. Appl. Phys.* **37**, 4818–4825 (1966).

18. Dai, T., Ren, Y., Qian, L. & Liu, X. Characterization of Molybdenum Oxide Thin Films Grown by Atomic Layer Deposition. *J. Electron. Mater.* **47**, 6709–6715 (2018).

19. Das, T., Tosoni, S. & Pacchioni, G. Structural and electronic properties of bulk and ultrathin layers of V2O5 and MoO3. *Computational Materials Science* **163**, 230–240 (2019).

20. Lajaunie, L., Boucher, F., Dessapt, R. & Moreau, P. Strong anisotropic influence of local-field effects on the dielectric response of α-MoO3. *Phys. Rev. B* **88**, 115141 (2013).

21. Zhang, Z. *et al.* Highly Selective Synthesis of Monolayer or Bilayer WSe2 Single Crystals by Pre-annealing the Solid Precursor. *Chem. Mater.* **33**, 1307–1313 (2021).

22. Robertson, J. & Wallace, R. M. High-K materials and metal gates for CMOS applications.

Mater. Sci. Eng. R Rep. **88**, 1–41 (2015).

23. Sharma, P. K., Gupta, N. & Dankov, P. I. Characterization of polydimethylsiloxane (PDMS) as a wearable antenna substrate using resonance and planar structure methods. *AEU - International Journal of Electronics and Communications* **127**, 153455 (2020).
24. Dröscher, S. *et al.* Quantum capacitance and density of states of graphene. *Phys. Scr.* **T146**, 014009 (2012).
25. Xia, J., Chen, F., Li, J. & Tao, N. Measurement of the quantum capacitance of graphene. *Nat. Nanotechnol.* **4**, 505–509 (2009).
26. Padovani, A., La Torraca, P., Strand, J., Larcher, L. & Shluger, A. L. Dielectric breakdown of oxide films in electronic devices. *Nat. Rev. Mater.* **9**, 607–627 (2024).
27. Ahn, J.-H., Parkin, W. M., Naylor, C. H., Johnson, A. T. C. & Drndić, M. Ambient effects on electrical characteristics of CVD-grown monolayer MoS2 field-effect transistors. *Sci. Rep.* **7**, 4075 (2017).
28. Wang, L. *et al.* A general one-step plug-and-probe approach to top-gated transistors for rapidly probing delicate electronic materials. *Nat. Nanotechnol.* **17**, 1206–1213 (2022).
29. Groeseneken, G., Maes, H. E., Beltran, N. & De Keersmaecker, R. F. A reliable approach to charge-pumping measurements in MOS transistors. *IEEE Trans. Electron Devices* **31**, 42–53 (1984).
30. Zeng, D. *et al.* Single-crystalline metal-oxide dielectrics for top-gate 2D transistors. *Nature* **632**, 788–794 (2024).
31. Lu, Z. *et al.* Wafer-scale high-κ dielectrics for two-dimensional circuits via van der Waals integration. *Nat. Commun.* **14**, 2340 (2023).
32. Nicollian, E. H. & Brews, J. R. *MOS (Metal Oxide Semiconductor) Physics and Technology*.

(John Wiley & Sons, 2002).

33. Taniguchi, K., Fang, N. & Nagashio, K. Direct observation of electron capture and emission processes by the time domain charge pumping measurement of MoS2 FET. *Appl. Phys. Lett.* **113**, 133505 (2018).

34. Jain, A. *et al.* One-Dimensional Edge Contacts to a Monolayer Semiconductor. *Nano Lett.* **19**, 6914–6923 (2019).

35. Lien, D.-H. *et al.* Large-area and bright pulsed electroluminescence in monolayer semiconductors. *Nat. Commun.* **9**, 1229 (2018).

36. Wang, S. *et al.* Light-emitting diodes based on intercalated transition metal dichalcogenides with suppressed efficiency roll-off at high generation rates. *Nat. Electron.* **8**, 56–65 (2025).

37. Hou, L., Zhang, Q., Shautsova, V. & Warner, J. H. Operational Limits and Failure Mechanisms in All-2D van der Waals Vertical Heterostructure Devices with Long-Lived Persistent Electroluminescence. *ACS Nano* **14**, 15533–15543 (2020).

38. Wang, S. *et al.* Efficient Carrier-to-Exciton Conversion in Field Emission Tunnel Diodes Based on MIS-Type van der Waals Heterostack. *Nano Lett.* **17**, 5156–5162 (2017).

39. Cho, J. *et al.* Centimeter-Scale and Visible Wavelength Monolayer Light-Emitting Devices. *Adv. Funct. Mater.* **30**, 1907941 (2020).
