# Supplementary material for: Large-area two-dimensional MoO3 as a high-κ dielectric for van der Waals integration
Source: Nat Commun. 2026 Jul 7;17:8371. doi: 10.1038/s41467-026-75196-1 (PMC13473665; doi:10.1038/s41467-026-75196-1)
Supplement: Supplementary file 2 [file 41467_2026_75196_MOESM2_ESM.pdf]

nature portfolio

Peer Review File

Large-area two-dimensional MoO_3 as a high-κ dielectric for van der Waals integration

Corresponding Author: Professor Junpeng Lu

This file contains all reviewer reports in order by version, followed by all author rebuttals in order by version.

Version 0:

Reviewer comments:

Reviewer #1

(Remarks to the Author)
This paper by Zhenliang Hu et al reported a facile face-to-face evaporation and fast-cooling method to grow several-nm-thick MoO3 film with a large domain size. Compared to previously reported work (Chem. Mater. 2016, 28, 11, 4042–4051), this work discovered the important role of fast-cooling rate to increase the domain size of 2D films. As to me, this is a very intersting phonomenon, and is also very imporant and instructive to the synthesis of large domain-size 2D materials, even though MoO3 has already been demonstrated as the high-κ dielectrics by several previous papers, such as Nat Commun 16, 6758 (2025). Overall, the manuscript presents a comprehensive and well-organized study that includes material growth, dielectric characterization, and device integration. Thus, I recommend its publication in Nature Communications if the authors can address the following questions:
- One of the most imporant discoveries in this paper is the imporant role of fast-cooling to increase the domain size of 2D materials. Can this fast-cooling method be applied to increase the domain size of other 2D materials?

- The bandgap of the material is 3.6 eV, which is smaller than hBN. A brief discussion comparing the trade-off between dielectric constant and bandgap of MoO3 versus other vdW dielectrics would be better.
- “consistent with the bandgap size of 3.13 eV calculated via density functional theory (DFT)”. Please state the function as well. In the method section you mentioned PBE and HSE, which one was used to calculate Eg, better to be clear.
-Figure 2g regarding the leakage current of MoO3 is confusing. Suggest the authors the present the data with only single-scan I-V curves, rather than dual-sweep I-V curves.
- The surface roughness and interfacial flatness matters a lot for the vdW intergration of dielectrics. The authors claim that the roughness below or approximately 0.1 nm across 16 different sampling areas (Fig. S3). However, it seems the surface is absolutely not clean with a lot of contaminations ontop. Besides, the as-transferred MoO3 film also has a very rough surface. Can these pariticles and wrinkles be avoided? Suggest the authors to re-evaluate their surface roughensss and interfacial flatness of MoO3 by AFM.
- What is the test condition of the stability test? It would be better to give some results on the long-term stability of MoO3 dielectric under ambient conditions.
- It would be better to provide an optical image of the CMOS inverter.

Reviewer #2

(Remarks to the Author)
This manuscript reports the growth of millimeter-scale single-crystalline α-MoO3 via a rapid-cooling–induced van der Waals epitaxy strategy on mica, and demonstrates its use as an ultra-thin, high-dielectric constant (κ) vdW dielectric for 2D electronics. The authors achieve atomically flat MoO3 with EOT down to ~0.42 nm, low leakage, high breakdown fields, near-ideal subthreshold swing in MoS2 FETs, and a functional 2D CMOS inverter.

The work addresses a central bottleneck in 2D electronics, namely the lack of scalable, high-κ dielectrics that preserve clean vdW interfaces. While the utilization of MoO3 as the vdW dielectric is nothing new, the reported growth concept—kinetically stabilizing a vdW-epitaxial oxide phase through rapid cooling—is original and potentially generalizable to other layered oxides. Accordingly, I expect the study will be of interest to the experimentalists in the field and suitable for Nature Communications, provided that several key issues are clarified and some claims are more carefully framed. My criticisms are

as follows:

1. The authors claim the achievement of an Equivalent Oxide Thickness (EOT) of ~ 0.42 nm (physical thickness ~ 2.0 nm). However, the critical performance metrics—specifically leakage current and reliability—appear to be derived from significantly thicker samples. Specifically, the leakage current density meeting the ITRS low-power limit (10^-3 A cm^-2) is shown for an EOT of 0.87 nm (4.1 nm physical), not the 0.42 nm sample. Similarly, the breakdown field statistics (E_0 ~ 7.0 MV cm^-1) are presented for a ~7.1 nm thick film, again not the 0.42 nm sample. This is my major concern, and the authors should provide leakage and breakdown statistics specifically for devices near the minimum EOT.

2. Regarding the claim on the "low density of trap states", the authors in fact relied on indirect evidence—Subthreshold Swing (SS) and theoretical DFT binding energies. This contradicts the discussion on the device arrays, where the authors explicitly attribute the low field-effect mobility.
Please provide a direct quantitative measurement of Dit (e.g., using frequency-dependent C-V conductance methods, charge pumping, or transient techniques adapted to 2D systems).

3. A central claim of the paper is the "scalability" of the MoO3 integration. However, the data provided for the device arrays suggests significant challenges that are not fully quantified. The statistical distribution of the array devices shows a clear degradation in performance (SS ~ 70–110 mV dec^-1 and On/Off ratios of 10^6-10^7) compared to the single champion device. They should provide a yield map of the fabricated array (showing functional vs. non-functional devices) and explicitly state the percentage yield. Additionally, the criteria used to define a "successful" device need to be clarified.

4. Two different methods yield substantially different dielectric constants (κ ≈ 25 vs. κ ≈ 18), which are attributed to polymer residues or interfacial effects. While plausible, this discrepancy affects all derived quantities (EOT, charge density, SS interpretation). Since κ is central to the paper's claims, this issue affects the quantitative credibility of the dielectric performance. The authors should provide statistical distributions (mean, standard deviation, number of devices) for κ and EOT, and if possible include additional cross-validation (e.g., well-defined MIM capacitors or thickness-localized metrology). At minimum, clarify which κ value should be regarded as intrinsic.

5. The reported static power consumption of 0.2 nW at VDD = 1.0 V is highlighted as a key achievement. However, this figure lacks the necessary context. The champion single transistors exhibit Ioff ~ 10^-14 A, which should theoretically yield static power in the femtowatt range ($~ 0.01 pW). The reported 0.2 nW is roughly 20,000 times higher, implying significant leakage degradation in the inverter integration.
To resolve the issue, provide the IDD-VIN curves for the inverter at VDD = 1.0 V, and specify the channel dimensions (W/L) of the inverter transistors and normalize the static power (e.g., W/micro-m) to allow for proper benchmarking. Also, clarify the specific back-gate bias used to achieve the 0.2 nW figure.

Finally, as a minor comment on the vdW integration literature, note that there has been notable progress via transferring Hf- and Zr-based TMDs as precursors and oxidizing them. These references need to be cited:
- Luo, P. et al. Molybdenum disulfide transistors with enlarged van der Waals gaps at their dielectric interface via oxygen accumulation. Nat. Electron. 5, 849–858 (2022).
- Zhang, M. et al. Integration of ultrathin hafnium oxide with a clean van der Waals interface for two-dimensional sandwich heterostructure electronics. Nano Lett. 24, 1234–1240 (2024).
- Kang, T., et al. High-κ dielectric van der Waals integration on 2D semiconductors for three-dimensional complementary logic systems. Nat. Commun. 16, 11648 (2025).

Reviewer #3

(Remarks to the Author)
In this manuscript, Hu and co-workers reported the growth and integration of large-area crystalline MoO3 as a dielectric layer for two-dimensional electronic devices. The authors demonstrated large-area, uniform MoO3 films with atomically flat surfaces, a dielectric constant of approximately 18.4, and a low equivalent oxide thickness (EOT) ~0.42 nm, together with low leakage current densities and a high breakdown field ~7.1 MV cm^{-1}. MoS2 transistors based on MoO3 exhibited low subthreshold swing and high current on-off ratio. Importantly, the demonstration of the transistor array indicates the potential of large-area MoO3 as an alternative high-κ dielectric in integrated electronics based on 2D semiconductors. The topic of the research is of potential interest to researchers in the fields of 2D materials and nanoscale electronics. Therefore, I recommend its publication after the following issues have been fully addressed.
1. The device integration relies on multiple transfer steps, including transferring MoO3 from mica to SiO2 and the transfer of MoS2 and graphene, which may introduce interfacial defects. In this context, the authors are encouraged to have some discussion of further optimization methods. It would be useful to comment on whether the MoO3 growth can be extended to other substrates, such as Al2O3 or SiO2. Furthermore, is the in-situ growth of MoO3 on MoS2 feasible, which I think will greatly reduce the interfacial disorders?
2. This work reports a water-assisted transfer method to transfer MoO3 from mica to SiO2. What is the success rate of the transfer?
3. The oxygen vacancies in MoO3 will greatly affect its conductivity, and MoOx is usually considered as a hole transport layer or anode buffer layer in the perovskite device. Characterization of the oxygen stoichiometry and electrical conductivity of the MoO3 film would help to confirm its suitability as a dielectric.
4. What is the dark gap in the cross-sectional HAADF-STEM image in Fig. 3b, which is observed between the MoO3 layers and the monolayer MoS2. Please clarify whether this gap is intrinsic, related to sample preparation, or indicative of imperfect

interfacial contact.
5. What are the average values of the subthreshold swing (SS) and on/off current ratio for the FET device array? Providing the statistical averages (and preferably the standard deviation) in the main text would allow a clearer assessment of device uniformity and overall performance.
6. There is no color bar in Fig. 1f.
7. Better to indicate the EOT in Fig. 2g.
8. In Fig. S29, please clearly indicate which branch corresponds to the forward sweep and which corresponds to the backward sweep.
9. In Fig. S33, it would be nice to plot the hysteresis explicitly as a function of the sweep rate or the voltage step.

Version 1:

Reviewer comments:

Reviewer #1

(Remarks to the Author)
The authors have addressed my questions satisfactorily. The revised version has been improved greatly. I recommend its publication in Nature Communications after the minor textual modification was made.

Minor comment

1) The authors declared that "a low equivalent oxide thickness (EOT) ~0.42 nm (physical 48
thickness ~2.0 nm)" in the abstract. However, the EOT value was not supported by the direct capacitance measurement. To be more rigorous， suggest the authors remove the claim of 0.42-nm EOT in the Abstract.

Reviewer #3

(Remarks to the Author)
I think the authors has made great improvements on the manuscript and well addressed all of my concerns. Their responses to other reviewers' comments are also comprehensive and convincing. It can be accepted for publication.

Open Access This Peer Review File is licensed under a Creative Commons Attribution 4.0 International License, which permits use, sharing, adaptation, distribution and reproduction in any medium or format, as long as you give appropriate credit to the original author(s) and the source, provide a link to the Creative Commons license, and indicate if changes were made.
In cases where reviewers are anonymous, credit should be given to 'Anonymous Referee' and the source.
The images or other third party material in this Peer Review File are included in the article’s Creative Commons license, unless indicated otherwise in a credit line to the material. If material is not included in the article’s Creative Commons license and your intended use is not permitted by statutory regulation or exceeds the permitted use, you will need to obtain permission directly from the copyright holder.

To view a copy of this license, visit https://creativecommons.org/licenses/by/4.0/

Point-by-point response to reviewers' comments for manuscript ID: NCOMMS-25-102445

General response: We are grateful to all reviewers for recognizing the importance of our work. We value those comments and have addressed all the questions and concerns with substantial experiments in the revised manuscript. These comments indeed drove us to improve the quality of our manuscript. We sincerely hope the reviewers will feel satisfied with our response.

(Original comments are in orange, our responses are in black, and the corresponding revisions in the updated manuscript are highlighted in red.)

Reviewer #1 (Remarks to the Author):

This paper by Zhenliang Hu et al reported a facile face-to-face evaporation and fast-cooling method to grow several-nm-thick MoO3 film with a large domain size. Compared to previously reported work (Chem. Mater. 2016, 28, 11, 4042–4051), this work discovered the important role of fast-cooling rate to increase the domain size of 2D films. As to me, this is a very intersting phonomenon, and is also very imporant and instructive to the synthesis of large domain-size 2D materials, even though MoO3 has already been demonstrated as the high-κ dielectrics by several previous papers, such as Nat Commun 16, 6758 (2025). Overall, the manuscript presents a comprehensive and well-organized study that includes material growth, dielectric characterization, and device integration. Thus, I recommend its publication in Nature Communications if the authors can address the following questions:

Response: We sincerely thank you for the positive evaluation of the significance and recommendation of our work. We have performed extensive experiments to fully address your questions and concerns, and we hope that you will find our revised version of the manuscript satisfactory for publication. Below, please find our detailed response to your comments.

- One of the most imporant discoveries in this paper is the imporant role of fast-cooling to increase the domain size of 2D materials. Can this fast-cooling method be applied to increase the domain size of other 2D materials?

Response: Thank you for your great suggestions about the fast cooling method. Following your suggestions, we found that the fast-cooling method can be used to increase the domain size of transition metal dichalcogenides such as WSe_2.

WSe_2 was synthesized in a single-zone chemical vapor deposition (CVD) system using SiO_2/Si substrates. In the growth process, 5 g of precursor (WSe_2 powder) was used, the argon gas flow rate was maintained at 150 sccm, and the furnace temperature was increased to 1170 °C under atmospheric pressure. After the 2 min growth stage, the sample was subjected to a fast-cooling process. In this work, the fast-cooling method refers to immediately pulling the substrate out of the heating zone once the growth was completed, so that the sample rapidly left the high-temperature region and cooled down quickly. Switchable gas flow was applied to accelerate heat dissipation and remove residual reactants around the substrate[1]. The fast-cooling process and switched gas flow together help suppress continued deposition and secondary nucleation during the cooling stage, thereby preserving the as-grown WSe_2 domains. By contrast, in the conventional cooling process, the gas-flow direction was not switched after growth, and the substrate was kept in its original position until the furnace cooled naturally to room temperature, after which the substrate was taken out. Except for the cooling protocol, the precursor amount, substrate type, gas flow rate, growth temperature, and growth time were all kept the same.

As shown in Fig. R1, without this method, the WSe_2 domain size was approximately 20 μm. However, when the fast-cooling method was employed, the WSe_2 domain size increased to approximately 444 μm, which is approximately 20 times larger.

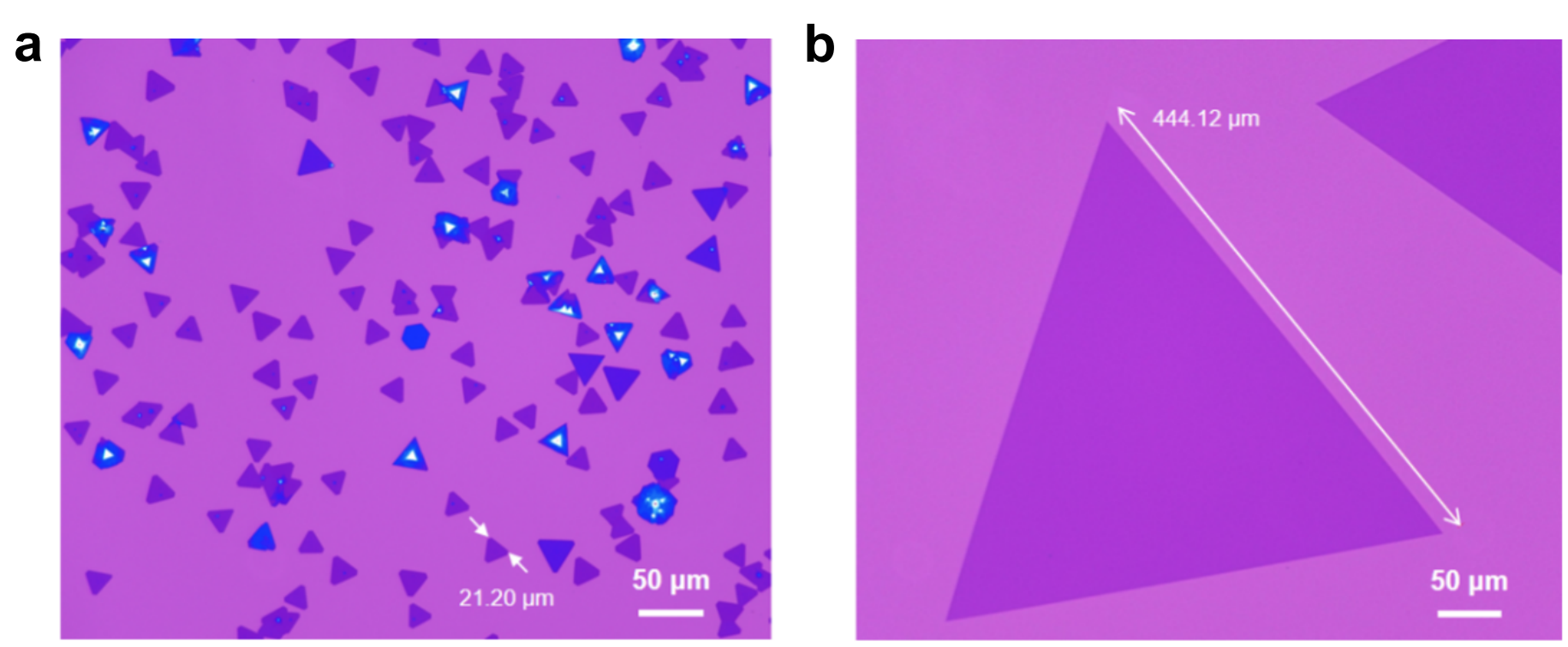

Fig. R1 The effect of the fast-cooling methods on the increase in WSe_2-domain size. a, The WSe_2 domains prepared without fast-cooling, **b,** The WSe_2 domains prepared using

the fast-cooling method. The white arrow indicates the characteristic domain size. Scale bar: 50 μm.

Changes made to the manuscript:

1. Line 142, "This method can also be applied to increase the domain size of transition metal dichalcogenides such as WSe_2 from 20 μm to 444.12 μm (Supplementary Note 1)."

2. Fig R1 has been added in the supplementary information as Note 1.

- The bandgap of the material is 3.6 eV, which is smaller than hBN. A brief discussion comparing the trade-off between dielectric constant and bandgap of MoO3 versus other vdW dielectrics would be better.

Response: Thank you for your comment about the bandgap and dielectric constant. The relationship between the dielectric constant and the bandgap is a fundamental trade-off in materials, for vdW dielectrics[2] and conventional oxide dielectrics[3,4]. A higher dielectric constant is usually associated with a smaller electronic bandgap, and a large bandgap is associated with a low dielectric constant. The ideal dielectric requires both a high dielectric constant to control charge efficiently and a wide bandgap to prevent the leakage current.

Although MoO_3's bandgap (~3.6 eV) is smaller than that of hBN and some other wide-bandgap vdW insulators, MoO_3 offers a substantially larger dielectric constant, which is beneficial for achieving lower EOT and stronger gate control. As for the leakage current, a smaller bandgap usually leads to increased leakage current. However, a large number of metal-insulator-metal (MIM) devices and FET devices have proved that the leakage current is comparable to that of other dielectric materials, as shown in Fig. 2g and 2h. These results indicate that MoO_3 provides a favorable balance between a high dielectric constant and insulating performance, making it a promising candidate for scaled two-dimensional electronic devices.

Changes made to the manuscript:

1. Line 182, "In addition, the absorption spectrum (Fig. S15) indicates that the bandgap of MoO_3 flake is approximately 3.60 eV, in reasonable agreement with the value of 3.13 eV obtained from density functional theory (DFT) calculations with the HSE06 hybrid functional (see Methods and Fig. S16). While the bandgap of MoO_3 is smaller than that of

h-BN and some other wide-bandgap dielectrics, its dielectric constant is significantly higher, as will be discussed later. This highlights an important trade-off for vdW dielectrics[29] and conventional oxide dielectrics[30], that is, larger-bandgap insulators generally provide weaker dielectric screening, whereas higher-κ dielectrics typically come with more moderate bandgaps."

- "consistent with the bandgap size of 3.13 eV calculated via density functional theory (DFT)". Please state the function as well. In the method section you mentioned PBE and HSE, which one was used to calculate Eg, better to be clear.

Response: We thank you for pointing this out. The description of the DFT-calculated bandgap in the original manuscript was not sufficiently explicit. In the revised manuscript, we have clarified that the bandgap value of 3.13 eV was calculated using the HSE06 hybrid functional, while the PBE functional was used for structural optimization. We have revised the relevant sentence in the main text accordingly and further clarified this point in the Methods section.

Changes made to the manuscript:

1. Line 506, we've corrected it as "To obtain an accurate electronic structure and bandgap of α-MoO_3, the Heyd-Scuseria-Ernzerhof (HSE06) hybrid functional was adopted."

2. Line 182, we've corrected it as "In addition, the absorption spectrum (Fig. S15) indicates that the bandgap of MoO_3 flake is approximately 3.60 eV, in reasonable agreement with the value of 3.13 eV obtained from density functional theory (DFT) calculations with the HSE06 hybrid functional (see Methods and Fig. S16)."

-Figure 2g regarding the leakage current of MoO3 is confusing. Suggest the authors the present the data with only single-scan I-V curves, rather than dual-sweep I-V curves.

Response: We appreciate your comment very much. To avoid confusion, we have updated Fig. 2g to present single-scan *I-V* curves only, instead of dual-sweep curves. In addition, to provide a more direct evaluation of the leakage current at the dielectric thickness relevant to device scaling, we further fabricated MoO_3-based MIM capacitors with a thickness corresponding to an EOT of 0.43 nm and measured their leakage current

characteristics. The results are presented in Fig. R2, which more clearly demonstrates the leakage performance of MoO_3 dielectric films.

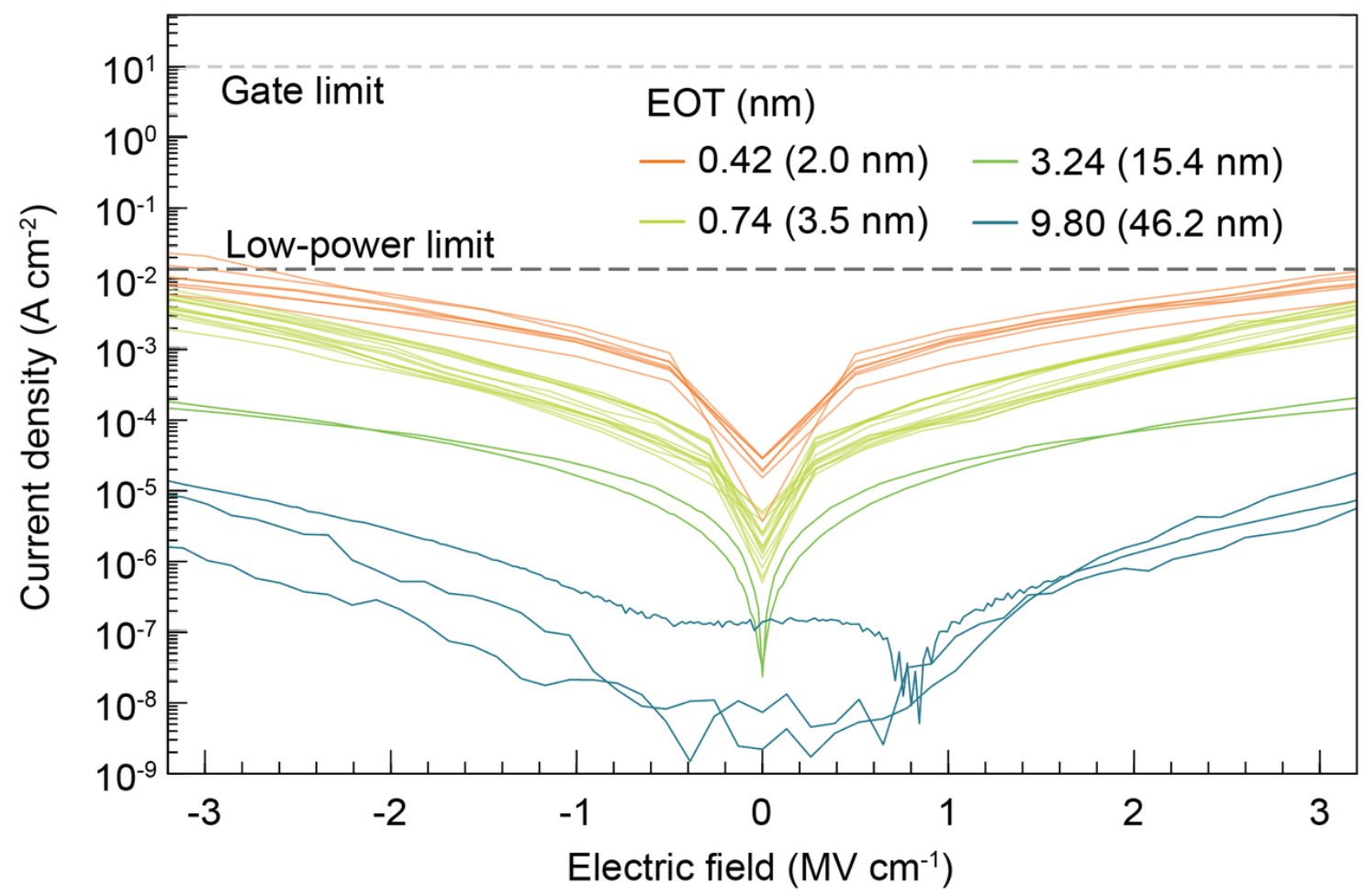

Fig. R2 Leakage current density of single-crystalline MoO_3 at different thicknesses as a function of applied electric fields.

Changes made to the manuscript:

1. Fig. 2g in the revised manuscript has been replaced by Fig. R2.

2. Line 278, "The leakage current for 2.0 nm MoO_3 (EOT = 0.43 nm) is lower than 10^{-2} A cm^{-2} at an electric field ~3 MV cm^{-1}, meeting the low-power logic limits (1.5×10^{-2} A cm^{-2}) set by ITRS (International Technology Roadmap for Semiconductors)[53]."

- The surface roughness and interfacial flatness matters a lot for the vdW intergration of dielectrics. The authors claim that the roughness below or approximately 0.1 nm across 16 different sampling areas (Fig. S3). However, it seems the surface is absolutely not clean with a lot of contaminations ontop. Besides, the as-transferred MoO3 film also has a very rough surface. Can these pariticles and wrinkles be avoided? Suggest the authors to re-evaluate their surface roughensss and interfacial flatness of MoO3 by AFM.

Response: Thank you for your important comments. We fully agree that the surface roughness and interfacial flatness are quite important for van der Waals integration of dielectric layers, and we appreciate the opportunity to clarify this point.

In the original manuscript, the reported roughness values were extracted from local AFM regions (10 μm × 10 μm in 40 μm × 40 μm) without obvious particles and bulk dusts. However, this could be misleading, because such locally selected regions do not fully

represent the overall surface morphology when particles are present on the surface. To avoid this confusion, in the revised manuscript, we have re-evaluated the roughness using the entire AFM scan area (~40 μm × 40 μm), and the results are shown below (Fig. R3). As expected, the extracted RMS roughness becomes higher, with most of them in the range of 1-2 nm. In regions containing dust or surface contaminants, the RMS roughness becomes significantly larger, as these extrinsic features introduce substantial local height fluctuations and therefore increase the measured overall roughness. We have revised the corresponding discussion accordingly to clearly distinguish the intrinsic local flatness from the overall surface roughness.

For the as-transferred MoO_3 films, we observed wrinkles and cracks. In practice, these features are difficult to completely avoid during the transfer of large-area ultrathin layered materials[5]. During our process optimization, we found that replacing the conventional thin PDMS stamp with a home-made thick PDMS stamp can greatly suppress the formation of crackles during transfer, although wrinkles remain (Fig. R4). In future work, we will continue optimizing the growth technique to reduce the occurrence of wrinkles and cracks.

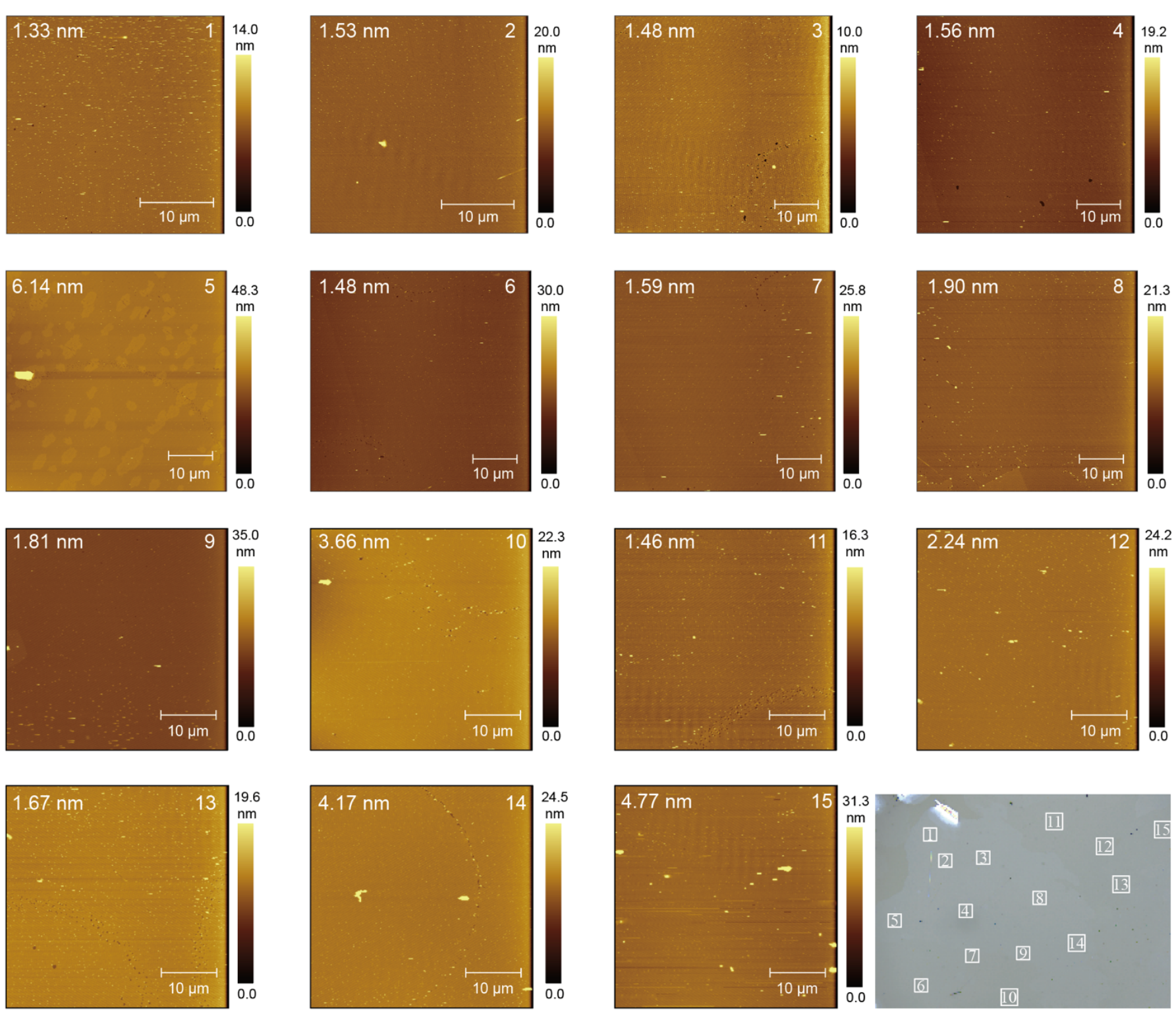

Fig. R3 Image of the revised Fig. S3.

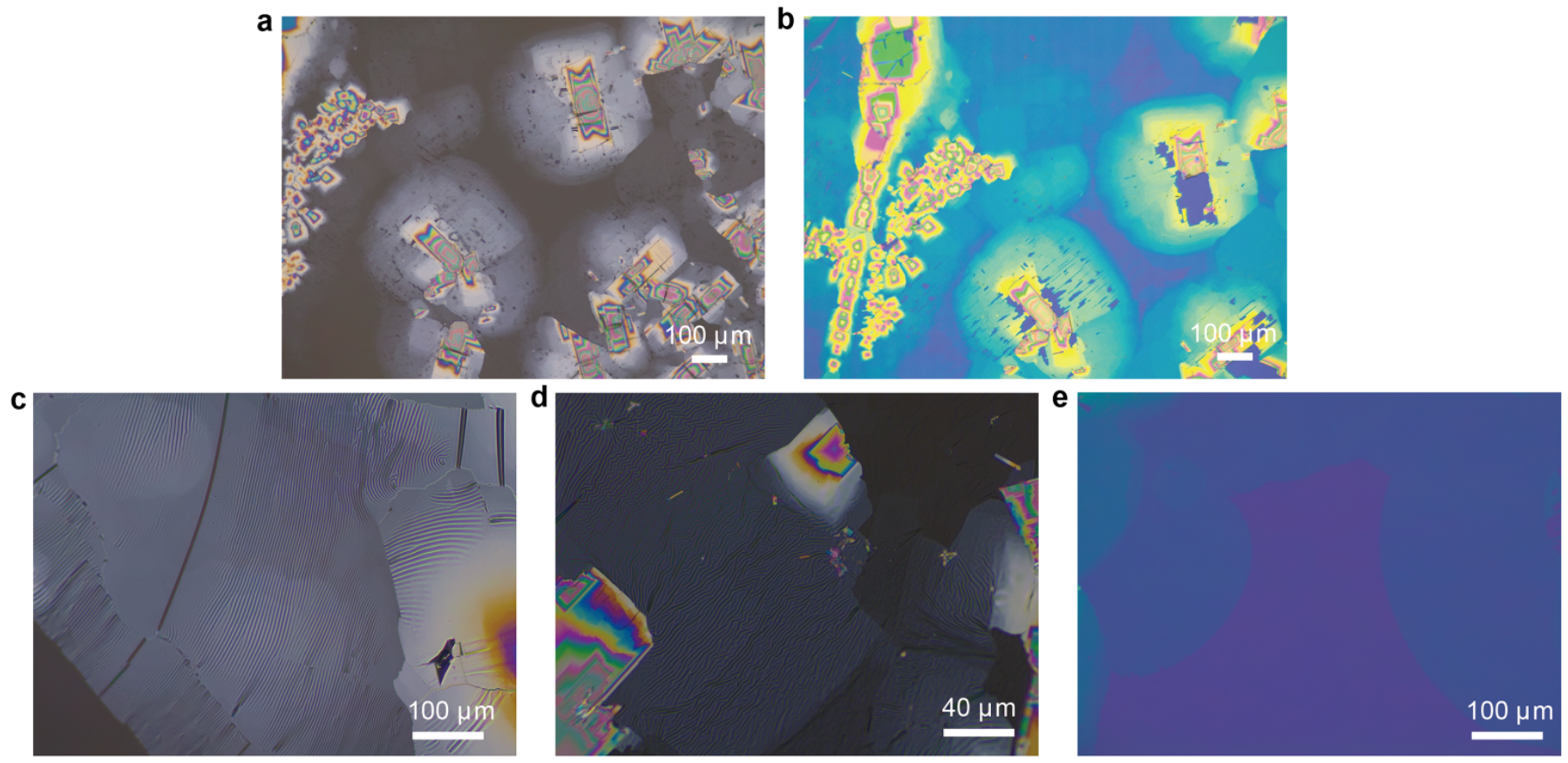

Fig. R4 Optimization of the transferred MoO_3. a, b, Optical images of the transferred MoO_3 on PDMS and SiO_2/Si substrate using the thin PDMS stamp. **c, d,** Optical images of the transferred MoO3 on PDMS substrate using the thick home-made PDMS stamp. **e,** The optical image of the transferred MoO3 on SiO_2/Si using the thick home-made PDMS stamp.

Changes made to the manuscript:

1. Line 149, "Over a relatively large scan area of 40 μm × 40 μm, the film exhibits an RMS roughness of 1-2 nm, indicating good large-area surface uniformity. In selected local regions, the roughness is even lower, reflecting the intrinsic local flatness and nanoscale thickness homogeneity of the film. These features are important for high-quality vdW integration and high-performance FETs."

2. Fig. S3 in the original manuscript (Fig. S4 in the revised version) has been replaced by Fig. R3.

3. Fig. S4 caption, "The extracted RMS roughness of most regions ranges from 1-2 nm in a scan area of 40 μm × 40 μm. In regions containing dust or surface contaminants, the RMS roughness becomes significantly larger, as these extrinsic features introduce substantial local height fluctuations and therefore increase the measured overall roughness."

- What is the test condition of the stability test? It would be better to give some results on the long-term stability of MoO3 dielectric under ambient conditions.

Response: We are grateful for your comments to improve the clarity. It is correct that the test conditions for the stability measurement should be clearly specified and that the long-term stability of the MoO_3 dielectric under ambient conditions is important.

In the revised manuscript, we have clarified that the stability test shown in Fig. 3h was carried out at room temperature in vacuum (~10^{-6} Torr) under a continuous gate bias of 1 V. We have revised the corresponding text and figure caption accordingly.

Following your suggestion, we further evaluated the stability of the MoO_3 device under ambient conditions. We measured a newly fabricated MoO_3 FET and measured the I_D-V_G curve under ambient conditions once every month. Noted that the device was stored under ambient conditions. There is no obvious change as shown in Fig. R5, indicating the good stability of the MoO_3 device during storage under ambient conditions. Additionally, we measured the device in ambient conditions under a continuous gate voltage. It shows that the current of the device experienced a slow degradation, which could be from the adsorption of ambient moisture and oxygen molecules[6].

These results indicate that the MoO_3 device maintains good stability not only during electrical operation under controlled vacuum conditions, but also during storage under ambient conditions.

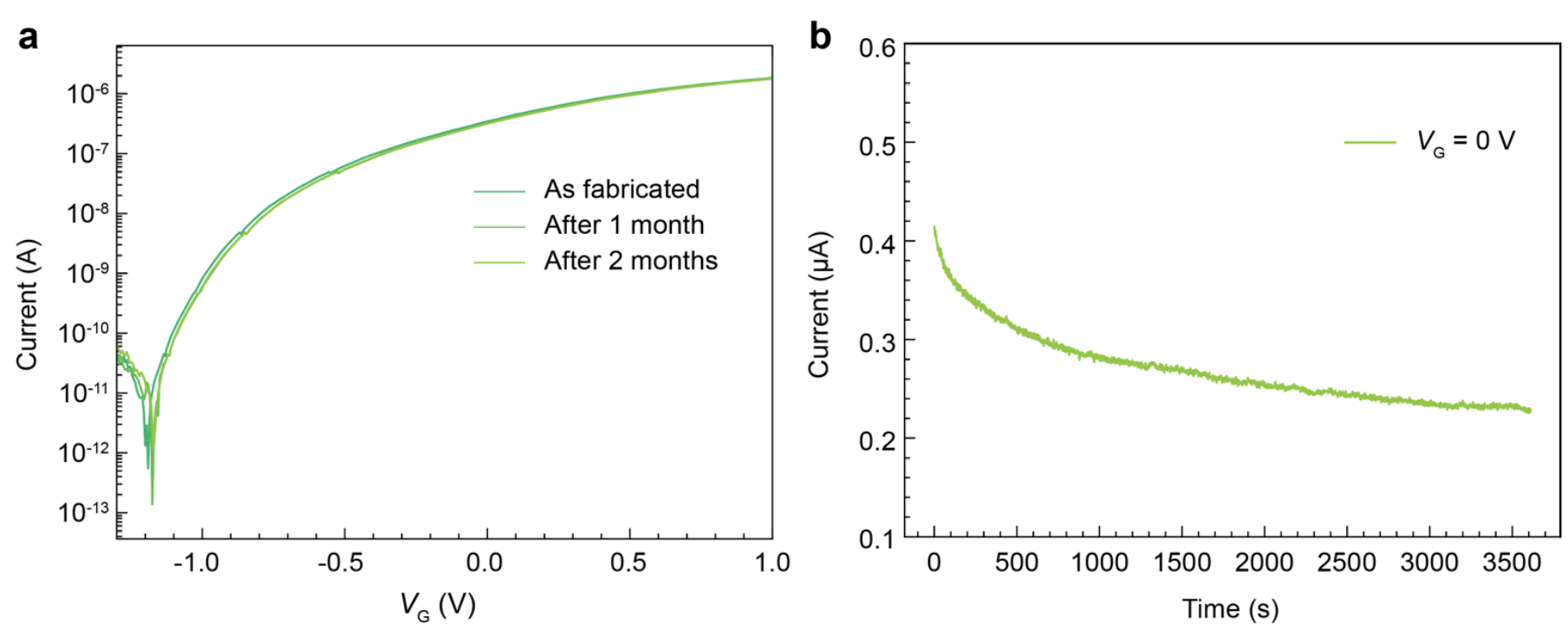

Fig. R5 Stability test of the MoO_3 device in ambient conditions. a, The transfer curves of the device as fabricated, after 1 month, and after 2 months. **b,** The operational stability of the device under a continuous gate voltage of 0 V for 1 hour in ambient conditions.

Changes made to the manuscript:

1. Line 341, "Besides, the transistor exhibited stable performance in vacuum at room temperature. The gate leakage current, on current, off current, and current on/off ratio were maintained nearly constant during the entire test, including the continuous and pulsed bias (Fig. 3h, Supplementary Note 4)."

2. Line 356, "The operational stability of the FETs was evaluated at room temperature in vacuum (~10^{-6} Torr) under a continuous gate voltage of 1 V for over 80000 seconds."

3. We’ve added the description of the test condition in the Supplementary Note 4.

4. Fig. R5 and the relative description have been added in the Supplementary Note 4.

- It would be better to provide an optical image of the CMOS inverter.

Response: We thank you for your valuable suggestion. In the original manuscript, the MoS_2 and WSe_2 devices were fabricated separately with the same device structure and similar MoO_3 thicknesses. To further confirm the integration feasibility of the MoS_2 and WSe_2 devices using the MoO_3 dielectric, we fabricated bottom-gate MoS_2 and WSe_2 devices on the same MoO_2 flake. The optical image of the integrated device, together with the measured output voltage as a function of input voltage for the CMOS inverter, is presented in Fig. R6.

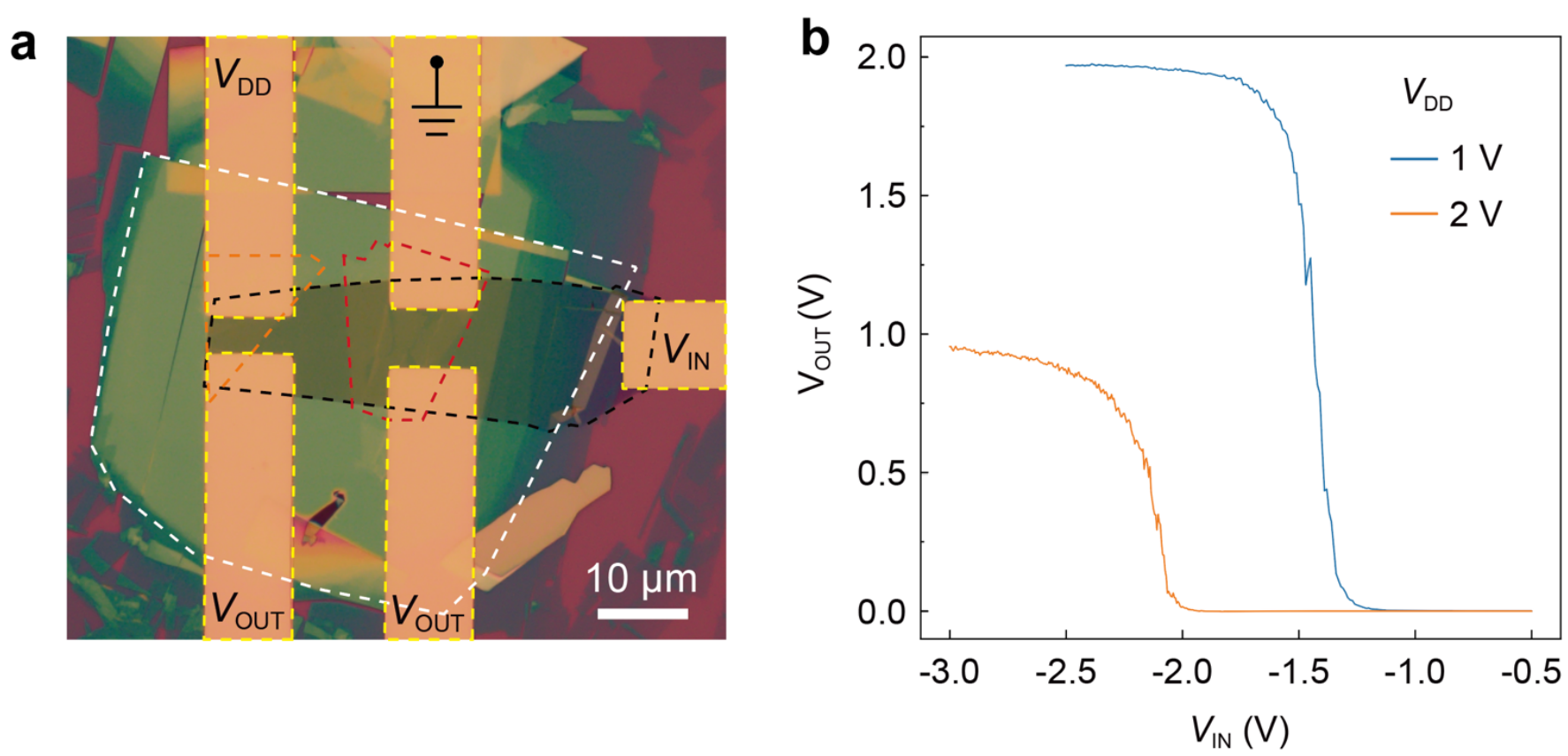

Fig. R6 Characterization of the inverter. a, Optical image of the inverter, with yellow, red, white, and dark lines representing WSe_2, MoS_2, MoO_3, and FLG gate, respectively. **b,** Measured output voltage as a function of input voltage of the CMOS inverter at V_{DD} = 1.0 V and 2.0 V.

Reviewer #2 (Remarks to the Author):

This manuscript reports the growth of millimeter-scale single-crystalline α-MoO3 via a rapid-cooling–induced van der Waals epitaxy strategy on mica, and demonstrates its use as an ultra-thin, high-dielectric constant (κ) vdW dielectric for 2D electronics. The authors achieve atomically flat MoO3 with EOT down to ~0.42 nm, low leakage, high breakdown fields, near-ideal subthreshold swing in MoS2 FETs, and a functional 2D CMOS inverter. The work addresses a central bottleneck in 2D electronics, namely the lack of scalable, high-κ dielectrics that preserve clean vdW interfaces. While the utilization of MoO3 as the vdW dielectric is nothing new, the reported growth concept—kinetically stabilizing a vdW-epitaxial oxide phase through rapid cooling—is original and potentially generalizable to other layered oxides. Accordingly, I expect the study will be of interest to the experimentalists in the field and suitable for Nature Communications, provided that several key issues are clarified and some claims are more carefully framed. My criticisms are as follows:

Response: We thank you for your careful evaluation and constructive comments. We appreciate the positive assessment of the originality and significance of our work, especially the rapid-cooling-induced vdW epitaxy strategy for stabilizing single-crystalline α-MoO_3 and its application as an ultra-thin high-*κ* vdW dielectric. We also appreciate your suggestions on clarifying several key issues. In response, we have revised the manuscript thoroughly according to your comments and provide detailed point-by-point responses below, and we hope that you will find our revised version of the manuscript satisfactory for publication.

1. The authors claim the achievement of an Equivalent Oxide Thickness (EOT) of ~ 0.42 nm (physical thickness ~ 2.0 nm). However, the critical performance metrics—specifically leakage current and reliability—appear to be derived from significantly thicker samples. Specifically, the leakage current density meeting the ITRS low-power limit (10^-3 A cm^-2) is shown for an EOT of 0.87 nm (4.1 nm physical), not the 0.42 nm sample. Similarly, the breakdown field statistics (E_0 ~ 7.0 MV cm^-1) are presented for a ~7.1 nm thick film, again not the 0.42 nm sample. This is my major concern, and the authors should provide leakage and breakdown statistics specifically for devices near the minimum EOT.

Response: We thank you for raising this important question. In the original manuscript, the leakage current and breakdown statistics were mainly presented for relatively thicker MoO_3 films, which did not directly address the dielectric performance near the minimum EOT of ~0.42 nm. In response to this comment, we further improved the device fabrication process and carried out additional electrical measurements on ultrathin MoO_3 metal-insulator-metal devices with MoO_3 thicknesses close to the minimum EOT.

Specifically, we adopted a new fabrication strategy in which pre-patterned Au electrodes were transfer-printed onto glass or sapphire substrates using a UV-curable adhesive, as shown in Fig. R7. In brief, the gold electrodes were deposited on a flat Si substrate using the conventional method. Then, a layer of Norland optical adhesive (NOA61) was spin-coated on the surface with a clean glass chip mounted on the NOA61 film, followed by the UV exposure for approximately one hour. The bottom Au electrodes with an ultra-flat surface were obtained by peeling the glass off the Si substrate. This method provides two important advantages. First, the height difference between the bottom electrode and the substrate becomes much smaller, resulting in an almost planar surface. Second, because the exposed Au surface was originally deposited on a smooth Si wafer and then peeled off during the transfer process, it is smoother than that obtained in our previous process. In contrast, in the original fabrication process, MoO_3 was directly transferred onto pre-fabricated Au electrodes. Owing to the 30-50 nm step height of the Au electrodes, ultrathin MoO_3 flakes were more easily damaged during transfer. Moreover, the surface roughness of the evaporated Au and possible fabrication-related residues could degrade the contact quality between Au and MoO_3. With the improved process, we obtained significantly higher-quality ultrathin MIM devices, as shown in Fig. R8.

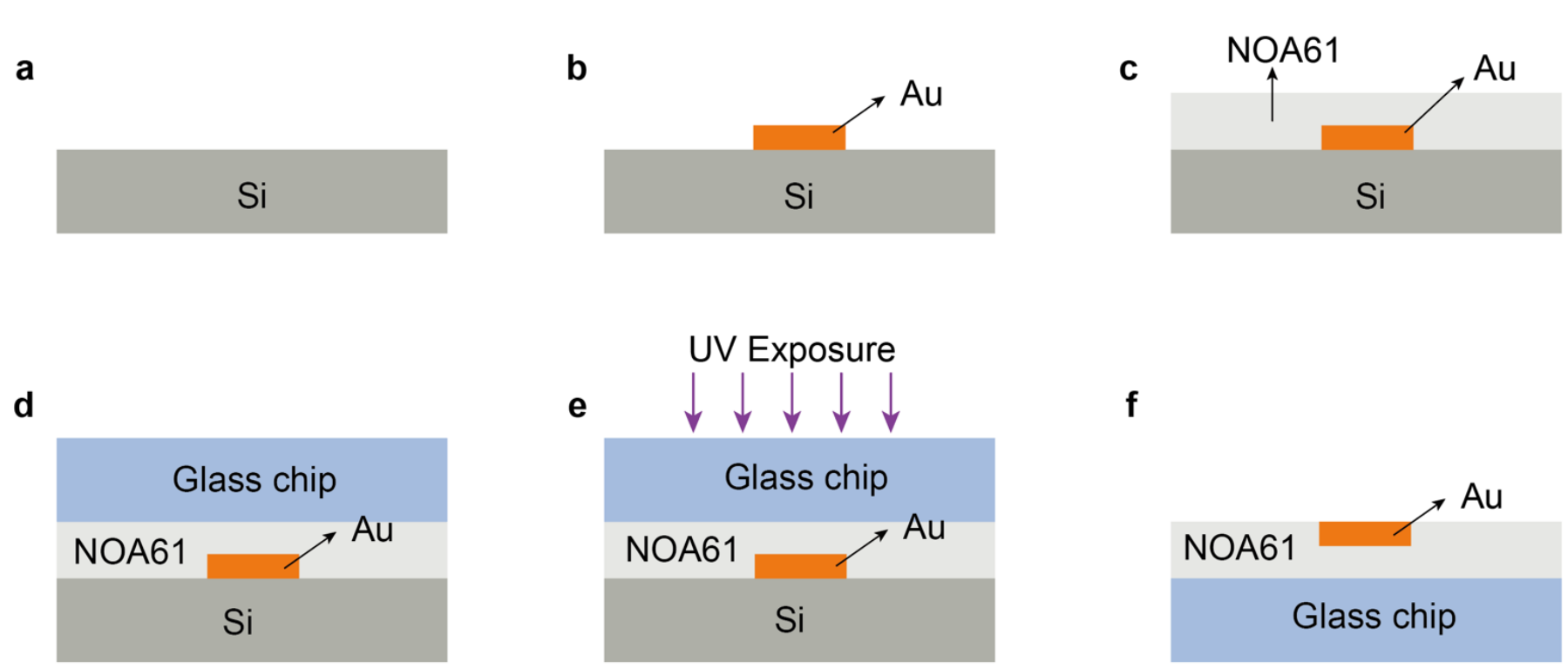

Fig. R7 Procedure of the fabrication of the Au substrate. a, Empty flat Si substrate. **b,** Standard evaporation of Au electrodes on a Si substrate. **c,** Spincoating of NOA61 on Au electrodes. **d,** Mounting of a glass chip on the optical adhesive. **e,** UV exposure. **f,** The Au substrate after the removal of the Si substrate.

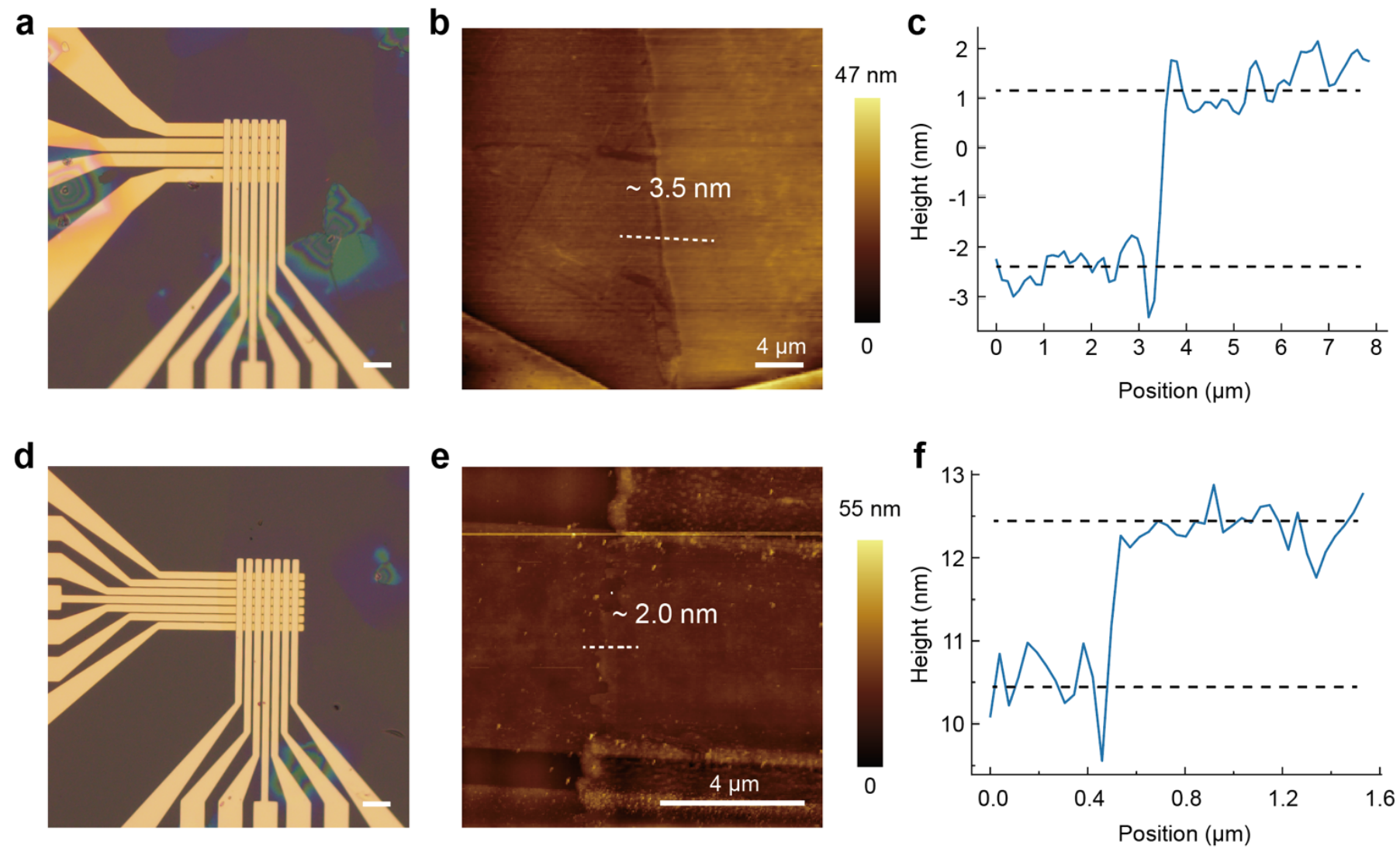

Fig. R8 Metal-insulator-metal device with thin MoO_3 film. a-c, Optical image, AFM image, and corresponding height profile of the MIM device with a 3.5 nm MoO_3 film. **d-f,** Optical image, AFM image, and corresponding height profile of the MIM device with a 2.0 nm MoO_3 film.

The thicknesses of the MoO_3 flakes were measured by AFM to be approximately ~2.0 nm and ~3.5 nm. Their leakage current and breakdown behavior were then characterized using a Keithley 4200 semiconductor parameter analyzer and a probe station under vacuum at room temperature. The corresponding leakage current density and breakdown data are presented in Fig. R9 and Fig. R10, respectively, with the x-axis converted into the corresponding electric field for direct comparison. Importantly, for the ~2.0 nm MoO_3 device, corresponding to an EOT of ~0.42 nm, the leakage current density remains at the level of ~10^{-2} A cm^{-2} at 3 MV cm^{-1}, meeting the ITRS low-power limit.

According to the *I-V* curves shown in Fig. R9, we evaluated the breakdown characteristics of the ~2.0 nm MoO_3 devices. We performed a Weibull analysis after excluding three obvious abnormal data points, and the results are shown in Fig. R10. The characteristic breakdown field extracted is approximately 26.75 MV cm^{-1}, which is

substantially higher than the value of about 7 MV cm^{-1} previously obtained for the 7.1 nm sample. This thickness dependence is consistent with the commonly reported trend in dielectric materials, where thinner dielectric layers generally exhibit a higher breakdown field[7].

Thank you again for your great suggestion, and we hope the newly added measurements and results will address your concern.

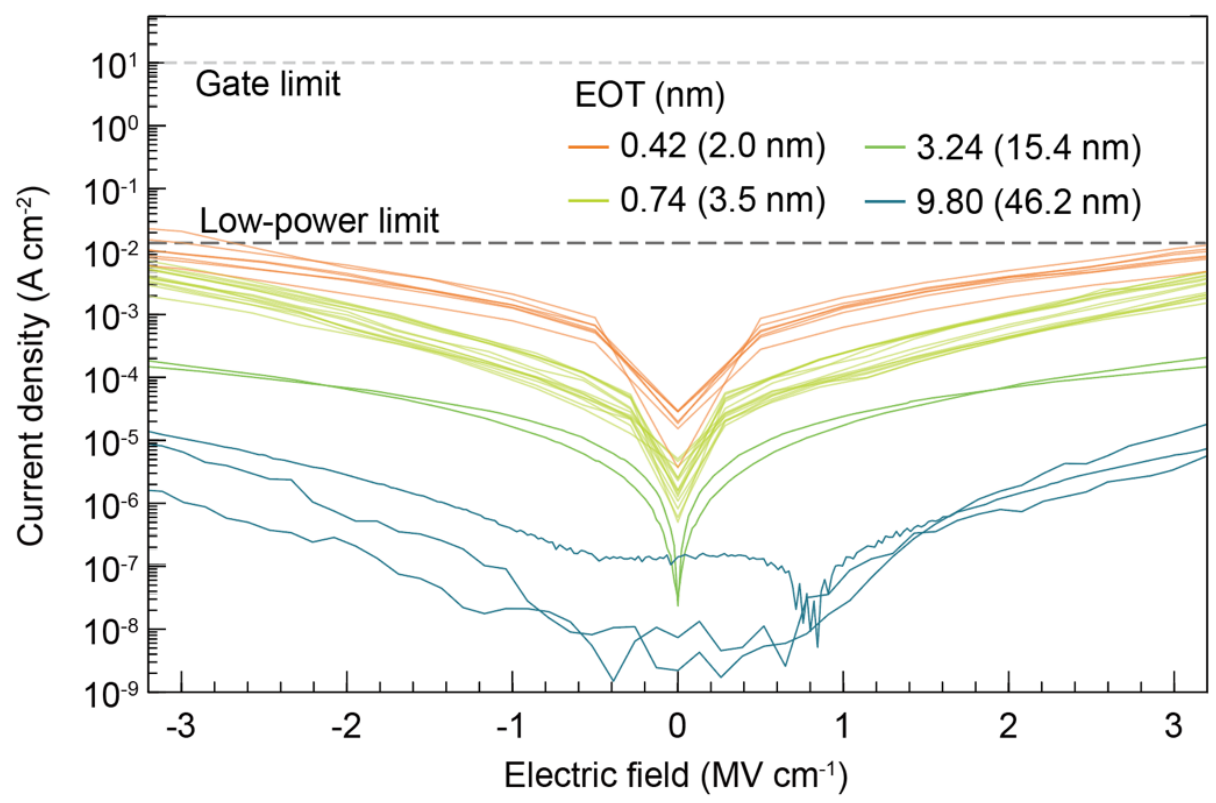

Fig. R9 Leakage current density of single-crystalline MoO_3 with different EOT values as a function of applied electric field.

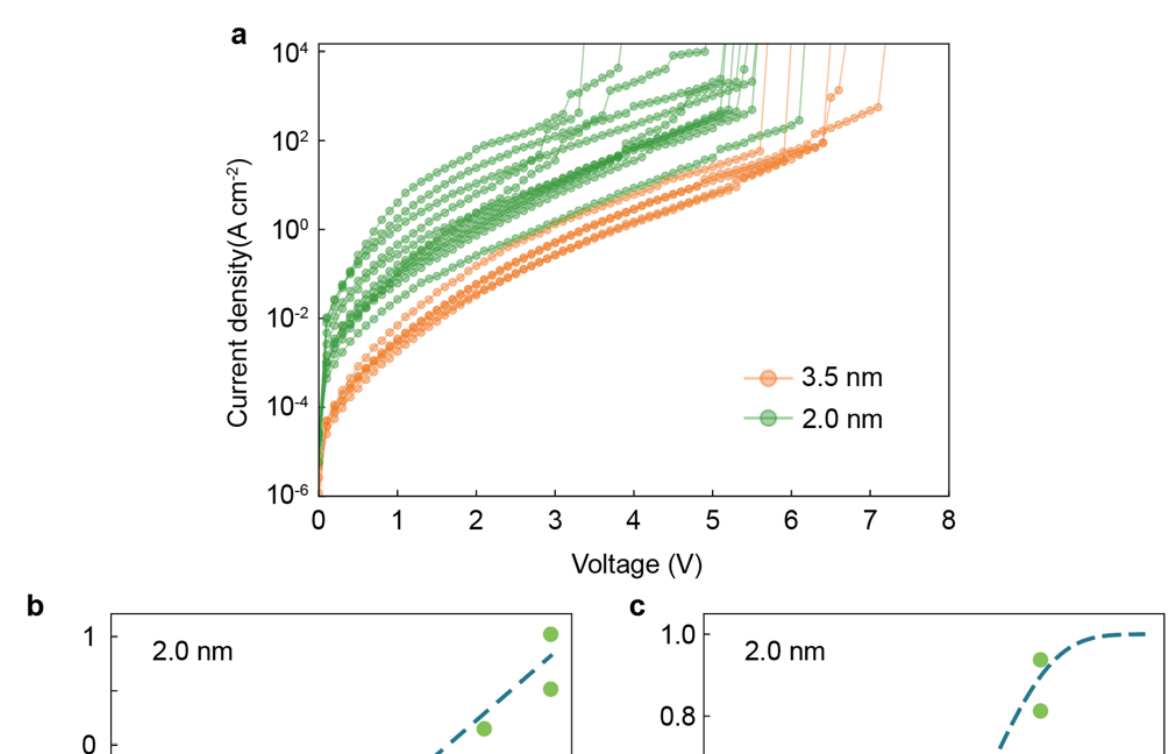

Fig. R10 Electric field at breakdown of MoO_3. a, Leakage current as a function of the voltage of MoO_3 with different thicknesses. **b,** According to the data of 2.0 nm MoO_3, the plot of $\ln[-\ln(1-P)]$ versus $\ln(E)$ and their linear fitting, from which we extracted β and E_0. **c,** The plot of cumulative failure probability P versus electric field, together with the Weibull fitting curve calculated using β and E_0. The dashed circle marks the electric field

when P is approximately 63.2%, corresponding to E_0, which is approximately 26.75 MV cm^{-1}.

Changes made to the manuscript:

1. Figure 2f and 2g have been replaced by Fig. R10 and Fig. R9.

2. Supplementary Note 3 for the estimation of the electric field at breakdown has been fully updated using the new data.

3. Fig. S21 in the original revision (Fig. S20 in the revised version) has been updated as shown below.

4. Line 46, "The size of single-crystalline MoO_3 can reach the millimeter scale, while maintaining a high dielectric constant of ~18.5 and a low equivalent oxide thickness (EOT) ~0.42 nm (physical thickness ~2.0 nm). At this ultrathin thickness, MoO_3 exhibits low gate leakage of approximately 10^{-2} A cm^{-2} at 3.0 MV cm^{-1} and a large breakdown field of ~26.8 MV cm^{-1}. Top-gated molybdenum disulfide (MoS_2) transistors exhibit an average subthreshold swing of ~71 mV dec^{-1}, and an on/off current ratio exceeding 10^8."

5. Line 93, "It has a high dielectric constant of ~18.5, an equivalent oxide thickness (EOT) of ~0.42 nm, a large electric breakdown field of ~26.8 MV cm^{-1}, and a low gate leakage current of approximately 10^{-2} A cm^{-2} at 3.0 MV cm^{-1} at an ultrathin thickness of ~2.0 nm."

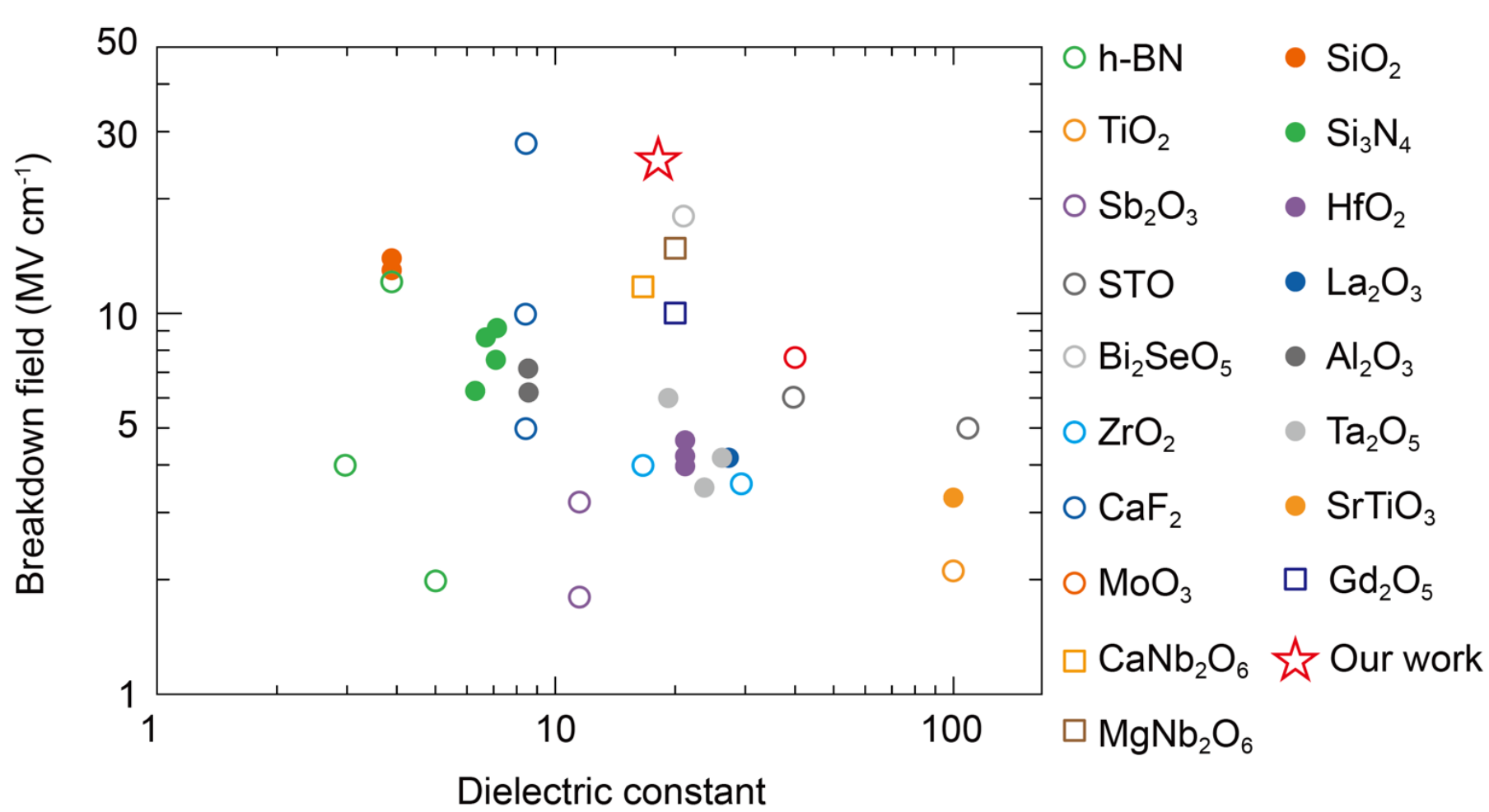

Fig. S20 The electric field at breakdown versus dielectric constant of state-of-the-art dielectrics**.** For convenience, vdW (quasi-vdW) and non-vdW dielectrics are labeled in hollow circles and solid circles, respectively.

2. Regarding the claim on the "low density of trap states", the authors in fact relied on indirect evidence—Subthreshold Swing (SS) and theoretical DFT binding energies. This contradicts the discussion on the device arrays, where the authors explicitly attribute the low field-effect mobility.

Please provide a direct quantitative measurement of Dit (e.g., using frequency-dependent C-V conductance methods, charge pumping, or transient techniques adapted to 2D systems).

Response: We thank you for raising this important point. In the original manuscript, our discussion of the low trap-state density mainly relied on indirect evidence, including the near-ideal subthreshold swing and the calculated interfacial binding characteristics. We also appreciate you for pointing out the inconsistency with our discussion of the device-array performance, where transfer-related imperfections may limit the field-effect mobility in some devices. In response to this comment, we carried out direct quantitative evaluations of the trap density D_{it} following your suggestions, using three complementary approaches, including frequency-dependent *C-V* conductance analysis[8], charge pumping method[9], and low-frequency 1/*f* noise measurements[10,11].

***C-V* conductance analysis.** Following your advice, we carried out frequency-dependent capacitance and conductance measurements on the Au(bottom)/MoO_3/MoS_2/Au(top) bottom-gate structure using the Keithley 4200-CMU system. During the measurement, the top Au electrode was grounded, while a small AC signal superimposed on a DC bias was applied to the bottom Au electrode. To extract the interfacial trap response, we plotted G_p/ω as a function of frequency under different DC bias as shown in Fig. R11a. G_p is the AC conductance of the traps and is obtained by considering the dielectric contribution of the MoO_3 layer using the formula below[12]:

$$\frac{G_p}{\omega} = \frac{\omega G_m C_{ox}^2}{G_m^2 + \omega^2 (C_{ox} - C_m)^2}$$

where $\omega = 2\pi f$, C_m and G_m are the measured capacitance and conductance of the device, and C_{OX} is the effective capacitance of the MoO_3 dielectric. The interface trap density was then estimated from the peak value of the corrected conductance using the equation

$$D_{it} \approx \frac{2.5}{qA}\left(\frac{G_p}{\omega}\right)_{peak}$$

where q is the elementary charge, and A is the device area. The extracted D_{it} values as a function of voltage are plotted in Fig. R11b. The interface trap density is on the order of ~5×10^{11} cm^{-2} eV^{-1} and shows little dependence on V_G within the measured bias range.

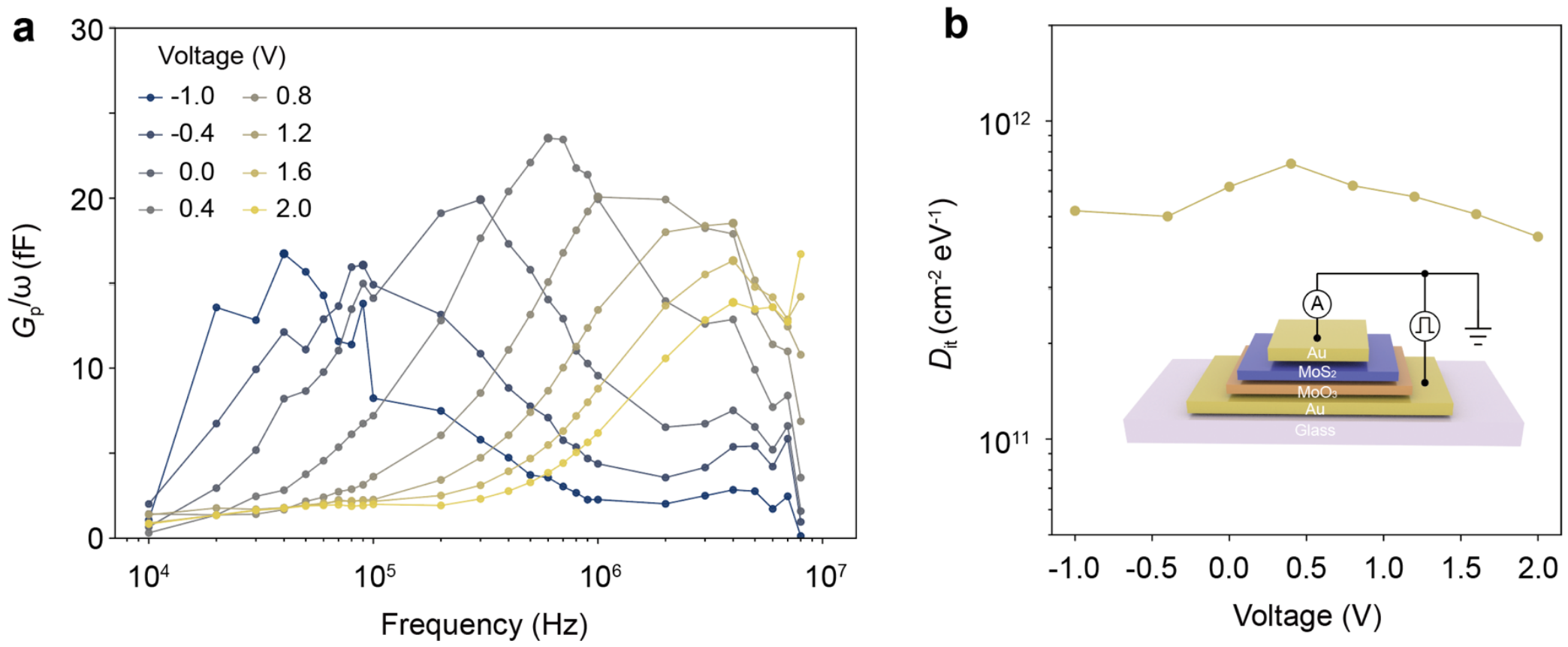

Fig. R11 Estimation of the interfacial trap density of a bottom gate device using the *C-V* conductance method. a, G_p/ω versus the frequency of the device under different voltages. **b,** Extracted trap density from the peak values in b as a function of voltage.

Charge pumping method. To obtain the interfacial trap density in the top-gated device, we further fabricated the MoS_2-MoO_3-Au device with a MoS_2 layer directly exfoliated on a sapphire substrate and measured D_{it} using the charge pumping method as you suggested, and $1/f$ method.

The charge pumping method has been adapted to 2D TMD FETs and probes interfacial traps through the periodic capture and emission of carriers during gate-pulse cycling[13]. In our measurements, the source and drain were grounded, while a DC offset together with a square-wave gate pulse was applied to the gate electrode, and the resulting current at the source terminal was monitored, as illustrated in Fig. R12. In this method, the gate pulse periodically sweeps the channel Fermi level across the interfacial trap states, leading to repeated carrier capture and emission during each pulse cycle. The resulting average charge-pumping current I_{cp} is therefore directly related to the density of electrically active traps. The average charge-pumping current can be written as $I_{cp} = qAfN_{it}$, where q is the elementary charge, A is the device area, f is the pulse frequency, and N_{it} is the interface

trap charge density involved in the pumping process. If the trap density is approximately uniform within the pulse-swept energy window ΔE, the effective interface trap density can be estimated as

$$D_{\mathrm{it}} \approx \frac{1}{qA\Delta E}\frac{dI_{\mathrm{cp}}}{df}$$

As shown in Fig. R12, I_{cp} increases linearly with frequency under fixed pulse conditions (V_{base} = -1V and V_{pp} = 1V), which is a characteristic signature of charge pumping. The fitted slope is about 8.1×10^{-15} A/Hz, confirming that the measured current mainly originates from periodic trap-assisted carrier exchange rather than simple leakage current. The dependence of I_{cp} on the V_{base} is shown in Fig. R12b for f = 0.5 MHz and V_{pp} = 1V. A clear charge-pumping window is observed, that is, the current is small at both low and high V_{base}, but reaches a maximum in an intermediate bias range. This indicates that only within this bias window does the pulse effectively sweep across the energy range where interfacial traps can actively participate in carrier capture and emission. The dependence of I_{cp} on the pulse amplitude is presented in Fig. R12c for f = 0.5 MHz and V_{base} = -1V. As V_{pp} increases, I_{cp} first rises and then tends to saturate. This behavior indicates that a larger pulse amplitude probes a wider trap energy window, while the saturation at high V_{pp} suggests that most accessible active traps have already been involved in the pumping process. Based on the processed charge-pumping data, we estimate an effective interface trap density of approximately ~2×10^{11} cm^{-2} eV^{-1}. Overall, the linear frequency dependence, the well-defined V_{base} window, and the V_{pp}-dependent activation behavior consistently confirm the presence of electrically active trap states at the MoO_3/MoS_2 interface.

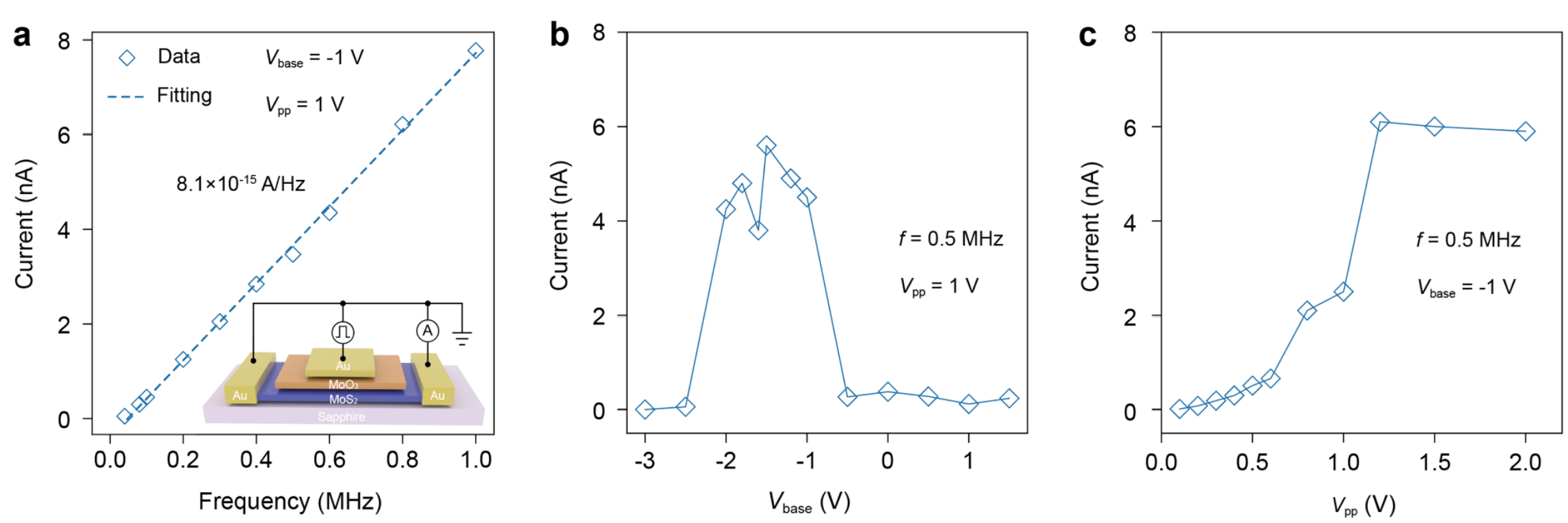

Fig. R12 Estimation of interfacial trap density of a top gate device using the charge pumping method. a, The charge pumping current as a function of frequency, inset is the

basic measurement circuit. **b,** The charge pumping current as a function of base voltage. **c,** The charge pumping current as a function of pulse amplitude.

Low-frequency 1/*f* noise measurements. In addition, using the same bottom-gate MoS_2 FET platform, we independently evaluated the trap density by low-frequency 1/*f* noise measurements[10,11]. In this method, the source and drain were grounded, and the current-noise spectra were measured under different gate voltages. The D_{it} could be extracted from the following equation:

$$D_{\mathrm{it}} = \frac{S_I\, WLC_{\mathrm{ox}}^2 f}{g_{\mathrm{m}}^2 q^2 k_{\mathrm{B}} T}$$

where S_I is the spectral density, W and L are the width and the length of the channel, C_{ox} is the gate capacitance per unit area, g_m is the gate transconductance, q is the electron elementary charge, k_B is the Boltzmann constant, and T is the temperature. The obtained noise spectra remain similar at different gate biases and exhibit good linearity in the low-frequency regime (Fig. R13a), consistent with 1/*f* behavior. According to the equation, the interfacial trap density extracted from the noise data and the transfer curve is approximately ~1.5×10^{11} cm^{-2} eV^{-1}, which is in good agreement with the value obtained from charge pumping. And the D_{it} value almost shows no obvious difference in the low frequency range as shown in Fig. R13c.

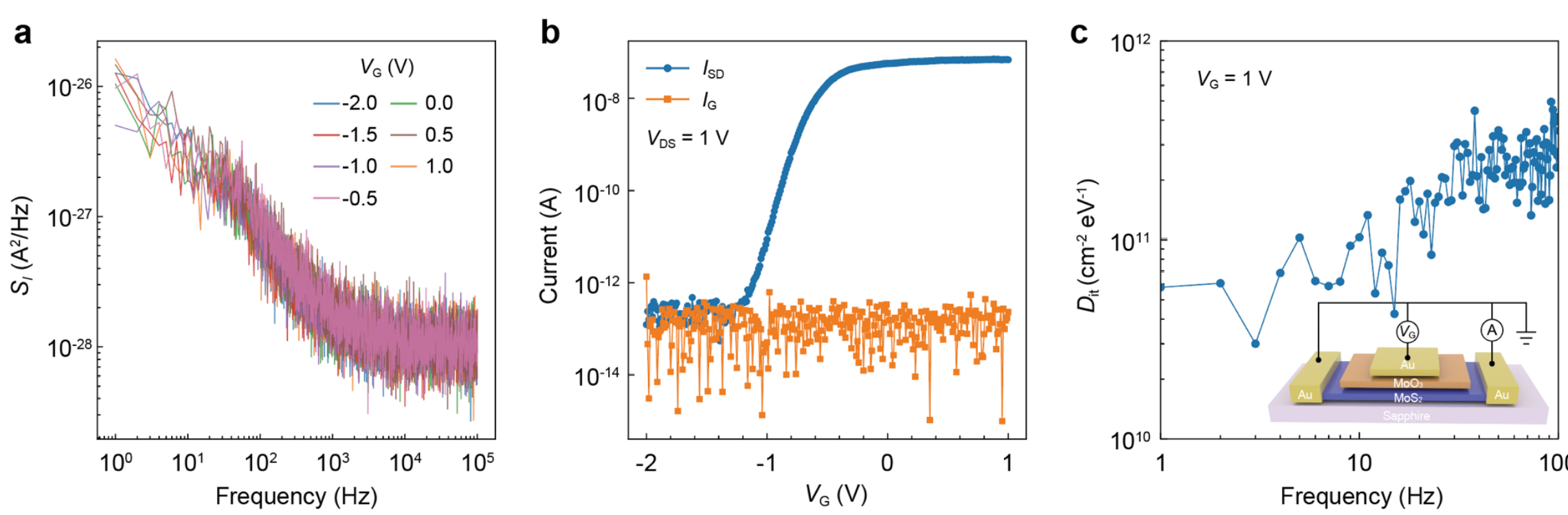

Fig. R13 Estimation of interfacial trap density of a top gate device using the 1/*f* method. a, Noise spectra as a function of frequency with different gate voltages at V_{DS} = 1 V. **b,** The source-drain current and gate leakage current as a function of gate voltage. **c,** Calculated interfacial trap density at V_G = 1 V, the interfacial trap density exhibits no obvious difference at low frequency.

Overall, the value of D_{it} is ~2 × 10^{11} cm^{-2} eV^{-1} from the charge pumping method, ~1.5 × 10^{11} cm^{-2} eV^{-1} from the 1/*f* noise method. By contrast, we obtained a larger value of D_{it} (~5 × 10^{11} cm^{-2} eV^{-1}) from the vertical *C*-*V* conductance structure in the Au-MoO_3-MoS_2-Au bottom-gate device structure, which is likely associated with the additional transfer step in the fabrication process. In this process, MoS_2 was transferred from PDMS to the MoO_3/Au bottom stack, which can introduce additional interfacial contamination and local defects, leading to a higher effective trap density. This is consistent with previous reports showing that transfer-induced residues, wrinkles, and contamination can substantially affect the interfacial quality and electronic properties of 2D-material devices[14].

Changes made to the manuscript:

1. We have revised the manuscript to make the discussion more precise and to avoid any errors. Specifically, the reduced field-effect mobility observed in some devices in the array is now discussed more carefully in terms of transfer-induced wrinkles, particles, local cracking, and non-uniform interfacial contact.

Line 370, "The non-ideal yield and relatively reduced mobility are mainly attributed to local cracking, wrinkles, and interfacial trap states introduced during the large-area integration of MoO_3 and MoS_2 films. Those performances can be further improved through continued optimization of the transfer and integration processes."

2. Accordingly, we have added the direct D_{it} measurements in the supplementary information as Note 5.

3. Line 325, "The interfacial trap density was further obtained as ~5 × 10^{11} cm^{-2} eV^{-1} by the frequency-dependent *C*-*V* conductance analysis[60], ~2 × 10^{11} cm^{-2} eV^{-1} by the charge pumping method[61], and ~1.5 × 10^{11} cm^{-2} eV^{-1} by the low-frequency 1/*f* noise measurements[62, 63], which is competitive to most D_{it} values of exfoliated MoS_2 channels[62]."

3. A central claim of the paper is the "scalability" of the MoO3 integration. However, the data provided for the device arrays suggests significant challenges that are not fully quantified. The statistical distribution of the array devices shows a clear degradation in performance (SS ~ 70–110 mV dec^-1 and On/Off ratios of 10^6-10^7) compared to the single champion device. They should provide a yield map of the fabricated array (showing

functional vs. non-functional devices) and explicitly state the percentage yield. Additionally, the criteria used to define a "successful" device need to be clarified.

Response: We thank you for raising this important point. We agree that the claim of scalability should be supported not only by representative device performance, but also by a clear evaluation of the array yield, and the criteria used to define functional devices.

In the original manuscript, we presented the transfer characteristics and the statistical distributions of key electrical parameters for more than 100 devices, which indicate the feasibility of array-level integration of large-area MoO_3. At the same time, we acknowledge that the original manuscript did not explicitly provide a yield map, did not state the percentage yield, and did not clearly define the criteria used to classify functional and failed devices.

In response to your advice, we have revised the manuscript accordingly. First, we have clarified the criterion used to define a functional (successful) device, which was not explicitly stated in the original manuscript.

Following previous reports[15], in which devices with an on/off current ratio below a predefined threshold were classified as failed devices. In this work, a successful transistor is defined as a device showing normal field-effect switching behavior, with I_{on}/I_{off} above 10^5 and I_{on} higher than 10^{-7} A. We use this criterion because it simultaneously reflects effective gate control and sufficient output current, which are both essential for practical array integration and yield evaluation. Similar definitions based on I_{on}/I_{off} and I_{on} have been used in the statistical distributions of array-level 2D electronics to evaluate device uniformity and functionality[14]. Devices that do not meet this condition are classified as failed devices. Based on this criterion, we have added a spatial map of I_{on}/I_{off} and I_{on} for the fabricated array, in which the on/off ratio of each device is explicitly shown, as presented in Fig. R14. According to this analysis, the yield of the array is 81%, corresponding to 102 functional devices out of 126 fabricated devices. We would like to emphasize that the present array results demonstrate the integration capability and scalability potential of large-area MoO_3, although the current yield is still limited. We attribute the non-ideal yield mainly to the transfer-induced imperfection during the large-area integration of MoO_3 and MoS_2 films, including local cracking, wrinkles,

contamination, and non-uniform interfacial contact. The yield can be further improved through continued optimization of the transfer and integration process.

Overall, the added yield map, yield percentage, and explicit success criterion provide a more rigorous and transparent assessment of the current array performance and potential of the MoO_3 film, which improves the quality of our manuscript quite a lot. We hope that the revised version will meet your satisfaction.

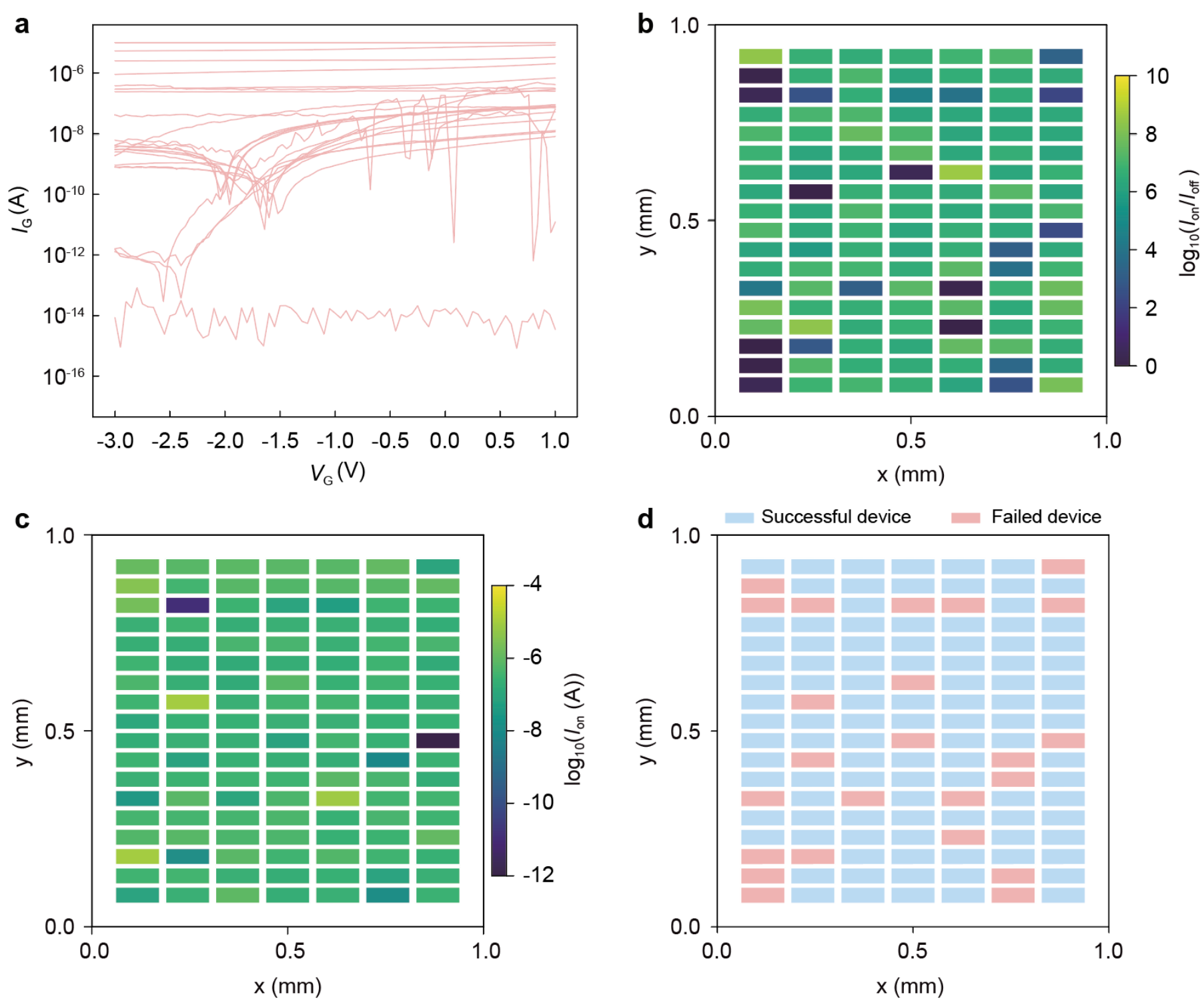

Fig. R14 Heatmaps of the key parameters of the MoS_2 FET array over a 1 × 1 mm^2 area. a, Transfer curves of those failed devices. **b,** I_{on}/I_{off}. **c,** I_{on}. **d,** yield map of the fabricated array.

Changes made to the manuscript:

1. Fig. R14 has been added in the supporting information as Fig. S35.

2. Line 362, "In our work, a device is defined as successful when it exhibits clear gate-modulated transfer behavior with both I_{on}/I_{off} beyond 10^4 and I_{on} beyond 10^{-7} A. Devices not meeting these criteria are classified as failed devices. Fig. 4b presents the I_D-V_G curves of successful devices, while the data of the failed devices are provided in Fig. S35a. The spatial distribution of I_{on}/I_{off}, I_{on}, and the device yield map are summarized in Fig. S35. The

overall device yield is approximately ~81%, corresponding to 102 functional devices out of 126 fabricated devices. Statistical analysis of the successful device shows relatively uniform performance (Fig. 4c, Fig. S36)."

4. Two different methods yield substantially different dielectric constants (κ ≈ 25 vs. κ ≈ 18), which are attributed to polymer residues or interfacial effects. While plausible, this discrepancy affects all derived quantities (EOT, charge density, SS interpretation). Since κ is central to the paper's claims, this issue affects the quantitative credibility of the dielectric performance. The authors should provide statistical distributions (mean, standard deviation, number of devices) for κ and EOT, and if possible include additional cross-validation (e.g., well-defined MIM capacitors or thickness-localized metrology). At minimum, clarify which κ value should be regarded as intrinsic.

Response: We thank you for raising this important concern. We agree that the dielectric constant κ is a central parameter in this work, and that the discrepancy between the two extracted values requires a clearer explanation, since it directly affects derived quantities such as EOT, charge density, and the electrostatic interpretation of the devices.

Following your suggestion, we carried out an additional cross-validation using newly fabricated Au/MoO_3/Au capacitor devices with five different MoO_3 thicknesses, as shown in Fig. R15. For each thickness, the capacitance-voltage (*C*-*V*) characteristics were measured using the Keithley 4200 and probe station under the same conditions as in the original manuscript. The new data were then processed using the same analysis procedure as in the original manuscript, that is, the capacitance data were converted into the corresponding areal capacitance and analyzed using the same thickness-dependent fitting method to extract the dielectric constant. The statistical mean values and standard deviations for each thickness are provided in Fig.R16. Fitting the newly measured dataset alone gives a dielectric constant of ~17.2, which is very close to the value ~18.4 reported in the original manuscript.

In addition, because the newly added devices and the original devices share the same capacitor structure and were measured using the same instrumentation and analysis protocol, we further performed a unified re-analysis by combining the new and original datasets. In this analysis, devices with MoO_3 thicknesses differing less than 10% were

grouped as the same thickness, and the corresponding mean capacitance values and standard deviations were calculated for each group. A linear fit based on the combined dataset was then performed, as shown in Fig. R17, yielding a dielectric constant of 18.5, again in good agreement with the original result. We therefore adopt this combined fitting result as the final dielectric constant in the revised manuscript.

In the original manuscript, the two κ values were obtained from two different methods: $\kappa \approx 25.1$ from the dual-gate graphene method, and $\kappa \approx 18.4$ from direct capacitance measurements. We agree that these two values should be more carefully distinguished in terms of their physical meaning. In the revised manuscript, we clarify that the value extracted from the direct capacitor measurement should be regarded as the one closest to the intrinsic dielectric constant of MoO_3, whereas the larger value extracted from the dual-gate graphene device is better understood as an effective dielectric constant in a specific device geometry, which may include contributions from interfacial residues, parasitic electrostatic coupling, and other non-idealities.

Based on these additional measurements and statistical analyses, we have added the statistical distributions of κ and EOT, including the mean values and standard deviations, to make the quantitative analysis more rigorous. Importantly, the additional capacitor-based cross-validation confirms that the dielectric constant extracted from direct capacitance measurements is robust and reproducible. Therefore, in the revised manuscript, we explicitly take this capacitor-based value as the intrinsic dielectric constant of MoO_3, while the larger value extracted from the dual-gate FLG structure is discussed as an effective device-level dielectric response.

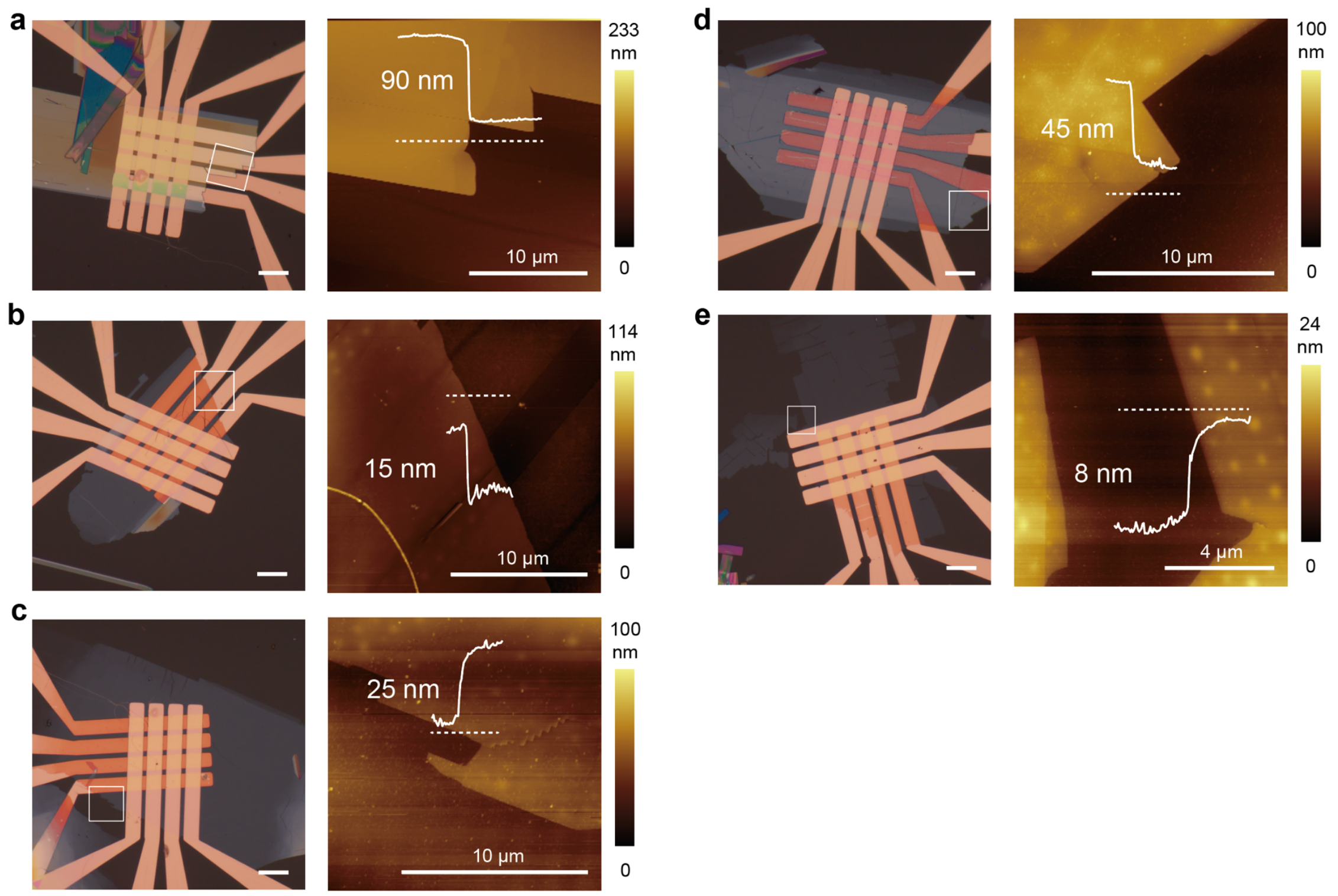

Fig. R15 Metal-insulator-metal device with different MoO_3 thickness, including optical image, AFM image, and corresponding height profile.

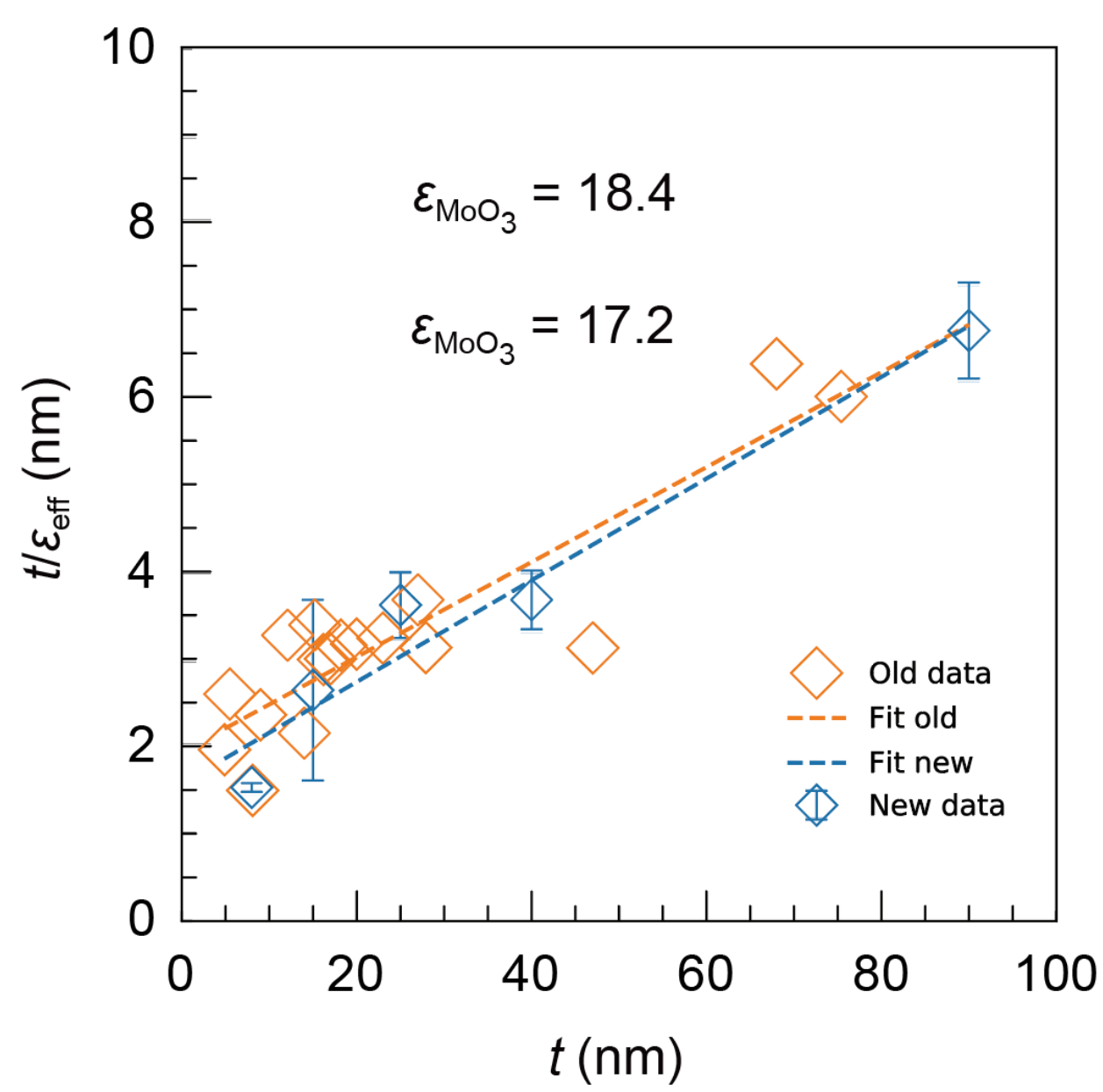

Fig. R16 t/ε_{eff} as a function of MoO_3 thickness, newly obtained results and results in the original manuscript are plotted separately. The results in our original manuscript is presented in yellow, and the new result is labeled in blue. The new results are obtained

from the devices shown in Fig. R14. For each thickness, we measured 4 to 6 points, and plotted the mean and standard deviation in the figure. The dashed line is the fitting result using the formula $t/\varepsilon_{\text{eff}} = t/\varepsilon_{\text{bulk}} + \text{D}$, and the bulk dielectric constants are extracted from the slope.

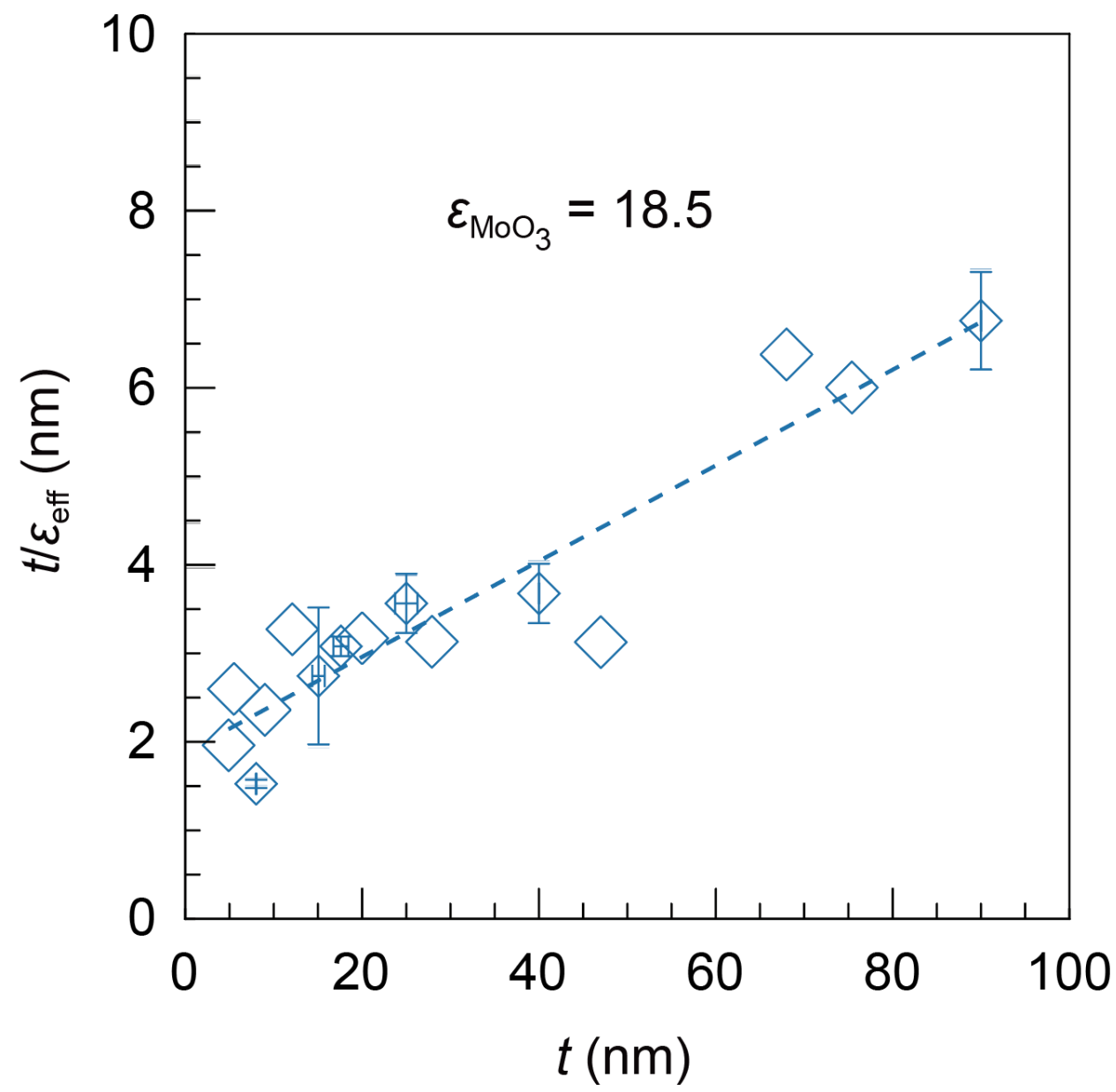

Fig. R17 t/ε_{eff} as a function of MoO_3 thickness. The new results and old results are combined together. For thicknesses that are within 10% are considered as a same thickness.

Changes made to the manuscript:

1. Fig. 2d has been updated using Fig. R17.

2. Line 232, "The constant D is approximately 1.9 nm, and ε_{bulk} is approximately 18.5 from the linear fitting, which is close to ~17 of ALD-grown MoO_3[38] (Table S1). It should be emphasized that this dielectric constant can be extended to ultrathin MoO_3 due to the thickness-independent dielectric constant that has been demonstrated before[39]. The value extracted from the direct capacitor measurement is considered as the intrinsic dielectric constant of MoO_3, whereas the larger value ~25.1 extracted from the dual-gate graphene device is an effective dielectric constant in a specific device geometry, which may include contributions from interfacial residues, parasitic electrostatic coupling, and other non-idealities. Fig. 2e summarizes the lateral single-crystalline size versus dielectric constant of state-of-the-art vdW dielectrics. Compared to other vdW dielectrics, MoO_3 possesses a

millimeter size and a high dielectric constant simultaneously, making it an exceptional choice for future monolithic 3D integration."

3. The dielectric constant is changed from 18.4 to 18.5, as well as the related derived quantities throughout the manuscript.

4. Fig. 2e has been updated using the updated dielectric constant and some new references that were published recently.

5. The reported static power consumption of 0.2 nW at VDD = 1.0 V is highlighted as a key achievement. However, this figure lacks the necessary context. The champion single transistors exhibit Ioff ~ 10^-14 A, which should theoretically yield static power in the femtowatt range ($~ 0.01 pW). The reported 0.2 nW is roughly 20,000 times higher, implying significant leakage degradation in the inverter integration. To resolve the issue, provide the IDD-VIN curves for the inverter at VDD = 1.0 V, and specify the channel dimensions (W/L) of the inverter transistors and normalize the static power (e.g., W/micro-m) to allow for proper benchmarking. Also, clarify the specific back-gate bias used to achieve the 0.2 nW figure.

Response: We thank you for raising this important concern. The original manuscript did not provide sufficient context for the reported static power consumption of the inverter, particularly the corresponding I_{DD}-V_{IN} characteristics, the transistor dimensions, and the bias conditions used in the measurement. According to your suggestions, we have therefore added the corresponding I_{DD}-V_{IN} curves, specified the channel dimensions of the inverter transistors, included the normalized static power, and clarified the back-gate bias used in the measurement in the revised manuscript.

The static power was calculated from $P_{static} = V_{DD} \times I_{DD}$, where I_{DD} is the measured supply current from the V_{DD} terminal. As you have mentioned, the minimum off current of the champion single transistor is in the order of 10^{-14} A, and the static power should also be in the order of 10^{-14} W. However, as shown in Fig. R18a, I_{DD} is in the order of 10^{-10} A, leading to a static power in the order of 10^{-10} W.

The reason is that the off current corresponds to an individually optimized device under its own bias condition, whereas the inverter power is measured from a wired complementary inverter consisting of separate WSe_2 and MoS_2 FETs under circuit

operation. In the transition region, both transistors contribute to the supply current, so the measured I_{DD} can be much larger than the minimum off current of an individual transistor. More importantly, we notice that I_{DD} is comparable to I_{IN}, while the current at the ground side is much smaller, as shown in Fig. R18b. This indicates that the measured supply current contains a non-negligible input contribution. Consistent with this, the individual gate leakage current characteristics (Fig. R18c) show that, in the bias range relevant to inverter switching, the MoS_2 n-FET exhibits relatively small gate leakage, remaining at approximately 10^{-12}-10^{-10} A, whereas the WSe_2 p-FET shows larger gate leakage in the range of 10^{-10}-10^{-9} A. Since the measured inverter supply current in the transition region is also on the order of 10^{-10}-10^{-9} A, the static power of the present inverter likely includes a substantial contribution from WSe_2 gate leakage, while the contribution from MoS_2 gate leakage is comparatively much smaller. Therefore, the measured static power likely includes an important contribution from WSe_2 gate leakage, in addition to the channel current in the transition region.

In addition, we have specified the transistor dimensions (W/L is 7 μm /3 μm for the MoS_2 transistor and 4 μm /2 μm for the WSe_2 transistor), and normalized the static power by the total channel width W_n+W_p, giving 18 pW/μm at V_{DD} = 1.0 V.

For the back-gate voltage used in the 0.2 nW figure (18 pW/μm at V_{DD} = 1.0 V), the back gates were left unconnected. We apologize for the confusion caused by our previous description.

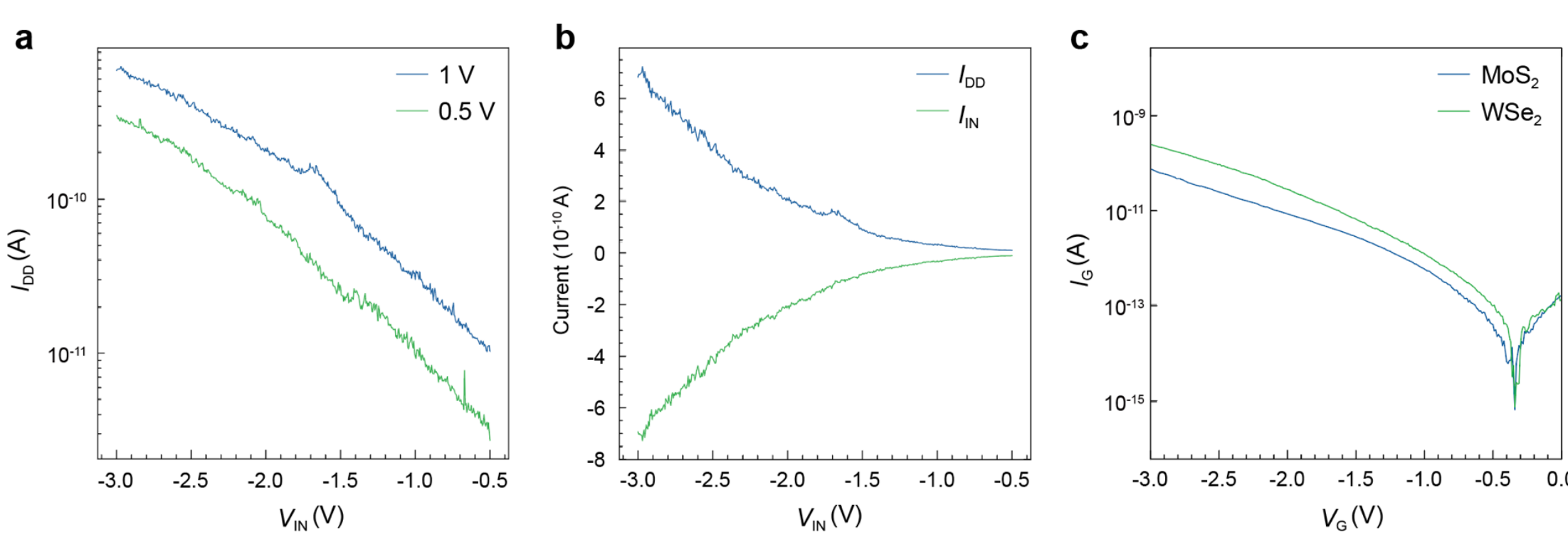

Fig. R18 Characterization of the inverter. a, I_{DD} as a function of V_{IN} at different V_{DD}. b, Comparison of I_{DD} and I_{DD}. c, Gate leakage current of MoS_2 and WSe_2 transistors.

Changes made to the manuscript:

1. Line 401, "The power exhibited a peak value for supply voltage at 2.0 V and 2.5 V. However, at lower supply voltages, no apparent peak of power consumption was observed, which is a result of 10^{-10}-10^{-9} A gate leakage current in the transition range in the WSe_2 transistor. In the transition region, the static power consumptions at different supply voltages are ~500 pW/μm for V_{DD} = 2.0 V, 0.130 nW/μm for V_{DD} = 1.5 V, ~38 pW/μm for V_{DD} = 1.0 V, and ~15 pW/μm for V_{DD} = 0.5 V. For another CMOS inverter, the power consumption is ~18 pW/μm at V_{DD} = 1.0 V (Fig. S42), which is competitive as compared to reported 2D CMOS inverters."

2. Line 98, "Importantly, MoO_3 exhibits potential in integrated circuits and low-power complementary metal-oxide-semiconductor (CMOS) inverter, which exhibited low power consumption of 18 pW/μm at V_{DD} = 1.0 V."

3. Caption of Fig. S41, "The static power consumption of the CMOS inverter as a function of input voltage at different supply voltages. Noted that the electrodes for the back gate are left unconnected."

Finally, as a minor comment on the vdW integration literature, note that there has been notable progress via transferring Hf- and Zr-based TMDs as precursors and oxidizing them. These references need to be cited:

- Luo, P. et al. Molybdenum disulfide transistors with enlarged van der Waals gaps at their dielectric interface via oxygen accumulation. Nat. Electron. 5, 849–858 (2022).
- Zhang, M. et al. Integration of ultrathin hafnium oxide with a clean van der Waals interface for two-dimensional sandwich heterostructure electronics. Nano Lett. 24, 1234–1240 (2024).
- Kang, T., et al. High-κ dielectric van der Waals integration on 2D semiconductors for three-dimensional complementary logic systems. Nat. Commun. 16, 11648 (2025).

Response: Thank you for your suggestions. We have cited the recommended references in the main text as Refs. 9-11. In addition, we have carefully reviewed other papers related to our work that appeared during the submission and revision process, and have cited them wherever appropriate.

Changes made to the manuscript:

1. Line 63, "Notable progress has been made by transferring Hf- and Zr-based TMDs as precursor layers and then oxidizing them into high-*κ* dielectrics[9-11]. While this approach has opened a viable route for dielectric integration on 2D semiconductors, it relies on multiple processing steps, and the resulting uniformity and interface quality depend sensitively on the precise control of the oxidation process."

2. Some new references that were published recently have been added in Fig. 2e.

Reviewer #3 (Remarks to the Author):

In this manuscript, Hu and co-workers reported the growth and integration of large-area crystalline MoO3 as a dielectric layer for two-dimensional electronic devices. The authors demonstrated large-area, uniform MoO3 films with atomically flat surfaces, a dielectric constant of approximately 18.4, and a low equivalent oxide thickness (EOT) ~0.42 nm, together with low leakage current densities and a high breakdown field ~7.1 MV cm^{-1}. MoS2 transistors based on MoO3 exhibited low subthreshold swing and high current on-off ratio. Importantly, the demonstration of the transistor array indicates the potential of large-area MoO3 as an alternative high-κ dielectric in integrated electronics based on 2D semiconductors. The topic of the research is of potential interest to researchers in the fields of 2D materials and nanoscale electronics. Therefore, I recommend its publication after the following issues have been fully addressed.

Response: We sincerely thank you for your positive evaluation of our work. We are grateful that you recognize the significance of our study, including the large-area growth and excellent dielectric properties, and potential as an alternative high-k dielectric for integrated electronics based on 2D semiconductors. Following your comments, we have carefully revised the manuscript and addressed all of them in details below. We believe that these revisions have improved the clarity and quality of our manuscript quite a lot, and hope that the revised manuscript will meet your satisfaction.

1. The device integration relies on multiple transfer steps, including transferring MoO3 from mica to SiO2 and the transfer of MoS2 and graphene, which may introduce interfacial defects. In this context, the authors are encouraged to have some discussion of further

optimization methods. It would be useful to comment on whether the MoO3 growth can be extended to other substrates, such as Al2O3 or SiO2. Furthermore, is the in-situ growth of MoO3 on MoS2 feasible, which I think will greatly reduce the interfacial disorders?

Response: Thank you for the valuable comments and suggestions regarding the interfacial defects introduced during the transfer steps in device integration. We agree that further optimization of the growth and transfer processes is important for improving interface quality.

In response to your question about extending MoO_3 growth to other substrates, our experimental results (Fig. R19a, b) demonstrate that MoO_3 can indeed be grown on both Al_2O_3 and SiO_2 substrates. However, the growth uniformity varies significantly. On Al_2O_3, MoO_3 forms a relatively uniform layer, while MoO_3 grown on SiO_2 tends to form a non-uniform, flocculent thick film. This suggests that substrate surface properties may play a critical role in the nucleation and layer-by-layer growth of MoO_3.

Regarding the feasibility of in-situ growth of MoO_3 on MoS_2, Fig. R19c provides direct evidence that MoO_3 can be grown directly on MoS_2 without any transfer process. The scratched region in Fig. R19c reveals the coexistence of both materials, and the Raman spectrum in the inset confirms the presence of characteristic peaks from both MoS_2 and MoO_3. The AFM data in Fig. R19d further show clear layered structures with thicknesses of ~0.7 nm for MoS_2 and ~2.5 nm for MoO_3, indicating that van der Waals epitaxy of MoO_3 on MoS_2 is achievable. This in-situ approach could indeed minimize interfacial disorders and contamination associated with transfer processes.

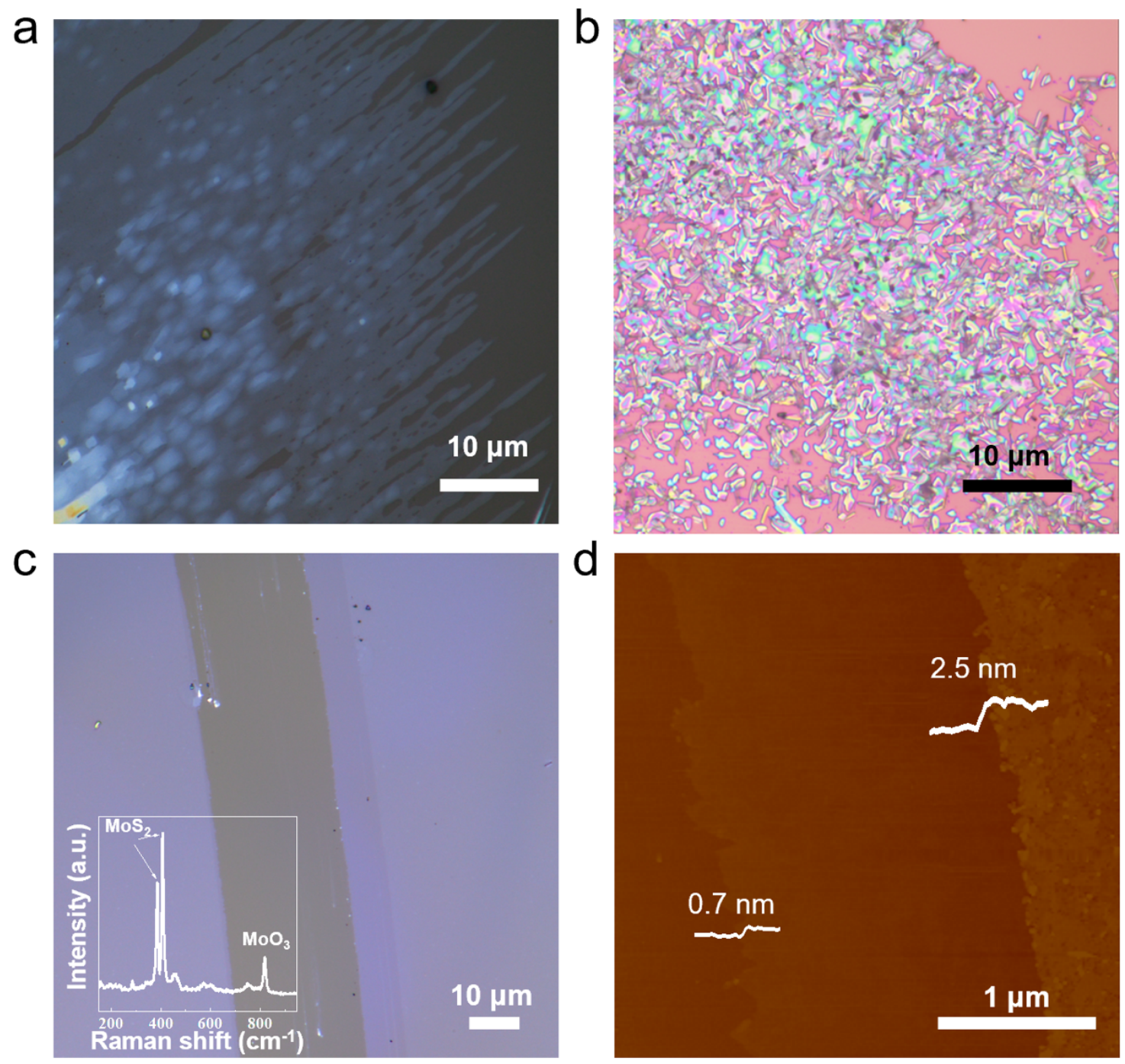

Fig. R19 Growth and characterization of MoO_3 on different substrates. a, b, Optical microscopy images of MoO_3 grown on Al_2O_3 and SiO_2/Si substrates, respectively. c, Optical microscopy image of MoO_3 directly grown on MoS_2 film. The lower-left inset shows the Raman spectrum taken from the MoO_3/MoS_2 area, **d,** Atomic force microscopy (AFM) image, and the corresponding height profile of the region in **c,** showing the MoS_2 thickness of ~0.7 nm and the MoO_3 thickness of ~2.5 nm.

Changes made to the manuscript:

1. Line 430, "Future work will focus on the direct growth of large-area MoO_3 on MoS_2, which could provide an in-situ integration strategy to substantially reduce the interfacial disorder and contamination associated with transfer processes."

2. This work reports a water-assisted transfer method to transfer MoO3 from mica to SiO2. What is the success rate of the transfer?

Response: We thank you for raising this important point. The transfer yield is a key metric for evaluating the practicality of the water-assisted transfer process. Here, the transfer success rate is evaluated based on the retained area coverage of MoO_3 on the target substrate after the water-assisted transfer process. Specifically, Fig. R20 shows the MoO_3 flakes on PDMS transferred from Mica. It shows that more than 80% of the MoO_3 area was

successfully delaminated from mica onto the PDMS stamp. The subsequent transfer from PDMS to the target SiO_2/Si substrate exhibits a success rate close to 100%. Therefore, based on the area coverage retained through the full transfer process, the effective transfer yield from mica to the target substrate is estimated to be higher than 80%. The main factors limiting the yield are transfer-induced imperfections, such as crackles/wrinkles and occasional contamination during the handling of large-area ultrathin flakes.

We have revised the manuscript accordingly and added a brief discussion on the current limitations and possible process to improve the transfer quality.

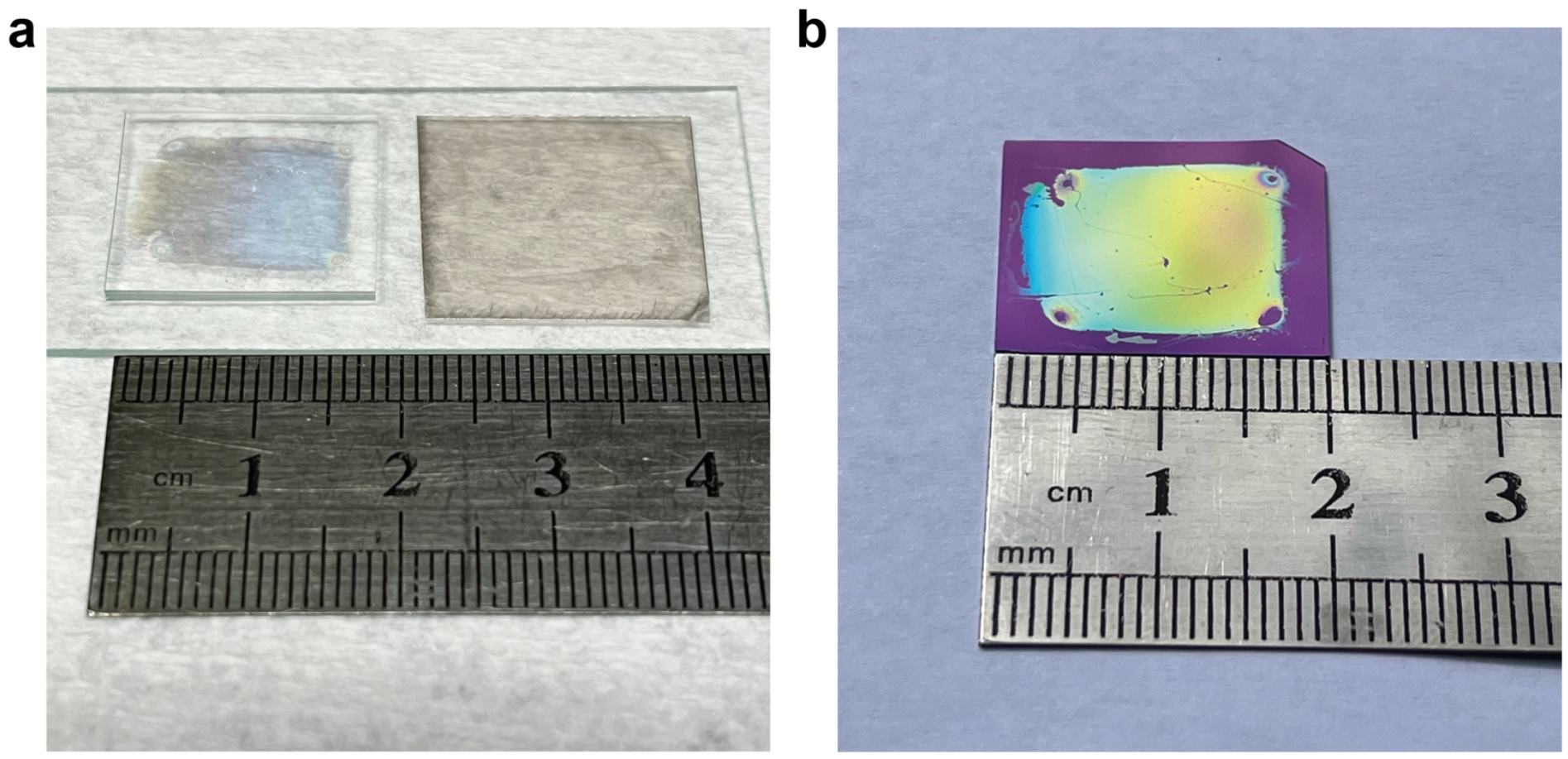

Fig. R20 Image of the MoO_3 film during the transfer process. a, MoO_3 film on a home-made PDMS stamp and mica substrate after the water-assisted transfer. **b,** The MoO_3 film on Si/SiO_2 substrate after dry-transfer from the PDMS stamp.

3. The oxygen vacancies in MoO3 will greatly affect its conductivity, and MoOx is usually considered as a hole transport layer or anode buffer layer in the perovskite device. Characterization of the oxygen stoichiometry and electrical conductivity of the MoO3 film would help to confirm its suitability as a dielectric.

Response: We thank you for this important suggestion. We fully agree that the oxygen stoichiometry is critical because the oxygen vacancies can introduce the electronic states and significantly increase conductivity, which would compromise the role of MoO_3 as a dielectric. To directly evaluate the oxygen stoichiometry, we performed X-ray photoelectron spectroscopy (XPS) on our as-grown MoO_3 film. The XPS spectra of the Mo 3d orbital and O ls orbital are presented in Fig. R21. The Mo 3d spectrum exhibits two

characteristic peaks located at 232.75 eV and 236.0 eV, which are assigned to Mo $3d_{5/2}$ and $3d_{3/2}$ of Mo 3d, respectively[17]. Meanwhile, the spectrum of O 1s is dominated by a single peak at ~530.1 eV, corresponding to lattice oxygen, with almost no obvious peak at ~531.6 eV that is typically associated with defective site oxygen[18]. The XPS results confirm that the as-grown film is stoichiometric MoO_3 with a low density of oxygen vacancies. For the electrical conductivity, we fabricated the in-plane MoO_3 device using Au as the electrodes, the result in Fig. R22 indicates that in-plane MoO_3 is insulating.

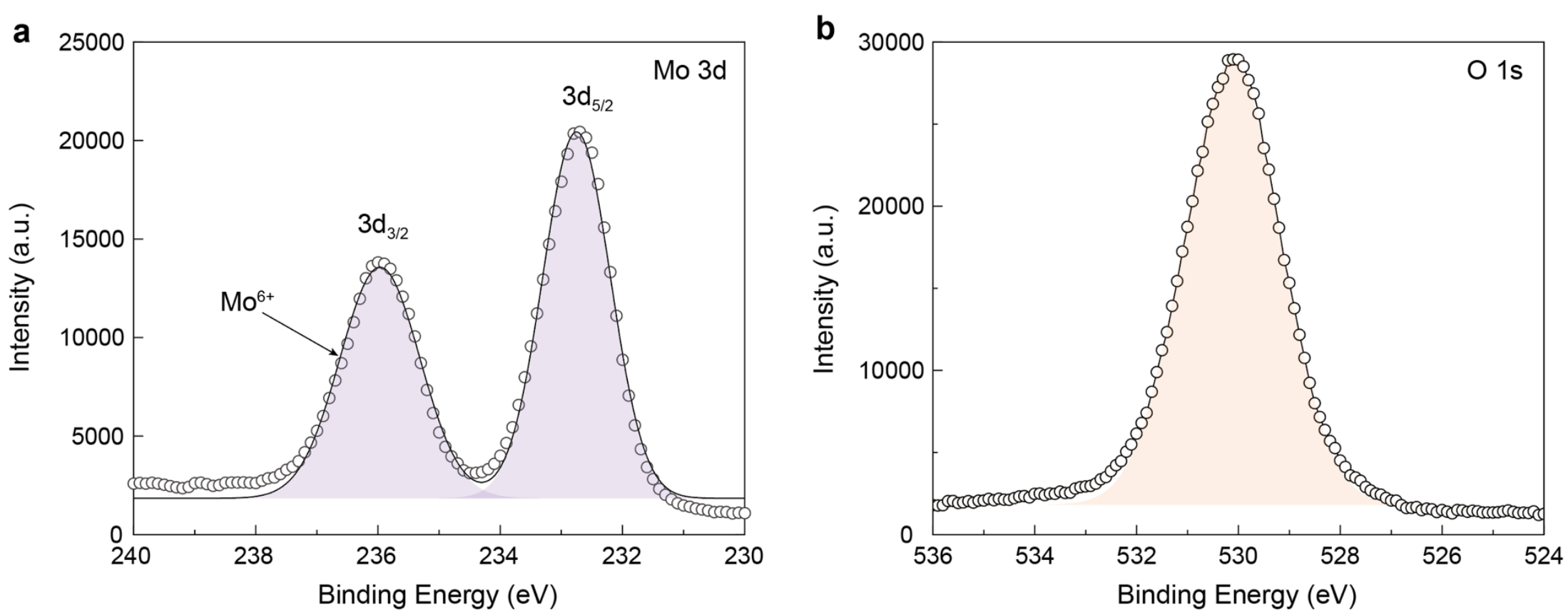

Fig. R21 XPS characterization of the grown MoO_3. a, the XPS spectra of Mo 3d. **b,** the XPS spectra of O 1s.

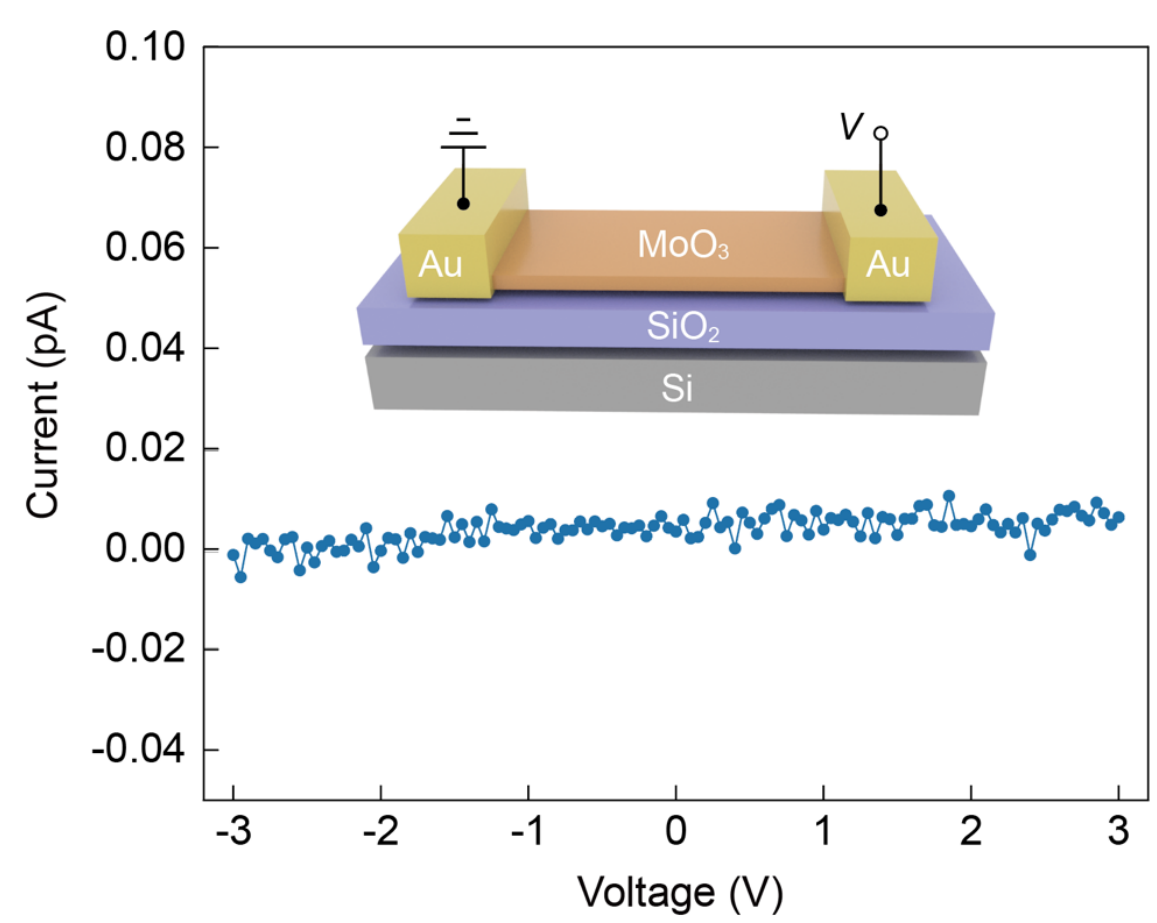

Fig. R22 *I-V* characteristic of a MoO_3 in-plane device. Inset is the schematic illumination of the device structure.

Changes made to the manuscript:

1. The XPS results have been added to Fig. S11.

2 Line 173, "The XPS results in Fig. S11 confirm the as-grown film is stoichiometric MoO_3 with a low density of oxygen vacancies."

4. What is the dark gap in the cross-sectional HAADF-STEM image in Fig. 3b, which is observed between the MoO3 layers and the monolayer MoS2. Please clarify whether this gap is intrinsic, related to sample preparation, or indicative of imperfect interfacial contact.

Response: We appreciate your careful attention to the structural characterization in Fig. 3b. Regarding the observed black gap between the MoO_3 layers and 1L MoS_2 in the cross-sectional HAADF-STEM image (Fig. R23), it could originate from the following reasons: (1) As shown in Fig. R23, the corresponding bright-field image reveals no clear lattice structure in the gap region, suggesting the presence of residual contamination introduced during the transfer or FIB sample preparation. Such contamination can locally reduce the scattering intensity, giving rise to an apparent "black gap" in the HAADF image; (2) As further illustrated in Fig. R24, which presents both HAADF and bright-field images of a larger region, from which the zoomed-in image shown in Fig. R23 was obtained. Residues can be clearly identified on the FLG on the right side of the image. This residue leads to partial physical separation at the MoS_2/MoO_3 interface, further contributing to the dark contrast observed in the HAADF image. Therefore, we attribute the dark gap to the residual contamination or the partial physical separation at the interface.

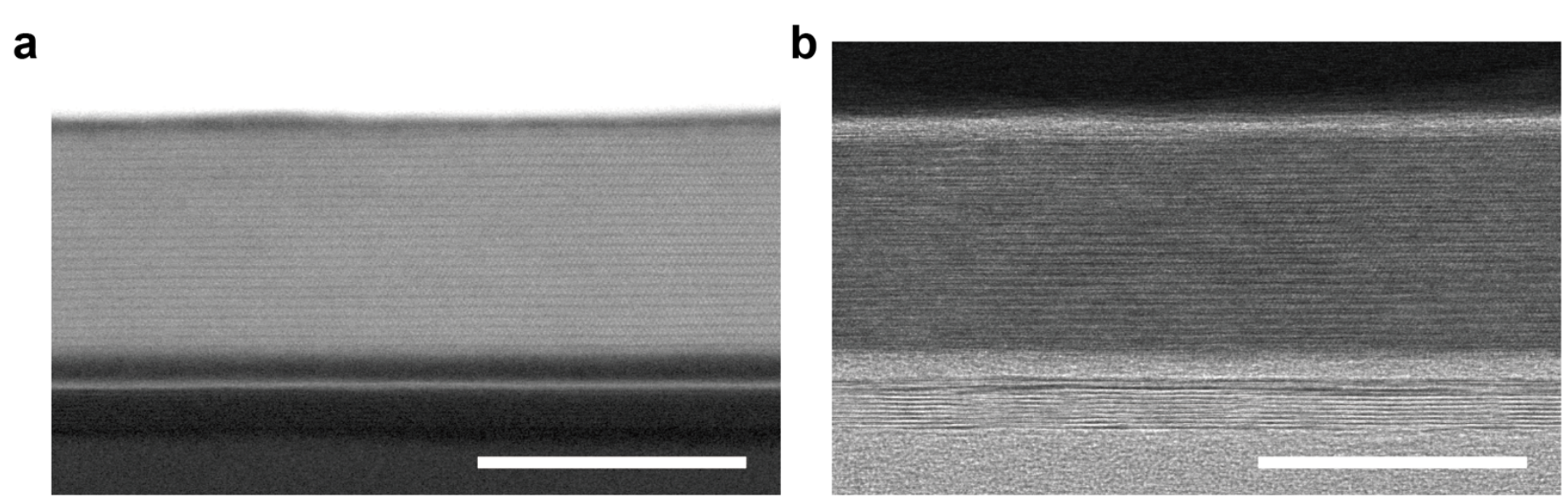

Fig. R23 (**a**) HAADF image and corresponding (**b**) bright-field image, scale bars are 20 nm.

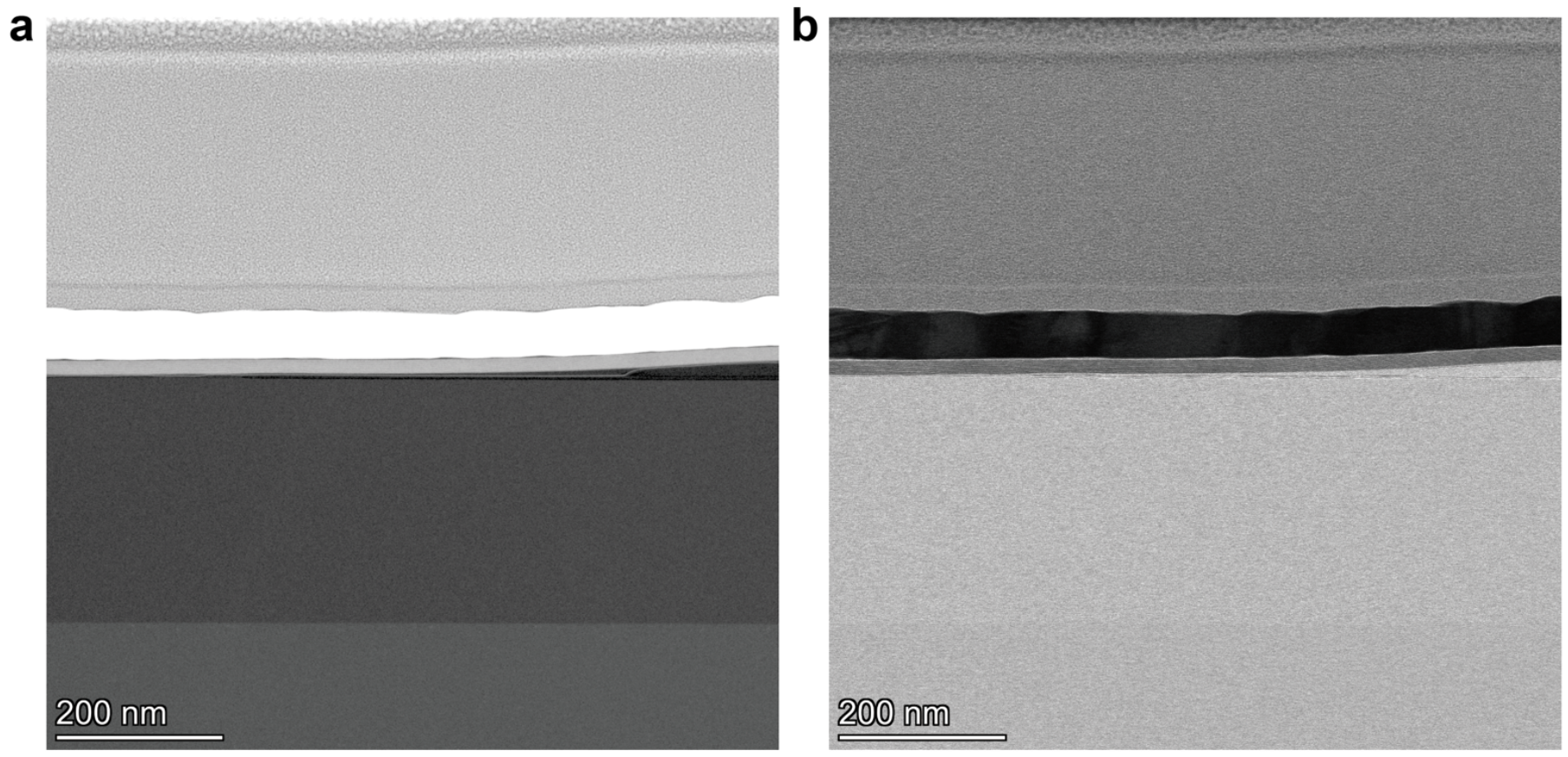

Fig. R24 (**a**) HAADF image and corresponding (**b**) bright-field image of a larger region.

5. What are the average values of the subthreshold swing (SS) and on/off current ratio for the FET device array? Providing the statistical averages (and preferably the standard deviation) in the main text would allow a clearer assessment of device uniformity and overall performance.

Response: We appreciate your great suggestion. Calculating the statistical averages of the SS and on/off ratio provides a more direct evaluation of device uniformity and overall array performance. Following your suggestion, we have added the average values and standard deviations of the SS and on/off ratios for the successful devices in the FET array. Specifically, the SS has an average value of ~103 mV dec^{-1}, with a standard deviation of ~27 mV dec^{-1}. For the on-off ratio, the average and standard deviation were calculated in logarithmic scale, yielding an average $\log_{10}(I_{on}/I_{off})$ value of ~6.8 and a standard deviation of ~0.59.

Changes made to the manuscript:

1. Line 369, "Specifically, the SS has an average value of ~103 mV dec^{-1}, with a standard deviation of ~27 mV dec^{-1}. For the on-off ratio, the average and standard deviation were calculated in logarithmic scale, yielding an average $\log_{10}(I_{on}/I_{off})$ value of ~6.8 and a standard deviation of ~0.59. The field-effect mobility extracted from the transfer curve is summarized in Fig. S37, with most devices exhibiting mobilities in the range from 5 to 20 cm^2 V^{-1}s^{-1}."

6. There is no color bar in Fig. 1f.

Response: Thank you for pointing out this mistake. We've added the corresponding color bar in the revised Fig.1f as shown below.

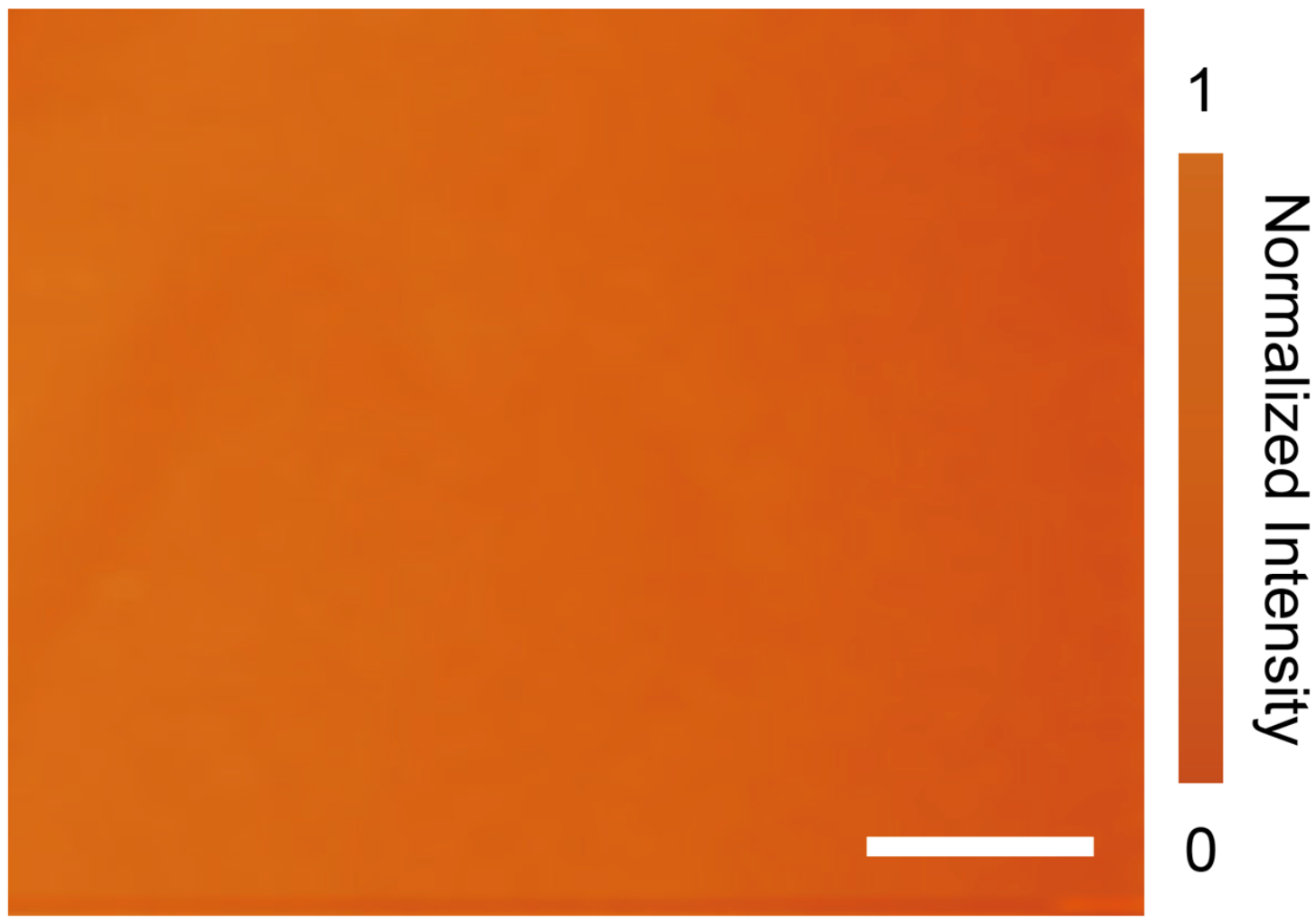

Fig. R25 Raman mapping (integration of peak 820 cm^{-1}) of the as-grown MoO_3 film. Scale bars, 100 μm.

Changes made to the manuscript:

1. Fig. 1f has been replaced by Fig. R25.

7. Better to indicate the EOT in Fig. 2g.

Response: Thank you for your valuable suggestion. In the revised manuscript, we have added the EOT values and corresponding physical thickness to Fig.2g as shown below and clarified them in the figure caption.

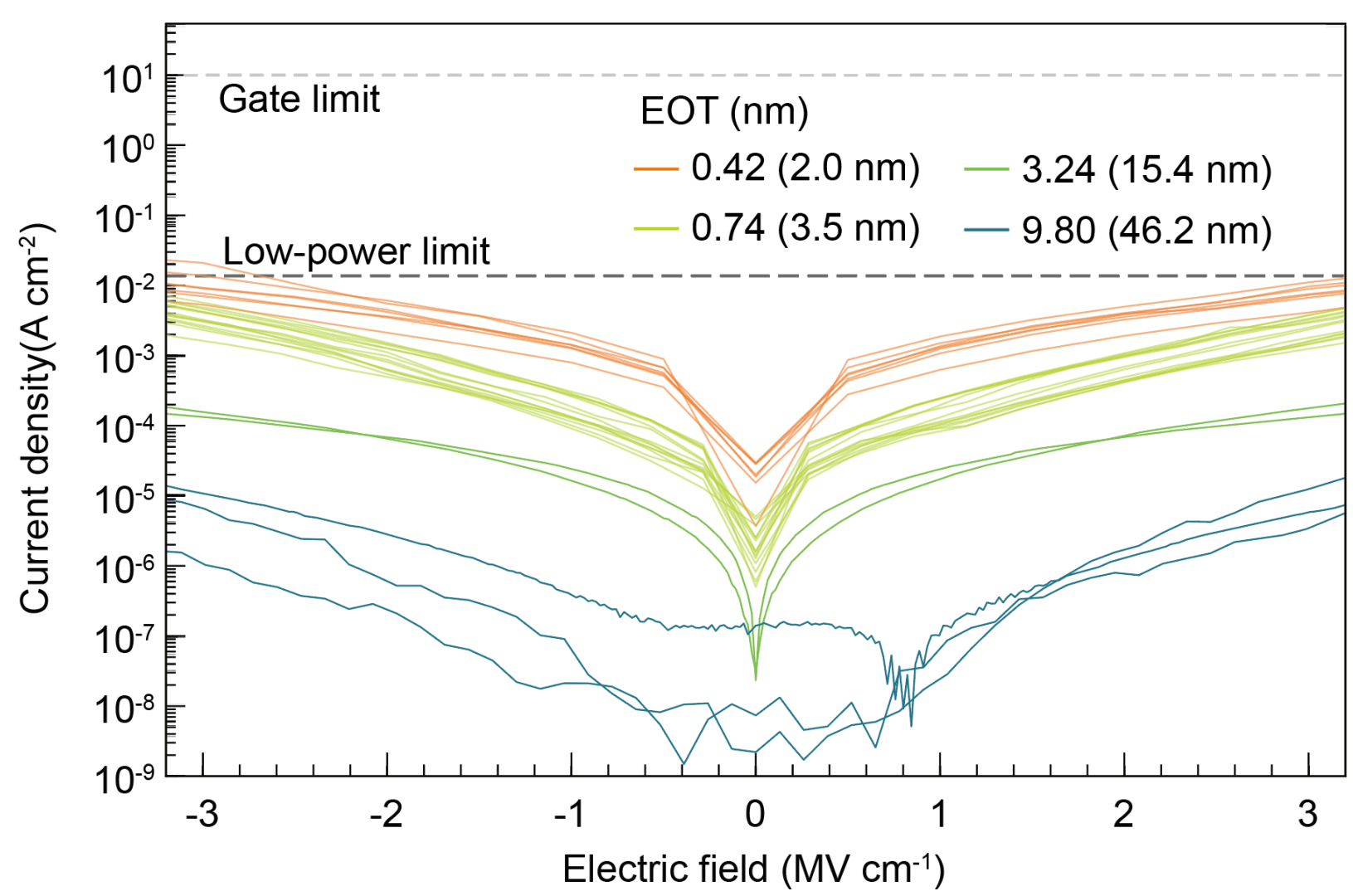

Fig. R26 Leakage current density of single-crystalline MoO_3 with different EOT values as a function of applied electric field.

Changes made to the manuscript:

1. Fig. 2g has been replaced by Fig. R25.

8. In Fig. S29, please clearly indicate which branch corresponds to the forward sweep and which corresponds to the backward sweep.

Response: Thank you for your valuable suggestion. To remove the ambiguity in interpreting the hysteresis behavior, we have updated Fig.S29 in the revised manuscript to explicitly label the forward and backward sweep branches in the figure, as shown below.

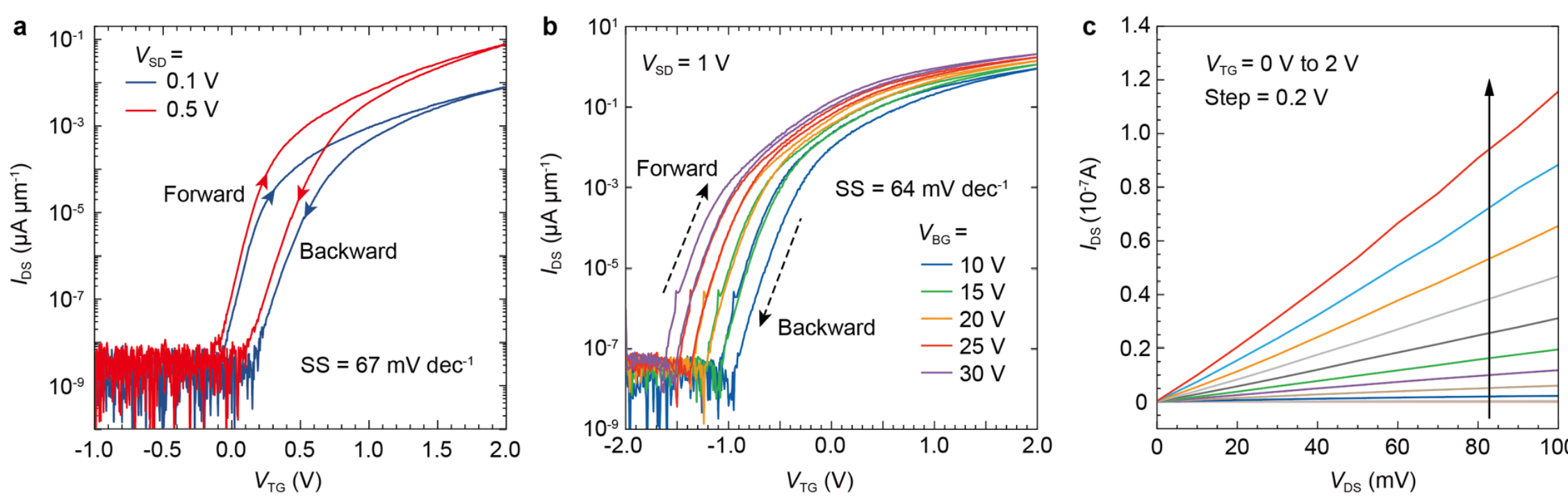

Fig. R27 Hysteretic behavior and Ohmic contact of the FET device.

Changes made to the manuscript:

1. Fig. S28 has been replaced by Fig. R27.

9. In Fig. S33, it would be nice to plot the hysteresis explicitly as a function of the sweep rate or the voltage step.

Response: Thank you for your great suggestion. Following your recommendation, we have explicitly extracted the hysteresis width and plotted it as a function of both sweep rate and voltage step in the revised supporting information Fig. S33, as now shown in Fig. R28. Overall, the hysteresis remains relatively small. Additionally, it shows a weak increasing trend with increasing sweep in the tested range and an obvious increase with the increasing voltage step. It is reasonable because a faster sweep usually gives less time for trap related chage process to fully equilibrate with the applied gate voltage, which leads to an offset between the forward and backward sweeps. In addition, a large voltage step not only

corresponds to a faster sweep rate but also reduces the voltage resolution in the extraction, which can further enlarge the hysteresis width.

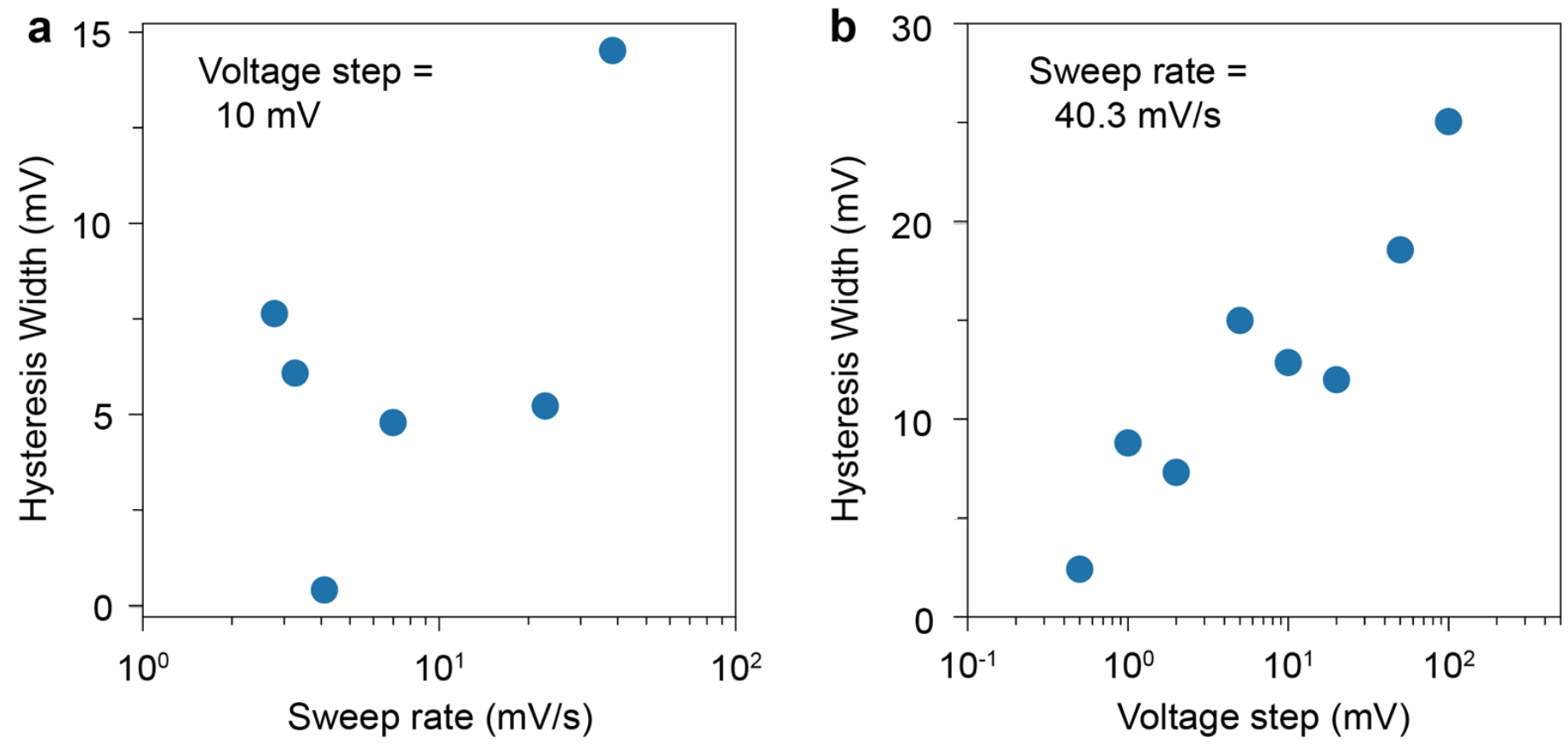

Fig. R28 Hysteresis width as a function of sweep rate and voltage step at I_{SD} = 10^{-9} A.

Changes made to the manuscript:

1. Fig. R28 has been added to Fig. S30 (Fig. S33 in the original version).
2. In the caption of Fig. S30, the description of hysteresis was added as "Overall, the hysteresis remains relatively small. Additionally, it shows a weak increasing trend with increasing sweep in the tested range and an obvious increase with the increasing voltage step. It is reasonable because a faster sweep usually gives less time for trap related chage process to fully equilibrate with the applied gate voltage, which leads to an offset between the forward and backward sweeps. In addition, a large voltage step not only corresponds to a faster sweep rate, but also reduces the voltage resolution in the extraction, which can further enlarge the hysteresis width."

References

1. Zhang, Z. *et al.* Highly Selective Synthesis of Monolayer or Bilayer WSe2 Single Crystals by Pre-annealing the Solid Precursor. *Chem. Mater.* **33**, 1307–1313 (2021).
2. Chen, J. *et al.* A Layered Wide-Bandgap BiOF Gate Dielectric with a High Dielectric Constant. *ACS Nano* **20**, 9722–9732 (2026).

3. Robertson, J. Electronic Structure and Band Offsets of High-Dielectric-Constant Gate Oxides. *MRS Bulletin* **27**, 217–221 (2002).

4. Yin, L. *et al.* High-κ monocrystalline dielectrics for low-power two-dimensional electronics. *Nat. Mater.* **24**, 197–204 (2025).

5. Zhang, J. *et al.* Super-tetragonal Sr4Al2O7 as a sacrificial layer for high-integrity freestanding oxide membranes. *Science* **383**, 388–394 (2024).

6. Ahn, J.-H., Parkin, W. M., Naylor, C. H., Johnson, A. T. C. & Drndić, M. Ambient effects on electrical characteristics of CVD-grown monolayer MoS2 field-effect transistors. *Sci. Rep.* **7**, 4075 (2017).

7. Zhu, C.-Y. *et al.* Magnesium niobate as a high-κ gate dielectric for two-dimensional electronics. *Nat. Electron.* **7**, 1137–1146 (2024).

8. Wang, L. *et al.* A general one-step plug-and-probe approach to top-gated transistors for rapidly probing delicate electronic materials. *Nat. Nanotechnol.* **17**, 1206–1213 (2022).

9. Groeseneken, G., Maes, H. E., Beltran, N. & De Keersmaecker, R. F. A reliable approach to charge-pumping measurements in MOS transistors. *IEEE Trans. Electron Devices* **31**, 42–53 (1984).

10. Zeng, D. *et al.* Single-crystalline metal-oxide dielectrics for top-gate 2D transistors. *Nature* **632**, 788–794 (2024).

11. Lu, Z. *et al.* Wafer-scale high-κ dielectrics for two-dimensional circuits via van der Waals integration. *Nat. Commun.* **14**, 2340 (2023).

12. Nicollian, E. H. & Brews, J. R. *MOS (Metal Oxide Semiconductor) Physics and Technology*. (John Wiley & Sons, 2002).

13. Taniguchi, K., Fang, N. & Nagashio, K. Direct observation of electron capture and emission processes by the time domain charge pumping measurement of MoS2 FET. *Appl. Phys. Lett.* **113**, (2018).

14. Jain, A. *et al.* One-Dimensional Edge Contacts to a Monolayer Semiconductor. *Nano Lett.* **19**, 6914–6923 (2019).

15. Guo, Y. *et al.* A towards-foundry strategy for creating fully interconnected two-dimensional microprocessors. *Nat. Electron.* **9**, 159–169 (2026).

16. Zhang, X. *et al.* Reliable wafer-scale integration of two-dimensional materials and metal electrodes with van der Waals contacts. *Nat. Commun.* **15**, 4619 (2024).

17. Borgschulte, A. *et al.* Hydrogen reduction of molybdenum oxide at room temperature. *Sci Rep* **7**, 40761 (2017).

18. Guha, P. *et al.* Defect-Engineered MoO2 Nanostructures as an Efficient Electrocatalyst for Oxygen Evolution Reaction. *ACS Appl. Energy Mater.* **3**, 5208–5218 (2020).

Point-by-point response to reviewers' comments for manuscript ID: NCOMMS-25-102445A

General response: We are grateful to all reviewers for their recommendation of the acceptance of our work. We've revised the manuscript according to your comments. The original comments are in orange, our responses are in black, and the corresponding revisions in the updated manuscript are highlighted in red.

Reviewer #1 (Remarks to the Author):

The authors have addressed my questions satisfactorily. The revised version has been improved greatly. I recommend its publication in Nature Communications after the minor textual modification was made.

Response: We thank you for your satisfaction and recommendation for the acceptance.

Minor comment

1) The authors declared that "a low equivalent oxide thickness (EOT) ~0.42 nm (physical 48 thickness ~2.0 nm)" in the abstract. However, the EOT value was not supported by the direct capacitance measurement. To be more rigorous, suggest the authors remove the claim of 0.42-nm EOT in the Abstract.

Response: We sincerely thank you for your advice. We have removed the claim of EOT in the abstract and revised the abstract.

Action: The abstract has been changed to "Large-area high-quality dielectrics are essential for the integration of two-dimensional (2D) transistors. However, this integration remains challenging, especially for conventional dielectrics that require deposition processes. Adopting van der Waals (vdW) dielectrics is attractive. Molybdenum trioxide (α-MoO_3) has been demonstrated as a vdW dielectric in 2D field-effect transistors (FETs), but previous demonstrations showed limited performance and small size. Here, we grow large-area single-crystalline MoO_3 with uniform thickness and explore its properties as a dielectric. Single-crystalline MoO_3 reaches the millimeter scale and maintains a high dielectric constant of ~18.5, low gate leakage of approximately 10^{-2} A cm^{-2} at 3.0 MV cm^{-1}, and a large breakdown field of ~26.8 MV cm^{-1}. Top-gated molybdenum disulfide (MoS_2) transistors exhibit an average subthreshold swing of ~71 mV dec^{-1}, and an on/off current

ratio exceeding 10^8. Furthermore, we demonstrate MoO_3-based integrated circuits and low-power complementary metal-oxide-semiconductor (CMOS) inverters."

Reviewer #3 (Remarks to the Author):

I think the authors has made great improvements on the manuscript and well addressed all of my concerns. Their responses to other reviewers' comments are also comprehensive and convincing. It can be accepted for publication.

Response: We thank you for your satisfaction and recommendation for the acceptance.
